# Supplementary material for: Tracking SARS-CoV-2 Spike Protein Mutations in the United States (January 2020—March 2021) Using a Statistical Learning Strategy
Source: Viruses. 2021 Dec 21;14(1):9. doi: 10.3390/v14010009 (PMC8777887; doi:10.3390/v14010009)
Supplement: Supplementary file 1 [file viruses-14-00009-s001.zip › viruses-1463499-supplementary.pdf]

Supplementary Materials for "Tracking SARS-CoV-2 Spike Protein Mutations in the United States (January 2020 – March 2021) Using a Statistical Learning Strategy" by Zhao et al.

Contents

Figure S1. For all Spike residues with sufficient variation, scatterplots of the maximum proportion (Pmax) of sequences from a given state/territory harboring a mutation at a given AA position vs. q-value...........2

Figure S2. Temporal patterns of VRVs identified in each state/territory 11

Figure S3. Locally averaged proportions over time for substitutions at 3 AA-subs in a VOI (Y144, F888, V1176) and at 3 AA-subs in a VOC (H69, Y144, K417) that were not detected by the SLS method in states/territories where at least three sequences had a substitution at the designated AA position 24

Figure S4. Presence of VRVs among all cases in each state/territory. 25

Table S1. Distribution of the 21,391 VOI/VOC sequences by specific variant and by state/territory. 32

Table S2. Distribution of the 167,893 SARS-CoV-2 sequences by state/territory and by GISAID submission month, along with state/territory-specific distribution of the 21,391 VOI/VOC excluded sequences. 33

Table S3. The 10 identified geo-VRV clusters (TP1 through TP10), based on temporal profiles. 34

Table S4. Frequencies of the 90 viral residue variants (VRVs) by state/territory, from an unsupervised learning from bi-clustering of all States and VRVs... 39

Table S5. VRV-haplotypes identified within each state/territory, along with state/territory-specific frequencies. The "positivity" column indicates the proportion of mutations in each haplotype block. 42

Table S6. List of individuals from the Originating laboratories responsible for obtaining the specimens and the Submitting laboratories where genetic sequence data were generated and shared via the GISAID Initiative, on which this research is based...49

Figure S1. For all Spike residues with sufficient variation, scatterplots of the maximum proportion (Pmax) of sequences from a given state/territory harboring a mutation at a given amino acid position vs. q-value.

Points in red represent residues that meet both criteria for classification as a VRV. Points in black represent residues that do not.

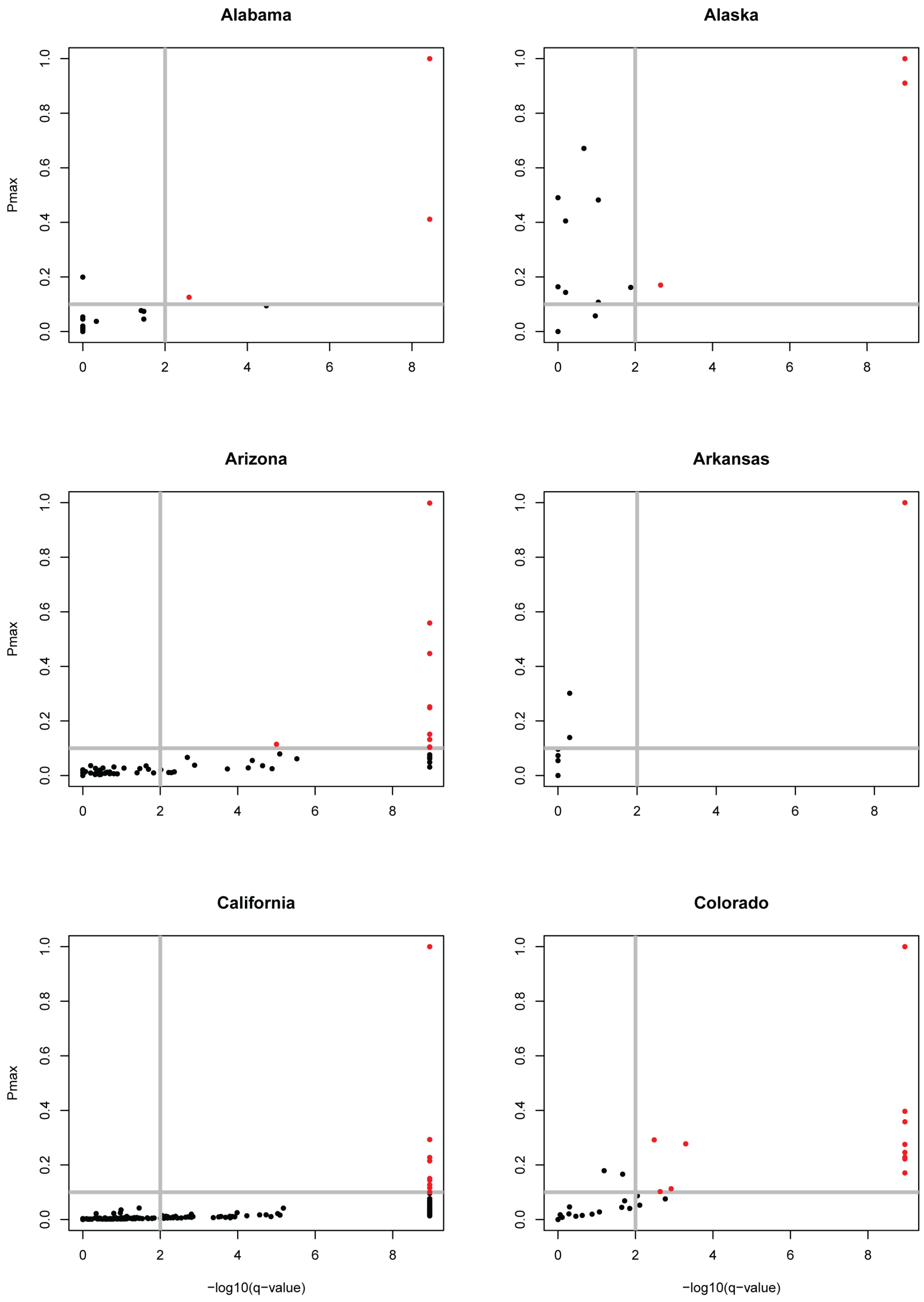

Figure S1 (continued).

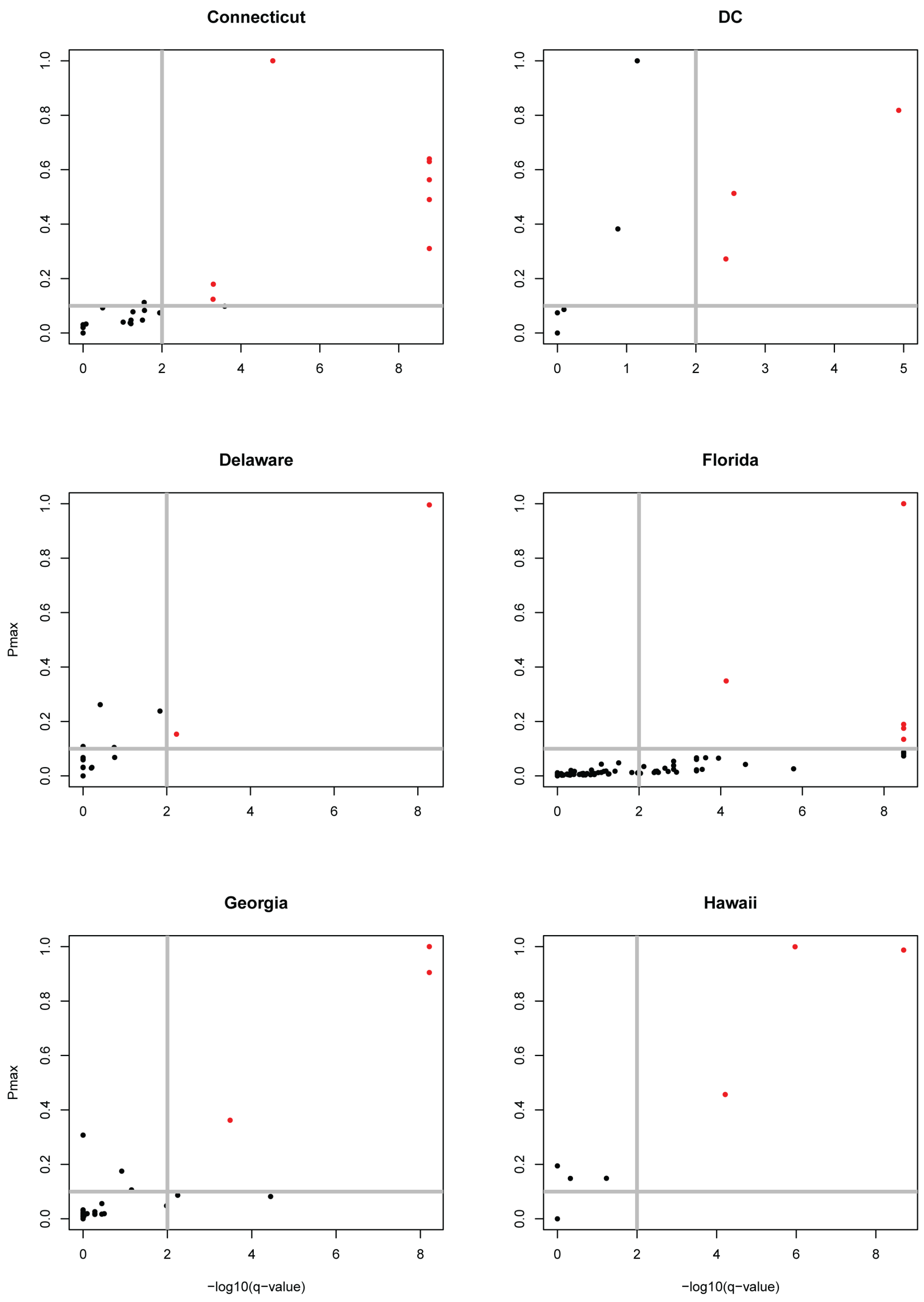

Figure S1 (continued).

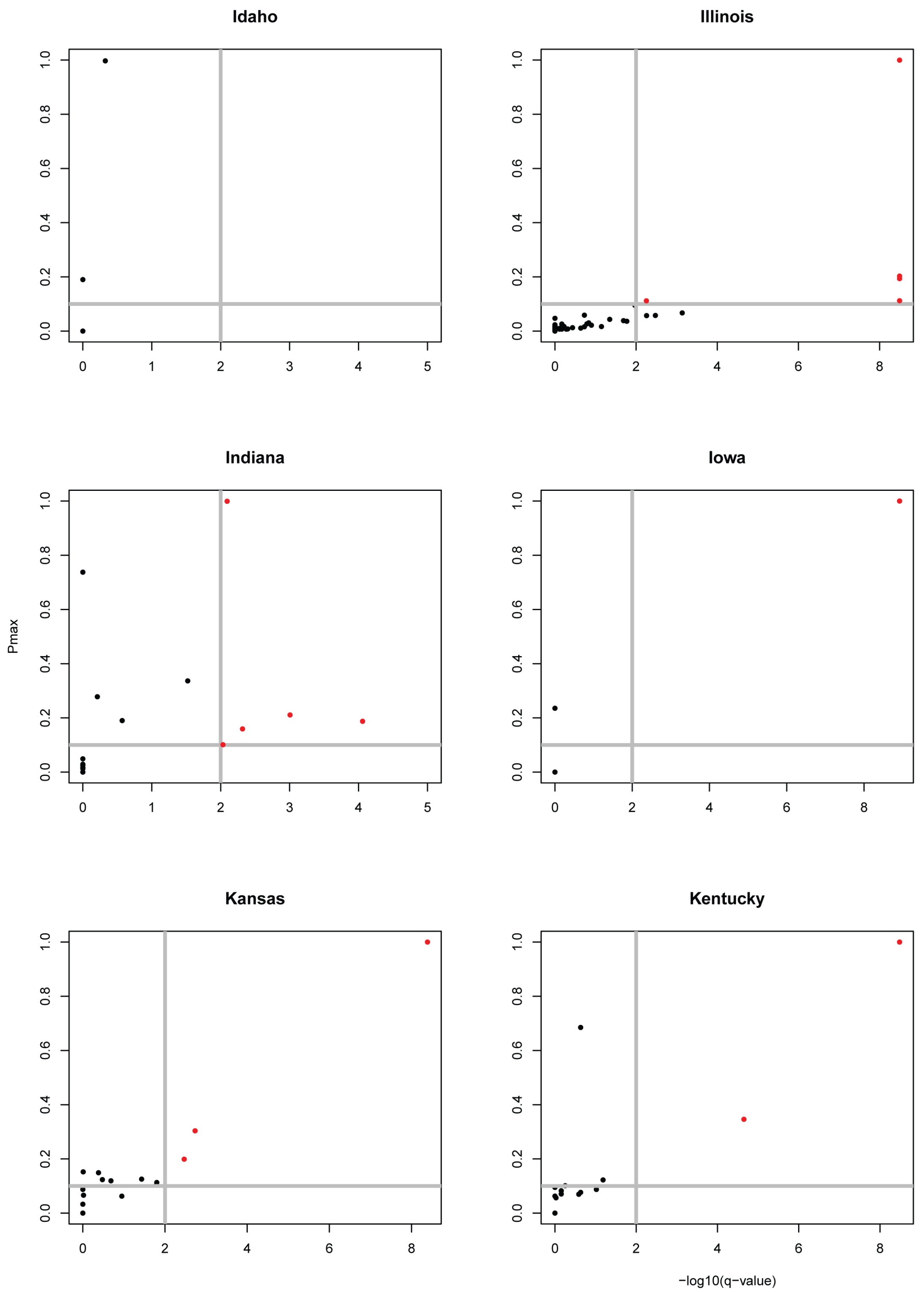

Figure S1 (continued).

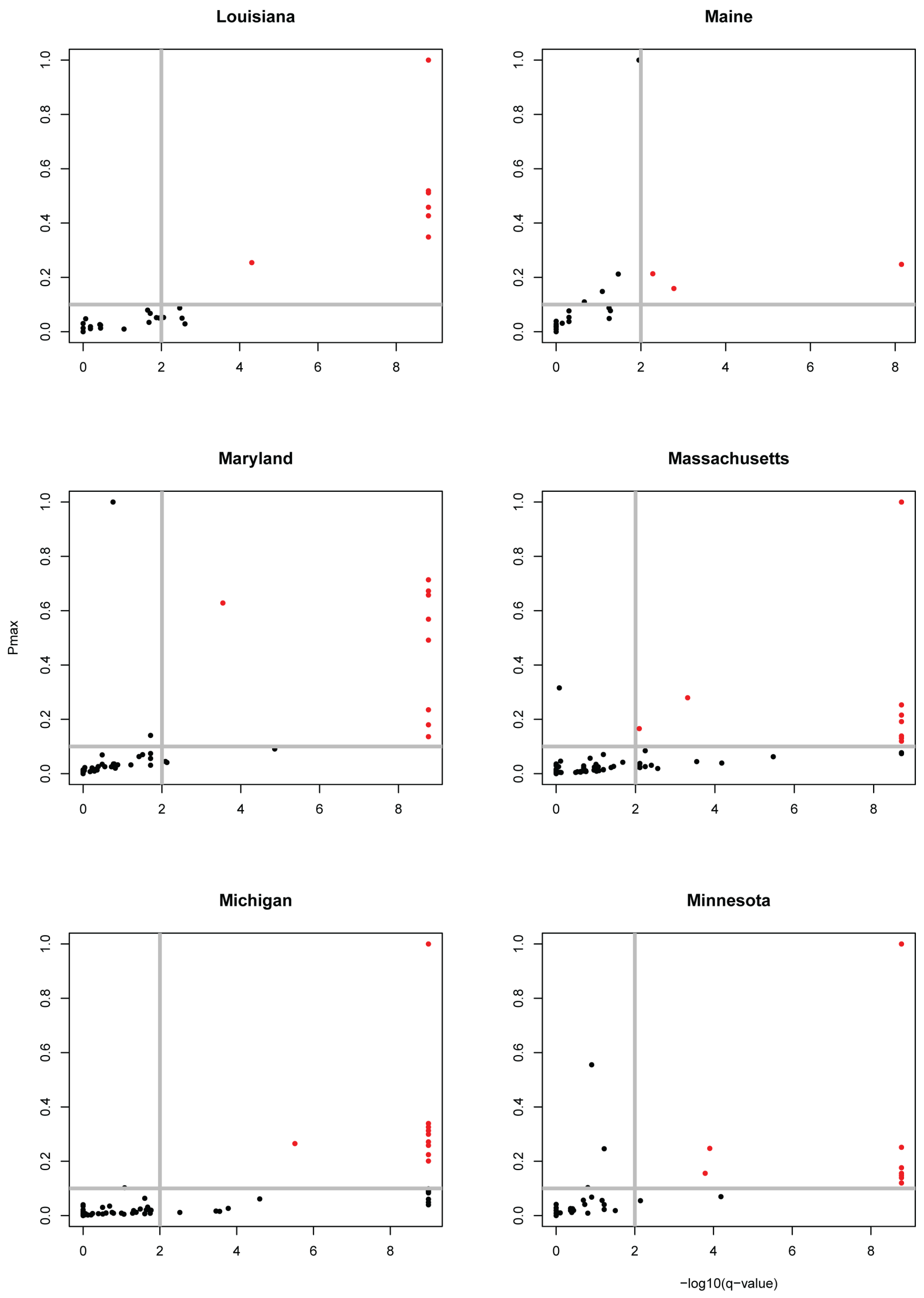

Figure S1 (continued).

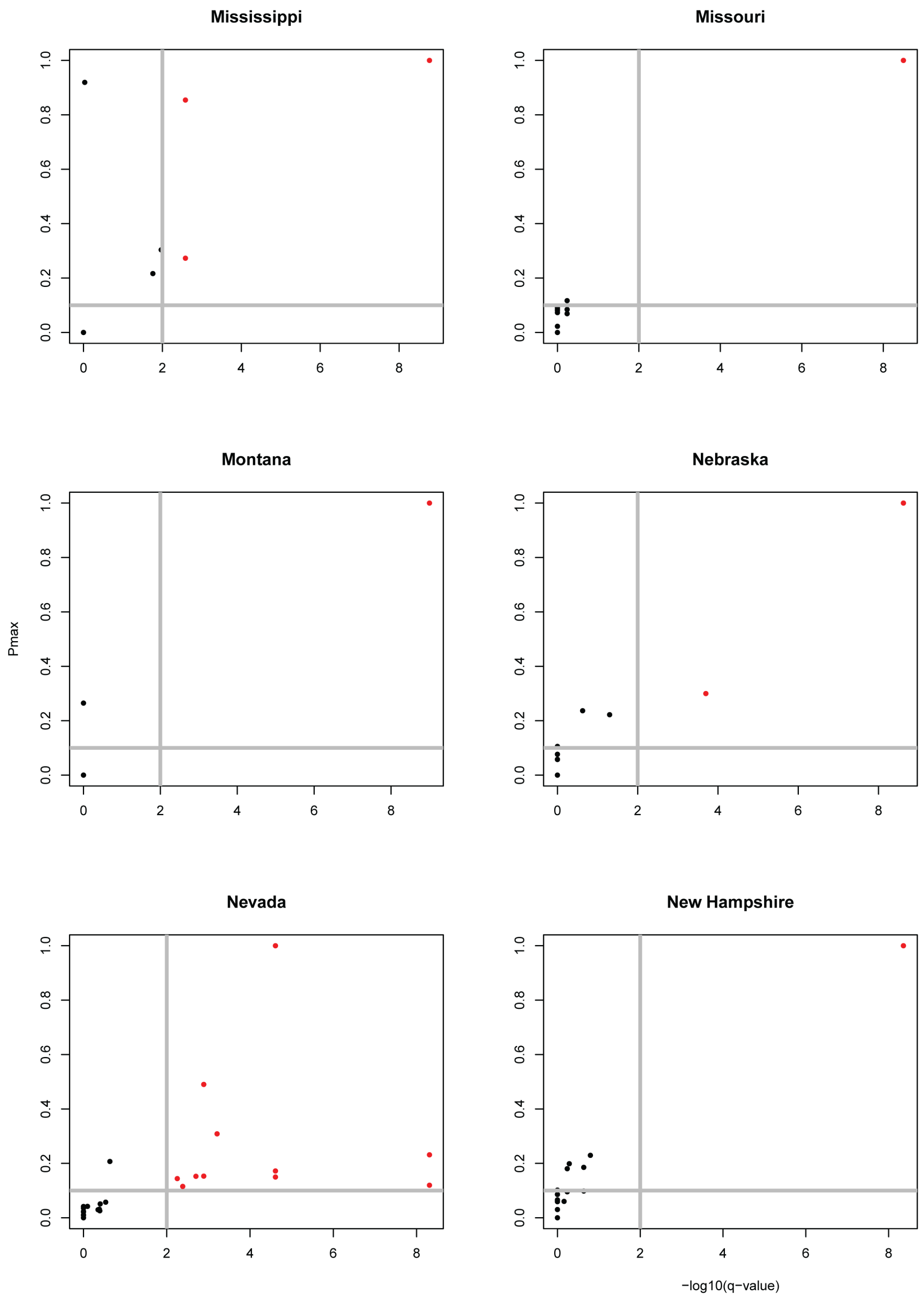

Figure S1 (continued).

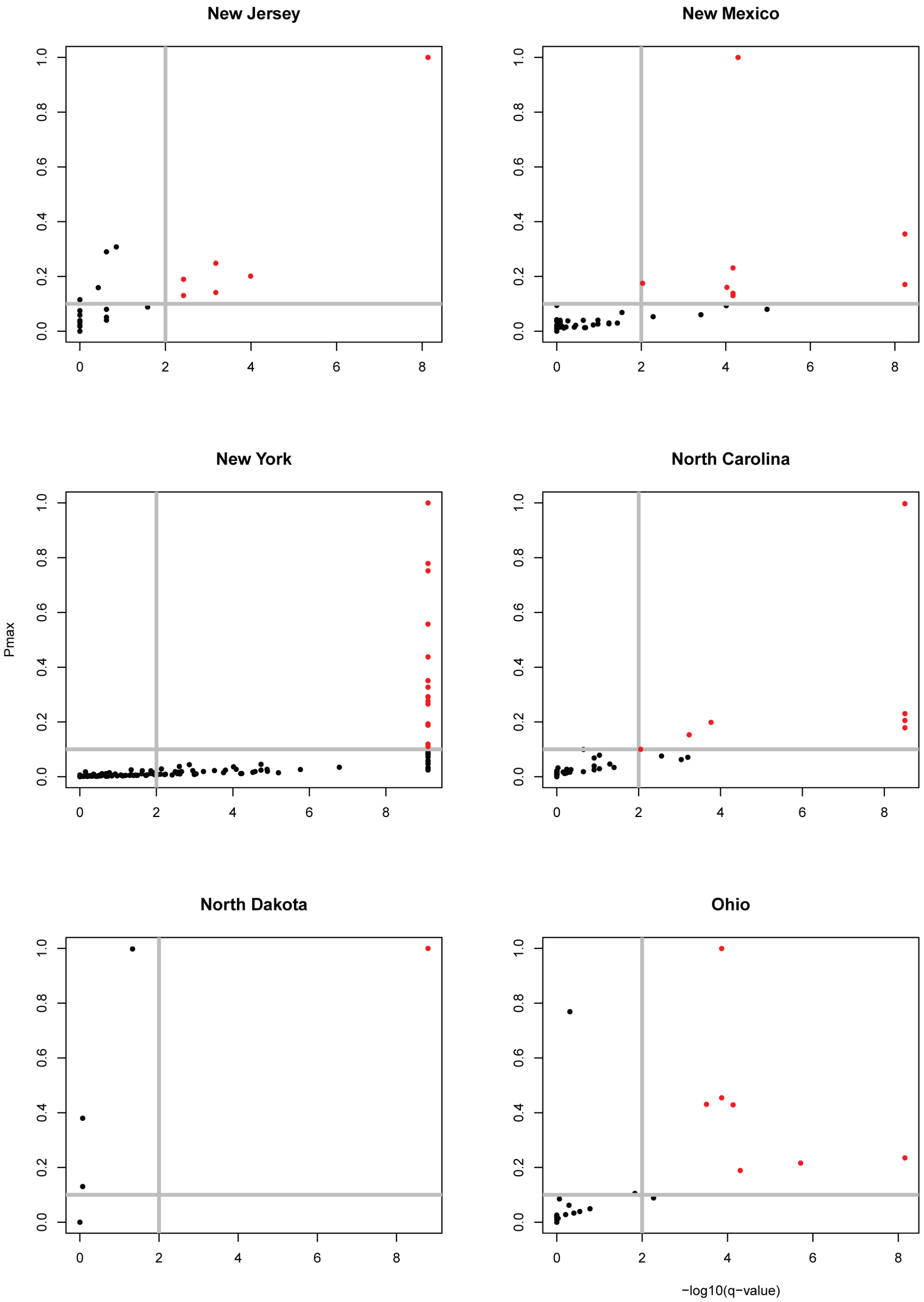

Figure S1 (continued).

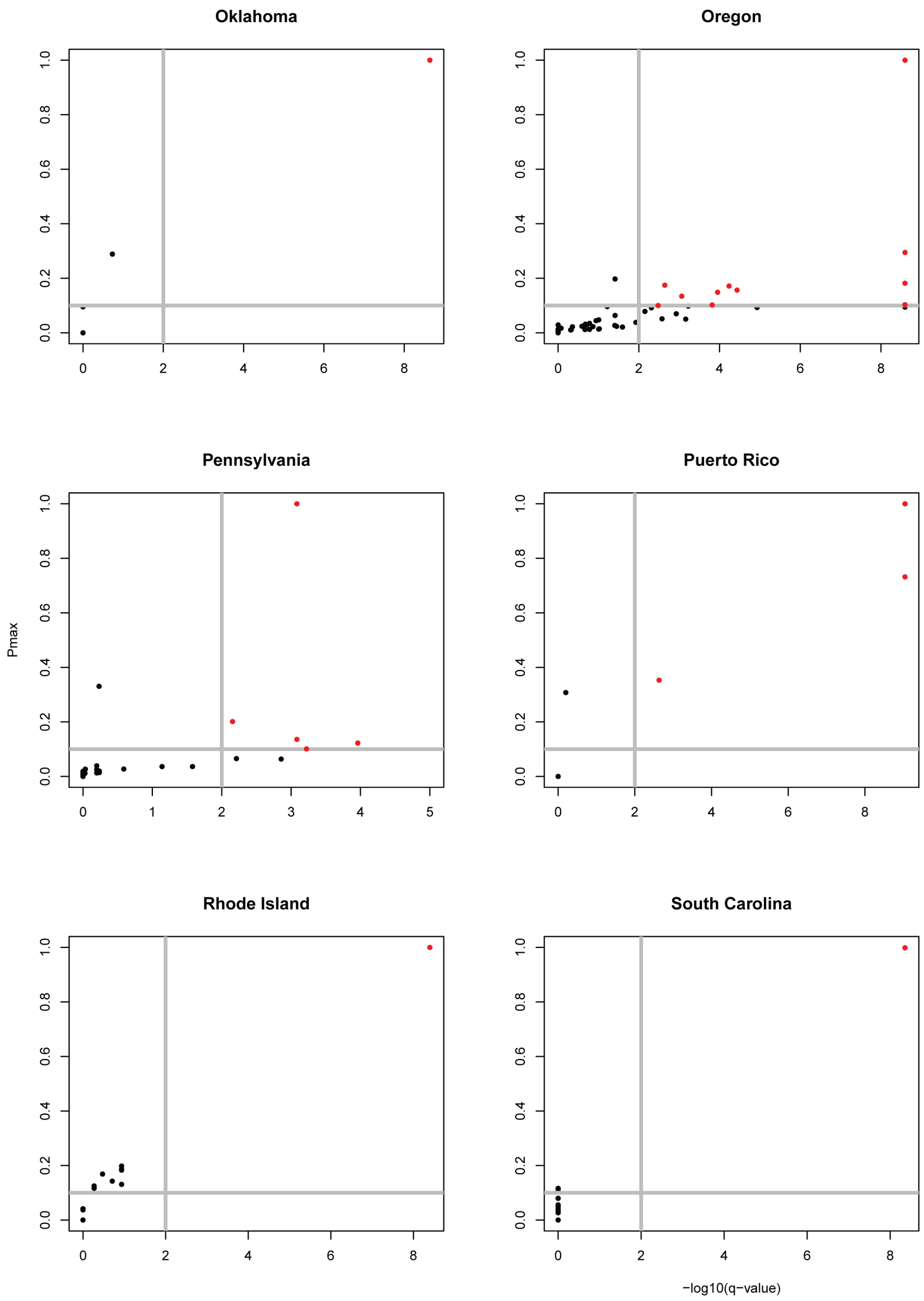

Figure S1 (continued).

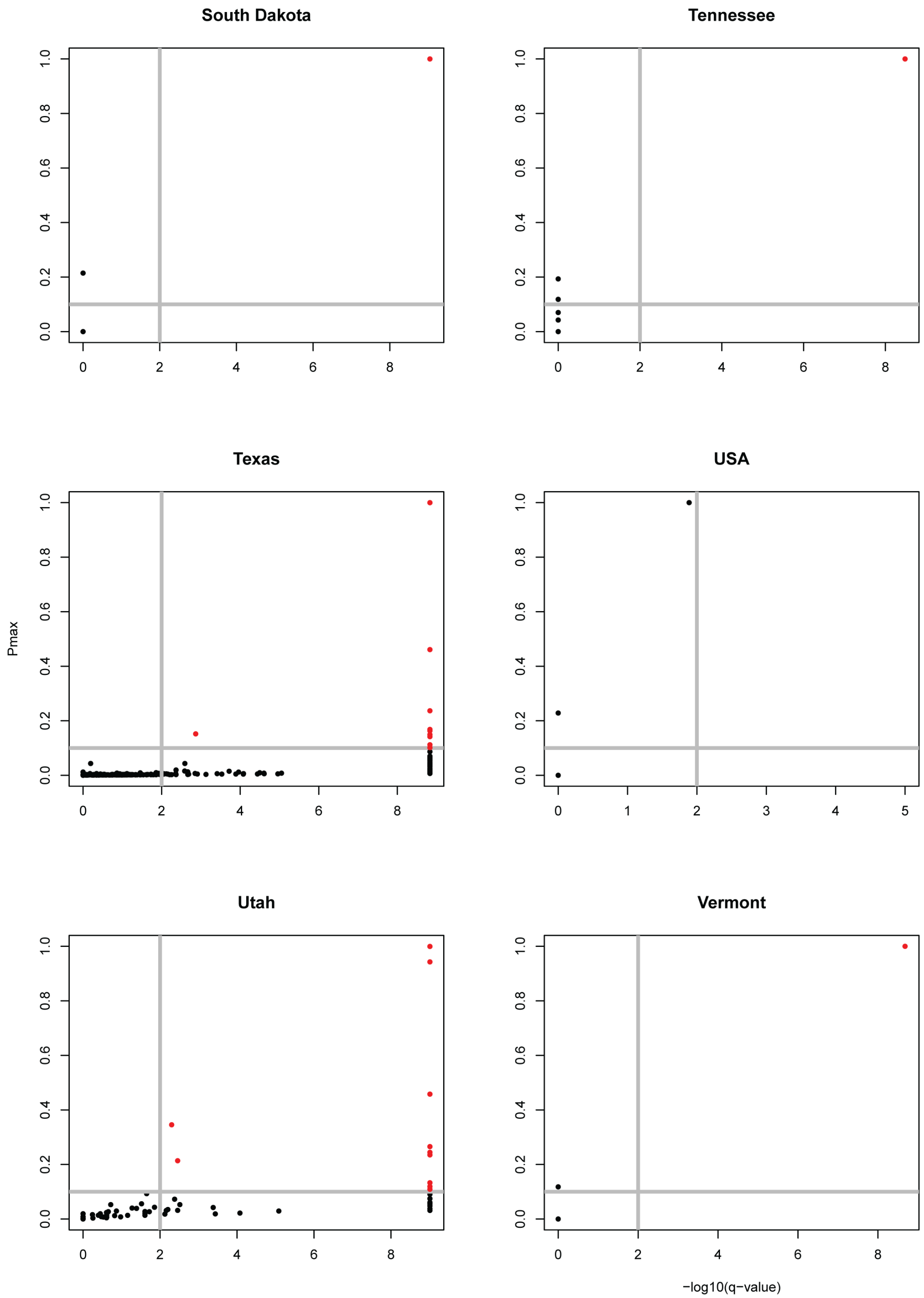

Figure S1 (continued).

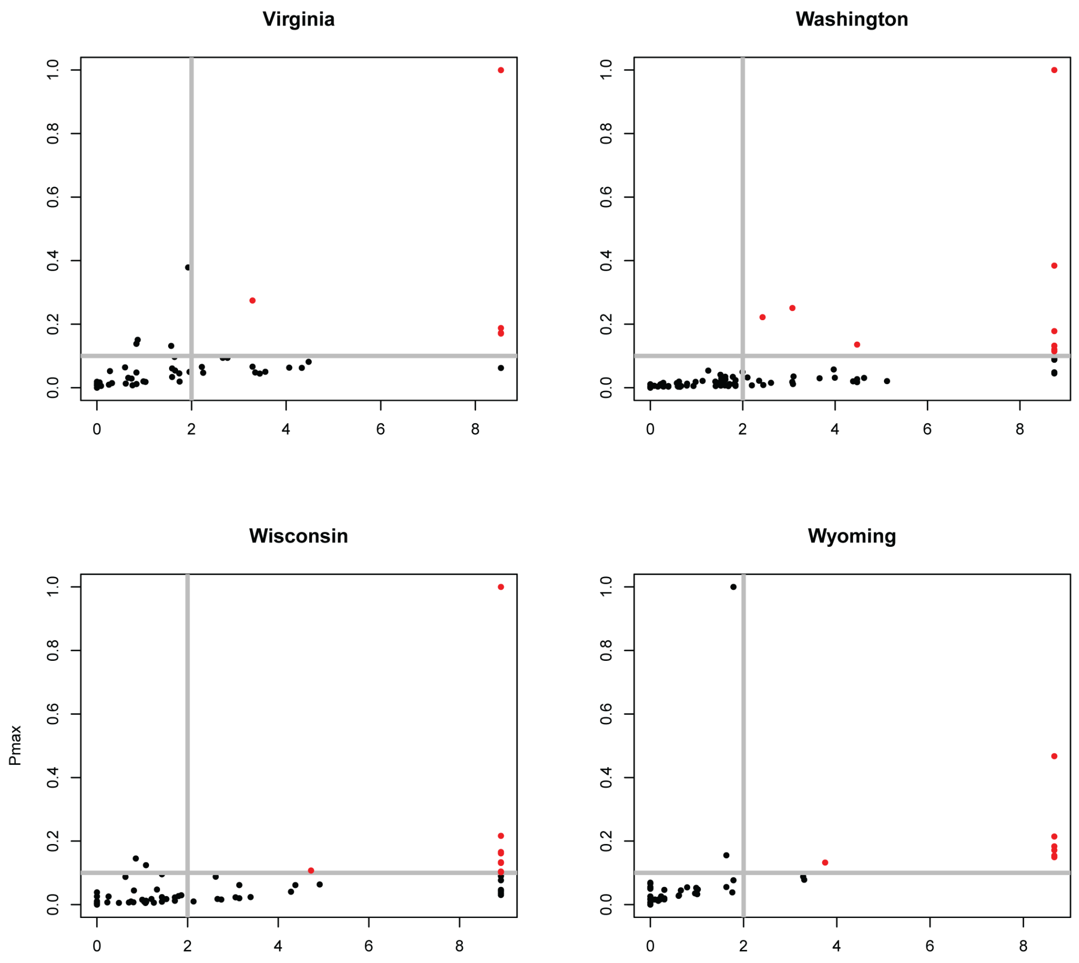

Figure S2. Temporal patterns of VRVs identified in each state/territory.

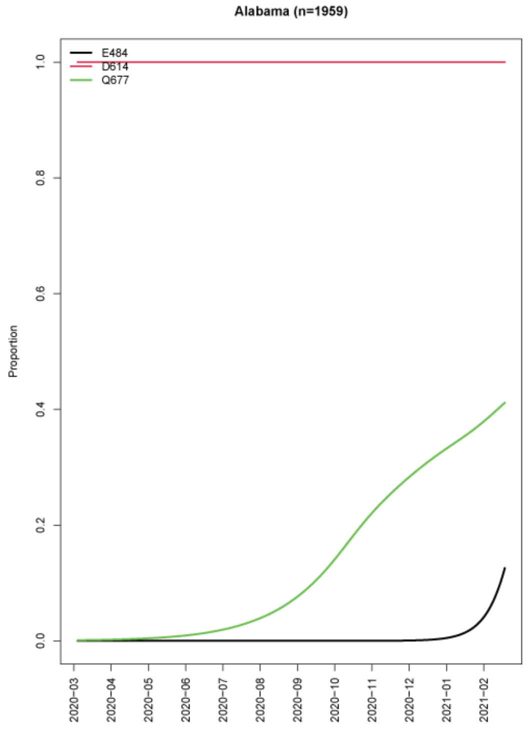

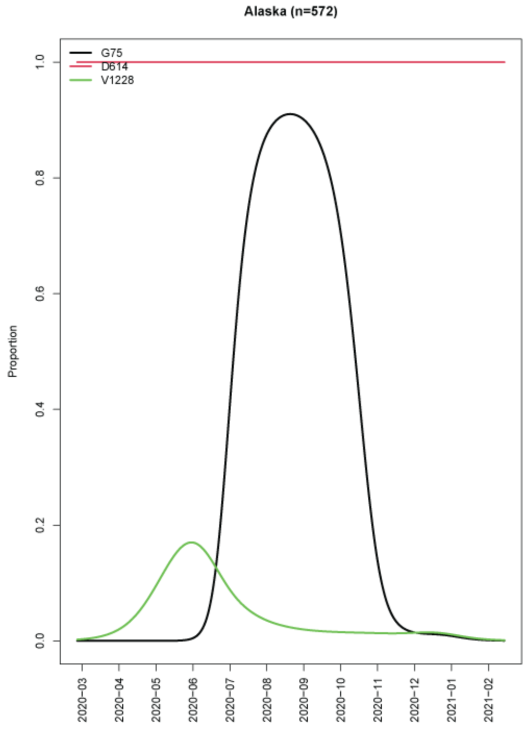

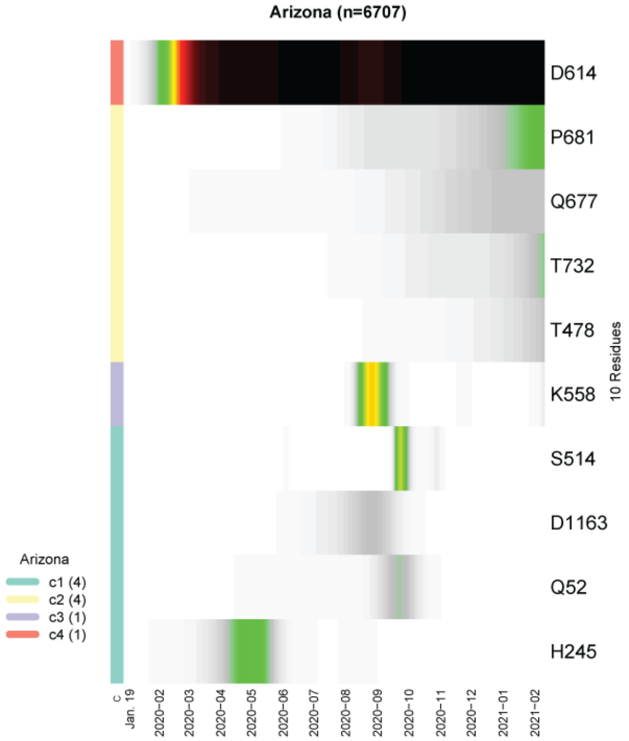

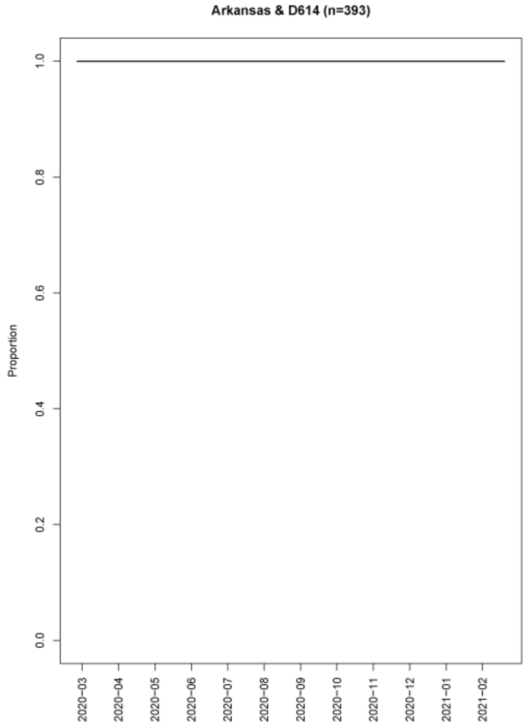

Figure S2 (continued).

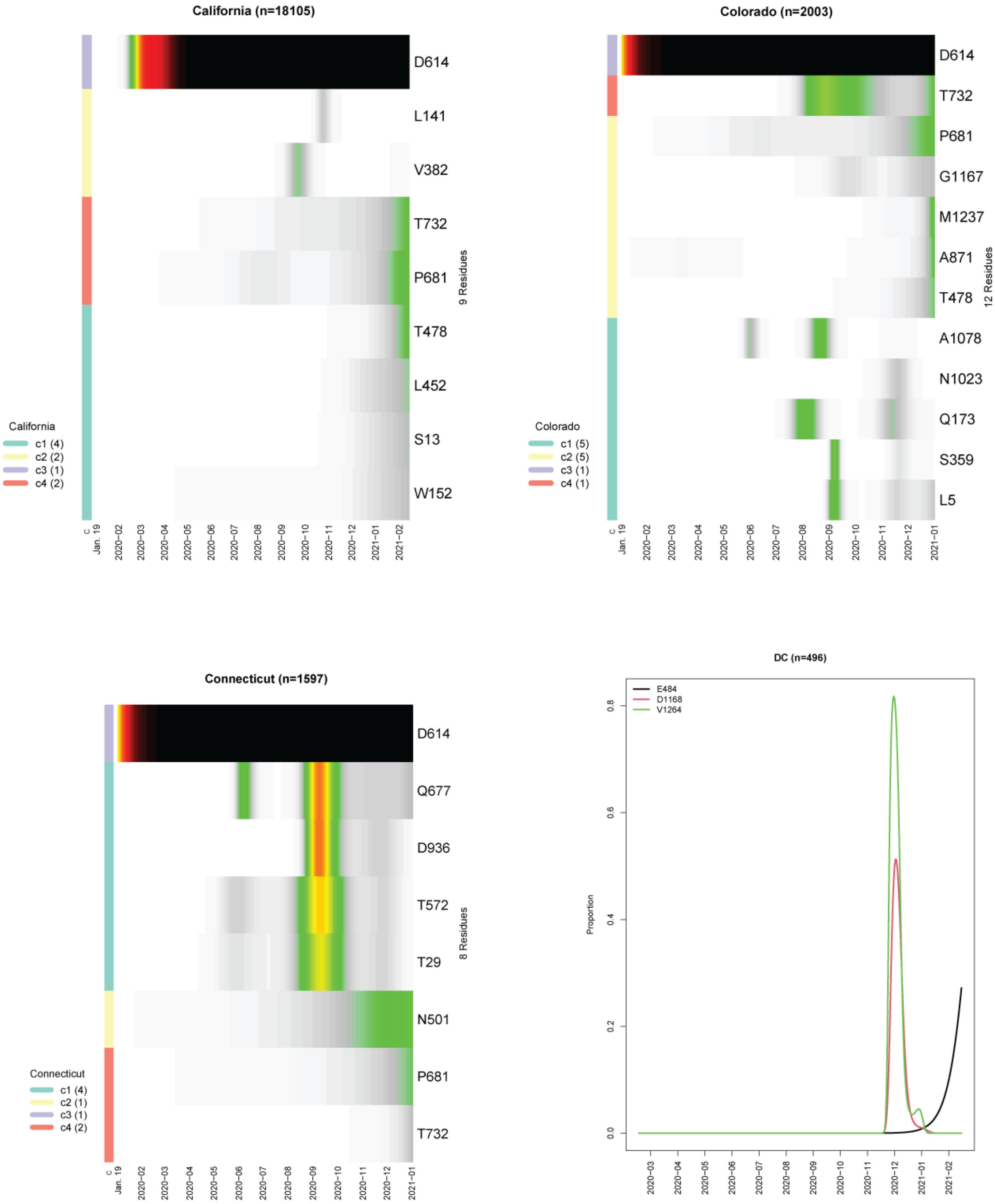

Figure S2 (continued).

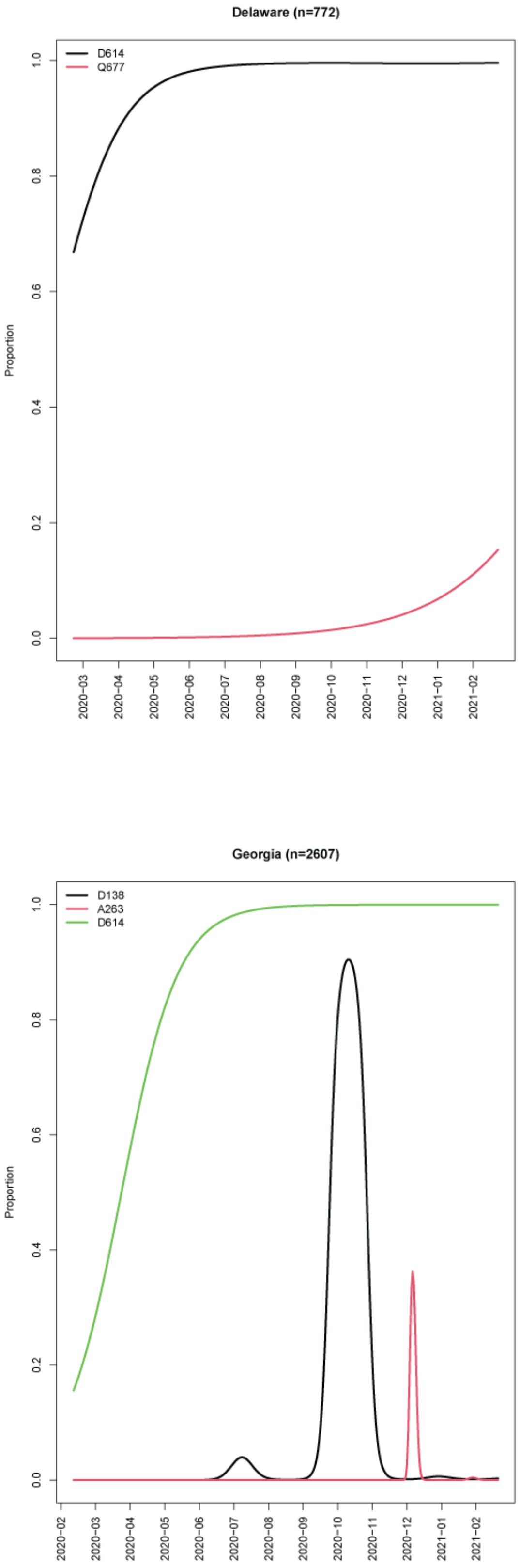

Figure S2 (continued).

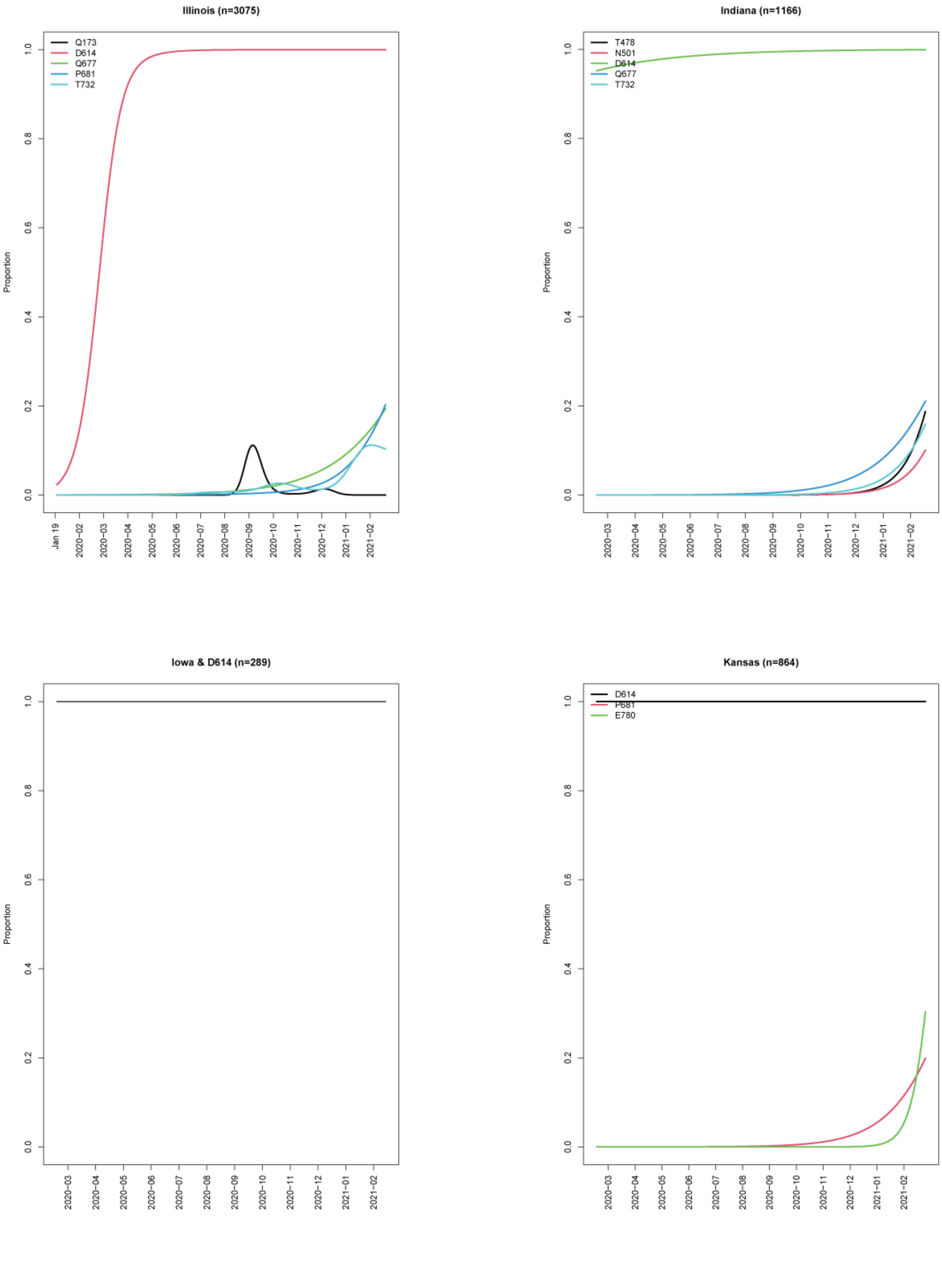

Figure S2 (continued).

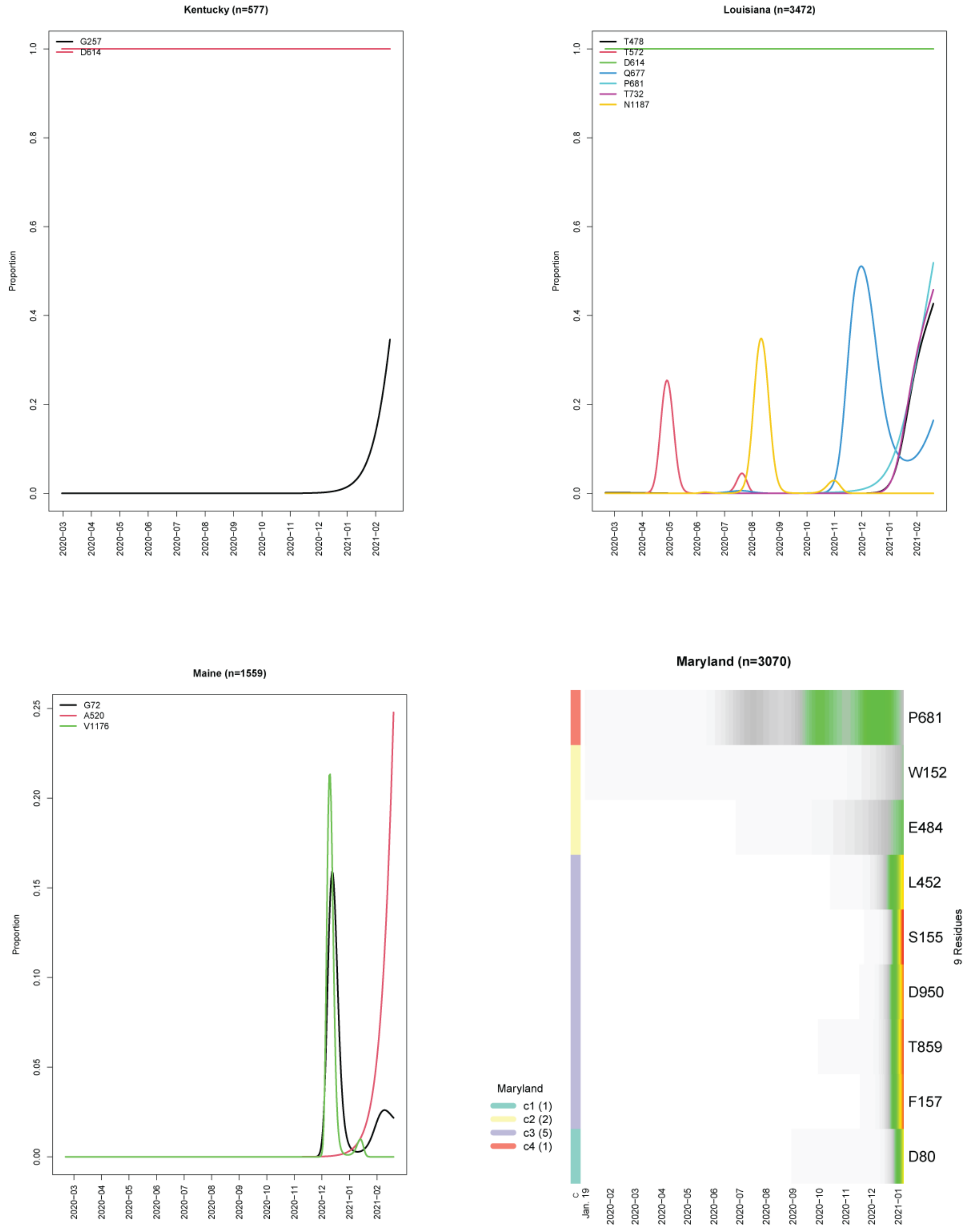

Figure S2 (continued).

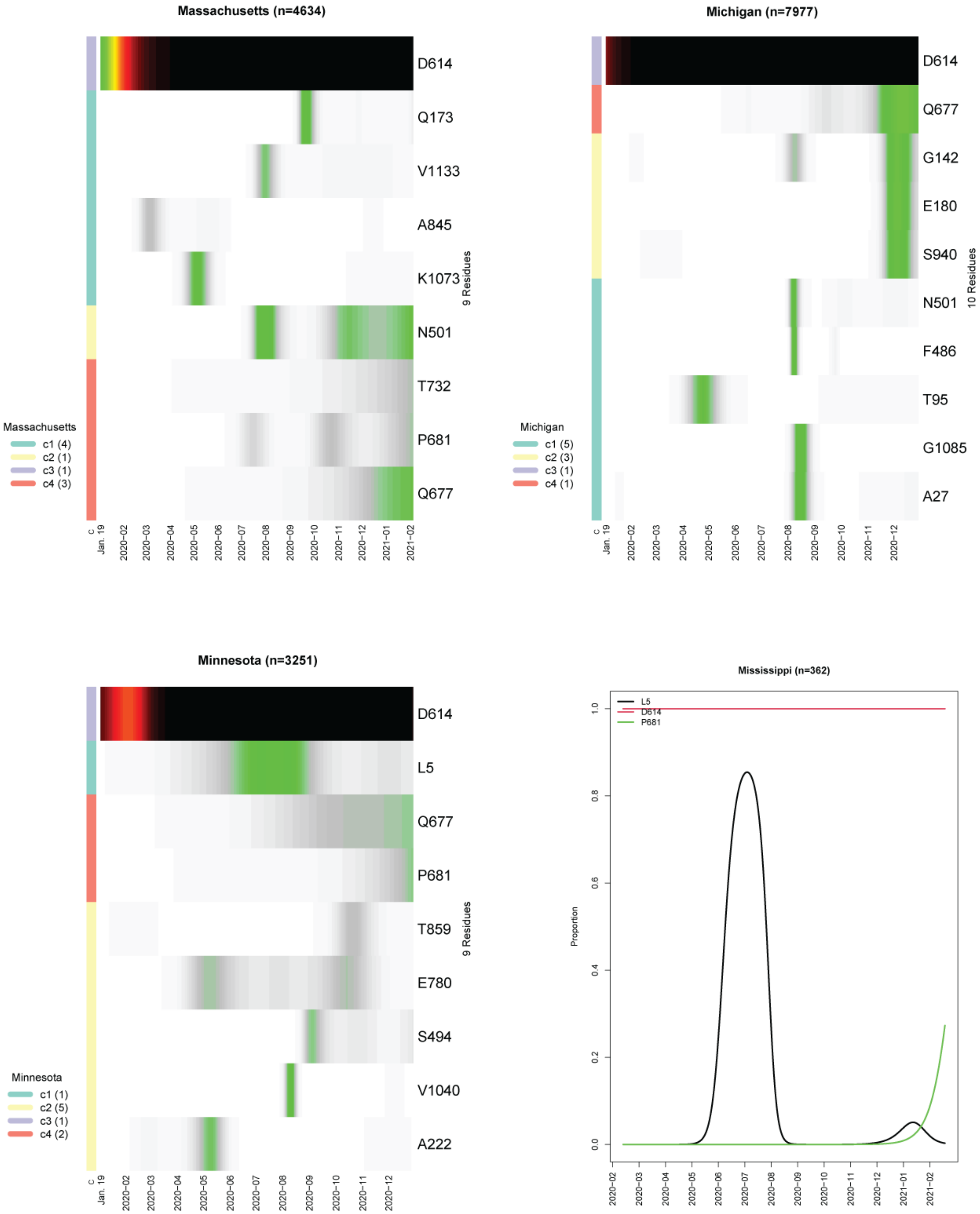

Figure S2 (continued).

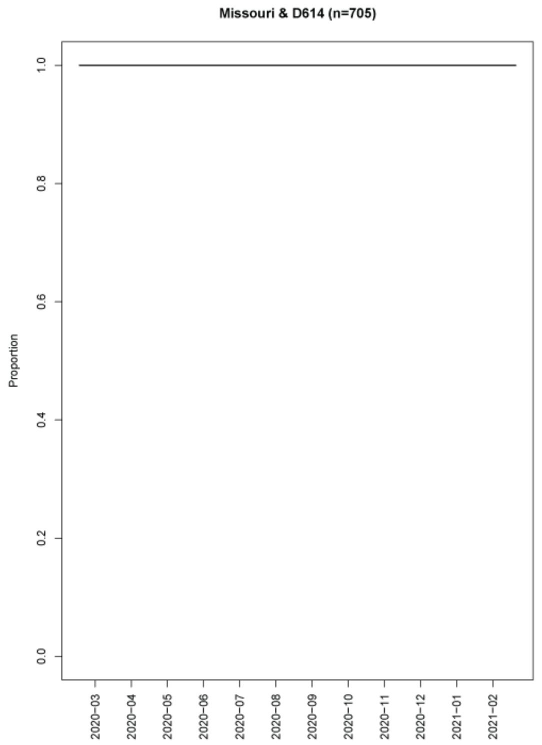

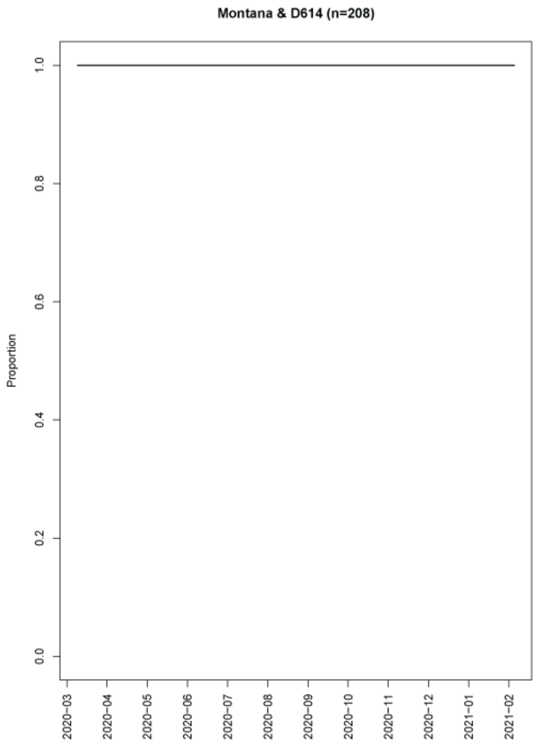

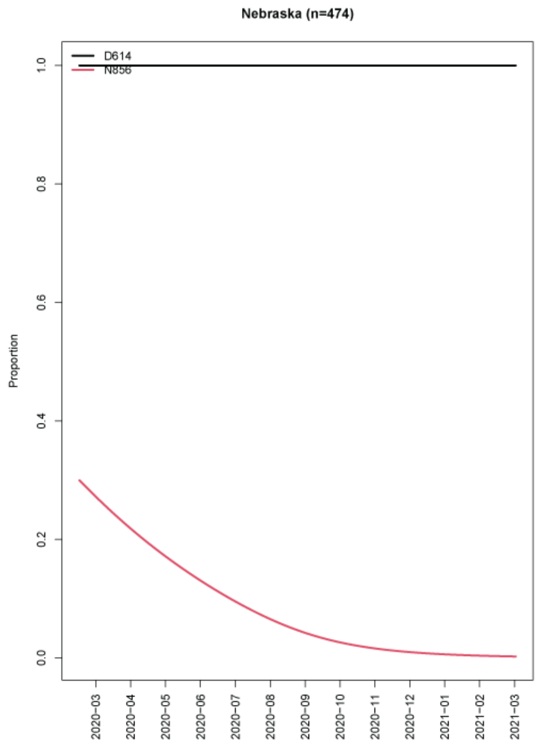

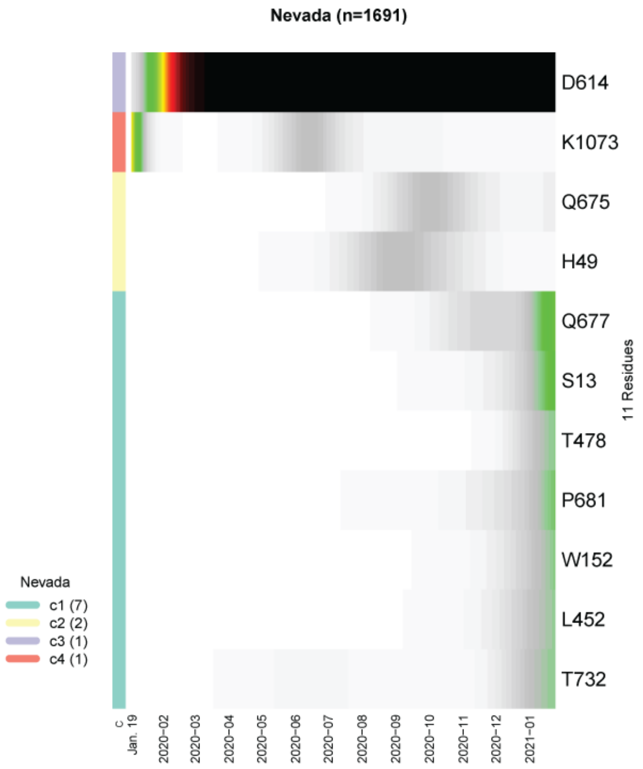

Figure S2 (continued).

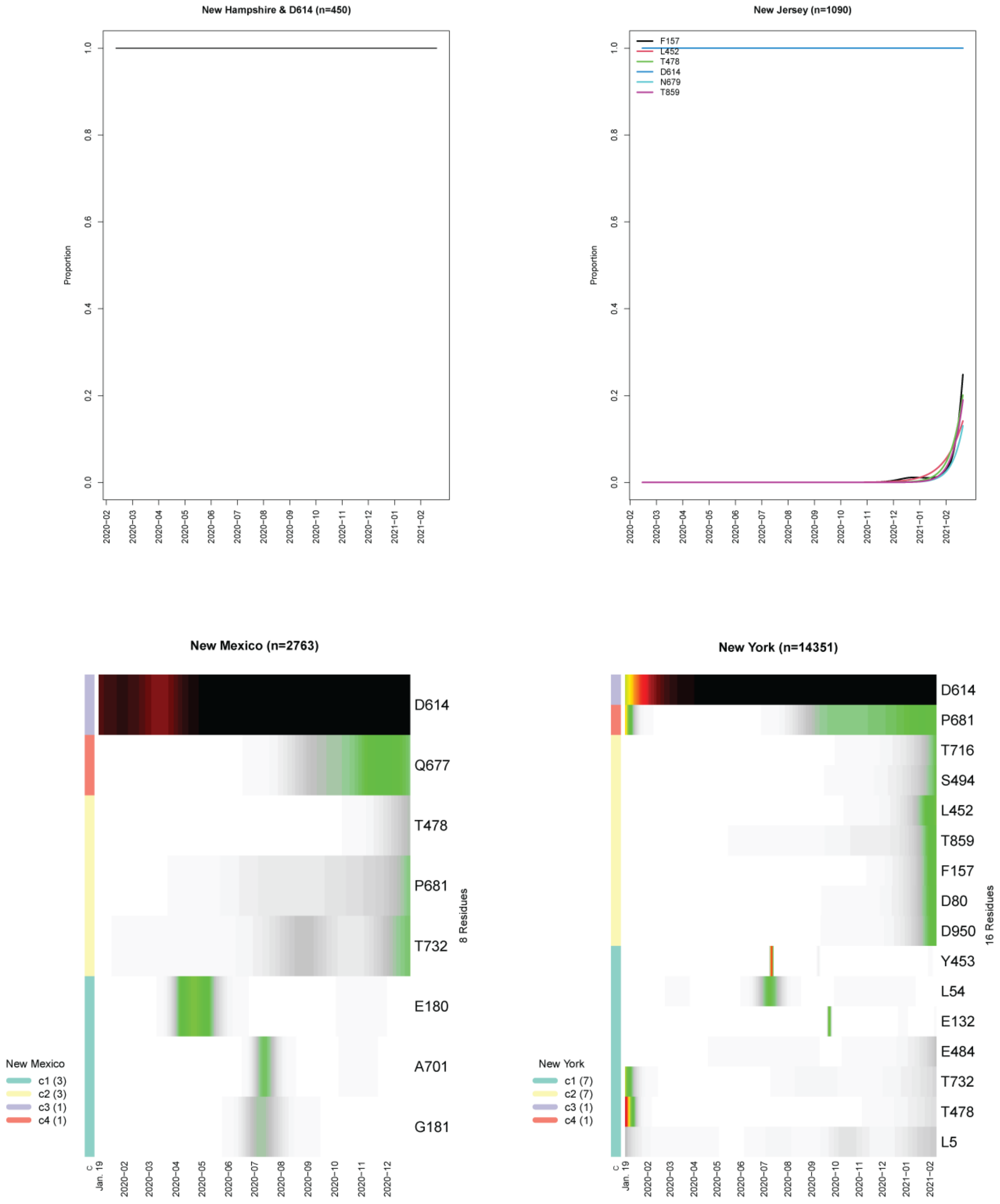

Figure S2 (continued).

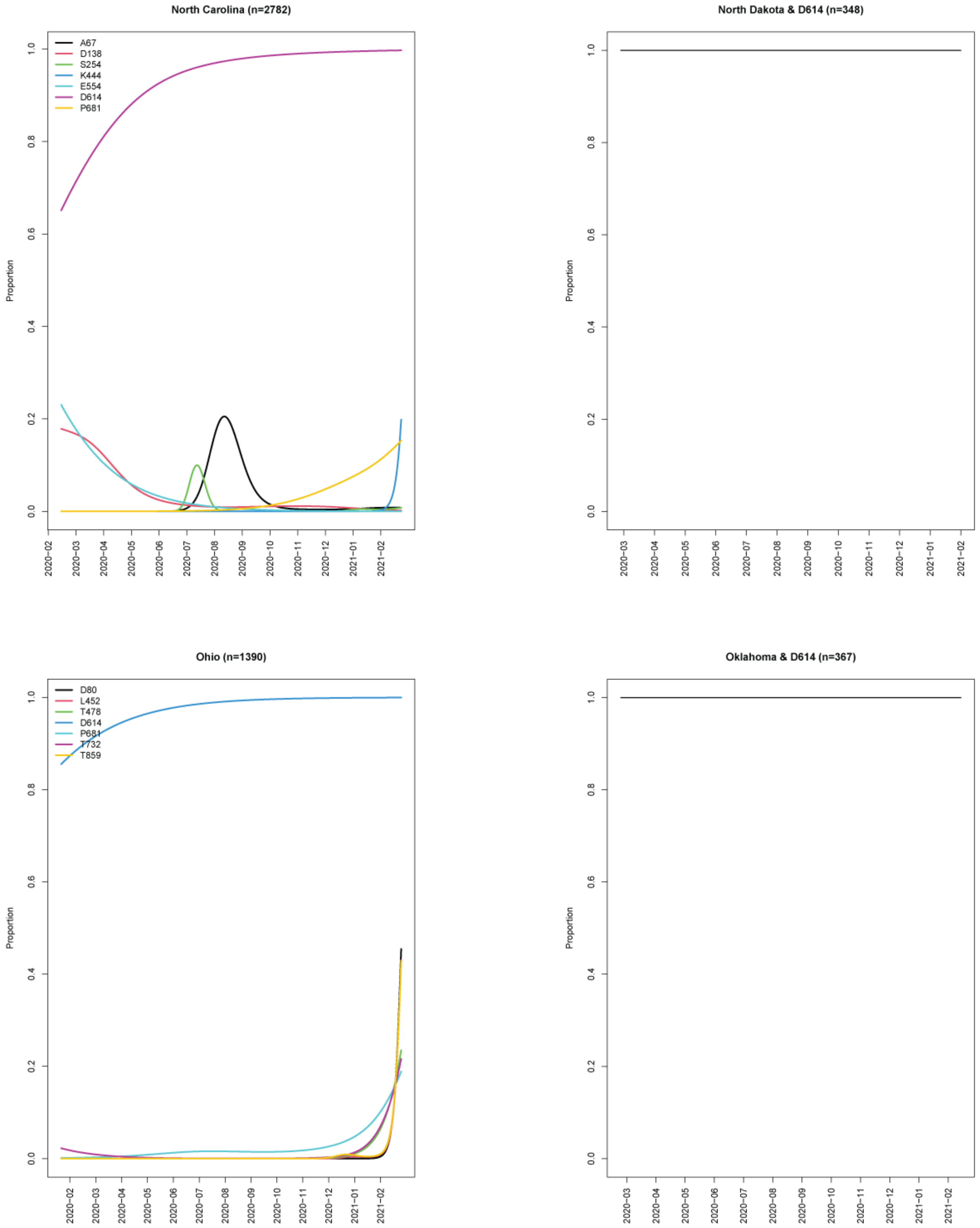

Figure S2 (continued).

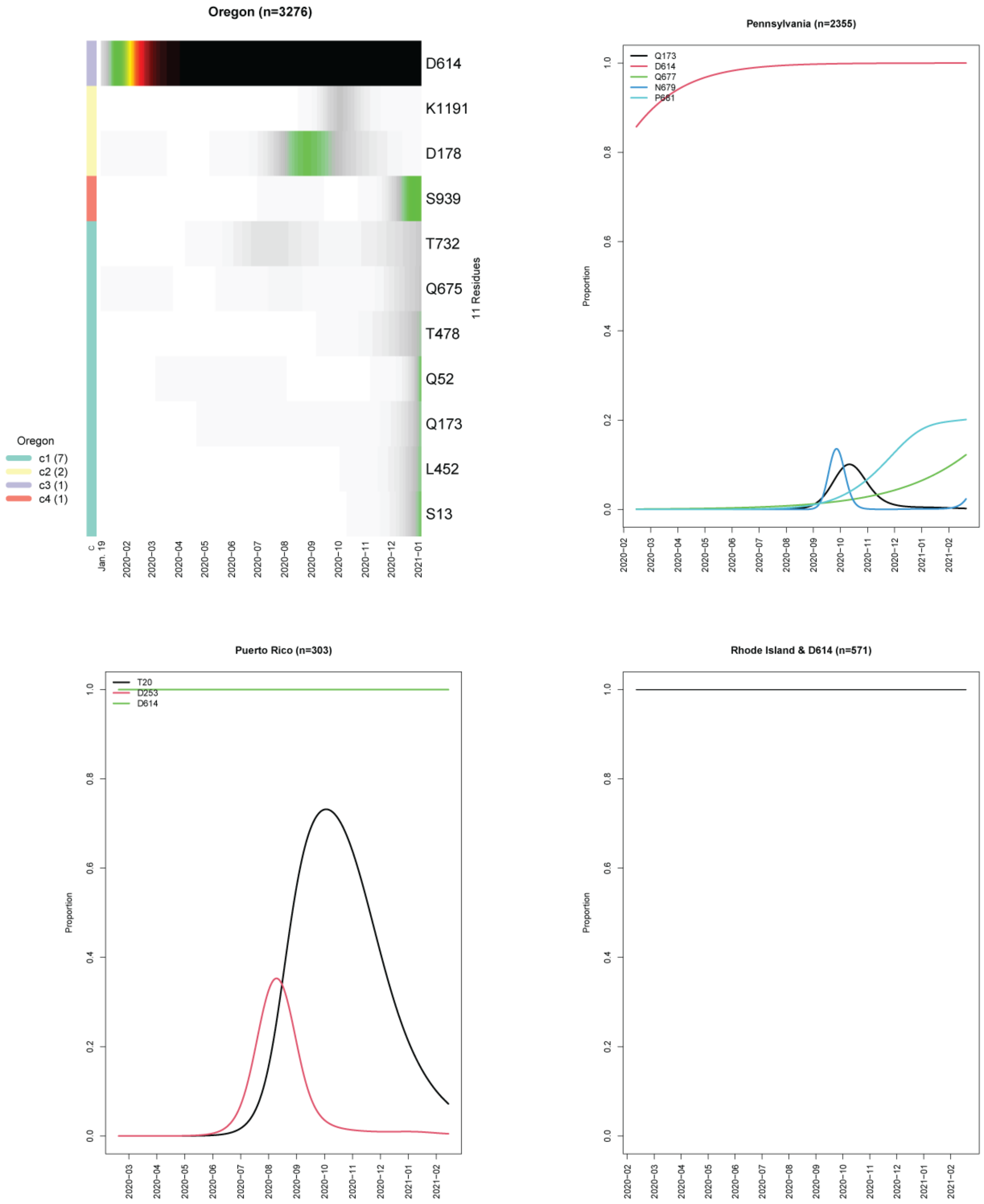

Figure S2 (continued).

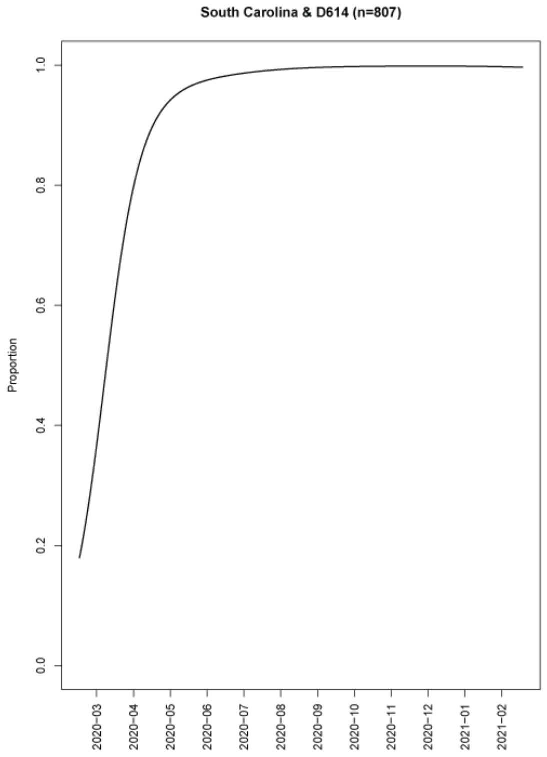

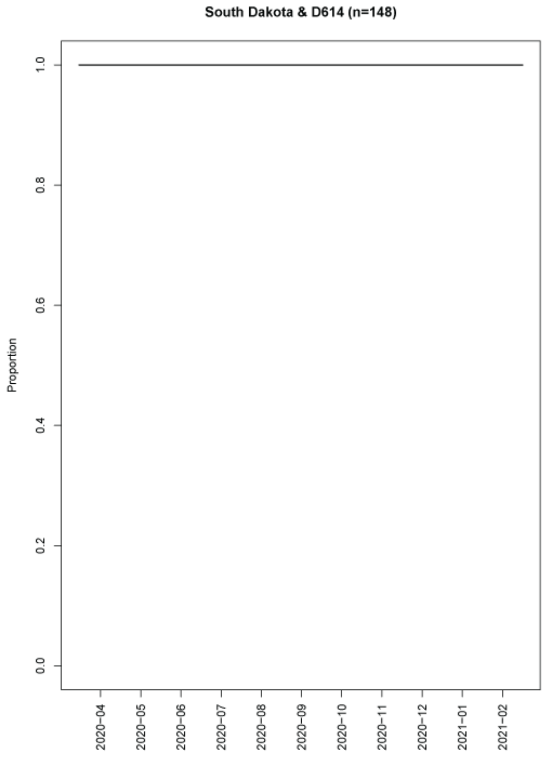

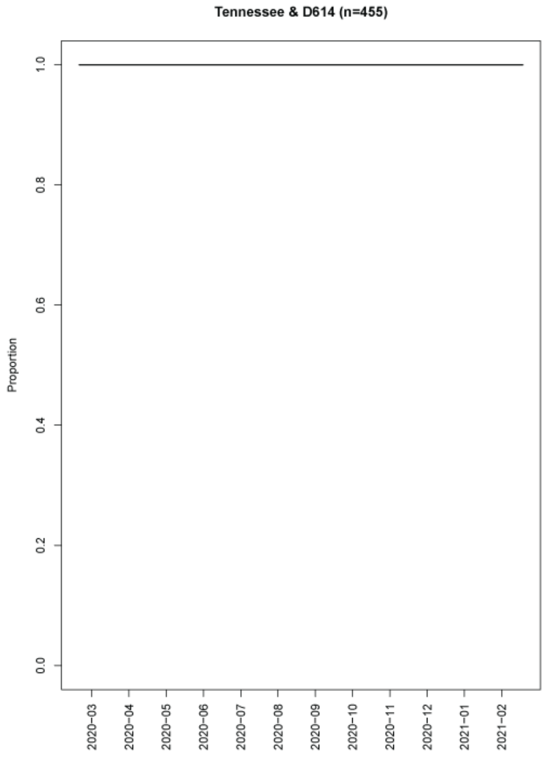

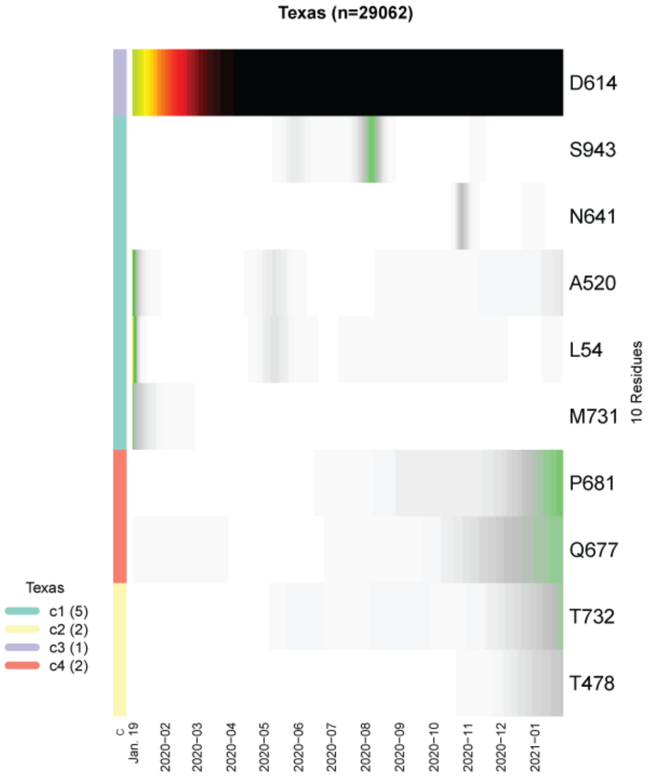

Figure S2 (continued).

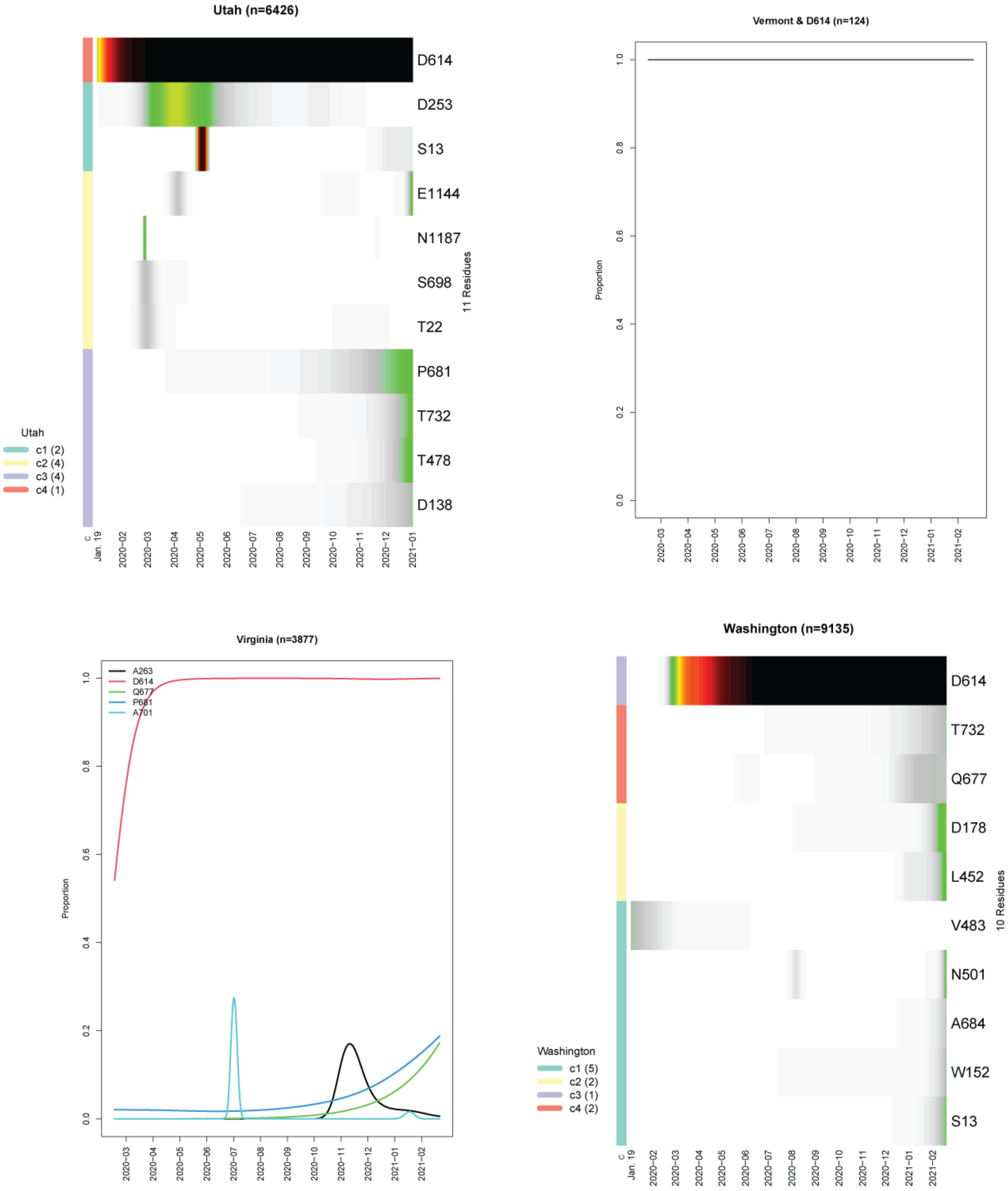

Figure S2 (continued).

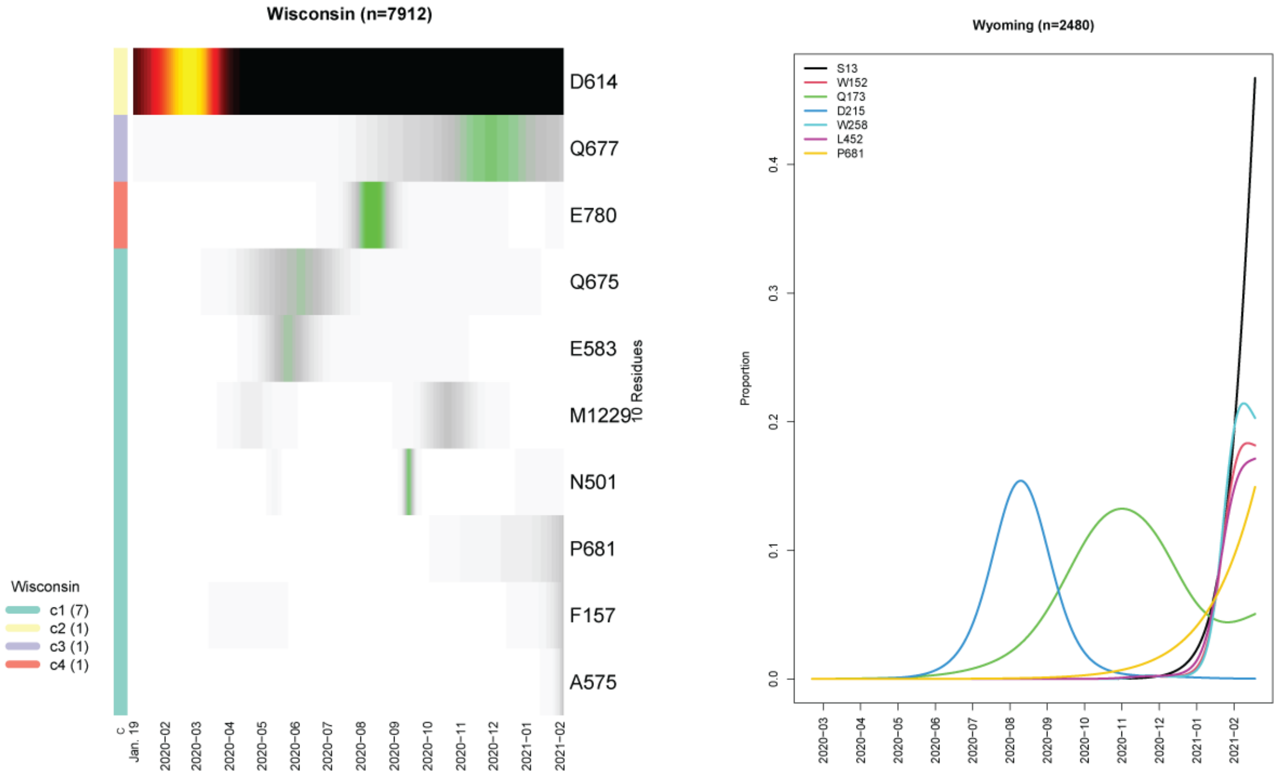

Figure S3. Locally averaged proportions over time for substitutions at 3 AA-subs in a VOI (Y144, F888, V1176) and at 3 AA-subs in a VOC (H69, Y144, K417) that were not detected by the SLS method in states/ territories where at least three sequences had a substitution at the designated AA position.

AA-sub, amino acid that has been shown to harbor a substitution in a US-circulating VOI or VOC. VOI, variant of interest; VOC, variant of concern. The "TP1" through "TP10" labels, along with the vertical color bar on the left side of the plot, refer to the 10 individual State-VRV clusters identified in the plot, based on their temporal patterns ("TP" = temporal pattern).

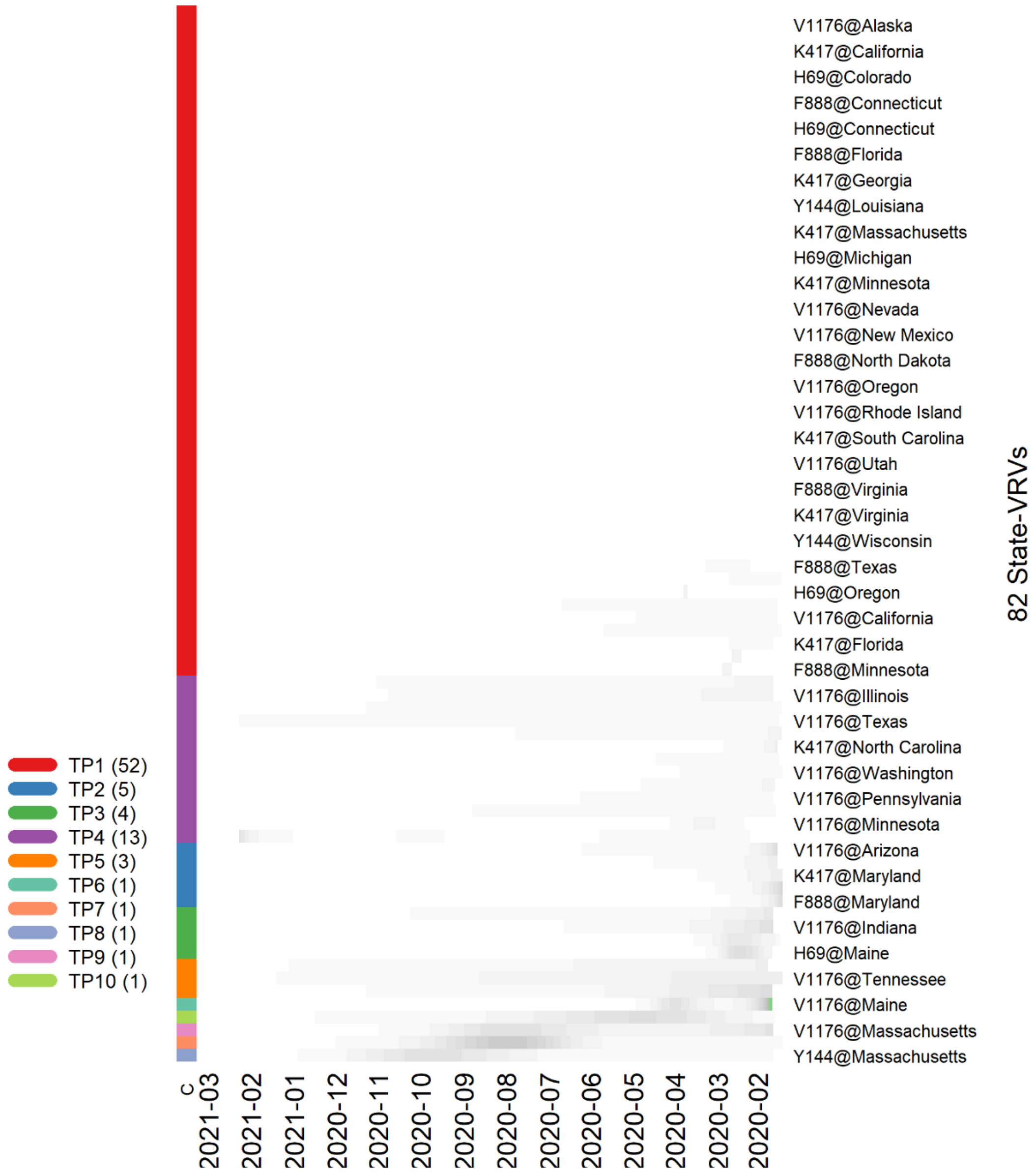

Figure S4. Presence of VRVs among all cases in each state/territory. A gray cell means the VRV was not identified in the given case; a black cell means that it was. Both cases and VRVs were clustered by two-way hierarchical cluster analysis. The “PG” (“patient group”) and “RG” (“residue group”) labels in the lower left corner of each plot correspond to the row (case) and column (VRV) clusters identified by two-way hierarchical cluster analysis, also identified by the vertical and horizontal color bars at the left and top, respectively, of each plot.

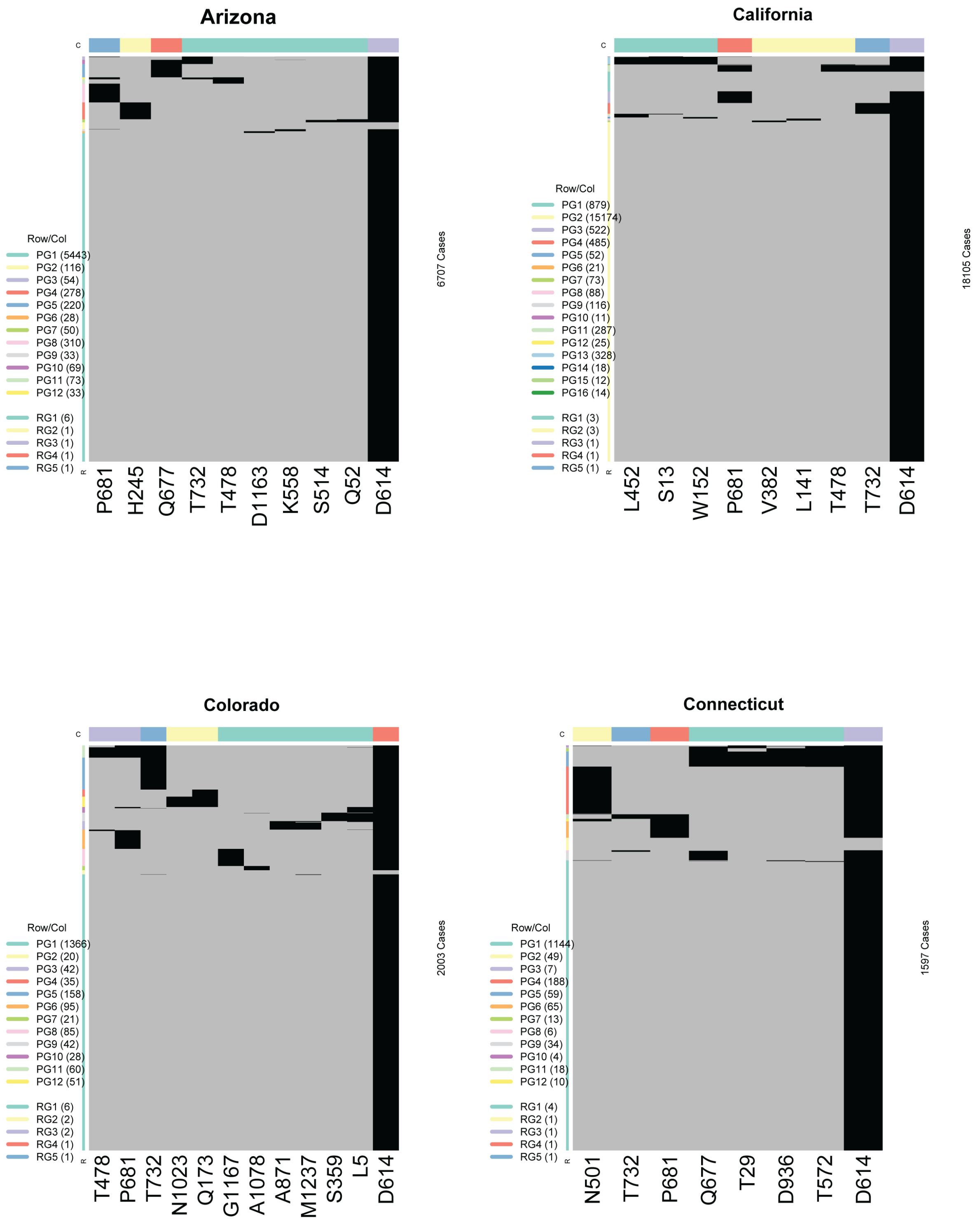

Figure S4 (continued).

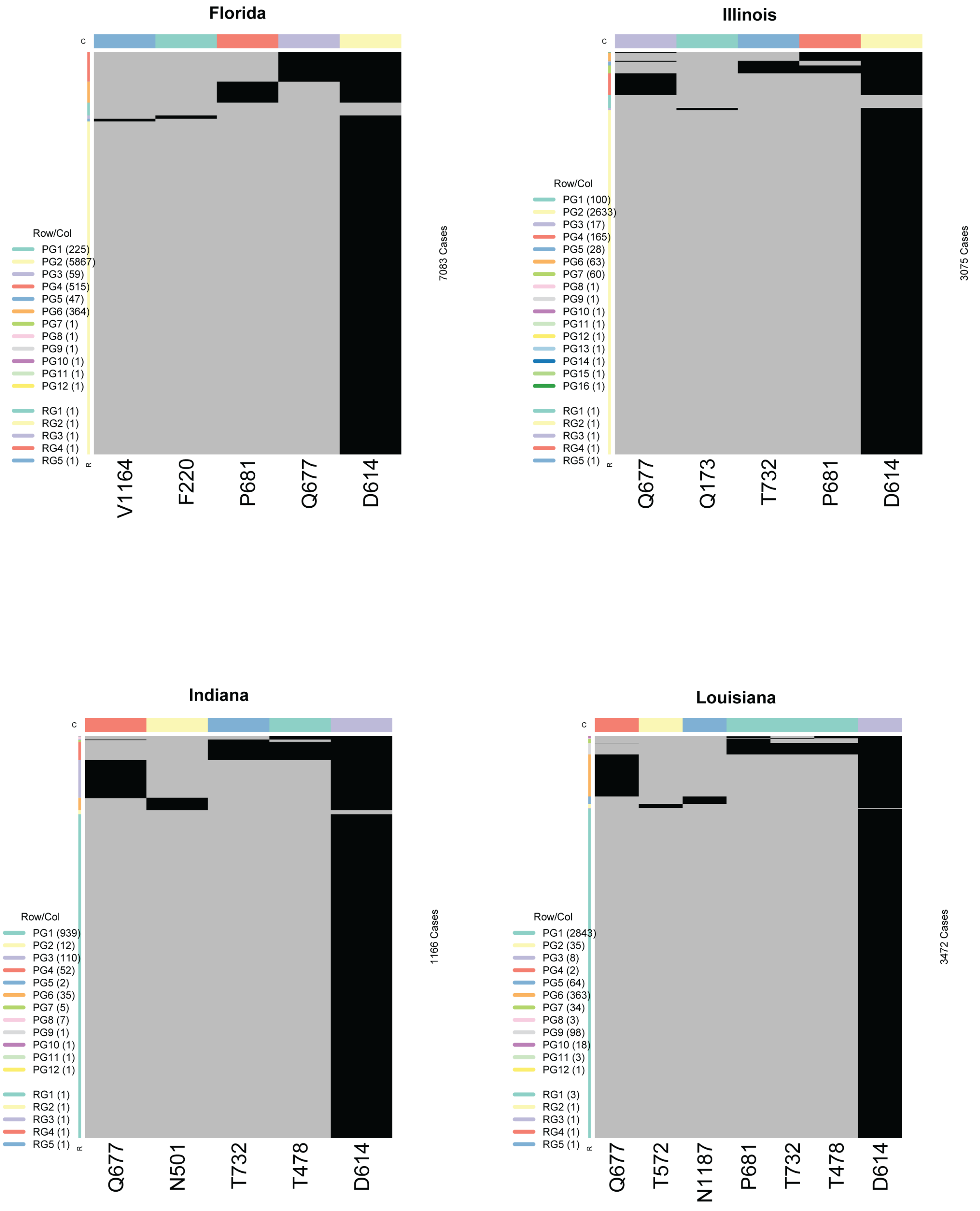

Figure S4 (continued).

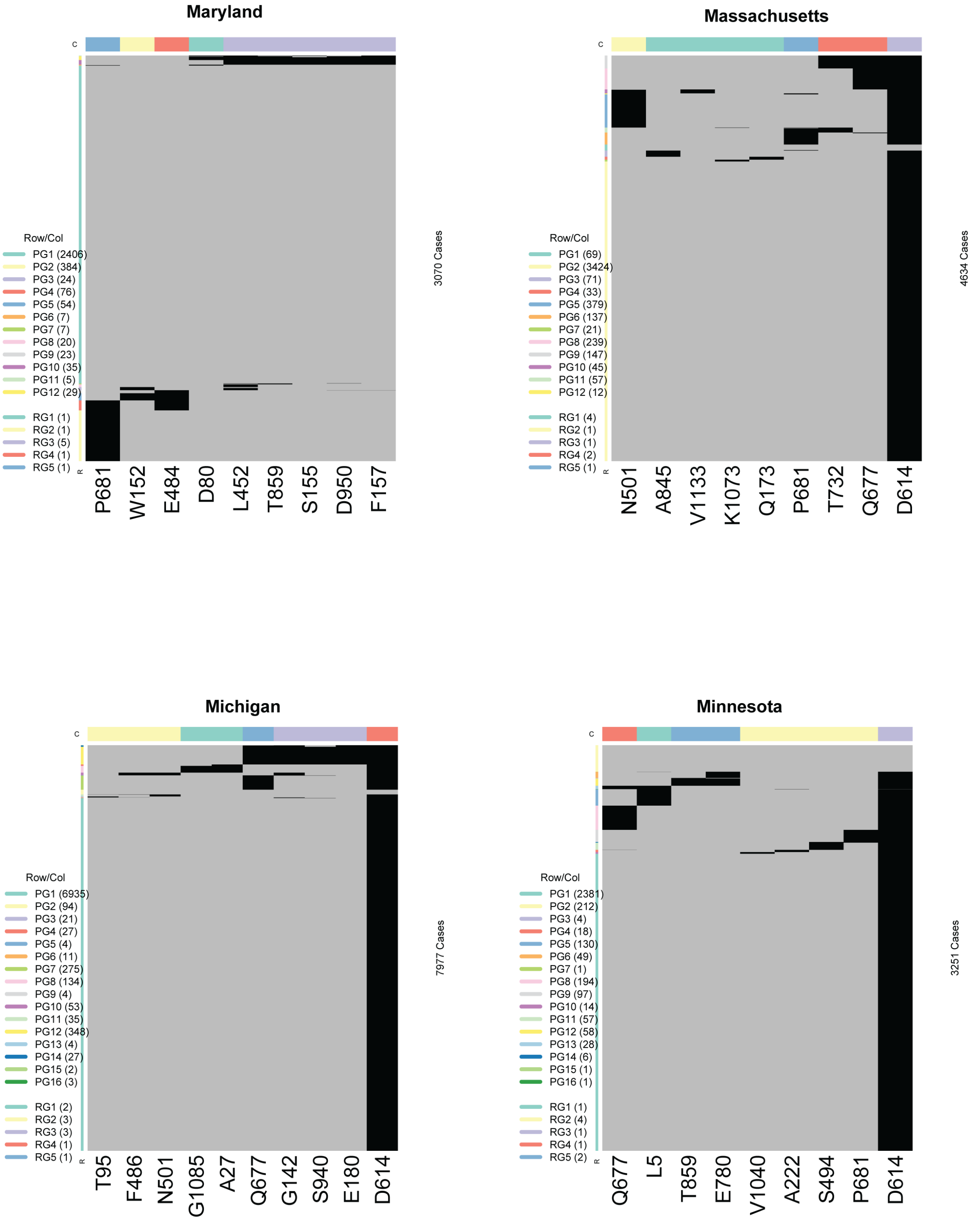

Figure S4 (continued).

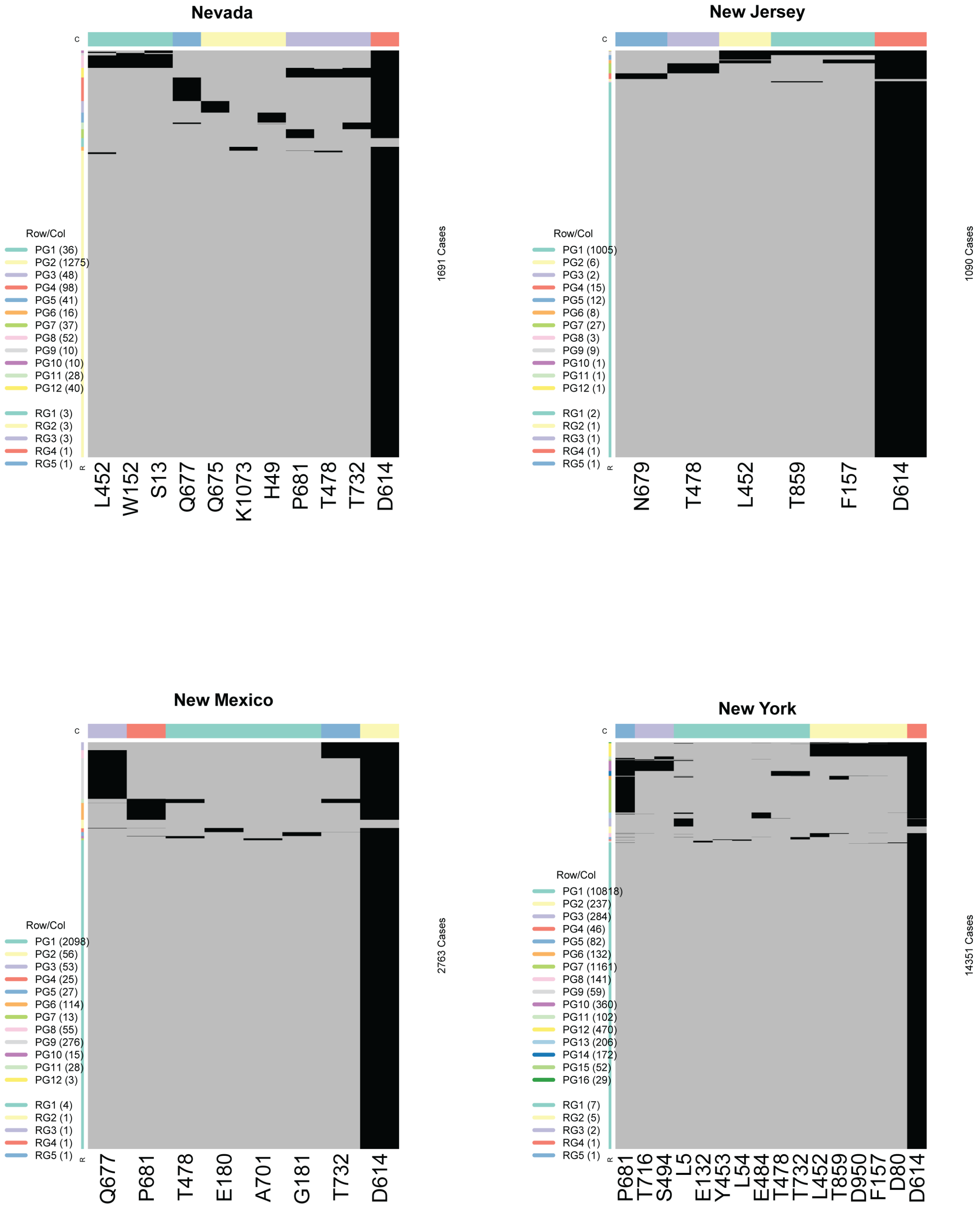

Figure S4 (continued).

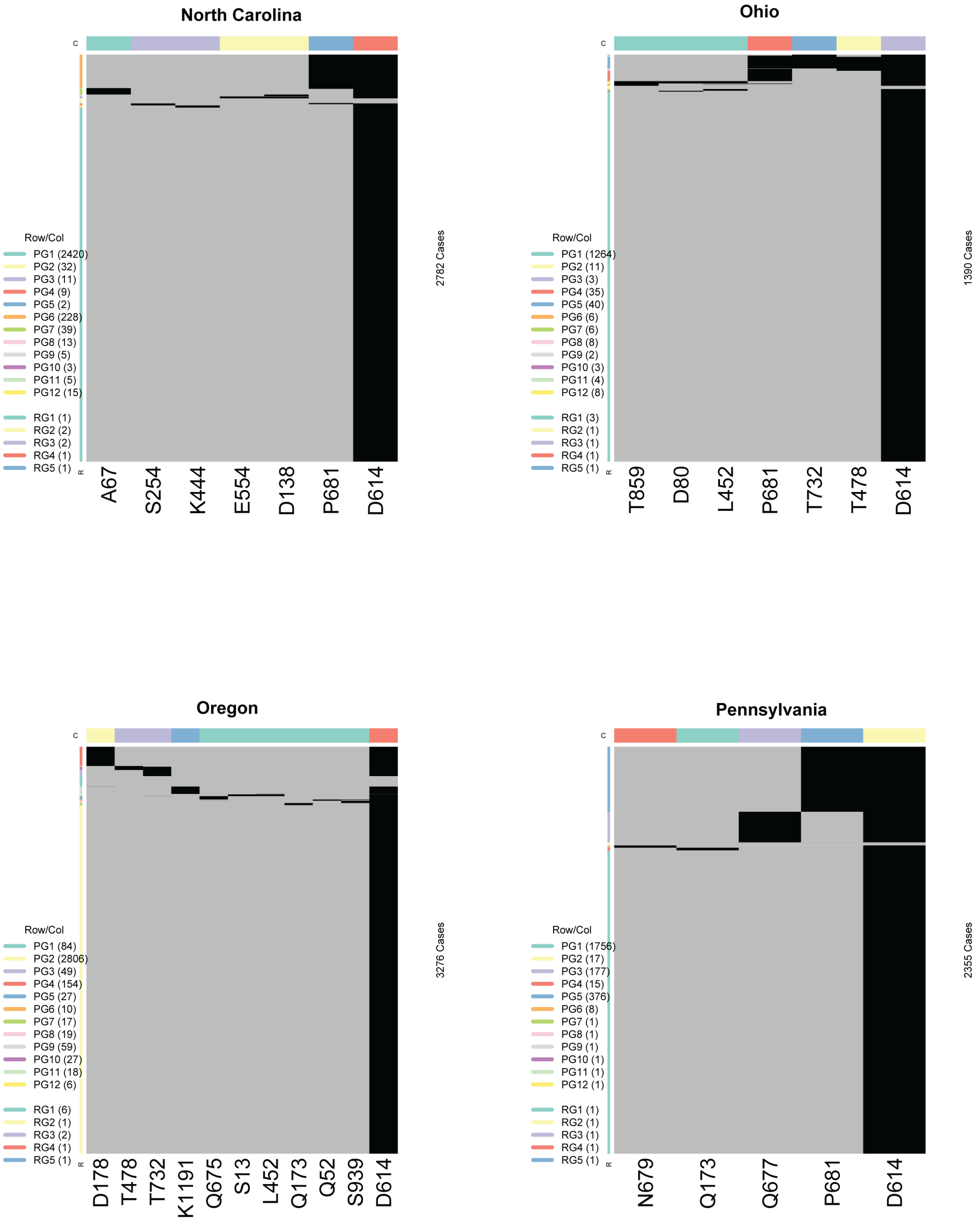

Figure S4 (continued).

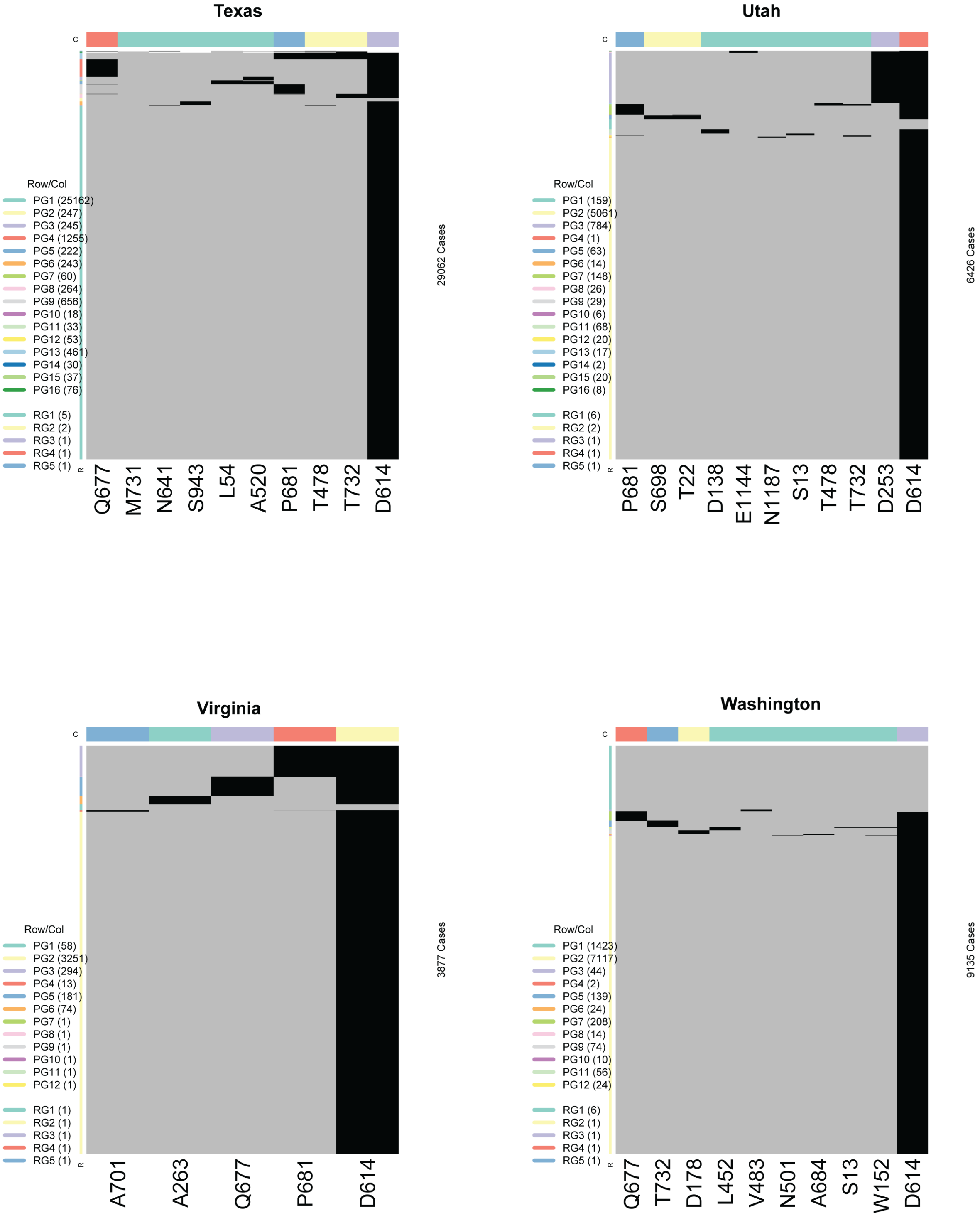

Figure S4 (continued).

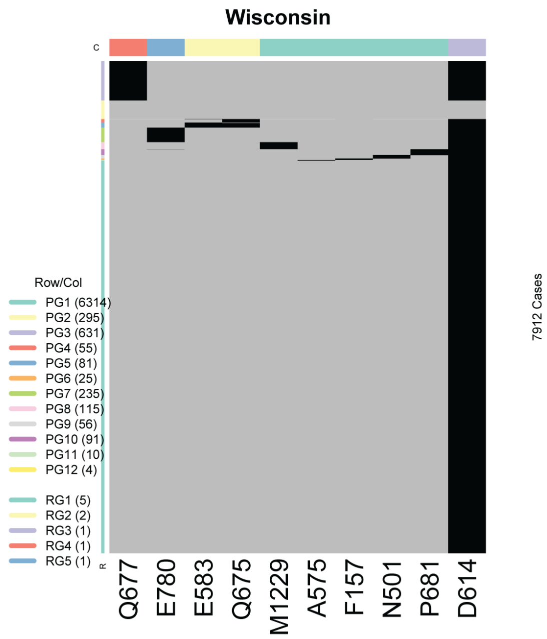

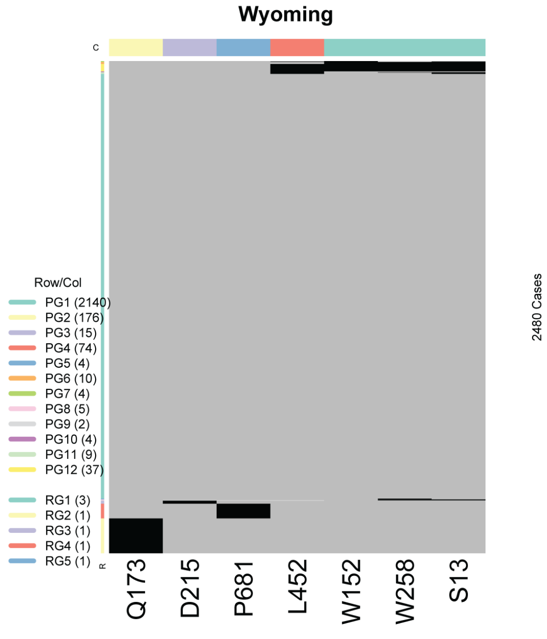

Table S1. Distribution of the 21,391 VOI/VOC sequences by specific variant and by state/territory.

State/Territory	B.1.1.7	B.1.351	B.1.427	B.1.429	B.1.525	B.1.526	P.1	P.2
**Alabama**	7		3	3		2		
**Alaska**	2			43			5	3
**Arizona**	44		119	396			3	7
**Arkansas**			4	3				
**California**	266	1	2432	5368	2	15	1	19
**Colorado**	195	7	79	189		6		2
**Connecticut**	146	2	18	57	5	40		3
**DC**	10		4	9	1	1		
**Delaware**	8	1	3	5	1	26		
**Florida**	708		205	257	2	19	12	74
**Georgia**	85	3	31	57	1	3		10
**Hawaii**	2		7	18				
**Idaho**	8	2	1	11				
**Illinois**	100	1	16	69	1	1	1	10
**Indiana**	18		23	20	1	1		24
**Iowa**	8		7	4				
**Kansas**	28		10	23				
**Kentucky**	8		2	11				6
**Louisiana**	38		4	32		1		
**Maine**	3	1	2	30		5		19
**Maryland**	220	20	7	55	4	27	1	7
**Massachusetts**	100	4	8	38		10		30
**Michigan**	706	1	48	83	5	7		14
**Minnesota**	249	1	20	77	3	1	2	4
**Mississippi**			6	4				1
**Missouri**			3	28				4
**Montana**	3		4	17		1		
**Nebraska**	6		4	15			2	
**Nevada**	12	2	198	643		1		4
**New Hampshire**	5		3	5				7
**New Jersey**	182		24	15	1	79	2	2
**New Mexico**	17		38	125				1
**New York**	769	3	103	174	16	2121		21
**North Carolina**	34	9	30	60		13		21
**North Dakota**	7		2	5	5			
**Ohio**	74		12	34	1	1	1	22
**Oklahoma**	8		1	4			1	
**Oregon**	17		34	135		1	1	1
**Pennsylvania**	51		23	74		10		11
**Puerto Rico**	8		1	6				3
**Rhode Island**	19			16	1	6		
**South Carolina**	1	6	4	25		2		3
**South Dakota**	5							
**Tennessee**	16		11	10				2
**Texas**	506	2	71	449	10	16	6	81
**Utah**	37		27	90		1		
**Vermont**	1			5		2		
**Virginia**	26	4	62	76	4	1		27
**Washington**	129	5	112	473	4	4	3	12
**Wisconsin**	35		21	60		1		11
**Wyoming**	10	1	34	65		14		
**USA-other**	7		1	4				

"USA-Other" includes sequences from South Dakota, Vermont, and the Virgin Islands, in addition to sequences for which state information was missing.

Table S2. Distribution of the 167,893 SARS-CoV-2 sequences by state/territory and by GISAID submission month, along with state/territory-specific distribution of the 21,391 VOI/VOC excluded sequences.

ID	State	2020-01	2020-02	2020-03	2020-04	2020-05	2020-06	2020-07	2020-08	2020-09	2020-10	2020-11	2020-12	2021-01	2021-02	2021-03	VOI/ VOC
1	Alabama			5	5	7		3	5	1	232	613	538	342	204	4	15
2	Alaska			25	10	1	42	60		1		82	51	81	219		53
3	Arizona	1		179	861	618	989	558	113	70	309	601	729	1130	546	3	569
4	Arkansas			13			1	65	1	13	6		17	174	99	4	7
5	California	6	21	1392	1228	1022	859	1345	665	718	564	1822	3115	3495	1776	77	8104
6	Colorado			66	148	53	14	26	51	114	61	289	71	614	447	49	478
7	Connecticut			272	207	40	7	9	29	31	22	55	121	360	409	35	271
8	DC			96	2	5	2	13			2	17	93	144	122		25
9	Delaware			105	14	31	23	6	22	33	50	35	91	264	77	21	44
10	Florida		3	351	630	198	308	580	336	240	210	298	406	1138	2364	21	1277
11	Georgia		2	94	26	2	18	30	16	20	85	47	428	720	1103	16	190
12	Hawaii			30	28	5	42	60	171	111	107	105	210	89	23		27
13	Idaho			161	33	2	4	9	8	1	3	15	15	49	35	3	22
14	Illinois	2		181	286	116	162	279	25	72	172	144	518	677	440	1	199
15	Indiana			264	9			2				7	96	288	501		87
16	Iowa			20	18	6	19	8	10	20	10	30	19	91	38		19
17	Kansas			21	19	10	9	127	26	9	8	43	103	310	169	10	61
18	Kentucky			27	20	5	16	1	3	12	18	41	51	237	146		27
19	Louisiana			108	645	201	225	320	307	61	51	169	505	546	324	10	75
20	Maine			106		1	9		10	100	18	89	276	614	328	8	60
21	Maryland			118	57	105	31	30	1	38	163	216	439	1074	725	73	341
22	Massachusetts	1		312	725	376	28	39	48	164	101	196	387	1064	1170	23	190
23	Michigan			1570	755	197	39	95	135	264	741	1605	231	1019	1228	98	864
24	Minnesota			579	325	208	111	49	102	88	171	190	304	648	474	2	357
25	Mississippi			21	5			4	13	14	2	26	26	136	111	4	11
26	Missouri			28			25	1	12	10	11	21	107	309	179	2	35
27	Montana			1	1	3	20	37	4		6	4	38	64	30		25
28	Nebraska			7	1	97		5	8	11	7	22	34	124	148	10	27
29	Nevada		1	80	66	53	3		57	21	185	17	88	676	417	27	860
30	New Hampshire		1	5	4	5	2	1				9	46	126	246	5	20
31	New Jersey			80	24	1	4	2	4	5	15	11	192	354	393	6	305
32	New Mexico			63	154	282	64	46	162	145	109	162	282	913	357	24	181
33	New York	1	2	2179	1074	257	65	105	176	313	613	523	2144	3700	2871	328	3207
34	North Carolina			18	45	11	33	54	133	23	10	37	128	980	1289	21	167
35	North Dakota			41	1	3	4	1	5	2	30	52	20	183	6		19
36	Ohio		1	72	63	5	4	33	9	3	4	63	144	482	491	16	145
37	Oklahoma			9	1	3		7	11	8	15	27	22	188	76		14
38	Oregon		1	67	148	138	332	342	226	143	288	320	533	624	113	1	189
39	Pennsylvania			101	84	7	15	21	8	36	43	57	292	408	1275	8	169
40	Puerto Rico			20	16	1	8	25	54	25	35	11	24	67	17		18
41	Rhode Island		1	5	8	3	2	6	1		2	38	95	180	228	2	42
42	South Carolina			29	11	32	99	64	2	6	1	7	27	325	203	1	41
43	South Dakota				4	7	3	10	8	13	34	11	36	12	10		5
44	Tennessee			10	1				3		8	70	34	189	138	2	39
45	Texas		2	504	725	789	5448	3214	1494	794	1931	2895	943	5702	4101	520	1141
46	Utah			397	948	1428	469	369	626	43	159	177	980	696	133	1	155
47	Vermont			4	1	1	3					4	6	44	59	2	8
48	Virginia			153	390	217	116	179	186	135	186	244	402	838	825	6	200
49	Washington	4	58	1625	855	892	1353	165	575	186	286	476	596	1161	886	17	742
50	Wisconsin	1		375	399	194	387	389	323	1126	1056	721	969	1180	719	74	128
51	Wyoming			97	16	4	12	60	53	2	60	92	900	921	262	1	124
52	USA		25	77	109	4	9	86	1	1	1		98	17	3		12

“USA includes sequences from South Dakota, Vermont, and the Virgin Islands, in addition to sequences for which state information was missing.

Table S3. The 10 identified geo-VRV clusters (TP1 through TP10), based on temporal profiles.

ID	CLS	#	State	VRV	p	q	Pmax
1	TP1	1	Alabama	E484	1.41E-04	2.61E-03	1.26E-01
2	TP1	2	Alaska	V1228	3.02E-04	2.32E-03	1.70E-01
3	TP1	3	Arizona	Q52	1.00E-10	1.10E-09	1.32E-01
4	TP1	4	Arizona	H245	1.00E-10	1.10E-09	2.49E-01
5	TP1	5	Arizona	S514	1.00E-10	1.10E-09	4.47E-01
6	TP1	6	Arizona	K558	1.00E-10	1.10E-09	5.59E-01
7	TP1	7	Arizona	D1163	1.08E-06	9.96E-06	1.15E-01
8	TP1	8	California	L141	1.00E-10	1.14E-09	1.01E-01
9	TP1	9	California	V382	1.00E-10	1.14E-09	1.50E-01
10	TP1	10	Colorado	L5	4.80E-04	3.32E-03	2.92E-01
11	TP1	11	Colorado	Q173	5.14E-05	5.14E-04	2.77E-01
12	TP1	12	Colorado	S359	1.00E-10	1.13E-09	3.58E-01
13	TP1	13	Colorado	N1023	3.10E-04	2.32E-03	1.02E-01
14	TP1	14	Colorado	A1078	1.00E-10	1.13E-09	2.46E-01
15	TP1	15	Connecticut	T732	5.26E-05	4.91E-04	1.24E-01
16	TP1	16	Florida	F220	1.00E-10	2.91E-09	1.75E-01
17	TP1	17	Florida	V1164	3.18E-06	6.73E-05	3.49E-01
18	TP1	18	Georgia	A263	1.02E-05	3.15E-04	3.62E-01
19	TP1	19	Illinois	Q173	3.41E-04	5.49E-03	1.12E-01
20	TP1	20	Louisiana	T572	3.81E-06	4.90E-05	2.54E-01
21	TP1	21	Louisiana	N1187	1.00E-10	1.50E-09	3.49E-01
22	TP1	22	Maine	G72	3.34E-05	8.12E-04	1.59E-01
23	TP1	23	Maine	E484	1.02E-06	3.71E-05	1.32E-01
24	TP1	24	Massachusetts	Q173	1.00E-10	2.00E-09	2.53E-01
25	TP1	25	Massachusetts	A845	1.00E-10	2.00E-09	1.20E-01
26	TP1	26	Massachusetts	K1073	1.00E-10	2.00E-09	2.16E-01
27	TP1	27	Massachusetts	V1133	8.17E-04	7.35E-03	1.66E-01
28	TP1	28	Michigan	A27	1.00E-10	1.02E-09	2.58E-01
29	TP1	29	Michigan	T95	1.00E-10	1.02E-09	2.01E-01
30	TP1	30	Michigan	F486	3.18E-07	3.05E-06	2.65E-01
31	TP1	31	Michigan	N501	1.00E-10	1.02E-09	2.24E-01
32	TP1	32	Michigan	G1085	1.00E-10	1.02E-09	2.71E-01
33	TP1	33	Minnesota	L5	1.00E-10	1.67E-09	2.51E-01
34	TP1	34	Minnesota	A222	1.00E-10	1.67E-09	1.76E-01
35	TP1	35	Minnesota	S494	1.32E-05	1.55E-04	1.56E-01
36	TP1	36	Minnesota	E780	1.00E-10	1.67E-09	1.39E-01
37	TP1	37	Minnesota	T859	1.00E-10	1.67E-09	1.20E-01
38	TP1	38	Minnesota	V1040	9.48E-06	1.23E-04	2.47E-01
39	TP1	39	Nebraska	N856	1.67E-05	2.01E-04	3.00E-01
40	TP1	40	Nevada	H49	1.00E-10	4.90E-09	1.20E-01
41	TP1	41	Nevada	Q675	4.07E-04	3.99E-03	1.15E-01
42	TP1	42	Nevada	K1073	9.42E-05	1.20E-03	4.90E-01
43	TP1	43	New Jersey	N679	3.17E-04	3.85E-03	1.29E-01
44	TP1	44	New Mexico	E180	1.00E-10	5.85E-09	3.55E-01
45	TP1	45	New Mexico	G181	3.93E-06	6.79E-05	1.38E-01
46	TP1	46	New Mexico	A701	9.61E-04	9.37E-03	1.75E-01
47	TP1	47	New York	L54	1.00E-10	7.97E-10	1.93E-01
48	TP1	48	New York	E132	1.00E-10	7.97E-10	3.51E-01
49	TP1	49	New York	Y453	1.00E-10	7.97E-10	7.52E-01
50	TP1	50	New York	T478	1.00E-10	7.97E-10	7.79E-01
51	TP1	51	New York	E484	1.00E-10	7.97E-10	1.07E-01
52	TP1	52	New York	T732	1.00E-10	7.97E-10	4.38E-01
53	TP1	53	North Carolina	A67	1.00E-10	3.25E-09	2.05E-01
54	TP1	54	North Carolina	D138	1.00E-10	3.25E-09	1.78E-01
55	TP1	55	North Carolina	K444	7.75E-06	2.01E-04	1.81E-01
56	TP1	56	North Carolina	E554	1.00E-10	3.25E-09	2.31E-01
57	TP1	57	Oregon	D178	1.00E-10	2.54E-09	1.82E-01

58	TP1	58	Oregon	K1191	1.18E-05	1.50E-04	1.02E-01
59	TP1	59	Pennsylvania	Q173	1.01E-05	5.84E-04	1.01E-01
60	TP1	60	Pennsylvania	N679	2.73E-05	7.91E-04	1.36E-01
61	TP1	61	Puerto Rico	D253	3.82E-04	2.42E-03	3.53E-01
62	TP1	62	Texas	L54	1.00E-10	1.48E-09	4.61E-01
63	TP1	63	Texas	A520	1.00E-10	1.48E-09	2.37E-01
64	TP1	64	Texas	N641	1.00E-10	1.48E-09	1.12E-01
65	TP1	65	Texas	M731	1.10E-04	1.24E-03	1.67E-01
66	TP1	66	Texas	S943	1.00E-10	1.48E-09	1.63E-01
67	TP1	67	Utah	S13	1.00E-10	9.75E-10	9.43E-01
68	TP1	68	Utah	T22	1.00E-10	9.75E-10	1.09E-01
69	TP1	69	Utah	D253	1.00E-10	9.75E-10	4.58E-01
70	TP1	70	Utah	S698	1.00E-10	9.75E-10	1.19E-01
71	TP1	71	Utah	N1187	8.01E-04	5.00E-03	3.45E-01
72	TP1	72	Virginia	A263	1.00E-10	2.90E-09	1.69E-01
73	TP1	73	Virginia	A701	4.00E-05	4.83E-04	2.75E-01
74	TP1	74	Washington	S13	1.00E-10	1.81E-09	1.77E-01
75	TP1	75	Washington	W152	1.00E-10	1.81E-09	1.33E-01
76	TP1	76	Washington	V483	2.32E-06	3.30E-05	1.36E-01
77	TP1	77	Washington	N501	4.61E-04	3.61E-03	2.22E-01
78	TP1	78	Washington	A684	1.00E-10	1.81E-09	1.15E-01
79	TP1	79	Wisconsin	F157	1.00E-10	1.30E-09	1.01E-01
80	TP1	80	Wisconsin	N501	1.00E-10	1.30E-09	1.66E-01
81	TP1	81	Wisconsin	A575	1.77E-06	1.97E-05	1.06E-01
82	TP1	82	Wisconsin	E583	1.00E-10	1.30E-09	1.33E-01
83	TP1	83	Wisconsin	Q675	1.00E-10	1.30E-09	1.31E-01
84	TP1	84	Wisconsin	P681	1.00E-10	1.30E-09	1.04E-01
85	TP1	85	Wisconsin	E780	1.00E-10	1.30E-09	2.16E-01
86	TP1	86	Wisconsin	M1229	1.00E-10	1.30E-09	1.03E-01
87	TP1	87	Wyoming	Q173	9.60E-06	1.78E-04	1.32E-01
88	TP1	88	Wyoming	D215	1.00E-10	2.17E-09	1.54E-01
89	TP10	1	Puerto Rico	T20	1.00E-10	9.50E-10	7.32E-01
90	TP2	1	Alabama	D614	1.00E-10	3.70E-09	1.00E+00
91	TP2	2	Alaska	D614	1.00E-10	1.15E-09	1.00E+00
92	TP2	3	Arizona	D614	1.00E-10	1.10E-09	9.98E-01
93	TP2	4	Arkansas	D614	1.00E-10	1.70E-09	1.00E+00
94	TP2	5	California	D614	1.00E-10	1.14E-09	1.00E+00
95	TP2	6	Colorado	D614	1.00E-10	1.13E-09	1.00E+00
96	TP2	7	Connecticut	D614	1.11E-06	1.56E-05	1.00E+00
97	TP2	8	Delaware	D614	1.00E-10	5.30E-09	9.96E-01
98	TP2	9	Florida	D614	1.00E-10	2.91E-09	1.00E+00
99	TP2	10	Georgia	D614	1.00E-10	6.15E-09	1.00E+00
100	TP2	11	Hawaii	D614	1.07E-07	1.07E-06	9.99E-01
101	TP2	12	Illinois	D614	1.00E-10	3.23E-09	1.00E+00
102	TP2	13	Indiana	D614	4.97E-04	7.46E-03	9.99E-01
103	TP2	14	Iowa	D614	1.00E-10	1.20E-09	1.00E+00
104	TP2	15	Kansas	D614	1.00E-10	4.10E-09	1.00E+00
105	TP2	16	Kentucky	D614	1.00E-10	3.40E-09	1.00E+00
106	TP2	17	Louisiana	D614	1.00E-10	1.50E-09	1.00E+00
107	TP2	18	Maine	D614	4.55E-04	8.31E-03	1.00E+00
108	TP2	19	Massachusetts	D614	1.00E-10	2.00E-09	1.00E+00
109	TP2	20	Michigan	D614	1.00E-10	1.02E-09	1.00E+00
110	TP2	21	Minnesota	D614	1.00E-10	1.67E-09	1.00E+00
111	TP2	22	Mississippi	D614	1.00E-10	1.80E-09	1.00E+00
112	TP2	23	Missouri	D614	1.00E-10	3.50E-09	1.00E+00
113	TP2	24	Montana	D614	1.00E-10	1.00E-09	1.00E+00
114	TP2	25	Nebraska	D614	1.00E-10	2.40E-09	1.00E+00
115	TP2	26	Nevada	D614	8.86E-07	2.48E-05	1.00E+00
116	TP2	27	New Hampshire	D614	1.00E-10	4.50E-09	1.00E+00

117	TP2	28	New Jersey	D614	1.00E-10	7.30E-09	1.00E+00
118	TP2	29	New Mexico	D614	1.77E-06	5.19E-05	1.00E+00
119	TP2	30	New York	D614	1.00E-10	7.97E-10	1.00E+00
120	TP2	31	North Carolina	D614	1.00E-10	3.25E-09	9.97E-01
121	TP2	32	North Dakota	D614	1.00E-10	1.60E-09	1.00E+00
122	TP2	33	Ohio	D614	1.05E-05	1.26E-04	1.00E+00
123	TP2	34	Oklahoma	D614	1.00E-10	2.30E-09	1.00E+00
124	TP2	35	Oregon	D614	1.00E-10	2.54E-09	1.00E+00
125	TP2	36	Pennsylvania	D614	2.43E-05	7.91E-04	1.00E+00
126	TP2	37	Puerto Rico	D614	1.00E-10	9.50E-10	1.00E+00
127	TP2	38	Rhode Island	D614	1.00E-10	4.00E-09	1.00E+00
128	TP2	39	South Carolina	D614	1.00E-10	4.50E-09	9.99E-01
129	TP2	40	Tennessee	D614	1.00E-10	3.30E-09	1.00E+00
130	TP2	41	Texas	D614	1.00E-10	1.48E-09	1.00E+00
131	TP2	42	Utah	D614	1.00E-10	9.75E-10	1.00E+00
132	TP2	43	Virginia	D614	1.00E-10	2.90E-09	1.00E+00
133	TP2	44	Washington	D614	1.00E-10	1.81E-09	1.00E+00
134	TP2	45	Wisconsin	D614	1.00E-10	1.30E-09	1.00E+00
135	TP2	46	USA(others)	D614	1.00E-10	5.10E-09	1.00E+00
136	TP3	1	Alabama	Q677	1.00E-10	3.70E-09	4.12E-01
137	TP3	2	Arizona	Q677	1.00E-10	1.10E-09	1.03E-01
138	TP3	3	Arizona	P681	1.00E-10	1.10E-09	2.48E-01
139	TP3	4	California	P681	1.00E-10	1.14E-09	2.92E-01
140	TP3	5	California	T732	1.00E-10	1.14E-09	2.14E-01
141	TP3	6	Colorado	P681	1.00E-10	1.13E-09	2.28E-01
142	TP3	7	Connecticut	N501	1.00E-10	1.68E-09	3.09E-01
143	TP3	8	Florida	Q677	1.00E-10	2.91E-09	1.85E-01
144	TP3	9	Hawaii	Q677	9.11E-06	6.08E-05	4.57E-01
145	TP3	10	Illinois	Q677	1.00E-10	3.23E-09	1.91E-01
146	TP3	11	Indiana	Q677	4.97E-05	1.49E-03	2.01E-01
147	TP3	12	Louisiana	T478	1.00E-10	1.50E-09	4.27E-01
148	TP3	13	Louisiana	P681	1.00E-10	1.50E-09	5.19E-01
149	TP3	14	Louisiana	T732	1.00E-10	1.50E-09	4.58E-01
150	TP3	15	Maryland	P681	1.00E-10	1.74E-09	2.35E-01
151	TP3	16	Massachusetts	N501	7.76E-05	9.98E-04	2.80E-01
152	TP3	17	Massachusetts	Q677	1.00E-10	2.00E-09	1.82E-01
153	TP3	18	Michigan	G142	1.00E-10	1.02E-09	3.12E-01
154	TP3	19	Michigan	E180	1.00E-10	1.02E-09	3.24E-01
155	TP3	20	Michigan	Q677	1.00E-10	1.02E-09	3.36E-01
156	TP3	21	Michigan	S940	1.00E-10	1.02E-09	2.97E-01
157	TP3	22	Minnesota	Q677	1.00E-10	1.67E-09	1.48E-01
158	TP3	23	New Mexico	Q677	3.03E-06	6.79E-05	2.31E-01
159	TP3	24	New York	P681	1.00E-10	7.97E-10	5.58E-01
160	TP3	25	Pennsylvania	P681	5.30E-04	8.79E-03	1.99E-01
161	TP3	26	Texas	Q677	1.00E-10	1.48E-09	1.48E-01
162	TP3	27	Utah	P681	1.00E-10	9.75E-10	2.35E-01
163	TP3	28	Virginia	P681	1.00E-10	2.90E-09	1.86E-01
164	TP3	29	Wisconsin	Q677	1.00E-10	1.30E-09	1.61E-01
165	TP3	30	Wyoming	S13	1.00E-10	2.17E-09	4.67E-01
166	TP4	1	Alaska	G75	1.00E-10	1.15E-09	9.10E-01
167	TP5	1	Arizona	T478	1.00E-10	1.10E-09	1.03E-01
168	TP5	2	Arizona	T732	1.00E-10	1.10E-09	1.49E-01
169	TP5	3	California	S13	1.00E-10	1.14E-09	1.27E-01
170	TP5	4	California	W152	1.00E-10	1.14E-09	1.15E-01
171	TP5	5	California	L452	1.00E-10	1.14E-09	1.43E-01
172	TP5	6	California	T478	1.00E-10	1.14E-09	2.26E-01
173	TP5	7	Colorado	T478	1.00E-10	1.13E-09	1.70E-01
174	TP5	8	Colorado	A871	1.00E-10	1.13E-09	2.22E-01
175	TP5	9	Colorado	G1167	1.37E-04	1.23E-03	1.13E-01

176	TP5	10	Colorado	M1237	1.00E-10	1.13E-09	2.75E-01
177	TP5	11	Connecticut	P681	4.88E-05	4.91E-04	1.79E-01
178	TP5	12	DC	E484	3.08E-04	3.69E-03	2.72E-01
179	TP5	13	Delaware	Q677	2.21E-04	5.85E-03	1.53E-01
180	TP5	14	Florida	P681	1.00E-10	2.91E-09	1.30E-01
181	TP5	15	Illinois	P681	1.00E-10	3.23E-09	2.00E-01
182	TP5	16	Illinois	T732	1.00E-10	3.23E-09	1.11E-01
183	TP5	17	Indiana	T478	3.60E-06	2.16E-04	1.78E-01
184	TP5	18	Indiana	T732	3.63E-04	7.26E-03	1.51E-01
185	TP5	19	Kansas	P681	2.50E-04	3.41E-03	1.99E-01
186	TP5	20	Kansas	E780	9.05E-05	1.86E-03	3.04E-01
187	TP5	21	Kentucky	G257	1.27E-06	2.17E-05	3.42E-01
188	TP5	22	Maine	A520	1.00E-10	7.30E-09	2.26E-01
189	TP5	23	Maryland	D80	1.00E-10	1.74E-09	4.94E-01
190	TP5	24	Maryland	W152	1.00E-10	1.74E-09	1.36E-01
191	TP5	25	Maryland	S155	1.00E-10	1.74E-09	7.14E-01
192	TP5	26	Maryland	F157	1.00E-10	1.74E-09	6.57E-01
193	TP5	27	Maryland	L452	1.00E-10	1.74E-09	5.69E-01
194	TP5	28	Maryland	E484	1.00E-10	1.74E-09	1.81E-01
195	TP5	29	Maryland	T859	1.00E-10	1.74E-09	6.73E-01
196	TP5	30	Maryland	D950	2.53E-05	3.51E-04	6.28E-01
197	TP5	31	Massachusetts	P681	1.00E-10	2.00E-09	1.33E-01
198	TP5	32	Massachusetts	T732	1.00E-10	2.00E-09	1.28E-01
199	TP5	33	Minnesota	P681	1.00E-10	1.67E-09	1.55E-01
200	TP5	34	Mississippi	P681	4.48E-04	2.69E-03	2.73E-01
201	TP5	35	Nevada	S13	1.00E-10	4.90E-09	2.31E-01
202	TP5	36	Nevada	W152	9.82E-05	1.20E-03	1.53E-01
203	TP5	37	Nevada	L452	6.05E-04	5.39E-03	1.44E-01
204	TP5	38	Nevada	T478	1.74E-04	1.90E-03	1.53E-01
205	TP5	39	Nevada	Q677	3.72E-05	6.08E-04	3.09E-01
206	TP5	40	Nevada	P681	1.26E-06	2.48E-05	1.72E-01
207	TP5	41	Nevada	T732	1.11E-06	2.48E-05	1.50E-01
208	TP5	42	New Jersey	F157	3.92E-05	7.16E-04	2.46E-01
209	TP5	43	New Jersey	L452	3.20E-05	7.16E-04	1.41E-01
210	TP5	44	New Jersey	T478	2.91E-06	1.06E-04	2.00E-01
211	TP5	45	New Jersey	T859	2.66E-04	3.85E-03	1.88E-01
212	TP5	46	New Mexico	T478	4.06E-06	6.79E-05	1.30E-01
213	TP5	47	New Mexico	P681	6.50E-06	9.51E-05	1.60E-01
214	TP5	48	New Mexico	T732	1.00E-10	5.85E-09	1.71E-01
215	TP5	49	New York	L5	1.00E-10	7.97E-10	1.19E-01
216	TP5	50	New York	D80	1.00E-10	7.97E-10	2.76E-01
217	TP5	51	New York	F157	1.00E-10	7.97E-10	2.94E-01
218	TP5	52	New York	L452	1.00E-10	7.97E-10	3.27E-01
219	TP5	53	New York	S494	1.00E-10	7.97E-10	1.91E-01
220	TP5	54	New York	T716	1.00E-10	7.97E-10	1.88E-01
221	TP5	55	New York	T859	1.00E-10	7.97E-10	2.91E-01
222	TP5	56	New York	D950	1.00E-10	7.97E-10	2.65E-01
223	TP5	57	North Carolina	P681	4.22E-05	7.83E-04	1.49E-01
224	TP5	58	Ohio	D80	9.98E-06	1.26E-04	4.58E-01
225	TP5	59	Ohio	L452	2.73E-05	2.80E-04	4.36E-01
226	TP5	60	Ohio	T478	1.00E-10	7.20E-09	2.30E-01
227	TP5	61	Ohio	P681	3.05E-06	6.04E-05	1.83E-01
228	TP5	62	Ohio	T732	1.81E-07	6.52E-06	2.12E-01
229	TP5	63	Ohio	T859	3.35E-06	6.04E-05	4.33E-01
230	TP5	64	Oregon	S13	3.65E-06	5.79E-05	1.72E-01
231	TP5	65	Oregon	Q52	2.68E-04	2.27E-03	1.75E-01
232	TP5	66	Oregon	Q173	7.88E-06	1.11E-04	1.49E-01
233	TP5	67	Oregon	L452	2.02E-06	3.66E-05	1.57E-01
234	TP5	68	Oregon	T478	9.45E-05	9.23E-04	1.35E-01

235	TP5	69	Oregon	Q675	1.00E-10	2.54E-09	1.03E-01
236	TP5	70	Oregon	T732	4.45E-04	3.33E-03	1.00E-01
237	TP5	71	Oregon	S939	1.00E-10	2.54E-09	2.95E-01
238	TP5	72	Pennsylvania	Q677	1.31E-06	1.52E-04	1.21E-01
239	TP5	73	Texas	T478	1.00E-10	1.48E-09	1.01E-01
240	TP5	74	Texas	P681	1.00E-10	1.48E-09	1.67E-01
241	TP5	75	Texas	T732	1.00E-10	1.48E-09	1.41E-01
242	TP5	76	Utah	D138	1.00E-10	9.75E-10	1.33E-01
243	TP5	77	Utah	T478	1.00E-10	9.75E-10	2.44E-01
244	TP5	78	Utah	T732	5.14E-04	3.49E-03	2.14E-01
245	TP5	79	Utah	E1144	1.00E-10	9.75E-10	2.65E-01
246	TP5	80	Virginia	E484	1.00E-10	2.90E-09	1.30E-01
247	TP5	81	Virginia	Q677	1.00E-10	2.90E-09	1.70E-01
248	TP5	82	Washington	D178	1.00E-10	1.81E-09	3.85E-01
249	TP5	83	Washington	L452	9.34E-05	8.44E-04	2.53E-01
250	TP5	84	Washington	Q677	1.00E-10	1.81E-09	1.19E-01
251	TP5	85	Washington	T732	1.00E-10	1.81E-09	1.31E-01
252	TP5	86	Wyoming	W152	1.00E-10	2.17E-09	1.83E-01
253	TP5	87	Wyoming	W258	1.00E-10	2.17E-09	2.14E-01
254	TP5	88	Wyoming	L452	1.00E-10	2.17E-09	1.71E-01
255	TP5	89	Wyoming	P681	1.00E-10	2.17E-09	1.49E-01
256	TP6	1	Colorado	T732	1.00E-10	1.13E-09	3.97E-01
257	TP6	2	Connecticut	T29	1.00E-10	1.68E-09	4.90E-01
258	TP6	3	Connecticut	T572	1.00E-10	1.68E-09	5.63E-01
259	TP6	4	Connecticut	Q677	1.00E-10	1.68E-09	6.30E-01
260	TP6	5	Connecticut	D936	1.00E-10	1.68E-09	6.40E-01
261	TP6	6	Georgia	D138	1.00E-10	6.15E-09	9.05E-01
262	TP7	1	DC	D1168	1.57E-04	2.83E-03	5.13E-01
263	TP7	2	DC	V1264	3.27E-07	1.18E-05	8.18E-01
264	TP7	3	Louisiana	Q677	1.00E-10	1.50E-09	5.11E-01
265	TP8	1	Hawaii	P681	1.00E-10	2.00E-09	9.87E-01
266	TP9	1	Mississippi	L5	3.42E-04	2.69E-03	8.54E-01

Table S4. Frequencies of the 90 viral residue variants (VRVs) by state/territory, from an unsupervised learning from bi-clustering of all States and VRVs.

ID	VRV	Freq	VRV cluster	Alabama	Alaska	Arizona	Arkansas	California	Colorado	Connecticut	DC	Delaware	Florida	Georgia	Hawaii	Idaho	Illinois	Indiana	Iowa	Kansas	Kentucky	Louisiana	Maine	Maryland	Massachusetts	Michigan	Minnesota	Mississippi	Missouri	Montana	Nebraska	Nevada	New Hampshire	New Jersey	New Mexico	New York	North Carolina	North Dakota	Ohio	Oklahoma	Oregon	Pennsylvania	Puerto Rico	Rhode Island	South Carolina	Tennessee	Texas	Utah	Virginia	Washington	Wisconsin	Wyoming	Other
		Freq		3	3	10	1	9	12	8	3	2	5	3	3	0	5	4	1	3	2	7	4	9	9	10	9	3	1	1	2	11	1	6	8	16	6	1	7	1	11	5	3	1	1	1	10	11	6	10	10	7	1
		State cluster #		1	2	3	4	5	3	1	2	1	1	2	1	2	1	3	4	1	4	3	2	6	1	1	1	1	4	4	4	5	4	6	3	6	2	4	6	4	5	1	2	4	4	4	3	3	1	5	1	5	4
1	L5	4	1						1																		1	1								1																	
2	S13	6	2					1																								1									1							1		1		1	
3	T20	1	3																																								1										
4	T22	1	3																																													1					
5	A27	1	4																							1																											
6	T29	1	5							1																																											
7	H49	1	2																													1																					
8	Q52	2	2			1																																			1												
9	L54	2	6																																	1											1						
10	A67	1	5																																		1																
11	G72	1	5																		1																																
12	G75	1	5		1																																																
13	D80	3	6																					1												1			1														
14	T95	1	4																							1																											
15	E132	1	6																																	1																	
16	D138	3	5											1																							1											1					
17	L141	1	2					1																																													
18	G142	1	4																							1																											
19	W152	5	2					1																1								1																		1		1	
20	S155	1	6																					1																													
21	F157	4	6																					1										1		1															1		
22	Q173	6	5						1								1								1																1	1										1	
23	D178	2	2																																						1									1			
24	E180	2	5																							1									1																		
25	G181	1	5																																1																		
26	D215	1	5																																																	1	
27	F220	1	5										1																																								
28	A222	1	1																								1																										
29	H245	1	7			1																																															
30	D253	2	3																																								1					1					
31	G257	1	5																		1																																
32	W258	1	5																																																	1	
33	A263	2	5											1																																			1				

34	S359	1	8						1																																												
35	V382	1	2					1																																													
36	K444	1	5																																		1																
37	L452	9	2					1																1								1		1		1			1		1									1		1	
38	Y453	1	6																																	1																	
39	T478	13	2			1		1	1									1				1										1		1	1	1			1		1						1	1					
40	V483	1	2																																															1			
41	E484	6	6	1							1												1	1												1													1				
42	F486	1	4																							1																											
43	S494	2	1																								1									1																	
44	N501	5	5							1															1	1																								1	1		
45	S514	1	7			1																																															
46	A520	2	5																				1																								1						
47	E554	1	5																																		1																
48	K558	1	7			1																																															
49	T572	2	5							1												1																															
50	A575	1	1																																																1		
51	E583	1	1																																																1		
52	D614	46	2	1	1	1	1	1	1	1		1	1	1	1		1	1	1	1	1	1	1		1	1	1	1	1	1	1	1	1	1	1	1	1	1	1	1	1	1	1	1	1	1	1	1	1	1	1		1
53	N641	1	5																																												1						
54	Q675	3	2																													1									1										1		
55	Q677	19	2	1		1				1		1	1		1		1	1				1			1	1	1					1			1							1					1		1	1	1		
56	N679	2	5																															1								1											
57	P681	24	2			1		1	1	1			1		1		1			1		1		1	1		1	1				1			1	1	1		1			1					1	1	1		1	1	
58	A684	1	2																																															1			
59	S698	1	3																																													1					
60	A701	2	5																																1														1				
61	T716	1	6																																	1																	
62	M731	1	5																																												1						
63	T732	16	2			1		1	1	1							1	1				1			1							1			1	1			1		1						1	1		1			
64	E780	3	1																	1							1																								1		
65	A845	1	5																						1																												
66	N856	1	5																												1																						
67	T859	5	6																					1			1							1		1			1														
68	A871	1	8						1																																												
69	D936	1	5							1																																											
70	S939	1	2																																						1												
71	S940	1	4																							1																											
72	S943	1	5																																												1						
73	D950	2	6																					1												1																	
74	N1023	1	8						1																																												
75	V1040	1	1																								1																										
76	K1073	2	2																						1							1																					

77	A1078	1	8			1								
78	G1085	1	4								1			
79	V1133	1	5							1				
80	E1144	1	3										1	
81	D1163	1	7		1									
82	V1164	1	5					1						
83	G1167	1	8			1								
84	D1168	1	5				1							
85	N1187	2	3						1				1	
86	K1191	1	2									1		
87	V1228	1	5	1										
88	M1229	1	1											1
89	M1237	1	8			1								
90	V1264	1	5				1							

“Other” includes sequences from South Dakota, Vermont, and the Virgin Islands, in addition to sequences for which state information was missing.

Table S5. VRV-haplotypes identified within each state/territory, along with state/territory-specific frequencies. The "positivity" column indicates the proportion of mutations in each haplotype block.

ID	state	row	Freq	col	#	Hap	positivity
1	Alabama	PG1	1441	D614	1	D614	1.00
2	Alabama	PG2	507	D614,Q677	2	D614-Q677	1.00
3	Alabama	PG3	6	E484,D614	2	E484-D614	1.00
4	Alabama	PG4	1	E484,D614,Q677	3	E484-D614-Q677	1.00
5	Alabama	PG5	1	E484,D614	2	E484-D614	1.00
6	Alabama	PG6	1	E484,D614	2	E484-D614	1.00
7	Alabama	PG7	1	E484,D614	2	E484-D614	1.00
8	Alabama	PG8	1	E484,D614	2	E484-D614	1.00
9	Alaska	PG1	515	D614	1	D614	1.00
10	Alaska	PG3	16	D614,V1228	2	D614-V1228	1.00
11	Alaska	PG4	33	G75,D614	2	G75-D614	1.00
12	Alaska	PG5	1	G75,D614	2	G75-D614	1.00
13	Alaska	PG6	1	G75,D614	2	G75-D614	1.00
14	Alaska	PG7	1	G75,D614	2	G75-D614	1.00
15	Alaska	PG8	1	G75,D614	2	G75-D614	1.00
16	Arizona	PG1	5451	RG3	1	D614	1.00
17	Arizona	PG4	278	RG2,RG3	2	H245-D614	1.00
18	Arizona	PG5	220	RG3,RG4	2	D614-Q677	1.00
19	Arizona	PG6	28	RG1,RG3	7	Q52-T478-S514-K558-D614-T732-D1163	0.58
20	Arizona	PG7	50	RG1,RG3	7	Q52-T478-S514-K558-D614-T732-D1163	0.65
21	Arizona	PG8	310	RG3,RG5	2	D614-P681	1.00
22	Arizona	PG9	33	RG1,RG3	7	Q52-T478-S514-K558-D614-T732-D1163	0.58
23	Arizona	PG10	69	RG1,RG3,RG4	8	Q52-T478-S514-K558-D614-Q677-T732-D1163	0.72
24	Arizona	PG11	73	RG1,RG3	7	Q52-T478-S514-K558-D614-T732-D1163	0.58
25	Arizona	PG12	33	RG1,RG3,RG5	8	Q52-T478-S514-K558-D614-P681-T732-D1163	0.77
26	Arkansas	PG1	390	D614	1	D614	1.00
27	California	PG2	15193	RG3	1	D614	1.00
28	California	PG3	522	RG3,RG4	2	D614-P681	1.00
29	California	PG4	485	RG3,RG5	2	D614-T732	1.00
30	California	PG5	52	RG1,RG3	4	S13-W152-L452-D614	0.64
31	California	PG6	21	RG2,RG3,RG5	5	L141-V382-T478-D614-T732	0.52
32	California	PG7	73	RG2,RG3	4	L141-V382-T478-D614	0.67
33	California	PG8	88	RG2,RG3	4	L141-V382-T478-D614	0.67
34	California	PG9	116	RG1,RG3	4	S13-W152-L452-D614	0.67
35	California	PG10	11	RG1,RG3,RG4	5	S13-W152-L452-D614-P681	0.51
36	California	PG11	287	RG2,RG3,RG4,RG5	6	L141-V382-T478-D614-P681-T732	0.83
37	California	PG12	25	RG1,RG3	4	S13-W152-L452-D614	0.83
38	California	PG13	328	RG1,RG3	4	S13-W152-L452-D614	1.00
39	California	PG14	18	RG1,RG3	4	S13-W152-L452-D614	0.83
40	California	PG15	12	RG1,RG3,RG5	5	S13-W152-L452-D614-T732	0.62
41	California	PG16	14	RG2,RG3,RG4	5	L141-V382-T478-D614-P681	0.78
42	Colorado	PG1	1368	RG4	1	D614	1.00
43	Colorado	PG3	42	RG1,RG4	7	L5-S359-D614-A871-A1078-G1167-M1237	0.67
44	Colorado	PG4	35	RG2,RG4	3	Q173-D614-N1023	0.75
45	Colorado	PG5	158	RG4,RG5	2	D614-T732	1.00
46	Colorado	PG6	95	RG3,RG4	3	T478-D614-P681	0.76
47	Colorado	PG7	21	RG1,RG4	7	L5-S359-D614-A871-A1078-G1167-M1237	0.58
48	Colorado	PG8	85	RG1,RG4	7	L5-S359-D614-A871-A1078-G1167-M1237	0.58
49	Colorado	PG9	42	RG1,RG4	7	L5-S359-D614-A871-A1078-G1167-M1237	0.66
50	Colorado	PG11	60	RG3,RG4,RG5	4	T478-D614-P681-T732	0.98
51	Colorado	PG12	51	RG2,RG4	3	Q173-D614-N1023	1.00
52	Connecticut	PG1	1147	RG3	1	D614	1.00
53	Connecticut	PG3	7	RG1,RG3	5	T29-T572-D614-Q677-D936	0.88
54	Connecticut	PG4	188	RG2,RG3	2	N501-D614	1.00
55	Connecticut	PG5	59	RG1,RG3	5	T29-T572-D614-Q677-D936	1.00
56	Connecticut	PG6	65	RG3,RG4	2	D614-P681	1.00

57	Connecticut	PG7	13	RG1,RG3	5	T29-T572-D614-Q677-D936	0.88
58	Connecticut	PG8	6	RG1,RG3,RG5	6	T29-T572-D614-Q677-T732-D936	0.72
59	Connecticut	PG9	34	RG1,RG3	5	T29-T572-D614-Q677-D936	0.63
60	Connecticut	PG10	4	RG1,RG2,RG3	6	T29-N501-T572-D614-Q677-D936	0.56
61	Connecticut	PG11	18	RG3,RG4,RG5	3	D614-P681-T732	1.00
62	Connecticut	PG12	10	RG2,RG3,RG4	3	N501-D614-P681	1.00
63	DC	PG2	10	V1264	1	V1264	1.00
64	DC	PG3	1	D1168	1	D1168	1.00
65	DC	PG4	14	E484	1	E484	1.00
66	DC	PG5	20	D1168,V1264	2	D1168-V1264	1.00
67	DC	PG6	1	D1168,V1264	2	D1168-V1264	1.00
68	DC	PG7	1	D1168,V1264	2	D1168-V1264	1.00
69	DC	PG8	1	D1168,V1264	2	D1168-V1264	1.00
70	Delaware	PG1	703	D614	1	D614	1.00
71	Delaware	PG3	36	D614,Q677	2	D614-Q677	1.00
72	Delaware	PG4	1	D614,Q677	2	D614-Q677	1.00
73	Florida	PG2	5941	RG2	1	D614	1.00
74	Florida	PG3	59	RG1,RG2	2	F220-D614	1.00
75	Florida	PG4	515	RG2,RG3	2	D614-Q677	1.00
76	Florida	PG5	47	RG2,RG5	2	D614-V1164	1.00
77	Florida	PG6	364	RG2,RG4	2	D614-P681	1.00
78	Florida	PG7	1	RG2,RG3,RG4	3	D614-Q677-P681	1.00
79	Florida	PG8	1	RG2,RG4	2	D614-P681	1.00
80	Florida	PG9	1	RG2,RG4	2	D614-P681	1.00
81	Florida	PG10	1	RG2,RG4	2	D614-P681	1.00
82	Florida	PG11	1	RG2,RG4	2	D614-P681	1.00
83	Florida	PG12	1	RG2,RG4	2	D614-P681	1.00
84	Georgia	PG2	2413	D614	1	D614	1.00
85	Georgia	PG3	104	D138,D614	2	D138-D614	1.00
86	Georgia	PG4	11	A263,D614	2	A263-D614	1.00
87	Georgia	PG5	1	A263,D614	2	A263-D614	1.00
88	Georgia	PG6	1	A263,D614	2	A263-D614	1.00
89	Georgia	PG7	1	A263,D614	2	A263-D614	1.00
90	Georgia	PG8	1	A263,D614	2	A263-D614	1.00
91	Hawaii	PG1	235	D614	1	D614	1.00
92	Hawaii	PG3	724	D614,P681	2	D614-P681	1.00
93	Hawaii	PG4	6	D614,Q677	2	D614-Q677	1.00
94	Hawaii	PG5	1	D614,Q677	2	D614-Q677	1.00
95	Hawaii	PG6	1	D614,Q677	2	D614-Q677	1.00
96	Hawaii	PG7	1	D614,Q677	2	D614-Q677	1.00
97	Hawaii	PG8	1	D614,Q677	2	D614-Q677	1.00
98	Illinois	PG2	2643	RG2	1	D614	1.00
99	Illinois	PG3	17	RG1,RG2	2	Q173-D614	1.00
100	Illinois	PG4	165	RG2,RG3	2	D614-Q677	1.00
101	Illinois	PG5	28	RG2,RG5	2	D614-T732	1.00
102	Illinois	PG6	63	RG2,RG4	2	D614-P681	1.00
103	Illinois	PG7	60	RG2,RG4,RG5	3	D614-P681-T732	1.00
104	Illinois	PG8	1	RG2,RG3,RG5	3	D614-Q677-T732	1.00
105	Illinois	PG9	1	RG2,RG3,RG4	3	D614-Q677-P681	1.00
106	Illinois	PG10	1	RG2,RG3,RG5	3	D614-Q677-T732	1.00
107	Illinois	PG11	1	RG2,RG3,RG5	3	D614-Q677-T732	1.00
108	Illinois	PG12	1	RG2,RG3,RG5	3	D614-Q677-T732	1.00
109	Illinois	PG13	1	RG2,RG3,RG5	3	D614-Q677-T732	1.00
110	Illinois	PG14	1	RG2,RG3,RG5	3	D614-Q677-T732	1.00
111	Illinois	PG15	1	RG2,RG3,RG5	3	D614-Q677-T732	1.00
112	Illinois	PG16	1	RG2,RG3,RG4	3	D614-Q677-P681	1.00
113	Indiana	PG1	998	RG2	1	D614	1.00
114	Indiana	PG3	111	RG2,RG3	2	D614-Q677	1.00
115	Indiana	PG4	52	RG1,RG2,RG4	3	T478-D614-T732	1.00

116	Indiana	PG5	2	RG2,RG3,RG4	3	D614-Q677-T732	1.00
117	Indiana	PG6	5	RG2,RG4	2	D614-T732	1.00
118	Indiana	PG7	5	RG1,RG2	2	T478-D614	1.00
119	Indiana	PG8	1	RG1,RG2	2	T478-D614	1.00
120	Indiana	PG9	1	RG1,RG2	2	T478-D614	1.00
121	Indiana	PG10	1	RG1,RG2	2	T478-D614	1.00
122	Indiana	PG11	1	RG1,RG2	2	T478-D614	1.00
123	Indiana	PG12	1	RG1,RG2	2	T478-D614	1.00
124	Iowa	PG1	288	D614	1	D614	1.00
125	Iowa	PG2	1	D614	1	D614	1.00
126	Kansas	PG1	806	D614	1	D614	1.00
127	Kansas	PG3	43	D614,P681	2	D614-P681	1.00
128	Kansas	PG4	7	D614,E780	2	D614-E780	1.00
129	Kansas	PG5	1	D614,E780	2	D614-E780	1.00
130	Kansas	PG6	1	D614,E780	2	D614-E780	1.00
131	Kansas	PG7	1	D614,E780	2	D614-E780	1.00
132	Kansas	PG8	1	D614,E780	2	D614-E780	1.00
133	Kentucky	PG1	548	D614	1	D614	1.00
134	Kentucky	PG3	28	G257,D614	2	G257-D614	1.00
135	Kentucky	PG4	1	G257,D614	2	G257-D614	1.00
136	Louisiana	PG1	2843	RG3	1	D614	1.00
137	Louisiana	PG2	35	RG2,RG3	2	T572-D614	1.00
138	Louisiana	PG4	2	RG1,RG3	4	T478-D614-P681-T732	0.67
139	Louisiana	PG5	64	RG3,RG5	2	D614-N1187	1.00
140	Louisiana	PG6	363	RG3,RG4	2	D614-Q677	1.00
141	Louisiana	PG7	34	RG1,RG3	4	T478-D614-P681-T732	0.67
142	Louisiana	PG8	3	RG1,RG3	4	T478-D614-P681-T732	0.67
143	Louisiana	PG9	98	RG1,RG3	4	T478-D614-P681-T732	1.00
144	Louisiana	PG10	18	RG1,RG3	4	T478-D614-P681-T732	0.83
145	Louisiana	PG11	3	RG1,RG3	4	T478-D614-P681-T732	0.83
146	Louisiana	PG12	1	RG1,RG3,RG4	5	T478-D614-Q677-P681-T732	1.00
147	Maine	PG1	1472	RG4	1	D614	1.00
148	Maine	PG3	39	RG1,RG4	2	G72-D614	1.00
149	Maine	PG4	27	RG3,RG4	2	A520-D614	1.00
150	Maine	PG5	17	RG2,RG4	2	E484-D614	1.00
151	Maine	PG6	1	RG2,RG4	2	E484-D614	1.00
152	Maine	PG7	1	RG2,RG4	2	E484-D614	1.00
153	Maine	PG8	1	RG2,RG4	2	E484-D614	1.00
154	Maine	PG9	1	RG2,RG4	2	E484-D614	1.00
155	Maine	PG10	1	RG2,RG4	2	E484-D614	1.00
156	Maine	PG11	1	RG2,RG4	2	E484-D614	1.00
157	Maine	PG12	1	RG2,RG4	2	E484-D614	1.00
158	Maryland	PG2	384	RG5	1	P681	1.00
159	Maryland	PG3	24	RG2,RG3	6	W152-S155-F157-L452-T859-D950	0.57
160	Maryland	PG4	76	RG4,RG5	2	E484-P681	1.00
161	Maryland	PG5	54	RG2,RG4	2	W152-E484	1.00
162	Maryland	PG6	8	RG1,RG4,RG5	3	D80-E484-P681	0.50
163	Maryland	PG9	29	RG4	1	E484	1.00
164	Maryland	PG10	35	RG3	5	S155-F157-L452-T859-D950	1.00
165	Maryland	PG11	5	RG1,RG3	6	D80-S155-F157-L452-T859-D950	0.76
166	Maryland	PG12	29	RG1,RG3	6	D80-S155-F157-L452-T859-D950	0.92
167	Massachusetts	PG2	3455	RG3	1	D614	1.00
168	Massachusetts	PG3	71	RG1,RG3	5	Q173-D614-A845-K1073-V1133	0.63
169	Massachusetts	PG4	379	RG2,RG3	2	N501-D614	1.00
170	Massachusetts	PG5	137	RG3,RG5	2	D614-P681	1.00
171	Massachusetts	PG6	32	RG1,RG3	5	Q173-D614-A845-K1073-V1133	0.63
172	Massachusetts	PG7	21	RG1,RG3	5	Q173-D614-A845-K1073-V1133	0.63
173	Massachusetts	PG8	239	RG3,RG4	3	D614-Q677-T732	0.75
174	Massachusetts	PG9	147	RG3,RG4	3	D614-Q677-T732	1.00

175	Massachusetts	PG10	45	RG1,RG2,RG3	6	Q173-N501-D614-A845-K1073-V1133	0.72
176	Massachusetts	PG11	57	RG3,RG4,RG5	4	D614-Q677-P681-T732	0.80
177	Massachusetts	PG12	12	RG2,RG3,RG5	3	N501-D614-P681	1.00
178	Michigan	PG1	6941	RG4	1	D614	1.00
179	Michigan	PG3	21	RG2,RG4	4	T95-F486-N501-D614	0.67
180	Michigan	PG4	27	RG1,RG4	3	A27-D614-G1085	0.75
181	Michigan	PG5	4	RG3,RG4	4	G142-E180-D614-S940	0.67
182	Michigan	PG6	19	RG3,RG4	4	G142-E180-D614-S940	0.67
183	Michigan	PG7	275	RG4,RG5	2	D614-Q677	1.00
184	Michigan	PG8	134	RG1,RG4	3	A27-D614-G1085	1.00
185	Michigan	PG9	4	RG2,RG4	4	T95-F486-N501-D614	0.96
186	Michigan	PG10	53	RG2,RG3,RG4	7	T95-G142-E180-F486-N501-D614-S940	0.67
187	Michigan	PG11	35	RG2,RG4	4	T95-F486-N501-D614	0.67
188	Michigan	PG12	348	RG3,RG4,RG5	5	G142-E180-D614-Q677-S940	1.00
189	Michigan	PG13	4	RG3,RG4,RG5	5	G142-E180-D614-Q677-S940	0.86
190	Michigan	PG14	27	RG3,RG4,RG5	5	G142-E180-D614-Q677-S940	0.86
191	Michigan	PG15	2	RG3,RG4,RG5	5	G142-E180-D614-Q677-S940	0.61
192	Michigan	PG16	3	RG3,RG4,RG5	5	G142-E180-D614-Q677-S940	0.78
193	Minnesota	PG1	2385	RG3	1	D614	1.00
194	Minnesota	PG3	4	RG3,RG5	3	D614-E780-T859	0.75
195	Minnesota	PG4	18	RG2,RG3	5	A222-S494-D614-P681-V1040	0.63
196	Minnesota	PG5	130	RG1,RG3	2	L5-D614	1.00
197	Minnesota	PG6	49	RG3,RG5	3	D614-E780-T859	0.75
198	Minnesota	PG7	1	RG1	1	L5	1.00
199	Minnesota	PG8	194	RG3,RG4	2	D614-Q677	1.00
200	Minnesota	PG9	97	RG2,RG3	5	A222-S494-D614-P681-V1040	0.63
201	Minnesota	PG10	14	RG2,RG3	5	A222-S494-D614-P681-V1040	0.63
202	Minnesota	PG11	57	RG2,RG3	5	A222-S494-D614-P681-V1040	0.63
203	Minnesota	PG12	58	RG3,RG5	3	D614-E780-T859	1.00
204	Minnesota	PG13	28	RG1,RG3,RG4	3	L5-D614-Q677	1.00
205	Minnesota	PG14	6	RG2,RG3	5	A222-S494-D614-P681-V1040	0.75
206	Minnesota	PG15	1	RG1,RG3,RG5	4	L5-D614-E780-T859	0.83
207	Minnesota	PG16	1	RG1,RG2,RG3	6	L5-A222-S494-D614-P681-V1040	0.75
208	Mississippi	PG1	331	D614	1	D614	1.00
209	Mississippi	PG3	14	L5,D614	2	L5-D614	1.00
210	Mississippi	PG4	9	D614,P681	2	D614-P681	1.00
211	Mississippi	PG5	1	D614,P681	2	D614-P681	1.00
212	Mississippi	PG6	1	D614,P681	2	D614-P681	1.00
213	Mississippi	PG7	1	D614,P681	2	D614-P681	1.00
214	Mississippi	PG8	1	D614,P681	2	D614-P681	1.00
215	Missouri	PG1	700	D614	1	D614	1.00
216	Montana	PG2	207	D614	1	D614	1.00
217	Nebraska	PG2	24	D614,N856	2	D614-N856	1.00
218	Nebraska	PG3	443	D614	1	D614	1.00
219	Nebraska	PG4	1	D614	1	D614	1.00
220	Nevada	PG2	1279	RG4	1	D614	1.00
221	Nevada	PG3	48	RG2,RG4	4	H49-D614-Q675-K1073	0.67
222	Nevada	PG4	98	RG4,RG5	2	D614-Q677	1.00
223	Nevada	PG5	41	RG2,RG4	4	H49-D614-Q675-K1073	0.67
224	Nevada	PG6	16	RG2,RG4	4	H49-D614-Q675-K1073	0.67
225	Nevada	PG7	37	RG3,RG4	4	T478-D614-P681-T732	0.67
226	Nevada	PG8	52	RG1,RG4	4	S13-W152-L452-D614	1.00
227	Nevada	PG9	10	RG1,RG4	4	S13-W152-L452-D614	0.77
228	Nevada	PG10	10	RG1,RG4	4	S13-W152-L452-D614	0.75
229	Nevada	PG11	28	RG3,RG4,RG5	5	T478-D614-Q677-P681-T732	0.52
230	Nevada	PG12	40	RG3,RG4	4	T478-D614-P681-T732	0.98
231	New Hampshire	PG1	450	D614	1	D614	1.00
232	New Jersey	PG1	1007	RG4	1	D614	1.00
233	New Jersey	PG3	2	RG1,RG4	3	F157-D614-T859	0.75

234	New Jersey	PG4	15	RG4,RG5	2	D614-N679	1.00
235	New Jersey	PG5	12	RG2,RG4	2	L452-D614	1.00
236	New Jersey	PG6	8	RG1,RG2,RG4	4	F157-L452-D614-T859	0.83
237	New Jersey	PG7	27	RG3,RG4	2	T478-D614	1.00
238	New Jersey	PG8	3	RG1,RG4	3	F157-D614-T859	0.75
239	New Jersey	PG9	9	RG1,RG2,RG4	4	F157-L452-D614-T859	1.00
240	New Jersey	PG10	1	RG1,RG2,RG4	4	F157-L452-D614-T859	1.00
241	New Jersey	PG11	1	RG1,RG2,RG4	4	F157-L452-D614-T859	1.00
242	New Jersey	PG12	1	RG1,RG2,RG4	4	F157-L452-D614-T859	1.00
243	New Mexico	PG1	2099	RG2	1	D614	1.00
244	New Mexico	PG3	53	RG2,RG5	2	D614-T732	1.00
245	New Mexico	PG4	25	RG1,RG2	5	E180-G181-T478-D614-A701	0.63
246	New Mexico	PG5	27	RG1,RG2	5	E180-G181-T478-D614-A701	0.63
247	New Mexico	PG6	114	RG2,RG4	2	D614-P681	1.00
248	New Mexico	PG7	13	RG1,RG2	5	E180-G181-T478-D614-A701	0.63
249	New Mexico	PG8	55	RG2,RG3,RG5	3	D614-Q677-T732	1.00
250	New Mexico	PG9	276	RG2,RG3	2	D614-Q677	1.00
251	New Mexico	PG11	28	RG1,RG2,RG4,RG5	7	E180-G181-T478-D614-P681-A701-T732	0.81
252	New Mexico	PG12	3	RG1,RG2,RG3	6	E180-G181-T478-D614-Q677-A701	0.75
253	New York	PG1	10822	RG4	1	D614	1.00
254	New York	PG3	284	RG1,RG4	8	L5-L54-E132-Y453-T478-E484-D614-T732	0.55
255	New York	PG5	82	RG1,RG4	8	L5-L54-E132-Y453-T478-E484-D614-T732	0.58
256	New York	PG6	132	RG2,RG4,RG5	7	D80-F157-L452-D614-P681-T859-D950	0.68
257	New York	PG7	1161	RG4,RG5	2	D614-P681	1.00
258	New York	PG8	141	RG2,RG4	6	D80-F157-L452-D614-T859-D950	0.61
259	New York	PG9	226	RG1,RG4	8	L5-L54-E132-Y453-T478-E484-D614-T732	0.59
260	New York	PG10	59	RG1,RG4	8	L5-L54-E132-Y453-T478-E484-D614-T732	0.57
261	New York	PG11	360	RG3,RG4,RG5	4	S494-D614-P681-T716	1.00
262	New York	PG12	102	RG3,RG4,RG5	4	S494-D614-P681-T716	0.67
263	New York	PG13	470	RG2,RG4	6	D80-F157-L452-D614-T859-D950	1.00
264	New York	PG14	172	RG1,RG4,RG5	9	L5-L54-E132-Y453-T478-E484-D614-P681-T732	0.76
265	New York	PG15	52	RG3,RG4,RG5	4	S494-D614-P681-T716	0.74
266	New York	PG16	26	RG2,RG4	6	D80-F157-L452-D614-T859-D950	0.83
267	North Carolina	PG1	2450	RG4	1	D614	1.00
268	North Carolina	PG3	11	RG2,RG4	3	D138-E554-D614	1.00
269	North Carolina	PG4	2	RG2,RG4	3	D138-E554-D614	0.75
270	North Carolina	PG5	233	RG4,RG5	2	D614-P681	1.00
271	North Carolina	PG6	39	RG1,RG4	2	A67-D614	1.00
272	North Carolina	PG7	13	RG2,RG4	3	D138-E554-D614	0.75
273	North Carolina	PG8	3	RG5	1	P681	1.00
274	North Carolina	PG9	5	RG1,RG4,RG5	3	A67-D614-P681	1.00
275	North Carolina	PG10	13	RG3,RG4	2	K444-D614	1.00
276	North Carolina	PG11	1	RG3,RG4	2	K444-D614	1.00
277	North Carolina	PG12	1	RG3,RG4	2	K444-D614	1.00
278	North Dakota	PG1	344	D614	1	D614	1.00
279	Ohio	PG1	1286	RG3	1	D614	1.00
280	Ohio	PG3	3	RG3,RG5	2	D614-T732	1.00
281	Ohio	PG4	35	RG3,RG4	2	D614-P681	1.00
282	Ohio	PG5	40	RG2,RG3,RG4,RG5	4	T478-D614-P681-T732	1.00
283	Ohio	PG6	6	RG1,RG3	4	D80-L452-D614-T859	0.69
284	Ohio	PG7	6	RG1,RG3	4	D80-L452-D614-T859	0.67
285	Ohio	PG8	8	RG2,RG3,RG4	3	T478-D614-P681	0.96
286	Ohio	PG9	2	RG1,RG3,RG4	5	D80-L452-D614-P681-T859	0.78
287	Ohio	PG10	3	RG1,RG3	4	D80-L452-D614-T859	0.67
288	Ohio	PG11	4	RG3,RG4,RG5	3	D614-P681-T732	1.00
289	Ohio	PG12	8	RG1,RG3	4	D80-L452-D614-T859	1.00
290	Oklahoma	PG2	365	D614	1	D614	1.00
291	Oregon	PG2	2807	RG4	1	D614	1.00
292	Oregon	PG3	49	RG3,RG4	3	T478-D614-T732	0.75

293	Oregon	PG4	154	RG2,RG4	2	D178-D614	1.00
294	Oregon	PG5	27	RG1,RG4	7	S13-Q52-Q173-L452-D614-Q675-S939	0.58
295	Oregon	PG6	10	RG1,RG4	7	S13-Q52-Q173-L452-D614-Q675-S939	0.62
296	Oregon	PG7	17	RG1,RG4	7	S13-Q52-Q173-L452-D614-Q675-S939	0.58
297	Oregon	PG8	19	RG1,RG4	7	S13-Q52-Q173-L452-D614-Q675-S939	0.59
298	Oregon	PG9	59	RG4,RG5	2	D614-K1191	1.00
299	Oregon	PG10	27	RG3,RG4	3	T478-D614-T732	1.00
300	Oregon	PG11	18	RG1,RG4	7	S13-Q52-Q173-L452-D614-Q675-S939	0.62
301	Oregon	PG12	6	RG3,RG4	3	T478-D614-T732	0.75
302	Pennsylvania	PG1	1768	RG2	1	D614	1.00
303	Pennsylvania	PG3	177	RG2,RG3	2	D614-Q677	1.00
304	Pennsylvania	PG4	15	RG1,RG2	2	Q173-D614	1.00
305	Pennsylvania	PG5	376	RG2,RG5	2	D614-P681	1.00
306	Pennsylvania	PG6	7	RG2,RG4	2	D614-N679	1.00
307	Pennsylvania	PG7	1	RG2,RG4	2	D614-N679	1.00
308	Pennsylvania	PG8	1	RG2,RG4	2	D614-N679	1.00
309	Pennsylvania	PG9	1	RG2,RG4	2	D614-N679	1.00
310	Pennsylvania	PG10	1	RG2,RG4	2	D614-N679	1.00
311	Pennsylvania	PG11	1	RG2,RG4	2	D614-N679	1.00
312	Pennsylvania	PG12	1	RG2,RG4	2	D614-N679	1.00
313	Puerto Rico	PG2	201	D614	1	D614	1.00
314	Puerto Rico	PG3	73	T20,D614	2	T20-D614	1.00
315	Puerto Rico	PG4	21	D253,D614	2	D253-D614	1.00
316	Puerto Rico	PG5	1	D253	1	D253	1.00
317	Puerto Rico	PG6	1	D253,D614	2	D253-D614	1.00
318	Puerto Rico	PG7	1	D253,D614	2	D253-D614	1.00
319	Puerto Rico	PG8	1	D253,D614	2	D253-D614	1.00
320	Rhode Island	PG1	570	D614	1	D614	1.00
321	South Carolina	PG2	782	D614	1	D614	1.00
322	Tennessee	PG1	456	D614	1	D614	1.00
323	Texas	PG1	25256	RG3	1	D614	1.00
324	Texas	PG3	245	RG1,RG3	6	L54-A520-D614-N641-M731-S943	0.60
325	Texas	PG4	1255	RG3,RG4	2	D614-Q677	1.00
326	Texas	PG5	222	RG1,RG3	6	L54-A520-D614-N641-M731-S943	0.70
327	Texas	PG6	243	RG1,RG3	6	L54-A520-D614-N641-M731-S943	0.60
328	Texas	PG7	62	RG1,RG3	6	L54-A520-D614-N641-M731-S943	0.60
329	Texas	PG8	275	RG2,RG3	3	T478-D614-T732	0.75
330	Texas	PG9	656	RG3,RG5	2	D614-P681	1.00
331	Texas	PG10	18	RG1,RG3	6	L54-A520-D614-N641-M731-S943	0.60
332	Texas	PG11	32	RG2,RG3	3	T478-D614-T732	0.75
333	Texas	PG12	53	RG2,RG3,RG4	4	T478-D614-Q677-T732	0.83
334	Texas	PG13	461	RG2,RG3,RG5	4	T478-D614-P681-T732	1.00
335	Texas	PG14	15	RG2,RG3,RG5	4	T478-D614-P681-T732	0.83
336	Texas	PG15	27	RG2,RG4,RG5	4	T478-Q677-P681-T732	0.83
337	Texas	PG16	76	RG2	2	T478-T732	0.50
338	Utah	PG2	5061	RG4	1	D614	1.00
339	Utah	PG3	785	RG3,RG4	2	D253-D614	1.00
340	Utah	PG5	63	RG2,RG4	3	T22-D614-S698	0.99
341	Utah	PG6	14	RG1,RG4	7	S13-D138-T478-D614-T732-E1144-N1187	0.58
342	Utah	PG7	148	RG4,RG5	2	D614-P681	1.00
343	Utah	PG8	26	RG1,RG3,RG4	8	S13-D138-D253-T478-D614-T732-E1144-N1187	0.72
344	Utah	PG9	29	RG1,RG4	7	S13-D138-T478-D614-T732-E1144-N1187	0.58
345	Utah	PG10	6	RG2,RG4	3	T22-D614-S698	0.75
346	Utah	PG11	68	RG1,RG4	7	S13-D138-T478-D614-T732-E1144-N1187	0.58
347	Utah	PG14	1	RG4	1	D614	1.00
348	Utah	PG15	20	RG1,RG4,RG5	8	S13-D138-T478-D614-P681-T732-E1144-N1187	0.78
349	Utah	PG16	8	RG1,RG4	7	S13-D138-T478-D614-T732-E1144-N1187	0.58
350	Virginia	PG2	3196	RG3	1	D614	1.00
351	Virginia	PG3	240	RG3,RG5	2	D614-P681	1.00

352	Virginia	PG4	13	RG1,RG3	3	A263-D614-A701	0.75
353	Virginia	PG5	180	RG3,RG4	2	D614-Q677	1.00
354	Virginia	PG6	82	RG2,RG3	2	E484-D614	1.00
355	Virginia	PG7	78	RG1,RG3	3	A263-D614-A701	0.75
356	Virginia	PG8	53	RG2,RG3,RG5	3	E484-D614-P681	1.00
357	Virginia	PG9	1	RG2,RG3,RG4	3	E484-D614-Q677	1.00
358	Virginia	PG10	1	RG1,RG3,RG5	4	A263-D614-P681-A701	0.83
359	Virginia	PG11	1	RG3,RG4,RG5	3	D614-Q677-P681	1.00
360	Virginia	PG12	1	RG2,RG3,RG5	3	E484-D614-P681	1.00
361	Washington	PG2	7130	RG3	1	D614	1.00
362	Washington	PG4	12	RG3,RG4,RG5	3	D614-Q677-T732	1.00
363	Washington	PG5	128	RG3,RG5	2	D614-T732	1.00
364	Washington	PG7	208	RG3,RG4	2	D614-Q677	0.98
365	Washington	PG8	14	RG1,RG3	7	S13-W152-L452-V483-N501-D614-A684	0.61
366	Washington	PG9	74	RG2,RG3	2	D178-D614	1.00
367	Washington	PG10	10	RG1,RG3	7	S13-W152-L452-V483-N501-D614-A684	0.53
368	Washington	PG11	56	RG1,RG3	7	S13-W152-L452-V483-N501-D614-A684	0.58
369	Washington	PG12	24	RG1,RG3	7	S13-W152-L452-V483-N501-D614-A684	0.74
370	Wisconsin	PG1	6325	RG3	1	D614	1.00
371	Wisconsin	PG3	631	RG3,RG4	2	D614-Q677	1.00
372	Wisconsin	PG4	55	RG2,RG3	3	E583-D614-Q675	0.75
373	Wisconsin	PG5	82	RG2,RG3	3	E583-D614-Q675	0.97
374	Wisconsin	PG6	25	RG1,RG3	6	F157-N501-A575-D614-P681-M1229	0.60
375	Wisconsin	PG7	235	RG3,RG5	2	D614-E780	1.00
376	Wisconsin	PG8	115	RG1,RG3	6	F157-N501-A575-D614-P681-M1229	0.60
377	Wisconsin	PG9	56	RG1,RG3	6	F157-N501-A575-D614-P681-M1229	0.61
378	Wisconsin	PG10	91	RG1,RG3	6	F157-N501-A575-D614-P681-M1229	0.60
379	Wisconsin	PG11	10	RG1,RG3	6	F157-N501-A575-D614-P681-M1229	0.60
380	Wisconsin	PG12	3	RG2	2	E583-Q675	1.00
381	Wyoming	PG2	176	RG2	1	Q173	1.00
382	Wyoming	PG3	15	RG3	1	D215	1.00
383	Wyoming	PG4	74	RG5	1	P681	1.00
384	Wyoming	PG6	10	RG1	3	S13-W152-W258	1.00
385	Wyoming	PG7	4	RG1,RG4	4	S13-W152-W258-L452	0.50
386	Wyoming	PG8	5	RG1	3	S13-W152-W258	0.60
387	Wyoming	PG10	4	RG4	1	L452	1.00
388	Wyoming	PG11	9	RG1,RG4	4	S13-W152-W258-L452	0.70
389	Wyoming	PG12	37	RG1,RG4	4	S13-W152-W258-L452	1.00
390	Other	PG2	640	D614	1	D614	1.00

“Other” includes sequences from South Dakota, Vermont, and the Virgin Islands, in addition to sequences for which state information was missing.

Table S6. List of individuals from the Originating laboratories responsible for obtaining the specimens and the Submitting laboratories where genetic sequence data were generated and shared via the GISAID Initiative, on which this research is based.

We gratefully acknowledge the following Authors from the Originating laboratories responsible for obtaining the specimens, as well as the Submitting laboratories where the genome data were generated and shared via GISAID, on which this research is based.

All Submitters of data may be contacted directly via www.gisaid.org

Authors are sorted alphabetically.

Accession ID	Originating Laboratory	Submitting Laboratory	Authors
EPI_ISL_7543837, EPI_ISL_7543885, EPI_ISL_7544121, EPI_ISL_7544319, EPI_ISL_7544331, EPI_ISL_7544369, EPI_ISL_7544456, EPI_ISL_7544613, EPI_ISL_7544642, EPI_ISL_7544715			
see above	2 Military Hospital wc MAA	NHLS/UCT	Arash Iranzadeh; Bruna Galvao; Carolyn Williamson; Deelan Doolabh; Diana Hardie; Gert Marais; Innocent Mudau; Luicer Olubayo; Lynn Tyers; Marvin Hsiao; Nokuzola Mbhele; Rageema Joseph; Stephen Korsman
EPI_ISL_7621467, EPI_ISL_7621483, EPI_ISL_7621936	4Cyte Pathology	NSW Health Pathology - Institute of Clinical Pathology and Medical Research; Westmead Hospital; University of Sydney	Arnott A.; Draper J.; Gall M.; Martinez E.; Rockett R.; Sintchenko V.; on behalf of ICPMR
EPI_ISL_7495248, EPI_ISL_7495249, EPI_ISL_7495250	A. Krumbholz, Labor Dr. Krause und Kollegen MVZ GmbH, Kiel	Charité Universitätsmedizin Berlin, Institut für Virologie	Barbara Mühlemann; Christian Drosten; Julia Schneider; Julia Tesch; Jörn Beheim-Schwarzbach; Talitha Veith; Terry Jones; Tobias Bleicker; Victor M Corman
EPI_ISL_7405721	A.S.L. CITTA DI TORINO - OSPEDALE AMEDEO DI SAVOIA	Fondazione del Piemonte per l'Oncologia IRCCS	Antonino Sottile; Giorgio Giardina; Paola Marino; Silvia Brossa
EPI_ISL_7306737	A.S.L. TO4	Fondazione del Piemonte per l'Oncologia IRCCS	Antonino Sottile; Giorgio Giardina; Paola Marino; Silvia Brossa
EPI_ISL_7016910, EPI_ISL_7569944, EPI_ISL_7569960, EPI_ISL_7569961, EPI_ISL_7569968, EPI_ISL_7569970, EPI_ISL_7569978, EPI_ISL_7569980, EPI_ISL_7569985, EPI_ISL_7569988			
see above	ACT Pathology	Schwessinger Lab	Ashley Jones; Austin Bird; Bayantes Dagvadorj; Benjamin Schwessinger; Carl McCombe; Carolina Correa Ospina; Catalina Barragán Quintero; Craig Kennedy; Elise Kellett; Emma Crean; Evie Hodgson; Gabrielle Smith; Karina Kennedy; Rachel Leonard; Rene Riedelbauch; Robyn Hall; Salome Wilson; Scott Ferguson
EPI_ISL_6891760, EPI_ISL_7129657, EPI_ISL_7129884, EPI_ISL_7130085, EPI_ISL_7130189, EPI_ISL_7130344, EPI_ISL_7130486			
see above	AGES-Institute for medical Microbiology and Hygiene Vienna	AGES-Institute for medical Microbiology and Hygiene Vienna	Alexander Indra; Elisabeth Polster; Florian Heger; Julia Klikovits; Kathrin Lippert; Marion Blaschitz; Patrick Hyden; Peter Hufnagl; Stefanie Dobrovolny; Vera Wallner
EPI_ISL_7381191	AHRI	CERI, Centre for Epidemic Response and Innvoation, Stellenbosch University and KRISP, KZN Research Innovation and Sequencing Platform, UKZN.	Arisha Maharaj; Bernstein Mallory; Cele Sandile; Giandhari J; Karim Farina; Khan Khadija; Moir M; Naidoo Y; Pillay S; Ramphal U; Ramphal Y; San JE; Sigal Alex; Tegally H; Tshiabuila D; Wilkinson E; de Oliveira T; van Wyk S
EPI_ISL_7358094	AHRI-Sigal	CERI, Centre for Epidemic Response and Innvoation, Stellenbosch University and KRISP, KZN Research Innovation and Sequencing Platform, UKZN.	Bernstein Mallory; Cele Sandile; Giandhari J; Karim Farina; Khan Khadija; Moir M; Naidoo Y; Nokukhanya Mdlalose; Pillay S; Ramphal U; Ramphal Y; San JE; Sigal Alex; Tegally H; Tshiabuila D; Wilkinson E; de Oliveira T
EPI_ISL_7636072	AZDelta	AZ Delta Medical Laboratories in Roeselare, Belgium	Dieter De Smet; Frederik Van Hoecke; Geert Martens; on behalf of AZ Delta COVID-19 Genomics core (member of Genomic surveillance of SARS-CoV-2 in Belgium network)
EPI_ISL_7154405	Aegis Sciences Corporation	Centers for Disease Control and Prevention Division of Viral Diseases, Pathogen Discovery	Alec Vest; Benjamin Rambo-Martin; Christopher Gulvick; Clinton Paden; Cyndi Clark; Dakota Howard; Dhwani Batra; Dillon Nall; Duncan MacCannell; Erisa Sula; Ethan Sanders; Holly Houdeshell; Jason Caravas; Kristine Lacek; Matthew Hardison; Matthew Schmerer; Ola Kvalvaag; Patrick Campbell; Peter Cook; Rob Case; Scott Sammons; Shatavia Morrison; Shaun Westlund; Tymeckia Kendall; Victoria Caban Figueroa; Vikramsinha Ghorpade; Yvette Unoarumhi
EPI_ISL_7452769, EPI_ISL_7452775	Aesculabor Hamburg, Institut der Labormedizin	Heinrich Pette Institute, Leibniz Institute for Experimental Virology	Adam Grundhoff; Alexis Robitaille; Johannes Knobloch; Martin Aepfelbacher; Nicole Fischer; Thomas Günther
EPI_ISL_7470217, EPI_ISL_7470278, EPI_ISL_7470360, EPI_ISL_7470368, EPI_ISL_7470391, EPI_ISL_7470446	Akershus University Hospital, Department for Microbiology and Infectious Disease Control	Norwegian Institute of Public Health, Department of Virology	Atiya R Ali; Debech Nadia; Engebretsen Serina Beate; Garcia Llorente Ignacio; Hilde Elshaug; Hilde Vollan; Jon Bråte; Kamilla Heddeland Instefjord; Karoline Bragstad; Kathrine Stene-Johansen; Line Victoria Moen; Marie Paulsen Madsen; Olav Hungnes; Pedersen Benedikte Nevjen; Rasmus Riis Kopperud
EPI_ISL_7620089	Alaska State Virology Laboratory	Alaska State Virology Laboratory	Elva House; Jack Chen; Jacob Zidek; Jeremy Roe; Lisa Smith; Ph.D.
EPI_ISL_7146436, EPI_ISL_7506705	Allergy, Immunology and Cell Biology Unit (AICBU)	Allergy, Immunology and Cell Biology Unit (AICBU)	Ayesha Wijesinghe; Chandima Jeewandara; Deshni Jayathilaka; Dinuka Ariyaratne; Diyanath Ranasinghe; Dumni Guasinghe; Farha Bary; Gathsaurie Neelika Malavige; Tibutius Thanesh
EPI_ISL_7544441	Alma CDC wc AHC	NHLS/UCT	Arash Iranzadeh; Bruna Galvao; Carolyn Williamson; Deelan Doolabh; Diana Hardie; Gert Marais; Innocent Mudau; Luicer Olubayo; Lynn Tyers; Marvin Hsiao; Nokuzola Mbhele; Rageema Joseph; Stephen Korsman
EPI_ISL_7264139, EPI_ISL_7265455, EPI_ISL_7265456	Alpha Labs	National Microbiology Laboratory (NML)	Anna Majer; Anneliese Landgraff; CanCOGeN's metadata curation team; Darian Hole; Dynacare Brampton COVID-19 Diagnostic team; Elsie Grudeski; Gary Van Domselaar; Gordon Jolly; Grace Seo; Jennifer Tanner; Madison Chapel; Morag Graham; Natalie Knox; Nathalie Bastien; Philip Mabon; Public Health Agency of Canada CanCOGeN team; Rhiannon Huzarewich; Russell Mandes; Shari Tyson; Timothy Booth; Yan Li
EPI_ISL_6914011, EPI_ISL_6914012, EPI_ISL_6914013, EPI_ISL_6914014, EPI_ISL_6914015, EPI_ISL_6914016, EPI_ISL_6914017, EPI_ISL_6914018, EPI_ISL_6914019, EPI_ISL_6914020, EPI_ISL_6914021, EPI_ISL_6914022, EPI_ISL_6914023, EPI_ISL_6914024, EPI_ISL_6914025, EPI_ISL_6914026, EPI_ISL_6914027, EPI_ISL_6914028, EPI_ISL_6914029, EPI_ISL_6914030, EPI_ISL_6914031, EPI_ISL_6914032, EPI_ISL_6914033, EPI_ISL_6914034, EPI_ISL_6914035, EPI_ISL_6914036			
see above	Ampath Laboratories	National Institute for Communicable Diseases of the National Health Laboratory Service	Amoako DG; Bhiman JN; Everatt J; Ismail A; Mahlangu B; Mnguni A; Mohale T; Ntuli N; Scheepers C; Wolter N
EPI_ISL_7605587, EPI_ISL_7605639, EPI_ISL_7605640, EPI_ISL_7605743, EPI_ISL_7605745	Anglo American	National Institute for Communicable Diseases of the National Health Laboratory Service	Amoako DG; Bhiman JN; Everatt J; Ismail A; Mahlangu B; Mnguni A; Mohale T; Ntuli N; Scheepers C; Wolter N
EPI_ISL_7011321, EPI_ISL_7568602	Area of Virology, Serology and Virology Division (SAViD), New South Wales Health Pathology Randwick	Virology Research Laboratory; Area of Virology, Serology and Virology Division (SAViD), New South Wales Health Pathology Randwick	Au, J.; Bull, R.; Deveson, I.; Foster, C.; Rawlinson, W.; Ruiz Silva, M.; Van Hal, S.
EPI_ISL_7503376, EPI_ISL_7503377, EPI_ISL_7503378, EPI_ISL_7548950, EPI_ISL_7548951, EPI_ISL_7548952	Arizona State University	Arizona State University	Efrem S. Lim; Joshua LaBaer; LaRinda A. Holland; Matthew F. Smith; Nathaniel Johnson; Regan A. Sullins; Steven C. Holland; Vel Murugan
EPI_ISL_6963510	Auriga Research Pvt.Ltd / Strand Life Sciences	National Centre for Biological Sciences, TIFR - Rockefeller Foundation	Aarati Karaba; Anson Kunjumon George; Aparnaa Ramanathan; Apurva Sarin; Chandrasekhar Vadlamudi; Chitra Pattabiraman; Darshan Sreenivas; Dasaradhi Palakodeti; Dimple Notani; Divya Priya A; Madhusudhan J; Manisha Bharadwaj; Manoj Kumar Jha; Mudasir Nazaar; Pradeep B P; Priyanka Ananta Mulay; Ramesh Hariharan; Rohan Pais; Satyajit Mayor; Saumitra Mardikar; Srivathsan Adimoolam; Uma Ramakrishnan; Vamsi Veeramachaneni; Vasanthapuram Ravi; Vijay Chandru; Vishal G Rao; Yasodha Kannan
EPI_ISL_7621950, EPI_ISL_7621951, EPI_ISL_7621953, EPI_ISL_7621957	Australian Clinical Labs (formerly Healthscope Pathology)	NSW Health Pathology - Institute of Clinical Pathology and Medical Research; Westmead Hospital; University of Sydney	Arnott A.; Draper J.; Gall M.; Martinez E.; Rockett R.; Sintchenko V.; on behalf of ICPMR
EPI_ISL_7195246, EPI_ISL_7195247, EPI_ISL_7195248	Austrian Agency for Health and Food Safety (AGES)	Bergthaler laboratory, CeMM Research Center for Molecular Medicine of the Austrian Academy of Sciences	Andreas Bergthaler; Anna Schedl; Bekir Erguner; Benedikt Agerer; Christoph Bock; Fabian Amman; Jan Laine; Lukas Endler; Martin Senekowitsch; Matthew Thornton; Michael Schuster; Michelle Chan; Petr Triska; Thomas Penz
EPI_ISL_7502111, EPI_ISL_7616000	Ayass Bioscience LLC	Ayass Bioscience LLC	Kevin Zhu; Lina Abi Mosleh; Mohamad Ammar Ayass; Natalya Griko; Nazanin Taheri
EPI_ISL_7400565	Azienda Ospedaliera Pugliese Ciaccio di Catanzaro SOC Microbiologia e Virologia	Azienda Ospedaliera Pugliese Ciaccio di Catanzaro SOC Microbiologia e Virologia	Rossana Tallerico Cinzia Peronace Federica Pasceri Marco De Fazio Ilenia Talotta Giuseppina Panduri Pasquale Minchella
EPI_ISL_7338921	Azienda Sanitaria dell´Alto Adige - Laboratorio Aziendale di Microbiologia e Virologia	Azienda Sanitaria dell'Alto Adige	Irene Bianconi
EPI_ISL_7226262	BNH Hospital	National Institute of Health,	Archawin Rojanawiwat; Ballang Uppapong; Beth Skaggs; Donlaya Maunplueng; Kazuhisa Okada; Nuttida Thongpramul; Pakorn Piromtong; Pilailuk Akkapaiboon Okada; Piroon Jenjaroenpun; Pongpun Sawatwong; Prapat Suriyaphol; Sirikanda Wimol; Siripaporn Phuygun; Sittiporn Parnmen; Supakit

		Department of Medical Sciences, Ministry of Public Health, Thailand	Sirilak; Suratchana Mitrat; Thanutsapa Thanadachakul; Thidathip Wongsurawat
EPI_ISL_7612892	BUMC Transplant Immunology Lab	BUMC Transplant Immunology Lab	Amanda Willis; Chiensao Wang; Jenifer Williams; Lynne Klingman; Medhat Askar; Pete Dysert
EPI_ISL_7565149, EPI_ISL_7565152	Bari	University of Bari Biomedical Sciences and Human Oncology	Maria Chironna
EPI_ISL_7149647, EPI_ISL_7197950, EPI_ISL_7346925, EPI_ISL_7347257, EPI_ISL_7347522, EPI_ISL_7355779, EPI_ISL_7391885, EPI_ISL_7391908, EPI_ISL_7391931, EPI_ISL_7391984, EPI_ISL_7392005, EPI_ISL_7392027, EPI_ISL_7392079, EPI_ISL_7392087, EPI_ISL_7392093, EPI_ISL_7392139, EPI_ISL_7485998, EPI_ISL_7486106, EPI_ISL_7486193, EPI_ISL_7486452, EPI_ISL_7486697, EPI_ISL_7486742, EPI_ISL_7486808, EPI_ISL_7486814, EPI_ISL_7486850, EPI_ISL_7486923, EPI_ISL_7487524, EPI_ISL_7487623, EPI_ISL_7487665, EPI_ISL_7487692, EPI_ISL_7512352, EPI_ISL_7512373, EPI_ISL_7512378, EPI_ISL_7512381, EPI_ISL_7512410, EPI_ISL_7512438, EPI_ISL_7512444, EPI_ISL_7512448, EPI_ISL_7520500, EPI_ISL_7538419, EPI_ISL_7538437, EPI_ISL_7538529, EPI_ISL_7538539, EPI_ISL_7538562, EPI_ISL_7538591, EPI_ISL_7538628, EPI_ISL_7538662, EPI_ISL_7538687, EPI_ISL_7538812, EPI_ISL_7538904, EPI_ISL_7538983, EPI_ISL_7538987, EPI_ISL_7539099, EPI_ISL_7539114, EPI_ISL_7539140, EPI_ISL_7539157, EPI_ISL_7539216, EPI_ISL_7539258, EPI_ISL_7539330, EPI_ISL_7539539, EPI_ISL_7539677, EPI_ISL_7539761			
see above	Berkshire and Surrey Pathology Services Lighthouse Laboratory	Wellcome Sanger Institute for the COVID-19 Genomics UK (COG-UK) Consortium	Berkshire and Surrey Pathology Services Lighthouse Laboratory and Alex Alderton; Cordelia Langford; David K. Jackson; Dominic Kwiatkowski; Ewan Harrison; Ian Johnston; Jeffrey Barrett; John Sillitoe on behalf of the Wellcome Sanger Institute COVID-19 Surveillance Team; Roberto Amato; Sonia Goncalves
EPI_ISL_7196120, EPI_ISL_7196121	Bidh Lab	National Public Health Laboratory	Bidh Laboartory; National Public Health Laboratory Team
EPI_ISL_7464464, EPI_ISL_7464465	Biolab Diagnostic Laboratories	Biolab Diagnostic Laboratories	Ahmad Tibi; Amid Abdelnour; Badia Saddedin; Eiad Atwa; Issa Abu-Dayyeh; Lama Hussein; Shaima Ali
EPI_ISL_7042354	Biolytix AG	Clinical Bacteriology, University Hospital Basel	Adrian Egli; Alfredo Mari; Fanny Wegner; Hans Hirsch; Helena MB Seth-Smith; Julia Bielicki; Karoline Leuzinger; Manuel Battegay; Tim Roloff
EPI_ISL_7417514	Bioscientia Labor Wermsdorf	Robert Koch Institute	
EPI_ISL_7544135, EPI_ISL_7544479, EPI_ISL_7544573	Bothasig CDC wc BLD	NHLS/UCT	Arash Iranzadeh; Bruna Galvao; Carolyn Williamson; Deelan Doolabh; Diana Hardie; Gert Marais; Innocent Mudau; Luicer Olubayo; Lynn Tyers; Marvin Hsiao; Nokuzola Mbhele; Rageema Joseph; Stephen Korsman
EPI_ISL_7548902, EPI_ISL_7548903, EPI_ISL_7548904, EPI_ISL_7548905, EPI_ISL_7548906, EPI_ISL_7548909, EPI_ISL_7548914, EPI_ISL_7548917, EPI_ISL_7548931, EPI_ISL_7552698, EPI_ISL_7552699, EPI_ISL_7566328, EPI_ISL_7566330, EPI_ISL_7566360			
see above	Botswana Harvard HIV Reference Laboratory	Botswana Harvard AIDS Institute Partnership, Plot 1836 North Ring Road, Princess Marina Hospital, Gaborone	Boitumelo Zuze; Botshelo Radibe; Dorcas Maruapula; Doreen Ditshwanelo; Joseph Makhema; Keoratile Ntshambiwa; Kgomotso Moruisi; Legodile Kooepile; Mosepele Mosepele; Mphaphi B. Mbulawa; Ontlametse T. Bareng; Pamela Smith-Lawrence; Roger Shapiro; Sefetogi Ramaologa; Shahin Lockman; Sikhulile Moyo; Simani Gaseitsiwe; Thongbotho Mphoyakgosi; Wonderful T. Choga
EPI_ISL_6640916, EPI_ISL_6640917, EPI_ISL_6640919, EPI_ISL_6670244, EPI_ISL_6752026, EPI_ISL_6752027, EPI_ISL_6774081, EPI_ISL_6774083, EPI_ISL_6774084, EPI_ISL_6774085, EPI_ISL_6774087, EPI_ISL_6774088, EPI_ISL_6774089, EPI_ISL_6774090, EPI_ISL_6774091, EPI_ISL_6774093, EPI_ISL_7121195, EPI_ISL_7121204, EPI_ISL_7121205, EPI_ISL_7380512, EPI_ISL_7380515, EPI_ISL_7380524			
see above	Botswana Harvard HIV Reference Laboratory	Botswana Harvard HIV Reference Laboratory	Boitumelo Zuze; Botshelo Radibe; Dorcas Maruapula; Joseph Makhema; Keoratile Ntshambiwa; Kgomotso Moruisi; Legodile Kooepile; Mosepele Mosepele; Mphaphi B. Mbulawa; Ontlametse T. Bareng; Pamela Smith-Lawrence; Roger Shapiro; Sefetogi Ramaologa; Shahin Lockman; Sikhulile Moyo; Simani Gaseitsiwe; Thongbotho Mphoyakgosi; Wonderful T. Choga
EPI_ISL_7132804, EPI_ISL_7370157, EPI_ISL_7370181, EPI_ISL_7370259, EPI_ISL_7370472	British Columbia Centre For Disease Control	BCCDC Public Health Laboratory	655 W 12th Avenue; Ana Pacagnella; BC Canada V5T 2N3; Corrinne Ng; Dan Fornika; John Tyson; Kim Macdonald; Kimia Kamelian; Linda Hoang; Loretta Janz; Mel Krajden; Prystajecky Natalie; Robert Azana; Shannon Russell; Vancouver
EPI_ISL_7390696, EPI_ISL_7391208, EPI_ISL_7453171, EPI_ISL_7453991, EPI_ISL_7454277, EPI_ISL_7542815, EPI_ISL_7543162, EPI_ISL_7543543, EPI_ISL_7543579, EPI_ISL_7544427, EPI_ISL_7544520, EPI_ISL_7547858, EPI_ISL_7548157, EPI_ISL_7550313, EPI_ISL_7550474, EPI_ISL_7550515, EPI_ISL_7550529, EPI_ISL_7550748			
see above	Broad Institute Clinical Research Sequencing Platform	Infectious Disease Program, Broad Institute of Harvard and MIT	Adams, G.; B.L.; B.W.; Bauer, M.; Birren; Blumenstiel, B.; Brown, C.; Carter, A.; Chaluvadi, S.; D.J.; DeFelice, M.; DeRuff, K.; Dodge, S.; Gabriel, S.; Gallagher, G.; Gladden-Young, A.; Granger, B.; J.E.; K.J.; Lagerborg, K.; Larkin, K.; Lee, M.; Lemieux; Lennon, N.; Loreth, C.; Madoff, L.; McGovern, S.; Meldrim, J.; Normandin, E.; P.C.; Park; Pearlman, L.; Reilly, S.; Rudy, M.; Sabeti; Siddle; Smole, S.; Tomkins-Tinch, C.; Vicente, G.; and MacInnis
EPI_ISL_7263830	CAP Roger de Flor	Banc de Sang i Teixits	Carlos Hobeich; Francisco Vidal; Irene Corrales; Lorena Ramírez; Maria Glòria Soria; Natália Comes; Nina Borràs; Noemí Gonzalez; Silvia Sauleda
EPI_ISL_7477252	CDPH VBL	California Department of Public Health	Emily Smith on behalf of CDPH-COVIDNet
EPI_ISL_7427856	CENTOGENE Frankfurt Laboratory: Niederlassung Industriepark Höchst	Robert Koch Institute	
EPI_ISL_7547731	CENTOLAB	National Reference Laboratory, Nigeria Centre for Disease Control	Catherine Okoi; Chimaobi Chukwu; Dr Ifedayo Adetifa; Dr Ndodo Nnaemeka; Dr Omoare Adesuyi; Nwando Mba; Olajumoke Babatunde; Olusola Anuoluwapo Akanbi; Oyeronke Ayansola
EPI_ISL_6862897	CERBALLIANCE	UMR PIMIT	David A Wilkinson; Patrick Mavingui
EPI_ISL_6962948, EPI_ISL_6967758	CERBALLIANCE PARIS ET IDF EST	CERBA HealthCare	Bénédicte Roquebert; Johanna Roux; Judith Zerah; Laura Verdurme; Sabine Trombert; Stéphanie Haim-Boukobza
EPI_ISL_7226961	CHU de Bordeaux	CNR Virus des Infections Respiratoires - France SUD	Antonin Bal; Bruno Lina; Bruno Simon; Denis Malvy; Gregory Destras; Gwendolyne Burfin; Hadrien Regue; Laurence Josset; Marie-Edith Lafon; Martine Valette; Pantxika Bellecave; Quentin Semanas
EPI_ISL_7268670, EPI_ISL_7269933	CLINA-LANCET LABORATORIES	National Reference Laboratory, Nigeria Centre for Disease Control	Catherine Okoi; Chimaobi Chukwu; Dr Ifedayo Adetifa; Dr Ndodo Nnaemeka; Dr Omoare Adesuyi; Nwando Mba; Olajumoke Babatunde; Olusola Anuoluwapo Akanbi; Oyeronke Ayansola
EPI_ISL_7313633	CT Department of Public Health	CT Department of Public Health	Claire Pearson; Tu N. Nguyen
EPI_ISL_7137326, EPI_ISL_7137327, EPI_ISL_7137328, EPI_ISL_7137330	California Department of Public Health	California Department of Public Health	CDPH IDLB COVIDNet
EPI_ISL_7217437, EPI_ISL_7217563	Cantacuzino National Military-Medical Institute, Viral Respiratory Infections Laboratory	Cantacuzino Institute Virology	Luiza Ustea; Mihaela Lazar; Mihaela Oprea; Nicoleta Paraschiv; Sorin Dinu
EPI_ISL_7571582	Centre Hospitalier Universitaire Clermont-Ferrand	CHU Clermont-Ferrand, service de virologie	Bisseux Maxime; Combes Patricia; Henquell Cecile; Mirand Audrey
EPI_ISL_6980876	Cerballiance, Reunion	UMR PIMIT	David A Wilkinson; Patrick Mavingui
EPI_ISL_7019047	Charité Universitätsmedizin Berlin, Institute of Virology	Charité Universitätsmedizin Berlin, Institute of Virology	Barbara Mühlemann; Christian Drosten; Julia Schneider; Julia Tesch; Jörn Beheim-Schwarzbach; Talitha Veith; Terry Jones; Tobias Bleicker; Victor M Corman
EPI_ISL_7337463, EPI_ISL_7337464, EPI_ISL_7337465, EPI_ISL_7337466, EPI_ISL_7337468, EPI_ISL_7337469, EPI_ISL_7337470, EPI_ISL_7337471, EPI_ISL_7337472, EPI_ISL_7337473, EPI_ISL_7337474, EPI_ISL_7337475, EPI_ISL_7337476, EPI_ISL_7337477, EPI_ISL_7337478, EPI_ISL_7337479, EPI_ISL_7337480, EPI_ISL_7337481, EPI_ISL_7337482, EPI_ISL_7337483, EPI_ISL_7337484, EPI_ISL_7337485, EPI_ISL_7337486, EPI_ISL_7337487, EPI_ISL_7337488, EPI_ISL_7337489, EPI_ISL_7337490, EPI_ISL_7337495, EPI_ISL_7337496, EPI_ISL_7337497, EPI_ISL_7337498, EPI_ISL_7337499, EPI_ISL_7337500, EPI_ISL_7337501, EPI_ISL_7337502, EPI_ISL_7337503, EPI_ISL_7337504, EPI_ISL_7337505, EPI_ISL_7337506, EPI_ISL_7337507, EPI_ISL_7337508, EPI_ISL_7337509, EPI_ISL_7337510, EPI_ISL_7337511			
see above	Charlotte Maxeke Johannesburg Academic Hospital	National Institute for Communicable Diseases of the National Health Laboratory Service	Amoako DG; Bhiman JN; Everatt J; Ismail A; Mahlangu B; Mnguni A; Mohale T; Ntuli N; Scheepers C
EPI_ISL_7337512, EPI_ISL_7337513, EPI_ISL_7337515, EPI_ISL_7337516, EPI_ISL_7337517, EPI_ISL_7337518, EPI_ISL_7337520, EPI_ISL_7337521, EPI_ISL_7337524, EPI_ISL_7337525, EPI_ISL_7337527, EPI_ISL_7337528, EPI_ISL_7337531, EPI_ISL_7337532, EPI_ISL_7337533, EPI_ISL_7337534, EPI_ISL_7337535, EPI_ISL_7337536, EPI_ISL_7337537, EPI_ISL_7337538, EPI_ISL_7337539, EPI_ISL_7337540, EPI_ISL_7337541, EPI_ISL_7337542, EPI_ISL_7337543, EPI_ISL_7337544, EPI_ISL_7337545, EPI_ISL_7337546, EPI_ISL_7337547, EPI_ISL_7337548, EPI_ISL_7337549, EPI_ISL_7337550, EPI_ISL_7337551, EPI_ISL_7337552, EPI_ISL_7337553, EPI_ISL_7337558, EPI_ISL_7337559, EPI_ISL_7337560, EPI_ISL_7337561, EPI_ISL_7337562, EPI_ISL_7337563, EPI_ISL_7337564			
see above	Chris Hani Baragwanath Laboratory	National Institute for Communicable Diseases of the National Health Laboratory Service	Amoako DG; Bhiman JN; Everatt J; Ismail A; Mahlangu B; Mnguni A; Mohale T; Ntuli N; Scheepers C
EPI_ISL_7154390	Clina-Lancet	National Reference Laboratory, Nigeria Centre for Disease Control	Catherine Okoi; Chimaobi Chukwu; Dr Ifedayo Adetifa; Dr Ndodo Nnaemeka; Dr Omoare Adesuyi; Nwando Mba; Olajumoke Babatunde; Olusola Anuoluwapo Akanbi; Oyeronke Ayansola
EPI_ISL_7265967, EPI_ISL_7265976, EPI_ISL_7265979, EPI_ISL_7266027, EPI_ISL_7266045, EPI_ISL_7266056, EPI_ISL_7266083, EPI_ISL_7266160, EPI_ISL_7552013, EPI_ISL_7552178, EPI_ISL_7552184, EPI_ISL_7552302, EPI_ISL_7552311, EPI_ISL_7552313, EPI_ISL_7552316, EPI_ISL_7552499, EPI_ISL_7552502, EPI_ISL_7552514, EPI_ISL_7552588, EPI_ISL_7552623, EPI_ISL_7552635, EPI_ISL_7552636			
see above	Clinical Microbiology Laboratory, Tel Aviv Sourasky Medical Center	Clinical Microbiology Laboratory, Tel Aviv Sourasky Medical Center	Alon Ziv; Amos Adler; Katya Levytskyi; Lior Handler; Ora Halutz
EPI_ISL_7042161, EPI_ISL_7042168, EPI_ISL_7462220, EPI_ISL_7462233, EPI_ISL_7462310, EPI_ISL_7462311, EPI_ISL_7462312			
see above	Clinical Virology	Clinical Bacteriology, University Hospital Basel	Adrian Egli; Alfredo Mari; Fanny Wegner; Hans Hirsch; Helena MB Seth-Smith; Julia Bielicki; Karoline Leuzinger; Manuel Battegay; Tim Roloff
EPI_ISL_7373598	Clinical Virology, Children's Hospital Los Angeles	Clinical Virology, Children's Hospital Los Angeles	Alexander Judkins; Cheryl Pool; Javier Mestas; Jennifer Dien Bard; John Fissel; Maurice O'Gorman
EPI_ISL_7462438	Cliniques universitaires Saint-Luc	UCLouvain/IREC/MBLG-CTMA	Benoit Kabamba Mukadi; Bertrand Bearzatto; Jean-Luc Gala; Nicolas Pinte; Paul Blanpain; Simon Ophélie; Valentin Coste
EPI_ISL_6951145	Color Genomics	Chiu Laboratory, University of California, San Francisco	Alicia Sotomayor-Gonzalez; Alicia Zhou; Amy Garlin; Charles Chiu; Darpun Sachdev; Katherine Hernandez; Scott Topper; Susan Philip; Venice Servellita; Yueyuan Zhang
EPI_ISL_7010485	Colorado Department of Public Health and Environment	Colorado Department of Public Health and Environment	Alexandria Rossheim; Diana Ir; Emily A. Travanty; Laura Bankers; Mandy Waters; Michael A. Martin; Molly C. Hetherington-Rauth; Sarah Elizabeth Totten; Shannon R. Matzinger
EPI_ISL_7613413	Community Labs, San Antonio	STRL UT Health San Antonio, Greehey Children's Cancer Research Institute	Bethany Landry; Dawn Garcia; Guillermo Nunez; Hongxin Fan; Josefina Stoever; Korri Weldon; Kumari Vadlamudi; Marjorie Parker David; San Antonio Metropolitan Health District; Texas Department of State Health Services; Weijing He; Yidong Chen; Zhao Lai; Zhenqing Ye

EPI_ISL_7451063, EPI_ISL_7451073, EPI_ISL_7451079, EPI_ISL_7451089	Cruz Vermelha Portuguesa	Instituto Nacional de Saude (INSA)	Borges et al
EPI_ISL_7609869	Curative Labs	Curative Labs	Elias L. Salfati; Eugenia Khorosheva; George Way; J.Cesar Ignacio-Espinoza; Janet Chen; Mikhail Hanewich-Hollatz; Nabjot Sandhu; Sophia Quasem; Vladimir Slepnev; Zhiyi Xie
EPI_ISL_7605689, EPI_ISL_7605739, EPI_ISL_7605775	DE AAR LABORATORY	National Institute for Communicable Diseases of the National Health Laboratory Service	Amoako DG; Bhiman JN; Everatt J; Ismail A; Mahlangu B; Mnguni A; Mohale T; Ntuli N; Scheepers C; Wolter N
EPI_ISL_7062087, EPI_ISL_7063588, EPI_ISL_7272249, EPI_ISL_7514034, EPI_ISL_7514991, EPI_ISL_7515043, EPI_ISL_7515374, EPI_ISL_7516043, EPI_ISL_7517471, EPI_ISL_7517646, EPI_ISL_7518103, EPI_ISL_7518795, EPI_ISL_7520214, EPI_ISL_7520543, EPI_ISL_7520819, EPI_ISL_7523657, EPI_ISL_7525859, EPI_ISL_7526218, EPI_ISL_7528707, EPI_ISL_7529040, EPI_ISL_7529817, EPI_ISL_7530609, EPI_ISL_7532336, EPI_ISL_7532391, EPI_ISL_7533005, EPI_ISL_7534407, EPI_ISL_7534541, EPI_ISL_7596661, EPI_ISL_7596668, EPI_ISL_7596669, EPI_ISL_7596670, EPI_ISL_7596683, EPI_ISL_7596684, EPI_ISL_7596696, EPI_ISL_7596697, EPI_ISL_7596702, EPI_ISL_7599427, EPI_ISL_7599604, EPI_ISL_7599994, EPI_ISL_7600175, EPI_ISL_7600348, EPI_ISL_7600349, EPI_ISL_7600356, EPI_ISL_7600365, EPI_ISL_7600506, EPI_ISL_7600699, EPI_ISL_7600710, EPI_ISL_7600940, EPI_ISL_7600946, EPI_ISL_7601285, EPI_ISL_7601634, EPI_ISL_7601635, EPI_ISL_7601645, EPI_ISL_7601647, EPI_ISL_7601691, EPI_ISL_7602008, EPI_ISL_7602017, EPI_ISL_7602018, EPI_ISL_7602026, EPI_ISL_7602027, EPI_ISL_7602028, EPI_ISL_7602031, EPI_ISL_7602182, EPI_ISL_7602236, EPI_ISL_7602366, EPI_ISL_7602373, EPI_ISL_7602581, EPI_ISL_7602582, EPI_ISL_7602583, EPI_ISL_7602584, EPI_ISL_7602585, EPI_ISL_7602591, EPI_ISL_7602592, EPI_ISL_7602593, EPI_ISL_7602594, EPI_ISL_7602601, EPI_ISL_7602607, EPI_ISL_7602608, EPI_ISL_7602609, EPI_ISL_7602610, EPI_ISL_7602617, EPI_ISL_7602618, EPI_ISL_7602619, EPI_ISL_7602620, EPI_ISL_7602625, EPI_ISL_7602626, EPI_ISL_7602627, EPI_ISL_7602628, EPI_ISL_7602636, EPI_ISL_7602637, EPI_ISL_7602638, EPI_ISL_7602644, EPI_ISL_7602645, EPI_ISL_7602646, EPI_ISL_7602647, EPI_ISL_7602655, EPI_ISL_7602656, EPI_ISL_7602657, EPI_ISL_7602658, EPI_ISL_7602664, EPI_ISL_7602665, EPI_ISL_7602666, EPI_ISL_7602667, EPI_ISL_7602673, EPI_ISL_7602674, EPI_ISL_7602675, EPI_ISL_7602676, EPI_ISL_7602685, EPI_ISL_7602686, EPI_ISL_7638530, EPI_ISL_7638596, EPI_ISL_7638689, EPI_ISL_7638718, EPI_ISL_7639039, EPI_ISL_7639117, EPI_ISL_7639395, EPI_ISL_7639484, EPI_ISL_7640293, EPI_ISL_7640569, EPI_ISL_7640706, EPI_ISL_7640724, EPI_ISL_7640969, EPI_ISL_7641033, EPI_ISL_7642298, EPI_ISL_7642471, EPI_ISL_7642555, EPI_ISL_7642579, EPI_ISL_7642615, EPI_ISL_7642958, EPI_ISL_7642986, EPI_ISL_7643084, EPI_ISL_7643799, EPI_ISL_7644130, EPI_ISL_7644179, EPI_ISL_7644186			
see above	Department of Bacteria, Parasites and Fungi, Statens Serum Institut, Copenhagen, Denmark	Statens Serum Institut Bioinformatics and Microbial Genomics	Danish Covid-19 Genome Consortium
EPI_ISL_7042669	Department of Clinical Microbiology	GIGA Medical Genomics	Bouchra Boujemla; Claire Gourzonès; Cécile Meex; Keith Durkin; Laurent Gillet; Maria Artesi; Marie-Pierre Hayette; Nadine Cambisano; Nathalie Renotte; Olivier Ek; Sébastien Bontems; Vincent Bours
EPI_ISL_7512899, EPI_ISL_7520649, EPI_ISL_7531709, EPI_ISL_7533537, EPI_ISL_7533749	Department of Clinical Microbiology, Odense University Hospital, Odense, Denmark	Statens Serum Institut Bioinformatics and Microbial Genomics	Danish Covid-19 Genome Consortium
EPI_ISL_7192723, EPI_ISL_7192733, EPI_ISL_7192734, EPI_ISL_7485700	Department of Health Technology and Informatics, The Hong Kong Polytechnic University	Department of Health Technology and Informatics, The Hong Kong Polytechnic University	Alan Ka-Lun Wu; Alex Yat-Man Ho; Barry Kin-Chung Wong; Chloe Toi-Mei Chan; David Ho-Keung Shum; Denise Sze-Hang Wong; Gilman Kit-Hang Siu; Hiu-Yin Lao; Hoi-Ching Jim; Ivan Tak-Fai Wong; Jake Siu-Lun Leung; Kam-Tong Yip; Kenneth Siu-Sing Leung; Kingsley King-Gee Tam; Kitty Sau-Chun Fung; Kristine Luk; Lam-Kwong Lee; Miranda Chong-Yee Yau; Sandy Ka-Yee Chau; Shea Ping Yip; Tak-Lun Que; Timothy Ting-Leung Ng; Wing Cheong Yam; Wing-Hei Lo; Wing-Kin To; Yvette Wai-Man Lai
EPI_ISL_6841980, EPI_ISL_6841981, EPI_ISL_7138045, EPI_ISL_7357684, EPI_ISL_7385702	Department of Microbiology, The University of Hong Kong	Department of Microbiology, The University of Hong Kong	Kelvin K.W. To; Kwok-Yung Yuen
EPI_ISL_7571413	Department of Pathology and Laboratory Medicine, AKUH Laboratories, Karachi, Pakistan	Department of Virology	A. Kanji; A. Nasir; A. Samreen; A.R. Bukhari; Aamer Ikram; Abdul Ahad; J. Ashraf; Massab Umair; Muhammad Ammar; Muhammad Salman; Nazish Badar; Qasim Ali; R. Hasan; Syed Adnan Haider; U.B. Aamir; Z. Hasan; Zaira Rehman
EPI_ISL_7201444, EPI_ISL_7624156, EPI_ISL_7624213	Department of Virology and Immunology, University of Helsinki and Helsinki University Hospital, Huslab Finland	Department of Virology, Faculty of Medicine, University of Helsinki, Helsinki, Finland	Hanna Jarva; Hanna Liimatainen; Hanna Vauhkonen; Hussein Alburkat; Maija Lappalainen; Mert Erdin; Olli Vapalahti; Phuoc Truong; Ravi Kant; Sari Hannula; Satu Kurkela; Teemu Smura
EPI_ISL_7623676	Dept of Microbilogy, Maulana Azad Medical College	J6094_MAMC_LNH	Avinash Lomash; Mohammad Faruq; Oves Siddiqui; SCOG_MAMC_LNH; Seema Kapoor. Sunil Kumar Polipalli; Somesh Kumar; Suresh Kumar
EPI_ISL_6972689	Dept. of Laboratory Medicine	Dept. of Laboratory Medicine	Claudia Weber; Fabian Konig; Harald Esterbauer; Oswald Wagner; Robert Strassl; Sabina Plumer; Victoria Six
EPI_ISL_7464539, EPI_ISL_7464543	Dept. of Microbiology and Infection Control, Akershus University Hospital HF	Dept. of Microbiology and Infection Control, Akershus University Hospital HF	Alexander Hesselberg Løvestad; Hege Vangstein Aamot
EPI_ISL_7121080	Diagnofirm Medical Laboratories	Botswana Harvard HIV Reference Laboratory	Boitumelo Zuze; Botshelo Radibe; Dorcas Maruapula; Joseph Makhema; Keoratile Ntshambiwa; Kgomotso Moruisi; Legodile Kooepile; Mosepele Mosepele; Mphaphi B. Mbulawa; Ontlametse T. Bareng; Pamela Smith-Lawrence; Roger Shapiro; Sefetogi Ramaologa; Shahin Lockman; Sikhulile Moyo; Simani Gaseitsiwe; Thongbotho Mphoyakgosi; Wonderful T. Choga
EPI_ISL_6959926, EPI_ISL_6959935, EPI_ISL_6959993, EPI_ISL_7406117, EPI_ISL_7406118, EPI_ISL_7406119, EPI_ISL_7406124, EPI_ISL_7406125, EPI_ISL_7406126			
see above	Division of Emerging Infectious Diseases, Bureau of Infectious Diseases Diagnosis Control, Korea Disease Control and Prevention Agency	Division of Emerging Infectious Diseases, Bureau of Infectious Diseases Diagnosis Control, Korea Disease Control and Prevention Agency	Ae Kyung Park; Chae Young Lee; Eun-Jin Kim; Heui Man Kim; Hyuck Jin Lee; Il-Hwan Kim; Jeong-Ah Kim; Jeong-Min Kim
EPI_ISL_6842156, EPI_ISL_6842159, EPI_ISL_6842162, EPI_ISL_6842163, EPI_ISL_6842165, EPI_ISL_7452732, EPI_ISL_7452733, EPI_ISL_7452734, EPI_ISL_7452735, EPI_ISL_7452736, EPI_ISL_7452737, EPI_ISL_7452738, EPI_ISL_7452741, EPI_ISL_7452742, EPI_ISL_7452744, EPI_ISL_7452745, EPI_ISL_7452746, EPI_ISL_7452749, EPI_ISL_7452750, EPI_ISL_7452751, EPI_ISL_7452758, EPI_ISL_7452761, EPI_ISL_7452762, EPI_ISL_7452763, EPI_ISL_7452764, EPI_ISL_7452765, EPI_ISL_7452771, EPI_ISL_7452780, EPI_ISL_7452781, EPI_ISL_7452782, EPI_ISL_7452783, EPI_ISL_7452785, EPI_ISL_7452792, EPI_ISL_7452793, EPI_ISL_7452794, EPI_ISL_7452795, EPI_ISL_7452796, EPI_ISL_7452797, EPI_ISL_7452798, EPI_ISL_7452799, EPI_ISL_7452800, EPI_ISL_7456525, EPI_ISL_7456526, EPI_ISL_7544929			
see above	Division of Medical Virology, National Health Laboratory Service (NHLS), Tygerberg Hospital / Stellenbosch University	Division of Medical Virology, National Health Laboratory Service (NHLS), Tygerberg Hospital / Stellenbosch University	Gert van Zyl; Kamela Mahlakwane; Shannon Wilson; Susan Engelbrecht; Tania Stander; Tongai Maponga; Wolfgang Preiser
EPI_ISL_7062590, EPI_ISL_7506397, EPI_ISL_7506422, EPI_ISL_7506490	Dr. Risch Ostschweiz AG	Dr Risch Laboratory	Dominique Fabien Hilti; Faina Wehrli; Lorenz Risch; Martin Risch; Nadia Wohlwend; Sinem Kas; Thomas Bodmer
EPI_ISL_7469070	Dutch COVID-19 response team	Medical Microbiology, Maastricht University Medical Centre	Brian van der Veer*; Carmen Reumkens; Christian Hoebe; Erik Beuken; Jozef Dingemans*; Lieke van Alphen; Paul Savelkoul
EPI_ISL_6841607, EPI_ISL_6841608, EPI_ISL_6841609, EPI_ISL_6841610, EPI_ISL_6841611, EPI_ISL_6841612, EPI_ISL_6841613, EPI_ISL_6841614, EPI_ISL_6841615, EPI_ISL_6841616, EPI_ISL_6841617, EPI_ISL_6841618, EPI_ISL_6841619, EPI_ISL_7470657, EPI_ISL_7471412, EPI_ISL_7471413, EPI_ISL_7471451, EPI_ISL_7471520, EPI_ISL_7471548, EPI_ISL_7471549, EPI_ISL_7471975			
see above	Dutch COVID-19 response team	National Institute for Public Health and the Environment (RIVM)	Adam Meijer; AnneMarie van den Brandt; Annelies Kroneman; Bas van der Veer; Chantal Reusken; Dennis Schmitz; Dirk Eggink; Florian Zwagemaker; Harry Vennema; Ivo van Walle; Jeroen Cremer; Jil Kocken; Karim Hajji; Kim Freriks; Linda van Someren; Lisa Wijsman; Lynn Aarts; Ryanne Jaarsma; Sanne Bos; Sharon van den Brink; Stijn van Rossum; on behalf of the national COVID-19 response team
EPI_ISL_6826713, EPI_ISL_6826714, EPI_ISL_6989667, EPI_ISL_7015154, EPI_ISL_7160041, EPI_ISL_7160042	Dynacare	National Microbiology Laboratory (NML)	Anna Majer; Anneliese Landgraff; CanCOGeN's metadata curation team; Darian Hole; Dynacare Brampton COVID-19 Diagnostic team; Elsie Grudeski; Gary Van Domselaar; Gordon Jolly; Grace Seo; Jennifer Tanner; Madison Chapel; Morag Graham; Natalie Knox; Nathalie Bastien; Philip Mabon; Public Health Agency of Canada CanCOGeN team; Rhiannon Huzarewich; Russell Mandes; Shari Tyson; Timothy Booth; Yan Li
EPI_ISL_6949582, EPI_ISL_7123666, EPI_ISL_7123680, EPI_ISL_7123692, EPI_ISL_7330020, EPI_ISL_7330021, EPI_ISL_7330161, EPI_ISL_7464335, EPI_ISL_7464340, EPI_ISL_7464346			
see above	Edmonton Provincial Lab	Alberta Precision Labs (APL)	Buss; Croxen M; Deo A; Dieu P; E; Ferrato C; Gill K; Khan F; Koleva P; Li V; Lloyd C; Lynch T; Ma R; Murphy S; Pabbaraju K; Shokoples S; Thayer J; Tipples G; Whitehouse M; Wong A; Yu C; Zelyas N
EPI_ISL_7547732, EPI_ISL_7547733	Everight diagnostics (Abuja)	National Reference Laboratory, Nigeria Centre for Disease Control	Catherine Okoi; Chimaobi Chukwu; Dr Ifedayo Adetifa; Dr Ndodo Nnaemeka; Dr Omoare Adesuyi; Nwando Mba; Olajumoke Babatunde; Olusola Anuoluwapo Akanbi; Oyeronke Ayansola
EPI_ISL_7313687	Florida Bureau of Public Health Laboratories	Florida Bureau of Public Health Laboratories	Jason Blanton; Namratha Tarigopula; Sarah Schmedes; Tiffany Splatt
EPI_ISL_7154399, EPI_ISL_7497709, EPI_ISL_7497711, EPI_ISL_7497739, EPI_ISL_7497740, EPI_ISL_7497741, EPI_ISL_7497742, EPI_ISL_7497767			
see above	Fulgent Genetics	Centers for Disease Control and Prevention Division of Viral Diseases, Pathogen Discovery	Becky Tsai; Benafsh Sapra; Benjamin Rambo-Martin; Christopher Gulvick; Clinton Paden; Dakota Howard; Dhwani Batra; Doreen Ng; Duncan MacCannell; Erisa Sula; Harry Gao; James Xie; Jason Caravas; John Gao; Joseph Fierro; Kristine Lacek; Matthew Schmerer; Mickey Li; Peter Cook; Scott Sammons; Shatavia Morrison; Tymeckia Kendall; Victoria Caban Figueroa; Yan Meng; Yvette Unoarumhi
EPI_ISL_6971572, EPI_ISL_6989155, EPI_ISL_7248772, EPI_ISL_7248778, EPI_ISL_7248797, EPI_ISL_7248805, EPI_ISL_7248814, EPI_ISL_7248821, EPI_ISL_7470261			
see above	Furst Medical Laboratory	Norwegian Institute of Public Health, Department of Virology	Atiya R Ali; Debech Nadia; Engebretsen Serina Beate; Garcia Llorente Ignacio; Hilde Elshaug; Hilde Vollan; Jon Bråte; Kamilla Heddeland Instefjord; Karoline Bragstad; Kathrine Stene-Johansen; Line Victoria Moen; Marie Paulsen Madsen; Olav Hungnes; Pedersen Benedikte Nevjen; Rasmus Riis Kopperud
EPI_ISL_7473158	GA Department of Public Health	GA Department of Public Health	Aliyah Fields; Jonathan Edwards; Sharmila Talekar; Stacy Reeves; Taylor Smith; Tonia Parrott
EPI_ISL_7173962	GENEPATH	NCL, Pune	Ajinkya Khilari; Anu Raghunathan; Bhagyashree Litikar; Dhanasekaran Shanmugam; Divya Niveditha; Jugal Kanekar; Shikha Takur
EPI_ISL_7313494, EPI_ISL_7478195, EPI_ISL_7478220, EPI_ISL_7478233	GH A.CHENEVIER-H.MONDOR	Department of Virology, Henri Mondor University Hospital, Assistance Publique Hôpitaux de Paris, Université Paris-Est Créteil, INSERM U955	Alexandre Soulier; Christophe Rodriguez; Elisabeth Trawinski; Guillaume Gricourt; Jean-Michel Pawlotsky; Melissa N'Debi; Slim Fourati; Vanessa Demontant
EPI_ISL_6892613, EPI_ISL_6892620, EPI_ISL_6892631, EPI_ISL_6892639, EPI_ISL_6892644, EPI_ISL_6892650, EPI_ISL_6892653, EPI_ISL_6892659, EPI_ISL_6892661, EPI_ISL_6892665, EPI_ISL_6892674, EPI_ISL_6892683, EPI_ISL_6892693			

see above	Germano de Sousa	Instituto Nacional de Saude (INSA)	Borges et al
EPI_ISL_7545349, EPI_ISL_7545360, EPI_ISL_7545585, EPI_ISL_7596392, EPI_ISL_7596401, EPI_ISL_7596417	Gibraltar Health Authority Lab	Gibraltar Health Authority Covid-19 Laboratory	Bruna Martins; Dr Daniel Cassaglia; Dr Martyn Bell; Dr Nicholas Cortes; Dr Zoe Vincent; Sofia Lavelle
EPI_ISL_7549110, EPI_ISL_7549111	Gravity Diagnostics, LLC	Gravity Diagnostics, LLC	Gravity Diagnostics
EPI_ISL_7544692	Great Brak River Clinic wc GBC	NHLS/UCT	Arash Iranzadeh; Bruna Galvao; Carolyn Williamson; Deelan Doolabh; Diana Hardie; Gert Marais; Innocent Mudau; Luicer Olubayo; Lynn Tyers; Marvin Hsiao; Nokuzola Mbhele; Rageema Joseph; Stephen Korsman
EPI_ISL_7543874, EPI_ISL_7544059, EPI_ISL_7544158, EPI_ISL_7544262, EPI_ISL_7544652	Groote Schuur Hospital wc GSH	NHLS/UCT	Arash Iranzadeh; Bruna Galvao; Carolyn Williamson; Deelan Doolabh; Diana Hardie; Gert Marais; Innocent Mudau; Luicer Olubayo; Lynn Tyers; Marvin Hsiao; Nokuzola Mbhele; Rageema Joseph; Stephen Korsman
EPI_ISL_6913917	Grupo CR Diagnosticos	Instituto Adolfo Lutz Strategic Laboratory	Claudio Tavares Sacchi; Karoline Rodrigues Campos
EPI_ISL_6900139, EPI_ISL_6900141, EPI_ISL_6900142, EPI_ISL_6900143	HELEN JOSEPH LABORATORY	National Institute for Communicable Diseases of the National Health Laboratory Service	Amoako DG; Bhiman JN; Everatt J; Ismail A; Mahlangu B; Mnguni A; Mohale T; Ntuli N; Scheepers C
EPI_ISL_6698790	HOME QUARANTINE TASKFORCE	Hong Kong Department of Health	Alan K.L. Tsang; Edman T.K. Lam; Ken H.L. Ng; Peter C.W. Yip; Rickjason C.W. Chan
EPI_ISL_7610402	HOPITAL SAINT ANDRE	CNR Virus des Infections Respiratoires - France SUD	Antonin Bal; Bruno Lina; Bruno Simon; Gregory Destras; Gwendolyne Burfin; Hadrien Regue; Laurence Josset; Martine Valette; Quentin Semanas
EPI_ISL_7045214, EPI_ISL_7571637, EPI_ISL_7571638	HOSPITAL UNIVERSITARI DE BELLVITGE	Microbiology Department	Aida Gonzalez-Diaz; Anna Carrera-Salinas Yolanda Hernandez; Carmen Ardanuy; Daniel Rodirguez; Jordi Camara; Jordi Niubó; Laura Calatayud; M Angeles Domínguez; Sara Marti; Veronica Saez; Yolanda Hernandez
EPI_ISL_7624161, EPI_ISL_7624188, EPI_ISL_7624204, EPI_ISL_7624230, EPI_ISL_7624232	HOSPITAL UNIVERSITARIO SON ESPASES	HOSPITAL UNIVERSITARIO SON ESPASES	Dr. Antonio Oliver; Dr. Carla López-Causapé; Dr. Gabriel Cabot; Hospital Universitario Son Espases; on behalf of Servicio de Microbiología
EPI_ISL_7565186, EPI_ISL_7565187	Harvard University	Infectious Disease Program, Broad Institute of Harvard and MIT	B.L.; D.J.; Dobbins, S.; K.J.; MacInnis; P.C.; Park; Petros, B.; Sabeti; Siddle; Slack, S.; Springer, M.; Welch, N.
EPI_ISL_7470201	Haukeland University Hospital, Dept. of Microbiology	Norwegian Institute of Public Health, Department of Virology	Atiya R Ali; Debech Nadia; Engebretsen Serina Beate; Garcia Llorente Ignacio; Hilde Elshaug; Hilde Vollan; Jon Bråte; Kamilla Heddeland Instefjord; Karoline Bragstad; Kathrine Stene-Johansen; Line Victoria Moen; Marie Paulsen Madsen; Olav Hungnes; Pedersen Benedikte Nevjen; Rasmus Riis Kopperud
EPI_ISL_7142185, EPI_ISL_7142260, EPI_ISL_7184579	Health Services Laboratories	Wellcome Sanger Institute for the COVID-19 Genomics UK (COG-UK) Consortium	Cordelia Langford; David K. Jackson; Dominic Kwiatkowski; Ewan Harrison; Health Services Laboratories and Alex Alderton; Ian Johnston; Jeffrey Barrett; John Sillitoe on behalf of the Wellcome Sanger Institute COVID-19 Surveillance Team; Roberto Amato; Sonia Goncalves
EPI_ISL_7544466	Helderberg Hospital wc HHH	NHLS/UCT	Arash Iranzadeh; Bruna Galvao; Carolyn Williamson; Deelan Doolabh; Diana Hardie; Gert Marais; Innocent Mudau; Luicer Olubayo; Lynn Tyers; Marvin Hsiao; Nokuzola Mbhele; Rageema Joseph; Stephen Korsman
EPI_ISL_7337440, EPI_ISL_7337441, EPI_ISL_7337442, EPI_ISL_7337443, EPI_ISL_7337444, EPI_ISL_7337445, EPI_ISL_7337446, EPI_ISL_7337447, EPI_ISL_7337448, EPI_ISL_7337449, EPI_ISL_7337450, EPI_ISL_7337451, EPI_ISL_7337452, EPI_ISL_7337453, EPI_ISL_7337454, EPI_ISL_7337455, EPI_ISL_7337458, EPI_ISL_7337460, EPI_ISL_7337461, EPI_ISL_7337462			
see above	Helen Joseph Laboratory	National Institute for Communicable Diseases of the National Health Laboratory Service	Amoako DG; Bhiman JN; Everatt J; Ismail A; Mahlangu B; Mnguni A; Mohale T; Ntuli N; Scheepers C
EPI_ISL_7497723	Helix	Centers for Disease Control and Prevention Division of Viral Diseases, Pathogen Discovery	Benjamin Rambo-Martin; Christopher Gulvick; Clinton Paden; Dakota Howard; Dhwani Batra; Duncan MacCannell; Erisa Sula; Helix CA; Jason Caravas; Kristine Lacek; Matthew Schmerer; Peter Cook; Scott Sammons; Shatavia Morrison; Tymeckia Kendall; Victoria Caban Figueroa; Yvette Unoarumhi
EPI_ISL_7265236, EPI_ISL_7265237, EPI_ISL_7462215, EPI_ISL_7620968, EPI_ISL_7620969, EPI_ISL_7620970, EPI_ISL_7620971, EPI_ISL_7620972, EPI_ISL_7620973, EPI_ISL_7620977, EPI_ISL_7621010, EPI_ISL_7621011, EPI_ISL_7621210, EPI_ISL_7621349, EPI_ISL_7621350, EPI_ISL_7621351, EPI_ISL_7621352, EPI_ISL_7621353, EPI_ISL_7621907, EPI_ISL_7621908, EPI_ISL_7621909, EPI_ISL_7621910, EPI_ISL_7621914, EPI_ISL_7621915, EPI_ISL_7621916, EPI_ISL_7621917			
see above	Histopath	NSW Health Pathology - Institute of Clinical Pathology and Medical Research; Westmead Hospital; University of Sydney	Arnott A.; Draper J.; Gall M.; Martinez E.; Rockett R.; Sintchenko V.; on behalf of ICPMR
EPI_ISL_6590782, EPI_ISL_6832108	Home Quarantine Taskforce	Hong Kong Department of Health	Alan K.L. Tsang; Edman T.K. Lam; Ken H.L. Ng; Peter C.W. Yip; Rickjason C.W. Chan
EPI_ISL_6716890, EPI_ISL_6716902	Hong Kong Department of Health	School of Public Health, The University of Hong Kong	Dominic N.C. Tsang; Haogao Gu; Leo L.M. Poon; Malik Peiris
EPI_ISL_7373061, EPI_ISL_7373165, EPI_ISL_7373230	Hospital General Universitario Albacete	Hospital General Universitario Albacete	Caridad Sainz de Baranda Camino; Lorena Robles-Fonseca
EPI_ISL_6851526, EPI_ISL_6902675, EPI_ISL_6971860, EPI_ISL_7042252, EPI_ISL_7604642, EPI_ISL_7604643, EPI_ISL_7604644, EPI_ISL_7604645, EPI_ISL_7604646, EPI_ISL_7604647, EPI_ISL_7604648, EPI_ISL_7604649, EPI_ISL_7604652, EPI_ISL_7604653, EPI_ISL_7604654, EPI_ISL_7604659, EPI_ISL_7604693, EPI_ISL_7604694, EPI_ISL_7604695, EPI_ISL_7604696, EPI_ISL_7604715			
see above	Hospital General Universitario Gregorio Marañón	Hospital General Universitario Gregorio Marañón	Cristina Rodriguez-Grande; Darío García de Viedma; Jorge Rodríguez-Grande; Julia Suárez; Laura Pérez-Lago; Marta Herranz Martin; Patricia Muñoz; Pedro Sola Campoy; Pilar Catalán; Sergio Buenestado Serrano; Victor Manuel de la Cueva
EPI_ISL_7571377, EPI_ISL_7571378, EPI_ISL_7571388, EPI_ISL_7571389	Hospital General Universitario de Ciudad Real	Hospital General Universitario de Ciudad Real	Cristina Colmenarejo; José Martínez-Alarcón; Lidia García-Agudo; Marta Torres-Narbona; Soledad Illescas Fernández-Bermejo
EPI_ISL_7204336	Hospital Universitari Dr. Josep Trueta	Institut d'Investigació Biomèdica de Girona | Hospital Universitari Dr. Josep Trueta	Bernat del Olmo; Mel·lina Pinsach; Meritxell Deulofeu; Nuria Esther Neto; Paula Costa
EPI_ISL_7598538	Hospital Universitari Joan XXIII de Tarragona	Hospital Universitari Vall d'Hebron - Vall d'Hebron Institut de Recerca	Alejandra González-Sánchez; Andrés Antón; Ariadna Rando; Carla Castillo; Cristina Andrés; Damir Garcia-Cehic; Josep Quer; Juliana Esperalba; Karen García; Maria Carmen Martin; Maria Gema Codina; Maria Piñana; Rodrigo Vásquez; Tomàs Pumarola
EPI_ISL_7050911, EPI_ISL_7050918, EPI_ISL_7406515, EPI_ISL_7598503, EPI_ISL_7598554, EPI_ISL_7598569	Hospital Universitari Vall d'Hebron - Vall d'Hebron Institut de Recerca	Hospital Universitari Vall d'Hebron - Vall d'Hebron Institut de Recerca	Alejandra González-Sánchez; Andrés Antón; Ariadna Rando; Carla Castillo; Cristina Andrés; Damir Garcia-Cehic; Josep Quer; Juliana Esperalba; Karen García; Maria Carmen Martin; Maria Gema Codina; Maria Piñana; Rodrigo Vásquez; Tomàs Pumarola
EPI_ISL_7277268	Hospital Universitario Son Espases	Hospital Universitario Son Espases	Dr. Antonio Oliver; Dr. Carla López-Causapé; Dr. Gabriel Cabot; Hospital Universitario Son Espases; on behalf of Servicio de Microbiología
EPI_ISL_7329633	Hospital Universitario de Guadalajara	Hospital General Universitario de Ciudad Real	Cristina Colmenarejo; José Martínez-Alarcón; Lidia García-Agudo; Marta Torres-Narbona; Soledad Illescas Fernández-Bermejo
EPI_ISL_7156454	Hospital Ángeles Lomas	Instituto de diagnóstico y Referencia Epidemiologicos (INDRE)	Abril Rodriguez-Maldonado; Ariadna Medina-Benitez; Armando Rojo; Claudia Wong-Arambula; Ernesto Ramirez-Gonzalez; Fernando Gonzalez-Dominguez; Gisela Barrera-Badillo; Irma Lopez-Martinez; Joaquin Quiroz-Mercado; Leonardo Medina Arias; Lucia Hernandez-Rivas; Maribel Gonzalez-Villa; Natividad Cruz-Ortiz; Pilar Escamilla Llano; Raymundo Rodríguez Sandoval; Tatiana Nunez-Garcia; Vanessa Rivero-Arredondo
EPI_ISL_7599319	Houston Health Dept.	Houston Health Dept.	Adolfo Lara; Pamela Brown; Ryker Penn; Yanlai Lai
EPI_ISL_7415721, EPI_ISL_7415723, EPI_ISL_7415731, EPI_ISL_7415752, EPI_ISL_7415767, EPI_ISL_7415770, EPI_ISL_7415774, EPI_ISL_7415830, EPI_ISL_7415887, EPI_ISL_7602881, EPI_ISL_7602890, EPI_ISL_7602896, EPI_ISL_7603271, EPI_ISL_7603282, EPI_ISL_7603681, EPI_ISL_7603695, EPI_ISL_7603810, EPI_ISL_7603844, EPI_ISL_7603977, EPI_ISL_7604046, EPI_ISL_7604065, EPI_ISL_7604211, EPI_ISL_7604216, EPI_ISL_7604217, EPI_ISL_7604227, EPI_ISL_7604250, EPI_ISL_7604285, EPI_ISL_7604367, EPI_ISL_7604480, EPI_ISL_7604505, EPI_ISL_7612854			
see above	Houston Methodist Hospital	Houston Methodist Hospital	Ilya J. Finkelstein; James J. Davis; Jessica Cambric; Jimmy Gollihar; Kristina Reppond; Layne Pruitt; Madison N. Shyer; Marcus Nguyen; Matthew Ojeda Saavedra; Paul A. Christensen; Prasanti Yerramilli; Randall J. Olsen; Robert Olson; Ryan Gadd; S. Wesley Long; Sishir Subedi; and James M. Musser
EPI_ISL_7210427, EPI_ISL_7635857	Hrvatski zavod za javno zdravstvo	Hrvatski zavod za javno zdravstvo	Anita Jurić; Dragan Jurić; Irena Tabain; Ivana Ferenčak; Josipa Kuzle; Ljiljana Žmak; Mihaela Obrovac
EPI_ISL_7154340, EPI_ISL_7156753, EPI_ISL_7308635, EPI_ISL_7308771, EPI_ISL_7308875, EPI_ISL_7381064, EPI_ISL_7552474, EPI_ISL_7552479, EPI_ISL_7552666, EPI_ISL_7552686, EPI_ISL_7552693, EPI_ISL_7602123, EPI_ISL_7602445, EPI_ISL_7602500, EPI_ISL_7602605, EPI_ISL_7602741, EPI_ISL_7602776, EPI_ISL_7602813			
see above	IHU Mediterranee Infection	IHU Mediterranee Infection	Philippe Colson et al.
EPI_ISL_7439547, EPI_ISL_7439558, EPI_ISL_7439622, EPI_ISL_7439646, EPI_ISL_7439650, EPI_ISL_7439657, EPI_ISL_7439722, EPI_ISL_7439730, EPI_ISL_7439754, EPI_ISL_7439781, EPI_ISL_7439806, EPI_ISL_7439827, EPI_ISL_7439832, EPI_ISL_7439902, EPI_ISL_7439935			

see above	IMD - MVZ Labor Martinsried	Robert Koch Institute	
EPI_ISL_7451030, EPI_ISL_7451040, EPI_ISL_7451054, EPI_ISL_7451096, EPI_ISL_7565411	INSA	Instituto Nacional de Saude (INSA)	Borges et al
EPI_ISL_7496734	Idaho Bureau of Laboratories	Idaho Bureau of Laboratories	"R. Beukelman; Aimee Ceniseros; Christian Loera; Christopher Ball"; Matthew Charles Burns; Robert L. Voermans
EPI_ISL_7544430	Ikhwezi CDC wc IKW	NHLS/UCT	Arash Iranzadeh; Bruna Galvao; Carolyn Williamson; Deelan Doolabh; Diana Hardie; Gert Marais; Innocent Mudau; Luicer Olubayo; Lynn Tyers; Marvin Hsiao; Nokuzola Mbhele; Rageema Joseph; Stephen Korsman
EPI_ISL_7381102	Indian Council of Medical Research-National Institute of Virology, Microbial Containment Complex	Indian Council of Medical Research-National Institute of Virology, Microbial Containment Complex	Pragya D. Yadav
EPI_ISL_7166400	Indira Gandhi Memorial Hospital	Indira Gandhi Memorial Hospital	D. Fathmath Nazla Rafeeq; Dr. Ibrahim Afzal; Mr. Ibrahim Nishan Ahmed; Ms. Aishath Shuhudha; Ms. Aminath Shazleena Abdul Rahman
EPI_ISL_7607424	Infectious Disease Diagnostics Laboratory at the Children's Hospital of Philadelphia	Planet Lab, Children's Hospital of Philadelphia	Ahmed M. Moustafa; Alex Arvanitis; Andries Feder; Azad Ahmed; Bhaswati Sen; Donald C. Hall; Joshua Chang Mell; Paul J. Planet; Rebecca M. Harris; Swetha Rajagopal; Will Dampier
EPI_ISL_7497749	Infinity Biologix	Centers for Disease Control and Prevention Division of Viral Diseases, Pathogen Discovery	Benjamin Rambo-Martin; Chirayu Goswami; Christian Bixby; Christopher Gulvick; Clinton Paden; Dakota Howard; Dhwani Batra; Duncan MacCannell; Erisa Sula; Jason Caravas; Jonathan Schultz; Kristine Lacek; Matthew Schmerer; Peter Cook; Robin Grimwood; Russ Hager; Scott Sammons; Shatavia Morrison; Tymeckia Kendall; Victoria Caban Figueroa; Yihe Wang; Yvette Unoarumhi
EPI_ISL_7405329	Inselspital Bern (Covid-Track)	Institute for Infectious Diseases, University of Bern	Alban Ramette; Christian Baumann; Cora Sägesser; Franziska Suter-Riniker; Loïc Borcard; Miguel A Terrazos Miani; Nicole Liechti; Pascal Bittel; Peter Keller; Sonja Gempeler; Stefan Neuenschwander; Stephen L Leib
EPI_ISL_7117396	Institut für Labormedizin Mikrobiologie und Hygiene	Robert Koch Institute	
EPI_ISL_6832737	Institute for Medical Virology Frankfurt	Institute for Medical Virology Frankfurt	Ciesek S.; Toptan T.
EPI_ISL_6959868, EPI_ISL_6959869, EPI_ISL_6959870, EPI_ISL_6959871, EPI_ISL_6959872, EPI_ISL_6959873, EPI_ISL_6959874, EPI_ISL_7479163, EPI_ISL_7479173, EPI_ISL_7479181, EPI_ISL_7479186, EPI_ISL_7479193, EPI_ISL_7479200, EPI_ISL_7479206			
see above	Institute for Medical Virology, Frankfurt	Institute for Medical Virology, Frankfurt	Ciesek S.; Toptan T.
EPI_ISL_7404462, EPI_ISL_7404463	Institute of Epidemiology, Disease Control and Research (IEDCR)	IEDCR-ideSHi Genomics Lab	Firdausi Qadri; Hassan Afrad; Manjur Hossain Khan; Omar Hamza; Tahmina Shirin
EPI_ISL_7507055	Institute of Medical Science, University of Tokyo	Center for Influenza and Respiratory Virus Research, National Institute of Infectious Diseases (NIID)	Emi Takashita; Hideka Miura; Seiichiro Fujisaki; Yoshihiro Kawaoka; Yuko Sakai-Tagawa
EPI_ISL_6825546, EPI_ISL_6902052, EPI_ISL_6902053	Institute of Virology, Department of Hygiene, Microbiology and Public Health at Innsbruck Medical University	Institute of Virology, Department of Hygiene, Microbiology and Public Health at Innsbruck Medical University	Andreas Aufschnaiter; Barbara Falkensammer; David Bante; Dorothee von Laer; Heribert Stoiber; Lukas Perro; Stephan Amstler; Wegene Borena
EPI_ISL_7550075	Instituto Adolfo Lutz - Regional de Rio Claro	Instituto Adolfo Lutz, Interdiciplinary Procedures Center, Strategic Laboratory	Claudio Tavares Sacchi; Karoline Rodrigues Campos
EPI_ISL_7632052	Invenimus AG	Institute of Medical Virology, University of Zurich	Alexandra Trkola; Annette Audigé; Catharine Aquino; Cyril Shah; Daniel Ehrsam; Gabriela Ziltener; Guido Bloemberg; Hubert Rehrauer; Isabel Stürmer; Joel Wirz; Jon Huder; Jürg Böni; Kevin Steiner; Maria Grünberg; Maryam Zaheri; Michael Huber; Riccarda Capaul; Stefan Schmutz; Verena Kufner; Weihong Qi
EPI_ISL_7400617, EPI_ISL_7571605, EPI_ISL_7571606, EPI_ISL_7571607, EPI_ISL_7571608, EPI_ISL_7571612, EPI_ISL_7571614			
see above	Iressef Genomics lab	IRESSEF	Abdou PADANE; Ambroise AHOUIDI; Aminata DIA; Aminata MBOUP; Astou Gaye GAYE; Barada CISSE; Birahim Piere NDIAYE; Cyrille Diedhiou; Diabou Diagne; Gora LO; Khadim GUEYE; Moustapha MBOW; Nafisatou LEYE; Ndeye Coumba Toure KANE; Papa Alassane DIAW; Samba Ndiour; Seni Ndiaye; Souleymane MBOUP; Yacine DIA
EPI_ISL_7160424	Johns Hopkins Hospital Department of Pathology	Johns Hopkins Hospital Department of Pathology	Amary Fall; C. Paul Morris; David Gaston; Heba H. Mostafa; Julie M. Norton; Matthew Schwartz; Michael Forman; Raghda Eldesouki
EPI_ISL_7605595, EPI_ISL_7605596, EPI_ISL_7605597, EPI_ISL_7605598, EPI_ISL_7605599, EPI_ISL_7605600, EPI_ISL_7605601, EPI_ISL_7605617, EPI_ISL_7605618, EPI_ISL_7605619, EPI_ISL_7605622, EPI_ISL_7605641, EPI_ISL_7605642, EPI_ISL_7605643, EPI_ISL_7605644, EPI_ISL_7605645, EPI_ISL_7605646, EPI_ISL_7605647, EPI_ISL_7605648, EPI_ISL_7605649, EPI_ISL_7605650, EPI_ISL_7605680, EPI_ISL_7605681, EPI_ISL_7605683, EPI_ISL_7605686, EPI_ISL_7605690, EPI_ISL_7605706, EPI_ISL_7605707, EPI_ISL_7605741			
see above	KIMBERLEY LABORATORY	National Institute for Communicable Diseases of the National Health Laboratory Service	Amoako DG; Bhiman JN; Everatt J; Ismail A; Mahlangu B; Mnguni A; Mohale T; Ntuli N; Scheepers C; Wolter N
EPI_ISL_6794907, EPI_ISL_6989250, EPI_ISL_7413964, EPI_ISL_7495278, EPI_ISL_7495279, EPI_ISL_7495280, EPI_ISL_7495281, EPI_ISL_7495282, EPI_ISL_7495283, EPI_ISL_7495284, EPI_ISL_7495285			
see above	KU Leuven, Rega Institute, Clinical and Epidemiological Virology	KU Leuven, Rega Institute, Clinical and Epidemiological Virology	Bert Vanmechelen; Casper Geenen; Emmanuel André; Guy Baele; Joan Marti-Carerras; Joren; Lize Cuypers; Piet Maes; Raymenants; Sarah Gorissen; Simon Dellicour; Tony Wawina-Bokalanga
EPI_ISL_7622272, EPI_ISL_7622286, EPI_ISL_7622287	Kaiser Permanente NW Reginal Lab	OHSU MM Lab	Amber Halse; Jeannine Lama; Xuan Qin; Yun Wu
EPI_ISL_7219990, EPI_ISL_7220437, EPI_ISL_7220444, EPI_ISL_7286284	Karolinska University Hospital Huddinge	Karolinska University Hospital Huddinge	Annika Tiveljung Lindell; Henning Onsbring; Jan Albert; Karina Hentrich; Lynda Eneh; Maria Ropat; Martin Ekman; Natalija Gerasimcik; Robert Dyrdak; Sandra Broddesson; Shambhu Ganeshappa Aralaguppe; Tanja Normark; Tobias Allander; Valtteri Wirta; Zhibing Yun
EPI_ISL_7381109, EPI_ISL_7381114, EPI_ISL_7457425, EPI_ISL_7457426	Karolinska University Hospital Solna	Karolinska University Hospital Huddinge	Annika Tiveljung Lindell; Henning Onsbring; Jan Albert; Karina Hentrich; Lynda Eneh; Maria Ropat; Martin Ekman; Natalija Gerasimcik; Robert Dyrdak; Sandra Broddesson; Shambhu Ganeshappa Aralaguppe; Tanja Normark; Tobias Allander; Valtteri Wirta; Zhibing Yun
EPI_ISL_7433816, EPI_ISL_7443687, EPI_ISL_7443713	Klinikum Ernst von Bergmann gemeinnützige GmbH - stationärer Bereich	Robert Koch Institute	
EPI_ISL_7611152	LABORATOIRE BIOALLIANCE	CNR Virus des Infections Respiratoires - France SUD	Antonin Bal; Bruno Lina; Bruno Simon; Gregory Destras; Gwendolyne Burfin; Hadrien Regue; Laurence Josset; Martine Valette; Quentin Semanas
EPI_ISL_7224971	LACEN do Distrito Federal	Instituto Adolfo Lutz Strategic Laboratory	Claudio Tavares Sacchi; Karoline Rodrigues Campos; Marlon Benedito Nascimento Santos
EPI_ISL_7550076	LACEN do Distrito Federal	Instituto Adolfo Lutz, Interdiciplinary Procedures Center, Strategic Laboratory	Claudio Tavares Sacchi; Karoline Rodrigues Campos
EPI_ISL_7610177	LAM CERBALLIANCE	CNR Virus des Infections Respiratoires - France SUD	Antonin Bal; Bruno Lina; Bruno Simon; Gregory Destras; Gwendolyne Burfin; Hadrien Regue; Laurence Josset; Martine Valette; Quentin Semanas
EPI_ISL_6647956, EPI_ISL_6647957, EPI_ISL_6647958, EPI_ISL_6647959, EPI_ISL_6647960, EPI_ISL_6647961, EPI_ISL_6647962, EPI_ISL_6698792, EPI_ISL_6704863, EPI_ISL_6704864, EPI_ISL_6704865, EPI_ISL_6704866, EPI_ISL_6704867, EPI_ISL_6704868, EPI_ISL_6704869, EPI_ISL_6704870, EPI_ISL_6704871, EPI_ISL_6704872, EPI_ISL_6704873, EPI_ISL_6704874, EPI_ISL_6704875, EPI_ISL_6704876			
see above	LANCET LABORATORY	National Institute for Communicable Diseases of the National Health Laboratory Service	Amoako DG; Bhiman JN; Everatt J; Glass A; Ismail A; Mahlangu B; Mnguni A; Mohale T; Ntuli N; Scheepers C; Viana R; Wolter N
EPI_ISL_6901960, EPI_ISL_6901961, EPI_ISL_7473154	LATE - Laboratório de Técnicas Especiais - Hospital Israelita Albert Einstein	LATE - Laboratório de Técnicas Especiais - Hospital Israelita Albert Einstein	Alexandre Hideaki Takara; Ana Paula Moreira Salles; Anelisie da Silva Santos; Deyvid Amgarten; Erick Gustavo Dorlass; Fernanda de Mello Malta; João Renato Rebello Pinho; Luiz Vicente Rizzo; Marcio Anunciacao Menezes; Pedro Henrique Sebe Rodrigues; Raquel Riyuzo
EPI_ISL_7472277	LKO	Jessa	Severine Berden et al. on behalf of the Jessa_cmdLab
EPI_ISL_7456529	LSUHS Emerging Viral Threat Lab	LSUHS Emerging Viral Threat Laboratory	Adrian Almodovar; Alexander Mijalis; Andrew D. Yurochko; Christopher G. Kevil; Gregory L. Ware; Jennifer L. Carroll; Jeremy P. Kamil; John A. Vanchiere; Krista Queen; Maarten Van Diest; Rona S. Scott
EPI_ISL_7339434, EPI_ISL_7339435, EPI_ISL_7339436	Lab voor klinische biologie	Lab voor klinische biologie	Bruno Verhasselt; Hannelore Hamerlinck; Marija Janevska; May-Linh Truong
EPI_ISL_7589521	Labo Analyses Med	National Reference Center for Viruses of Respiratory Infections, Institut	Angela Brisebarre; Camille Capel; Christophe Malabat; Corinne Maufrais; Etienne Simon-Lorière; Frédéric Lemoine; Julien Fumey; Louise Lefrançois; Marion Barbet; Maud Vanpeene; Méline Bizard; Philippe GIRARD; Slim El Khiari; Sylvie Behillil; Sylvie Van der Werf; Vincent Enouf

		Pasteur, Paris	
EPI_ISL_7437787	Labor Prof. Dr. G. Enders MVZ GbR	Robert Koch Institute	
EPI_ISL_7450035, EPI_ISL_7450728	Laboratoire BIORANCE	CHU Pontchaillou	DE TAYRAC Marie; DENOUAL Florent; ETCHEVERRY Amandine; FEBREAU Christine; GALIBERT Marie Dominique; GROLHIER Claire; JAGLINE Steven; PRONIER Charlotte; QUENET Benjamin; SASSI Mohamed; THIBAULT Vincent
EPI_ISL_6971091	Laboratoire analyse med	LABORIZON CENTRE BIOGROUP	HAGUENOER EVE; HOLSTEIN ANNE; JIMENEZ MELANIE; LEFLEUTER NICOLAS; POITIRON GREGOIRE
EPI_ISL_7309168	Laboratorio Central de Saude Publica do Rio Grande do Sul/Centro Estadual de Vigilancia em Saude	Centro de Desenvolvimento Cientifico e Tecnologico (CDCT)/Centro Estadual de Vigilancia em Saude	Alana Rossetti; Cláudia Maria Dornelles da Silva; Fernanda Marques Godinho; Larissa Vitoria da Silva; Ludmila Fiorenzano Baethgen; Miguel S Andrade,; Regina Bones Barcellos; Richard Steiner Salvato; Tatiana Schaffer Gregianini
EPI_ISL_7497691	Laboratory Corporation of America	Centers for Disease Control and Prevention Division of Viral Diseases, Pathogen Discovery	Amanda Douglas; Amanda Suchanek; Andrea Throop; Ayla Burns; Benjamin Rambo-Martin; Bobbi Croy; Brian Krueger; Brian Norvell; Christopher Gulvick; Christos Petropoulos; Clinton Paden; Craig Lukasik; Dakota Howard; Debbie Boles; Dhwani Batra; Duncan MacCannell; Eyad Almasri; Goran Stevovic; Howard Engler; Hrushikesh Deshmukh; Jake Humphrey; Jana Schroth; Jason Caravas; Joe Voshell; John Pruitt; Jonathan Meltzer; Jonathan Williams; Kimberly Wagner; Kristine Lacek; Lax Iyer; Lisa Pfefferle; Lyndon Tilson; Manoj Jain; Marcia Eisenberg; Mary Cristobal; Mary Williamson; Matthew Robinson; Matthew Schmerer; Michael Levandoski; Mike Sapeta; Mindy Nye; Minoo Agarwal; Mohan Kolli; Nuthawin Charoensri; Oren Cohen; Peter Cook; Prashant Gupta; Qian Zeng; Rama Ghatti; Scott Parker; Scott Ryan; Scott Sammons; Shatavia Morrison; Stanley Letovsky; Steven Ragan; Suresh Selvaraju; Susan Countryman; Susan Hicks; Suzanne Dale; Thomas Urban; Tim Kuphal; Tricia Zwiefelhofer; Tymeckia Kendall; Victoria Caban Figueroa; Vincent Drouillon; Yvette Unoarumhi
EPI_ISL_7567146	Laboratory of Clinical Microbiology, Virology and Bioemergencies, ASST Fatebenefratelli Sacco - Sacco Hospital	Laboratory of Clinical Microbiology, Virology and Bioemergencies, ASST Fatebenefratelli Sacco - Sacco Hospital	Alberto Rizzo; Fiorenza Bracchitta; Valeria Micheli
EPI_ISL_7220176	Laboratory of Clinical Virology Heraklion Crete	Laboratory of Clinical Virology Heraklion Crete	Alexandros Zafiropoulos; George Sourvinos
EPI_ISL_7264143	Lahey Hospital	New England Biolabs	Abel, G.; B.W.; C.J.; Elfahal, M.; Flynn; Heim, K.; Karolides, M.; L. and Langhorst; Leger, P.; Michaels, L.; Pinet, K.; Skelton, T.; Sun
EPI_ISL_7543847, EPI_ISL_7543913, EPI_ISL_7543935, EPI_ISL_7543949, EPI_ISL_7543960, EPI_ISL_7544508, EPI_ISL_7544601, EPI_ISL_7544676, EPI_ISL_7544705			
see above	Lancet	NHLS/UCT	Arash Iranzadeh; Bruna Galvao; Carolyn Williamson; Deelan Doolabh; Diana Hardie; Gert Marais; Innocent Mudau; Luicer Olubayo; Lynn Tyers; Marvin Hsiao; Nokuzola Mbhele; Rageema Joseph; Stephen Korsman
EPI_ISL_6913991, EPI_ISL_6913992, EPI_ISL_6913993, EPI_ISL_6913994, EPI_ISL_6913995, EPI_ISL_6913996, EPI_ISL_6913997, EPI_ISL_6913998, EPI_ISL_6913999, EPI_ISL_6914000, EPI_ISL_6914001, EPI_ISL_6914002, EPI_ISL_6914003, EPI_ISL_6914004, EPI_ISL_6914005, EPI_ISL_6914006, EPI_ISL_6914007			
see above	Lancet Laboratories	National Institute for Communicable Diseases of the National Health Laboratory Service	Amoako DG; Bhiman JN; Everatt J; Ismail A; Mahlangu B; Mnguni A; Mohale T; Ntuli N; Scheepers C; Wolter N
EPI_ISL_7420297, EPI_ISL_7420298, EPI_ISL_7420371, EPI_ISL_7420381	Landesgesundheitsamt Baden-Württemberg	Robert Koch Institute	
EPI_ISL_7543863	Langa Clinic wc LAN	NHLS/UCT	Arash Iranzadeh; Bruna Galvao; Carolyn Williamson; Deelan Doolabh; Diana Hardie; Gert Marais; Innocent Mudau; Luicer Olubayo; Lynn Tyers; Marvin Hsiao; Nokuzola Mbhele; Rageema Joseph; Stephen Korsman
EPI_ISL_7620900, EPI_ISL_7621182, EPI_ISL_7621503, EPI_ISL_7621817	Laverty Pathology	NSW Health Pathology - Institute of Clinical Pathology and Medical Research; Westmead Hospital; University of Sydney	Arnott A.; Draper J.; Gall M.; Martinez E.; Rockett R.; Sintchenko V.; on behalf of ICPMR
EPI_ISL_7348417, EPI_ISL_7348427	Lifebrain Covid Labor GmbH	Lifebrain Covid Labor GmbH	Filip Sima
EPI_ISL_6821008, EPI_ISL_6916148, EPI_ISL_7023724, EPI_ISL_7144808, EPI_ISL_7144986, EPI_ISL_7148837, EPI_ISL_7187307, EPI_ISL_7200868, EPI_ISL_7290346, EPI_ISL_7293790, EPI_ISL_7293812, EPI_ISL_7293816, EPI_ISL_7293841, EPI_ISL_7293869, EPI_ISL_7293903, EPI_ISL_7294032, EPI_ISL_7294185, EPI_ISL_7296873, EPI_ISL_7301592, EPI_ISL_7301667, EPI_ISL_7305287, EPI_ISL_7344013, EPI_ISL_7344598, EPI_ISL_7344677, EPI_ISL_7346484, EPI_ISL_7351048, EPI_ISL_7351423, EPI_ISL_7351936, EPI_ISL_7353170, EPI_ISL_7382609, EPI_ISL_7383321, EPI_ISL_7385376, EPI_ISL_7389680, EPI_ISL_7392768, EPI_ISL_7397825, EPI_ISL_7484328, EPI_ISL_7488052, EPI_ISL_7490160, EPI_ISL_7491433, EPI_ISL_7491455, EPI_ISL_7491475, EPI_ISL_7492192, EPI_ISL_7492231, EPI_ISL_7511780, EPI_ISL_7514364, EPI_ISL_7514804, EPI_ISL_7515071, EPI_ISL_7515102, EPI_ISL_7515212, EPI_ISL_7515213, EPI_ISL_7515290, EPI_ISL_7515719, EPI_ISL_7516761, EPI_ISL_7535745, EPI_ISL_7535814, EPI_ISL_7535856, EPI_ISL_7535938, EPI_ISL_7536494, EPI_ISL_7536603, EPI_ISL_7537524, EPI_ISL_7538327, EPI_ISL_7539165, EPI_ISL_7539548, EPI_ISL_7539590, EPI_ISL_7539720, EPI_ISL_7541054, EPI_ISL_7575953, EPI_ISL_7577543, EPI_ISL_7578261, EPI_ISL_7578477, EPI_ISL_7578511, EPI_ISL_7579253, EPI_ISL_7624651, EPI_ISL_7624729, EPI_ISL_7624812, EPI_ISL_7624845, EPI_ISL_7624907, EPI_ISL_7624982, EPI_ISL_7625130, EPI_ISL_7625133, EPI_ISL_7625144, EPI_ISL_7625153, EPI_ISL_7625195, EPI_ISL_7625239, EPI_ISL_7625258, EPI_ISL_7625280, EPI_ISL_7625304, EPI_ISL_7625309, EPI_ISL_7625315, EPI_ISL_7625322, EPI_ISL_7625367, EPI_ISL_7625383, EPI_ISL_7625399, EPI_ISL_7625421, EPI_ISL_7625526, EPI_ISL_7625576, EPI_ISL_7625609, EPI_ISL_7625644, EPI_ISL_7625694, EPI_ISL_7625703, EPI_ISL_7627930, EPI_ISL_7627979, EPI_ISL_7627980, EPI_ISL_7628180, EPI_ISL_7628307, EPI_ISL_7628386, EPI_ISL_7628524, EPI_ISL_7628571, EPI_ISL_7628637, EPI_ISL_7628726, EPI_ISL_7628901, EPI_ISL_7628998, EPI_ISL_7629019, EPI_ISL_7629020, EPI_ISL_7629176, EPI_ISL_7629711, EPI_ISL_7629998, EPI_ISL_7630011, EPI_ISL_7630066, EPI_ISL_7630877, EPI_ISL_7630946, EPI_ISL_7630956, EPI_ISL_7630960, EPI_ISL_7630981, EPI_ISL_7630998, EPI_ISL_7631021, EPI_ISL_7631066, EPI_ISL_7631081, EPI_ISL_7631083, EPI_ISL_7631094, EPI_ISL_7631138, EPI_ISL_7631408, EPI_ISL_7631425, EPI_ISL_7631506, EPI_ISL_7632206, EPI_ISL_7632745, EPI_ISL_7632760, EPI_ISL_7634914, EPI_ISL_7635213, EPI_ISL_7635249, EPI_ISL_7635336, EPI_ISL_7635376, EPI_ISL_7635385, EPI_ISL_7635502, EPI_ISL_7635701, EPI_ISL_7635816			
see above	Lighthouse Lab in Alderley Park	Wellcome Sanger Institute for the COVID-19 Genomics UK (COG-UK) Consortium	Cordelia Langford; David K. Jackson; Dominic Kwiatkowski; Ewan Harrison; Ian Johnston; Jacquelyn Wynn; Jeffrey Barrett; John Sillitoe on behalf of the Wellcome Sanger Institute COVID-19 Surveillance Team; Mairead Hyland; Roberto Amato; Sonia Goncalves; The Lighthouse Lab in Alderley Park and Alex Alderton
EPI_ISL_6869226, EPI_ISL_6869363, EPI_ISL_6869407, EPI_ISL_6869556, EPI_ISL_6873612, EPI_ISL_6920174, EPI_ISL_6926451, EPI_ISL_6926743, EPI_ISL_6927893, EPI_ISL_7019951, EPI_ISL_7022011, EPI_ISL_7027157, EPI_ISL_7027809, EPI_ISL_7029582, EPI_ISL_7031141, EPI_ISL_7031412, EPI_ISL_7035922, EPI_ISL_7036426, EPI_ISL_7145006, EPI_ISL_7145516, EPI_ISL_7148141, EPI_ISL_7148719, EPI_ISL_7158402, EPI_ISL_7158414, EPI_ISL_7158423, EPI_ISL_7158488, EPI_ISL_7158498, EPI_ISL_7158548, EPI_ISL_7292889, EPI_ISL_7292960, EPI_ISL_7294550, EPI_ISL_7294585, EPI_ISL_7295180, EPI_ISL_7295212, EPI_ISL_7295689, EPI_ISL_7296032, EPI_ISL_7296139, EPI_ISL_7296254, EPI_ISL_7296303, EPI_ISL_7297042, EPI_ISL_7297075, EPI_ISL_7297120, EPI_ISL_7299420, EPI_ISL_7299535, EPI_ISL_7299536, EPI_ISL_7299709, EPI_ISL_7299880, EPI_ISL_7302122, EPI_ISL_7302177, EPI_ISL_7302222, EPI_ISL_7302501, EPI_ISL_7302554, EPI_ISL_7303186, EPI_ISL_7303590, EPI_ISL_7303781, EPI_ISL_7304114, EPI_ISL_7304964, EPI_ISL_7305445, EPI_ISL_7305675, EPI_ISL_7305684, EPI_ISL_7305734, EPI_ISL_7305755, EPI_ISL_7305800, EPI_ISL_7305876, EPI_ISL_7305903, EPI_ISL_7305934, EPI_ISL_7306117, EPI_ISL_7306238, EPI_ISL_7306338, EPI_ISL_7306343, EPI_ISL_7306399, EPI_ISL_7343337, EPI_ISL_7343938, EPI_ISL_7343985, EPI_ISL_7344167, EPI_ISL_7344174, EPI_ISL_7345819, EPI_ISL_7345863, EPI_ISL_7345932, EPI_ISL_7345966, EPI_ISL_7345975, EPI_ISL_7346136, EPI_ISL_7346249, EPI_ISL_7346862, EPI_ISL_7346896, EPI_ISL_7347059, EPI_ISL_7347482, EPI_ISL_7350891, EPI_ISL_7351023, EPI_ISL_7351093, EPI_ISL_7352907, EPI_ISL_7353351, EPI_ISL_7355546, EPI_ISL_7355957, EPI_ISL_7355992, EPI_ISL_7356061, EPI_ISL_7356171, EPI_ISL_7356256, EPI_ISL_7384310, EPI_ISL_7389585, EPI_ISL_7392688, EPI_ISL_7392706, EPI_ISL_7394189, EPI_ISL_7394919, EPI_ISL_7397103, EPI_ISL_7397308, EPI_ISL_7397319, EPI_ISL_7397480, EPI_ISL_7510398, EPI_ISL_7510491, EPI_ISL_7510550, EPI_ISL_7510572, EPI_ISL_7510800, EPI_ISL_7510886, EPI_ISL_7510922, EPI_ISL_7510928, EPI_ISL_7510978, EPI_ISL_7511143, EPI_ISL_7511154, EPI_ISL_7511233, EPI_ISL_7511305, EPI_ISL_7511455, EPI_ISL_7511587, EPI_ISL_7511662, EPI_ISL_7511677, EPI_ISL_7511702, EPI_ISL_7511820, EPI_ISL_7511832, EPI_ISL_7511896, EPI_ISL_7511986, EPI_ISL_7517098, EPI_ISL_7517101, EPI_ISL_7517216, EPI_ISL_7518683, EPI_ISL_7519628, EPI_ISL_7520531, EPI_ISL_7521048, EPI_ISL_7522619, EPI_ISL_7522673, EPI_ISL_7531008, EPI_ISL_7531228, EPI_ISL_7531322, EPI_ISL_7532295, EPI_ISL_7535753, EPI_ISL_7536021, EPI_ISL_7536166, EPI_ISL_7536246, EPI_ISL_7536342, EPI_ISL_7536453, EPI_ISL_7536705, EPI_ISL_7536927, EPI_ISL_7557186, EPI_ISL_7558117, EPI_ISL_7558170, EPI_ISL_7558350, EPI_ISL_7558383, EPI_ISL_7571990, EPI_ISL_7572056, EPI_ISL_7572178, EPI_ISL_7572425, EPI_ISL_7572906, EPI_ISL_7572919, EPI_ISL_7572943, EPI_ISL_7572961, EPI_ISL_7572962, EPI_ISL_7572986, EPI_ISL_7573003, EPI_ISL_7573055, EPI_ISL_7573101, EPI_ISL_7573102, EPI_ISL_7573151, EPI_ISL_7573158, EPI_ISL_7573256, EPI_ISL_7573278, EPI_ISL_7573306, EPI_ISL_7573335, EPI_ISL_7573358, EPI_ISL_7573377, EPI_ISL_7573415, EPI_ISL_7573450, EPI_ISL_7573467, EPI_ISL_7573468, EPI_ISL_7573477, EPI_ISL_7573884, EPI_ISL_7573917, EPI_ISL_7573931, EPI_ISL_7574004, EPI_ISL_7574007, EPI_ISL_7574049, EPI_ISL_7574070, EPI_ISL_7574201, EPI_ISL_7574281, EPI_ISL_7575331, EPI_ISL_7575392, EPI_ISL_7575420, EPI_ISL_7575480, EPI_ISL_7575510, EPI_ISL_7575511, EPI_ISL_7575543, EPI_ISL_7575550, EPI_ISL_7575586, EPI_ISL_7575588, EPI_ISL_7575633, EPI_ISL_7575684, EPI_ISL_7575753, EPI_ISL_7575773, EPI_ISL_7578677, EPI_ISL_7578871, EPI_ISL_7579130, EPI_ISL_7582829, EPI_ISL_7585552, EPI_ISL_7585596, EPI_ISL_7585650, EPI_ISL_7585720, EPI_ISL_7585731, EPI_ISL_7585732, EPI_ISL_7585734, EPI_ISL_7585741, EPI_ISL_7585789, EPI_ISL_7585847, EPI_ISL_7585936, EPI_ISL_7585968, EPI_ISL_7585991, EPI_ISL_7585998, EPI_ISL_7586089, EPI_ISL_7586090, EPI_ISL_7586102, EPI_ISL_7586111, EPI_ISL_7586288, EPI_ISL_7586290, EPI_ISL_7586324, EPI_ISL_7586362, EPI_ISL_7586434, EPI_ISL_7586500, EPI_ISL_7586596, EPI_ISL_7624515, EPI_ISL_7624601, EPI_ISL_7624610, EPI_ISL_7624706, EPI_ISL_7624769, EPI_ISL_7624865, EPI_ISL_7624949, EPI_ISL_7624976, EPI_ISL_7624991, EPI_ISL_7625874, EPI_ISL_7625890, EPI_ISL_7625957, EPI_ISL_7626058, EPI_ISL_7626063, EPI_ISL_7626092, EPI_ISL_7626095, EPI_ISL_7626105, EPI_ISL_7626118, EPI_ISL_7626122, EPI_ISL_7626123, EPI_ISL_7626132, EPI_ISL_7626147, EPI_ISL_7626176, EPI_ISL_7626207, EPI_ISL_7626229, EPI_ISL_7626304, EPI_ISL_7626319, EPI_ISL_7626327, EPI_ISL_7626440, EPI_ISL_7626502, EPI_ISL_7626530, EPI_ISL_7627296, EPI_ISL_7627311, EPI_ISL_7627327, EPI_ISL_7627334, EPI_ISL_7627354, EPI_ISL_7627355, EPI_ISL_7627363, EPI_ISL_7627416, EPI_ISL_7627492, EPI_ISL_7627503, EPI_ISL_7627529, EPI_ISL_7627668, EPI_ISL_7627706, EPI_ISL_7627760, EPI_ISL_7627771, EPI_ISL_7627780, EPI_ISL_7627787, EPI_ISL_7627790, EPI_ISL_7627815, EPI_ISL_7627838, EPI_ISL_7627841, EPI_ISL_7627868, EPI_ISL_7627879, EPI_ISL_7627885, EPI_ISL_7627891, EPI_ISL_7627937, EPI_ISL_7627940, EPI_ISL_7627973, EPI_ISL_7627975, EPI_ISL_7627995, EPI_ISL_7627997, EPI_ISL_7628000, EPI_ISL_7628008, EPI_ISL_7628050, EPI_ISL_7628053, EPI_ISL_7628102, EPI_ISL_7628111, EPI_ISL_7628142, EPI_ISL_7628157, EPI_ISL_7628199, EPI_ISL_7628213, EPI_ISL_7628232, EPI_ISL_7628255, EPI_ISL_7628287, EPI_ISL_7628343, EPI_ISL_7628415, EPI_ISL_7628424, EPI_ISL_7628425, EPI_ISL_7628466, EPI_ISL_7628522, EPI_ISL_7628530, EPI_ISL_7628549, EPI_ISL_7628550, EPI_ISL_7628576, EPI_ISL_7628577, EPI_ISL_7628591, EPI_ISL_7628633, EPI_ISL_7628674, EPI_ISL_7628709, EPI_ISL_7628780, EPI_ISL_7628795, EPI_ISL_7628817, EPI_ISL_7628857, EPI_ISL_7628910, EPI_ISL_7628932, EPI_ISL_7628934, EPI_ISL_7628944, EPI_ISL_7628971, EPI_ISL_7629002, EPI_ISL_7629073, EPI_ISL_7629085, EPI_ISL_7629087, EPI_ISL_7629116, EPI_ISL_7629126, EPI_ISL_7629149, EPI_ISL_7630217, EPI_ISL_7630247, EPI_ISL_7630295, EPI_ISL_7630305, EPI_ISL_7630312, EPI_ISL_7630319, EPI_ISL_7630320, EPI_ISL_7630324, EPI_ISL_7630338, EPI_ISL_7630381, EPI_ISL_7630413, EPI_ISL_7630432, EPI_ISL_7630438, EPI_ISL_7630464, EPI_ISL_7630528, EPI_ISL_7630557, EPI_ISL_7630599, EPI_ISL_7630626, EPI_ISL_7630652, EPI_ISL_7630661, EPI_ISL_7630693, EPI_ISL_7630702, EPI_ISL_7630727, EPI_ISL_7631177, EPI_ISL_7631374, EPI_ISL_7631420, EPI_ISL_7631438, EPI_ISL_7631459, EPI_ISL_7631599, EPI_ISL_7631618, EPI_ISL_7631728, EPI_ISL_7631904, EPI_ISL_7632067, EPI_ISL_7632092, EPI_ISL_7632160, EPI_ISL_7632168, EPI_ISL_7632318, EPI_ISL_7632335, EPI_ISL_7632471, EPI_ISL_7632553, EPI_ISL_7632572, EPI_ISL_7632603, EPI_ISL_7632604, EPI_ISL_7632613, EPI_ISL_7632655, EPI_ISL_7632690, EPI_ISL_7633109, EPI_ISL_7633127, EPI_ISL_7633138, EPI_ISL_7633227, EPI_ISL_7633252, EPI_ISL_7633276, EPI_ISL_7633321, EPI_ISL_7633528, EPI_ISL_7633653, EPI_ISL_7633655, EPI_ISL_7633676, EPI_ISL_7633702, EPI_ISL_7633722, EPI_ISL_7634899, EPI_ISL_7634958, EPI_ISL_7634967, EPI_ISL_7634976, EPI_ISL_7634991, EPI_ISL_7635014, EPI_ISL_7635032, EPI_ISL_7635055, EPI_ISL_7635110, EPI_ISL_7635183, EPI_ISL_7635196, EPI_ISL_7635283, EPI_ISL_7635319, EPI_ISL_7635341, EPI_ISL_7635403, EPI_ISL_7635473, EPI_ISL_7635581, EPI_ISL_7635641, EPI_ISL_7635681, EPI_ISL_7635744, EPI_ISL_7635799			
see above	Lighthouse Lab in Glasgow	Wellcome Sanger Institute for the COVID-19 Genomics UK (COG-UK) Consortium	Anna Dominiczak and Alex Alderton; Carol Clugston; Cordelia Langford; David Gray; David K. Jackson; Dominic Kwiatkowski; Ewan Harrison; Harper VanSteenhouse; Ian Johnston; Jeffrey Barrett; John Sillitoe on behalf of the Wellcome Sanger Institute COVID-19 Surveillance Team; Roberto Amato; Sonia Goncalves; Yumi Kasai
EPI_ISL_6818284, EPI_ISL_6833884, EPI_ISL_6834467, EPI_ISL_6915964, EPI_ISL_6919027, EPI_ISL_6920616, EPI_ISL_6923186, EPI_ISL_7022755, EPI_ISL_7023187, EPI_ISL_7023265, EPI_ISL_7023667, EPI_ISL_7023675, EPI_ISL_7023683, EPI_ISL_7023726, EPI_ISL_7023789, EPI_ISL_7023867, EPI_ISL_7024334, EPI_ISL_7024438, EPI_ISL_7024495, EPI_ISL_7024659, EPI_ISL_7024687, EPI_ISL_7024839, EPI_ISL_7024991, EPI_ISL_7025373, EPI_ISL_7026085, EPI_ISL_7026396, EPI_ISL_7026536, EPI_ISL_7026616, EPI_ISL_7026695, EPI_ISL_7026716, EPI_ISL_7026777, EPI_ISL_7026952, EPI_ISL_7028268, EPI_ISL_7028487, EPI_ISL_7029353, EPI_ISL_7033208, EPI_ISL_7037264, EPI_ISL_7143464, EPI_ISL_7146338, EPI_ISL_7146517, EPI_ISL_7146524, EPI_ISL_7146933, EPI_ISL_7147199, EPI_ISL_7157375, EPI_ISL_7157554, EPI_ISL_7181721, EPI_ISL_7182128, EPI_ISL_7182133, EPI_ISL_7182156, EPI_ISL_7185699, EPI_ISL_7185720, EPI_ISL_7185763, EPI_ISL_7185784, EPI_ISL_7185838, EPI_ISL_7185864, EPI_ISL_7185866, EPI_ISL_7187248, EPI_ISL_7187503, EPI_ISL_7188445, EPI_ISL_7188816, EPI_ISL_7188897, EPI_ISL_7188957, EPI_ISL_7189046, EPI_ISL_7189048, EPI_ISL_7189067, EPI_ISL_7189073, EPI_ISL_7196773, EPI_ISL_7197261, EPI_ISL_7197402, EPI_ISL_7197454, EPI_ISL_7198739, EPI_ISL_7198774, EPI_ISL_7198902, EPI_ISL_7199159, EPI_ISL_7202939, EPI_ISL_7202951, EPI_ISL_7202968, EPI_ISL_7202977, EPI_ISL_7202998, EPI_ISL_7203007, EPI_ISL_7203016, EPI_ISL_7203017, EPI_ISL_7203032, EPI_ISL_7203085, EPI_ISL_7203096, EPI_ISL_7203100, EPI_ISL_7203105, EPI_ISL_7203132, EPI_ISL_7203159, EPI_ISL_7203161, EPI_ISL_7203182, EPI_ISL_7203185, EPI_ISL_7203202, EPI_ISL_7203249, EPI_ISL_7203710, EPI_ISL_7203762, EPI_ISL_7203765, EPI_ISL_7203936, EPI_ISL_7204224, EPI_ISL_7293149, EPI_ISL_7293182, EPI_ISL_7293573, EPI_ISL_7293699, EPI_ISL_7293701, EPI_ISL_7293733, EPI_ISL_7293785, EPI_ISL_7296211, EPI_ISL_7297545, EPI_ISL_7297579, EPI_ISL_7300159, EPI_ISL_7300279, EPI_ISL_7300286, EPI_ISL_7300537, EPI_ISL_7300658, EPI_ISL_7300878, EPI_ISL_7300889, EPI_ISL_7300915, EPI_ISL_7300986, EPI_ISL_7301005, EPI_ISL_7301013, EPI_ISL_7301015, EPI_ISL_7301165, EPI_ISL_7301209, EPI_ISL_7301266, EPI_ISL_7301307, EPI_ISL_7301521, EPI_ISL_7301561, EPI_ISL_7301876, EPI_ISL_7304476, EPI_ISL_7304564, EPI_ISL_7304690, EPI_ISL_7304994, EPI_ISL_7305027, EPI_ISL_7305176, EPI_ISL_7340046, EPI_ISL_7340118, EPI_ISL_7340964, EPI_ISL_7341048, EPI_ISL_7344931, EPI_ISL_7345012, EPI_ISL_7345138, EPI_ISL_7345164, EPI_ISL_7346326, EPI_ISL_7346393, EPI_ISL_7346410, EPI_ISL_7346479, EPI_ISL_7346521, EPI_ISL_7346666, EPI_ISL_7346704, EPI_ISL_7346758, EPI_ISL_7346809, EPI_ISL_7346816, EPI_ISL_7346835, EPI_ISL_7347737, EPI_ISL_7347952, EPI_ISL_7347992, EPI_ISL_7349648, EPI_ISL_7349666, EPI_ISL_7349764, EPI_ISL_7349797, EPI_ISL_7349804, EPI_ISL_7350042, EPI_ISL_7350201, EPI_ISL_7350318, EPI_ISL_7350332, EPI_ISL_7350365, EPI_ISL_7350520, EPI_ISL_7350526, EPI_ISL_7350556, EPI_ISL_7350583, EPI_ISL_7353443, EPI_ISL_7353487, EPI_ISL_7353795, EPI_ISL_7353847, EPI_ISL_7353853, EPI_ISL_7353856, EPI_ISL_7353981, EPI_ISL_7354003, EPI_ISL_7354110, EPI_ISL_7354518, EPI_ISL_7354594, EPI_ISL_7354762, EPI_ISL_7354795, EPI_ISL_7354941, EPI_ISL_7354957, EPI_ISL_7354991, EPI_ISL_7355006, EPI_ISL_7355123, EPI_ISL_7381834, EPI_ISL_7381856, EPI_ISL_7381881, EPI_ISL_7382005, EPI_ISL_7382097, EPI_ISL_7382103, EPI_ISL_7382156, EPI_ISL_7382200, EPI_ISL_7382215, EPI_ISL_7382254, EPI_ISL_7382416, EPI_ISL_7383376, EPI_ISL_7383676, EPI_ISL_7383683, EPI_ISL_7383685, EPI_ISL_7383740, EPI_ISL_7383773, EPI_ISL_7383781, EPI_ISL_7383783, EPI_ISL_7383798, EPI_ISL_7383852, EPI_ISL_7383856, EPI_ISL_7383863, EPI_ISL_7383885, EPI_ISL_7383921, EPI_ISL_7383922, EPI_ISL_7383936, EPI_ISL_7383941, EPI_ISL_7383945, EPI_ISL_7383954, EPI_ISL_7383977, EPI_ISL_7383978, EPI_ISL_7383980, EPI_ISL_7384190, EPI_ISL_7384545, EPI_ISL_7384619, EPI_ISL_7385445, EPI_ISL_7385606, EPI_ISL_7386026, EPI_ISL_7386052, EPI_ISL_7386325, EPI_ISL_7386397, EPI_ISL_7390306, EPI_ISL_7390618, EPI_ISL_7391659, EPI_ISL_7391668, EPI_ISL_7391823, EPI_ISL_7393627, EPI_ISL_7393737, EPI_ISL_7393800, EPI_ISL_7393853, EPI_ISL_7393873, EPI_ISL_7395502, EPI_ISL_7395791, EPI_ISL_7395797, EPI_ISL_7395849, EPI_ISL_7395905, EPI_ISL_7396016, EPI_ISL_7396038, EPI_ISL_7396042, EPI_ISL_7396056, EPI_ISL_7396060, EPI_ISL_7396114, EPI_ISL_7396120, EPI_ISL_7396167, EPI_ISL_7396207, EPI_ISL_7396238, EPI_ISL_7396262, EPI_ISL_7396271, EPI_ISL_7396409, EPI_ISL_7396415, EPI_ISL_7396446, EPI_ISL_7396483, EPI_ISL_7396525, EPI_ISL_7396533, EPI_ISL_7396579, EPI_ISL_7396628, EPI_ISL_7396640, EPI_ISL_7396987, EPI_ISL_7398089, EPI_ISL_7398228, EPI_ISL_7398295, EPI_ISL_7398393, EPI_ISL_7478708, EPI_ISL_7478734, EPI_ISL_7478799, EPI_ISL_7478827, EPI_ISL_7479102, EPI_ISL_7479225, EPI_ISL_7479311, EPI_ISL_7479350, EPI_ISL_7479729, EPI_ISL_7480249, EPI_ISL_7480340, EPI_ISL_7480459, EPI_ISL_7480563, EPI_ISL_7480779, EPI_ISL_7480983, EPI_ISL_7481103, EPI_ISL_7481888, EPI_ISL_7483648, EPI_ISL_7483732, EPI_ISL_7483778, EPI_ISL_7483853, EPI_ISL_7484047, EPI_ISL_7484394, EPI_ISL_7484568, EPI_ISL_7484575, EPI_ISL_7484580, EPI_ISL_7484583, EPI_ISL_7484584, EPI_ISL_7484632, EPI_ISL_7484936, EPI_ISL_7485137, EPI_ISL_7485159, EPI_ISL_7485300, EPI_ISL_7485391, EPI_ISL_7485734, EPI_ISL_7485763, EPI_ISL_7485880, EPI_ISL_7485890, EPI_ISL_7485899, EPI_ISL_7485918, EPI_ISL_7486077, EPI_ISL_7486120, EPI_ISL_7486163, EPI_ISL_7486373, EPI_ISL_7486447, EPI_ISL_7486481, EPI_ISL_7486517, EPI_ISL_7486635, EPI_ISL_7486819, EPI_ISL_7486971, EPI_ISL_7487426, EPI_ISL_7487488, EPI_ISL_7487790, EPI_ISL_7487954, EPI_ISL_7488141, EPI_ISL_7488200, EPI_ISL_7488279, EPI_ISL_7489187, EPI_ISL_7489326, EPI_ISL_7489389, EPI_ISL_7489446, EPI_ISL_7489786, EPI_ISL_7489819, EPI_ISL_7489953, EPI_ISL_7489980, EPI_ISL_7490259, EPI_ISL_7490487, EPI_ISL_7490584, EPI_ISL_7490883, EPI_ISL_7490957, EPI_ISL_7509888, EPI_ISL_7509901, EPI_ISL_7509939, EPI_ISL_7510081, EPI_ISL_7510216, EPI_ISL_7510241, EPI_ISL_7511995, EPI_ISL_7512017, EPI_ISL_7512047, EPI_ISL_7512058, EPI_ISL_7512077, EPI_ISL_7512119, EPI_ISL_7512155, EPI_ISL_7512175, EPI_ISL_7512265, EPI_ISL_7512279, EPI_ISL_7512284, EPI_ISL_7512302, EPI_ISL_7512321, EPI_ISL_7512475, EPI_ISL_7512549, EPI_ISL_7512707, EPI_ISL_7512966, EPI_ISL_7513072, EPI_ISL_7513131, EPI_ISL_7513234, EPI_ISL_7514854, EPI_ISL_7515519, EPI_ISL_7515566, EPI_ISL_7515634, EPI_ISL_7515973, EPI_ISL_7516148, EPI_ISL_7517004, EPI_ISL_7519115, EPI_ISL_7520174, EPI_ISL_7520334, EPI_ISL_7520362, EPI_ISL_7520998, EPI_ISL_7522351, EPI_ISL_7522461, EPI_ISL_7523110, EPI_ISL_7523203, EPI_ISL_7523364, EPI_ISL_7523595, EPI_ISL_7524106, EPI_ISL_7524864, EPI_ISL_7525422, EPI_ISL_7526186, EPI_ISL_7526214, EPI_ISL_7526231, EPI_ISL_7526276, EPI_ISL_7526372, EPI_ISL_7526379, EPI_ISL_7526426, EPI_ISL_7526472, EPI_ISL_7526476, EPI_ISL_7526571, EPI_ISL_7526583, EPI_ISL_7526592, EPI_ISL_7526597, EPI_ISL_7526604, EPI_ISL_7526609, EPI_ISL_7526665, EPI_ISL_7526672, EPI_ISL_7526690, EPI_ISL_7526766, EPI_ISL_7526796, EPI_ISL_7526806, EPI_ISL_7526823, EPI_ISL_7526841, EPI_ISL_7526878, EPI_ISL_7526961, EPI_ISL_7526985, EPI_ISL_7526997, EPI_ISL_7527046, EPI_ISL_7527101, EPI_ISL_7527267, EPI_ISL_7527283, EPI_ISL_7527298, EPI_ISL_7527348, EPI_ISL_7527381, EPI_ISL_7527452, EPI_ISL_7528184, EPI_ISL_7528477, EPI_ISL_7528569, EPI_ISL_7528699, EPI_ISL_7528999, EPI_ISL_7529344, EPI_ISL_7529479, EPI_ISL_7529568, EPI_ISL_7529705, EPI_ISL_7529714, EPI_ISL_7529749, EPI_ISL_7529754, EPI_ISL_7530006, EPI_ISL_7530158, EPI_ISL_7530274, EPI_ISL_7530290, EPI_ISL_7530610, EPI_ISL_7532349, EPI_ISL_7532487, EPI_ISL_7532636, EPI_ISL_7532667, EPI_ISL_7533126, EPI_ISL_7533558, EPI_ISL_7533573, EPI_ISL_7535208, EPI_ISL_7535261, EPI_ISL_7535308, EPI_ISL_7535330, EPI_ISL_7535331, EPI_ISL_7535381, EPI_ISL_7535476, EPI_ISL_7535510, EPI_ISL_7535600, EPI_ISL_7535650, EPI_ISL_7536373, EPI_ISL_7536571, EPI_ISL_7536608, EPI_ISL_7536664, EPI_ISL_7536712, EPI_ISL_7536778, EPI_ISL_7536955, EPI_ISL_7537157, EPI_ISL_7537194, EPI_ISL_7537382, EPI_ISL_7537419, EPI_ISL_7537698, EPI_ISL_7537754, EPI_ISL_7537774, EPI_ISL_7537864, EPI_ISL_7538259, EPI_ISL_7538311, EPI_ISL_7538654, EPI_ISL_7538855, EPI_ISL_7539129, EPI_ISL_7539442, EPI_ISL_7541246, EPI_ISL_7541250, EPI_ISL_7541383, EPI_ISL_7541410, EPI_ISL_7541455, EPI_ISL_7541551, EPI_ISL_7541700, EPI_ISL_7541743, EPI_ISL_7541804, EPI_ISL_7541826, EPI_ISL_7541859, EPI_ISL_7541865, EPI_ISL_7541886, EPI_ISL_7541892, EPI_ISL_7541941, EPI_ISL_7541961, EPI_ISL_7541994, EPI_ISL_7553144, EPI_ISL_7553149, EPI_ISL_7553161, EPI_ISL_7553192, EPI_ISL_7553197, EPI_ISL_7553204, EPI_ISL_7553212, EPI_ISL_7553248, EPI_ISL_7553279, EPI_ISL_7553287, EPI_ISL_7553310, EPI_ISL_7553311, EPI_ISL_7553332, EPI_ISL_7553333, EPI_ISL_7553339, EPI_ISL_7553392, EPI_ISL_7553477, EPI_ISL_7553485, EPI_ISL_7553491, EPI_ISL_7553529, EPI_ISL_7553567, EPI_ISL_7553579, EPI_ISL_7553585, EPI_ISL_7553618, EPI_ISL_7553651, EPI_ISL_7553666, EPI_ISL_7553670, EPI_ISL_7553711, EPI_ISL_7553748, EPI_ISL_7553762, EPI_ISL_7553833, EPI_ISL_7553873, EPI_ISL_7553888, EPI_ISL_7553903, EPI_ISL_7553907, EPI_ISL_7553927, EPI_ISL_7553934, EPI_ISL_7553963, EPI_ISL_7553964, EPI_ISL_7553983, EPI_ISL_7553987, EPI_ISL_7554005, EPI_ISL_7554013, EPI_ISL_7554019, EPI_ISL_7554096, EPI_ISL_7554137, EPI_ISL_7554171, EPI_ISL_7554248, EPI_ISL_7554281, EPI_ISL_7554321, EPI_ISL_7554346, EPI_ISL_7554445, EPI_ISL_7554476, EPI_ISL_7554489, EPI_ISL_7554490, EPI_ISL_7554516, EPI_ISL_7554550, EPI_ISL_7554560, EPI_ISL_7554680, EPI_ISL_7554690, EPI_ISL_7554699, EPI_ISL_7554703, EPI_ISL_7554783, EPI_ISL_7554850, EPI_ISL_7554872, EPI_ISL_7554949, EPI_ISL_7554973, EPI_ISL_7554995, EPI_ISL_7555045, EPI_ISL_7555072, EPI_ISL_7555086, EPI_ISL_7555141, EPI_ISL_7555150, EPI_ISL_7555163, EPI_ISL_7555176, EPI_ISL_7555186, EPI_ISL_7555190, EPI_ISL_7555191, EPI_ISL_7555195, EPI_ISL_7555212, EPI_ISL_7555216, EPI_ISL_7555273, EPI_ISL_7555291, EPI_ISL_7555306, EPI_ISL_7555380, EPI_ISL_7555396, EPI_ISL_7555470, EPI_ISL_7555477, EPI_ISL_7555530, EPI_ISL_7555535, EPI_ISL_7555659, EPI_ISL_7555700, EPI_ISL_7555721, EPI_ISL_7555744, EPI_ISL_7555758, EPI_ISL_7555774, EPI_ISL_7555781, EPI_ISL_7555793, EPI_ISL_7555851, EPI_ISL_7555885, EPI_ISL_7555991, EPI_ISL_7556003, EPI_ISL_7556033, EPI_ISL_7556161, EPI_ISL_7556164, EPI_ISL_7556207, EPI_ISL_7556299, EPI_ISL_7556333, EPI_ISL_7556340, EPI_ISL_7556345, EPI_ISL_7556347, EPI_ISL_7556440, EPI_ISL_7556446, EPI_ISL_7556454, EPI_ISL_7556458, EPI_ISL_7556547, EPI_ISL_7556562, EPI_ISL_7556583, EPI_ISL_7556612, EPI_ISL_7556832, EPI_ISL_7556878, EPI_ISL_7556879, EPI_ISL_7556883, EPI_ISL_7556891, EPI_ISL_7556899, EPI_ISL_7556905, EPI_ISL_7556979, EPI_ISL_7557010, EPI_ISL_7557022, EPI_ISL_7557048, EPI_ISL_7557104, EPI_ISL_7557126, EPI_ISL_7557152, EPI_ISL_7557167, EPI_ISL_7557302, EPI_ISL_7557304, EPI_ISL_7557331, EPI_ISL_7557391, EPI_ISL_7557422, EPI_ISL_7557463, EPI_ISL_7557476, EPI_ISL_7557485, EPI_ISL_7557490, EPI_ISL_7557510, EPI_ISL_7557520, EPI_ISL_7557522, EPI_ISL_7557536, EPI_ISL_7557542, EPI_ISL_7557546, EPI_ISL_7557555, EPI_ISL_7557558, EPI_ISL_7557594, EPI_ISL_7557630, EPI_ISL_7557742, EPI_ISL_7557755, EPI_ISL_7557757, EPI_ISL_7557788, EPI_ISL_7557793, EPI_ISL_7557821, EPI_ISL_7557823, EPI_ISL_7557844, EPI_ISL_7557974, EPI_ISL_7558442, EPI_ISL_7558468, EPI_ISL_7558493, EPI_ISL_7558519, EPI_ISL_7558533, EPI_ISL_7558555, EPI_ISL_7558619, EPI_ISL_7558638, EPI_ISL_7558693, EPI_ISL_7558721, EPI_ISL_7558737, EPI_ISL_7558768, EPI_ISL_7558795, EPI_ISL_7558844, EPI_ISL_7558848, EPI_ISL_7558852, EPI_ISL_7558893, EPI_ISL_7558908, EPI_ISL_7558926, EPI_ISL_7558929, EPI_ISL_7558973, EPI_ISL_7558997, EPI_ISL_7559064, EPI_ISL_7559105, EPI_ISL_7559107,			

EPI_ISL_7559111, EPI_ISL_7559169, EPI_ISL_7559234, EPI_ISL_7559254, EPI_ISL_7559289, EPI_ISL_7559301, EPI_ISL_7559319, EPI_ISL_7559331, EPI_ISL_7559333, EPI_ISL_7559345, EPI_ISL_7559382, EPI_ISL_7559401, EPI_ISL_7559415, EPI_ISL_7559416, EPI_ISL_7559426, EPI_ISL_7559448, EPI_ISL_7559467, EPI_ISL_7559481, EPI_ISL_7559482, EPI_ISL_7559516, EPI_ISL_7559535, EPI_ISL_7559692, EPI_ISL_7559715, EPI_ISL_7559728, EPI_ISL_7559782, EPI_ISL_7559931, EPI_ISL_7559966, EPI_ISL_7559980, EPI_ISL_7559987, EPI_ISL_7560016, EPI_ISL_7560039, EPI_ISL_7560044, EPI_ISL_7560085, EPI_ISL_7560095, EPI_ISL_7560099, EPI_ISL_7560109, EPI_ISL_7560111, EPI_ISL_7560125, EPI_ISL_7560131, EPI_ISL_7560137, EPI_ISL_7560156, EPI_ISL_7560160, EPI_ISL_7560165, EPI_ISL_7560175, EPI_ISL_7560179, EPI_ISL_7560191, EPI_ISL_7560192, EPI_ISL_7560194, EPI_ISL_7560211, EPI_ISL_7560228, EPI_ISL_7560245, EPI_ISL_7560247, EPI_ISL_7560254, EPI_ISL_7560269, EPI_ISL_7560284, EPI_ISL_7560291, EPI_ISL_7560293, EPI_ISL_7560295, EPI_ISL_7560300, EPI_ISL_7560324, EPI_ISL_7560333, EPI_ISL_7560337, EPI_ISL_7560345, EPI_ISL_7560354, EPI_ISL_7560355, EPI_ISL_7560384, EPI_ISL_7560389, EPI_ISL_7560390, EPI_ISL_7560393, EPI_ISL_7560466, EPI_ISL_7560520, EPI_ISL_7560584, EPI_ISL_7560632, EPI_ISL_7560648, EPI_ISL_7560715, EPI_ISL_7560752, EPI_ISL_7560781, EPI_ISL_7560803, EPI_ISL_7560823, EPI_ISL_7560833, EPI_ISL_7560854, EPI_ISL_7560904, EPI_ISL_7560952, EPI_ISL_7561012, EPI_ISL_7561019, EPI_ISL_7561026, EPI_ISL_7561097, EPI_ISL_7561162, EPI_ISL_7561163, EPI_ISL_7561172, EPI_ISL_7571908, EPI_ISL_7571910, EPI_ISL_7571950, EPI_ISL_7571953, EPI_ISL_7571956, EPI_ISL_7571957, EPI_ISL_7571962, EPI_ISL_7571998, EPI_ISL_7572132, EPI_ISL_7572153, EPI_ISL_7572163, EPI_ISL_7572209, EPI_ISL_7572219, EPI_ISL_7572256, EPI_ISL_7572292, EPI_ISL_7572329, EPI_ISL_7572355, EPI_ISL_7572415, EPI_ISL_7572449, EPI_ISL_7572473, EPI_ISL_7572483, EPI_ISL_7572501, EPI_ISL_7572515, EPI_ISL_7572541, EPI_ISL_7572576, EPI_ISL_7572591, EPI_ISL_7572602, EPI_ISL_7572611, EPI_ISL_7572628, EPI_ISL_7572647, EPI_ISL_7572689, EPI_ISL_7572720, EPI_ISL_7572723, EPI_ISL_7572728, EPI_ISL_7572765, EPI_ISL_7572792, EPI_ISL_7572798, EPI_ISL_7572819, EPI_ISL_7572820, EPI_ISL_7572821, EPI_ISL_7572823, EPI_ISL_7572825, EPI_ISL_7572837, EPI_ISL_7572875, EPI_ISL_7574310, EPI_ISL_7574318, EPI_ISL_7574331, EPI_ISL_7574345, EPI_ISL_7574378, EPI_ISL_7574414, EPI_ISL_7574420, EPI_ISL_7574428, EPI_ISL_7574444, EPI_ISL_7574460, EPI_ISL_7574469, EPI_ISL_7574473, EPI_ISL_7574479, EPI_ISL_7574506, EPI_ISL_7574536, EPI_ISL_7574540, EPI_ISL_7574545, EPI_ISL_7574550, EPI_ISL_7574553, EPI_ISL_7574557, EPI_ISL_7574580, EPI_ISL_7574583, EPI_ISL_7574596, EPI_ISL_7574608, EPI_ISL_7574609, EPI_ISL_7574624, EPI_ISL_7574638, EPI_ISL_7574680, EPI_ISL_7574685, EPI_ISL_7574697, EPI_ISL_7574701, EPI_ISL_7574713, EPI_ISL_7574714, EPI_ISL_7574715, EPI_ISL_7574726, EPI_ISL_7574737, EPI_ISL_7574753, EPI_ISL_7574761, EPI_ISL_7574770, EPI_ISL_7574779, EPI_ISL_7574787, EPI_ISL_7574850, EPI_ISL_7574857, EPI_ISL_7574872, EPI_ISL_7574875, EPI_ISL_7574929, EPI_ISL_7574938, EPI_ISL_7574953, EPI_ISL_7574957, EPI_ISL_7574965, EPI_ISL_7574967, EPI_ISL_7574975, EPI_ISL_7575010, EPI_ISL_7575029, EPI_ISL_7575093, EPI_ISL_7575243, EPI_ISL_7575433, EPI_ISL_7575537, EPI_ISL_7575747, EPI_ISL_7575779, EPI_ISL_7576056, EPI_ISL_7576084, EPI_ISL_7576236, EPI_ISL_7576420, EPI_ISL_7576528, EPI_ISL_7576627, EPI_ISL_7576647, EPI_ISL_7576653, EPI_ISL_7576689, EPI_ISL_7576697, EPI_ISL_7576759, EPI_ISL_7576788, EPI_ISL_7576795, EPI_ISL_7576855, EPI_ISL_7576879, EPI_ISL_7576933, EPI_ISL_7576976, EPI_ISL_7577022, EPI_ISL_7577089, EPI_ISL_7577095, EPI_ISL_7577133, EPI_ISL_7577141, EPI_ISL_7577182, EPI_ISL_7577201, EPI_ISL_7577217, EPI_ISL_7577244, EPI_ISL_7577302, EPI_ISL_7577306, EPI_ISL_7577352, EPI_ISL_7577387, EPI_ISL_7577409, EPI_ISL_7577420, EPI_ISL_7577462, EPI_ISL_7577527, EPI_ISL_7577528, EPI_ISL_7577743, EPI_ISL_7577877, EPI_ISL_7577953, EPI_ISL_7577956, EPI_ISL_7577964, EPI_ISL_7577972, EPI_ISL_7577974, EPI_ISL_7577994, EPI_ISL_7578143, EPI_ISL_7578256, EPI_ISL_7578292, EPI_ISL_7578340, EPI_ISL_7578353, EPI_ISL_7578459, EPI_ISL_7578518, EPI_ISL_7578663, EPI_ISL_7578729, EPI_ISL_7579103, EPI_ISL_7579594, EPI_ISL_7579656, EPI_ISL_7579923, EPI_ISL_7579925, EPI_ISL_7579947, EPI_ISL_7579965, EPI_ISL_7579967, EPI_ISL_7579991, EPI_ISL_7579992, EPI_ISL_7580021, EPI_ISL_7580025, EPI_ISL_7580029, EPI_ISL_7580037, EPI_ISL_7580044, EPI_ISL_7580054, EPI_ISL_7580104, EPI_ISL_7580109, EPI_ISL_7580116, EPI_ISL_7580135, EPI_ISL_7580145, EPI_ISL_7580170, EPI_ISL_7580180, EPI_ISL_7580205, EPI_ISL_7580335, EPI_ISL_7580387, EPI_ISL_7580388, EPI_ISL_7580420, EPI_ISL_7580447, EPI_ISL_7580448, EPI_ISL_7580476, EPI_ISL_7580535, EPI_ISL_7580548, EPI_ISL_7580555, EPI_ISL_7580568, EPI_ISL_7580599, EPI_ISL_7580623, EPI_ISL_7580637, EPI_ISL_7580682, EPI_ISL_7580705, EPI_ISL_7580729, EPI_ISL_7580732, EPI_ISL_7580843, EPI_ISL_7580850, EPI_ISL_7580851, EPI_ISL_7580869, EPI_ISL_7580876, EPI_ISL_7580886, EPI_ISL_7580965, EPI_ISL_7580997, EPI_ISL_7581008, EPI_ISL_7581047, EPI_ISL_7581088, EPI_ISL_7581089, EPI_ISL_7581099, EPI_ISL_7581114, EPI_ISL_7581163, EPI_ISL_7581192, EPI_ISL_7581321, EPI_ISL_7581351, EPI_ISL_7581399, EPI_ISL_7581409, EPI_ISL_7581445, EPI_ISL_7581525, EPI_ISL_7581571, EPI_ISL_7581625, EPI_ISL_7581641, EPI_ISL_7581662, EPI_ISL_7581663, EPI_ISL_7581676, EPI_ISL_7581768, EPI_ISL_7581809, EPI_ISL_7582242, EPI_ISL_7582510, EPI_ISL_7582511, EPI_ISL_7582572, EPI_ISL_7582651, EPI_ISL_7582661, EPI_ISL_7582674, EPI_ISL_7582788, EPI_ISL_7582935, EPI_ISL_7583017, EPI_ISL_7583130, EPI_ISL_7583138, EPI_ISL_7583151, EPI_ISL_7583185, EPI_ISL_7583206, EPI_ISL_7583284, EPI_ISL_7583310, EPI_ISL_7583376, EPI_ISL_7583481, EPI_ISL_7583574, EPI_ISL_7583607, EPI_ISL_7583696, EPI_ISL_7583748, EPI_ISL_7583830, EPI_ISL_7583878, EPI_ISL_7583942, EPI_ISL_7584036, EPI_ISL_7584057, EPI_ISL_7584077, EPI_ISL_7584766, EPI_ISL_7584869, EPI_ISL_7584965, EPI_ISL_7585008, EPI_ISL_7585036, EPI_ISL_7585211, EPI_ISL_7585238, EPI_ISL_7586597, EPI_ISL_7586606, EPI_ISL_7586607, EPI_ISL_7586608, EPI_ISL_7586789, EPI_ISL_7586796, EPI_ISL_7586807, EPI_ISL_7586819, EPI_ISL_7586841, EPI_ISL_7586844, EPI_ISL_7586854, EPI_ISL_7586865, EPI_ISL_7586900, EPI_ISL_7586911, EPI_ISL_7586933, EPI_ISL_7586943, EPI_ISL_7587053, EPI_ISL_7587071, EPI_ISL_7587072, EPI_ISL_7587082, EPI_ISL_7587159, EPI_ISL_7587216, EPI_ISL_7587225, EPI_ISL_7587236, EPI_ISL_7587306, EPI_ISL_7587349, EPI_ISL_7587389, EPI_ISL_7587438, EPI_ISL_7587459, EPI_ISL_7587484, EPI_ISL_7587601, EPI_ISL_7587618, EPI_ISL_7587700, EPI_ISL_7587772, EPI_ISL_7587790, EPI_ISL_7587791, EPI_ISL_7587834, EPI_ISL_7587872, EPI_ISL_7587936, EPI_ISL_7587950, EPI_ISL_7588011, EPI_ISL_7624460, EPI_ISL_7624566, EPI_ISL_7624618, EPI_ISL_7624627, EPI_ISL_7624684, EPI_ISL_7624691, EPI_ISL_7624699, EPI_ISL_7624754, EPI_ISL_7624783, EPI_ISL_7624802, EPI_ISL_7624813, EPI_ISL_7624829, EPI_ISL_7624840, EPI_ISL_7624918, EPI_ISL_7624952, EPI_ISL_7625001, EPI_ISL_7625005, EPI_ISL_7625025, EPI_ISL_7625079, EPI_ISL_7625096, EPI_ISL_7625150, EPI_ISL_7625171, EPI_ISL_7625192, EPI_ISL_7625311, EPI_ISL_7625376, EPI_ISL_7625386, EPI_ISL_7625400, EPI_ISL_7625414, EPI_ISL_7625433, EPI_ISL_7625439, EPI_ISL_7625463, EPI_ISL_7625472, EPI_ISL_7625473, EPI_ISL_7625479, EPI_ISL_7625494, EPI_ISL_7625541, EPI_ISL_7625547, EPI_ISL_7625552, EPI_ISL_7625586, EPI_ISL_7625588, EPI_ISL_7625589, EPI_ISL_7625593, EPI_ISL_7625620, EPI_ISL_7625623, EPI_ISL_7625629, EPI_ISL_7625639, EPI_ISL_7625654, EPI_ISL_7625697, EPI_ISL_7625701, EPI_ISL_7625707, EPI_ISL_7625722, EPI_ISL_7625754, EPI_ISL_7625771, EPI_ISL_7626567, EPI_ISL_7626606, EPI_ISL_7626607, EPI_ISL_7626612, EPI_ISL_7626615, EPI_ISL_7626620, EPI_ISL_7626625, EPI_ISL_7626630, EPI_ISL_7626633, EPI_ISL_7626644, EPI_ISL_7626649, EPI_ISL_7626662, EPI_ISL_7626664, EPI_ISL_7626672, EPI_ISL_7626680, EPI_ISL_7626683, EPI_ISL_7626692, EPI_ISL_7626696, EPI_ISL_7626698, EPI_ISL_7626701, EPI_ISL_7626704, EPI_ISL_7626711, EPI_ISL_7626714, EPI_ISL_7626735, EPI_ISL_7626743, EPI_ISL_7626748, EPI_ISL_7626755, EPI_ISL_7626765, EPI_ISL_7626839, EPI_ISL_7626852, EPI_ISL_7626862, EPI_ISL_7626863, EPI_ISL_7626870, EPI_ISL_7626874, EPI_ISL_7626894, EPI_ISL_7626906, EPI_ISL_7626922, EPI_ISL_7626928, EPI_ISL_7626937, EPI_ISL_7626950, EPI_ISL_7626954, EPI_ISL_7626967, EPI_ISL_7626969, EPI_ISL_7626973, EPI_ISL_7626975, EPI_ISL_7626976, EPI_ISL_7626977, EPI_ISL_7626995, EPI_ISL_7626997, EPI_ISL_7627006, EPI_ISL_7627008, EPI_ISL_7627016, EPI_ISL_7627027, EPI_ISL_7627039, EPI_ISL_7627053, EPI_ISL_7627054, EPI_ISL_7627058, EPI_ISL_7627063, EPI_ISL_7627091, EPI_ISL_7627097, EPI_ISL_7627129, EPI_ISL_7627136, EPI_ISL_7627139, EPI_ISL_7627154, EPI_ISL_7627160, EPI_ISL_7627165, EPI_ISL_7627168, EPI_ISL_7627176, EPI_ISL_7627179, EPI_ISL_7627196, EPI_ISL_7627197, EPI_ISL_7627200, EPI_ISL_7627216, EPI_ISL_7627217, EPI_ISL_7627236, EPI_ISL_7629194, EPI_ISL_7629214, EPI_ISL_7629269, EPI_ISL_7629303, EPI_ISL_7629403, EPI_ISL_7629484, EPI_ISL_7629510, EPI_ISL_7629604, EPI_ISL_7629619, EPI_ISL_7629646, EPI_ISL_7629705, EPI_ISL_7629753, EPI_ISL_7629785, EPI_ISL_7629792, EPI_ISL_7629817, EPI_ISL_7629973, EPI_ISL_7629979, EPI_ISL_7630007, EPI_ISL_7630026, EPI_ISL_7630050, EPI_ISL_7630068, EPI_ISL_7630098, EPI_ISL_7630868, EPI_ISL_7630897, EPI_ISL_7630900, EPI_ISL_7630918, EPI_ISL_7630925, EPI_ISL_7630928, EPI_ISL_7631018, EPI_ISL_7631029, EPI_ISL_7631048, EPI_ISL_7631073, EPI_ISL_7631097, EPI_ISL_7631131, EPI_ISL_7631148, EPI_ISL_7631149, EPI_ISL_7631206, EPI_ISL_7631223, EPI_ISL_7631236, EPI_ISL_7631257, EPI_ISL_7631498, EPI_ISL_7631754, EPI_ISL_7632315, EPI_ISL_7632790, EPI_ISL_7632843, EPI_ISL_7632860, EPI_ISL_7632864, EPI_ISL_7632879, EPI_ISL_7632885, EPI_ISL_7632914, EPI_ISL_7632933, EPI_ISL_7632935, EPI_ISL_7632958, EPI_ISL_7632959, EPI_ISL_7632966, EPI_ISL_7632974, EPI_ISL_7632997, EPI_ISL_7633011, EPI_ISL_7633032, EPI_ISL_7633043, EPI_ISL_7633046, EPI_ISL_7633084, EPI_ISL_7633100, EPI_ISL_7633102, EPI_ISL_7633139, EPI_ISL_7633149, EPI_ISL_7633268, EPI_ISL_7633306, EPI_ISL_7633315, EPI_ISL_7633396, EPI_ISL_7633400, EPI_ISL_7633434, EPI_ISL_7633451, EPI_ISL_7633514, EPI_ISL_7633572, EPI_ISL_7633596, EPI_ISL_7633662, EPI_ISL_7633698, EPI_ISL_7633744, EPI_ISL_7633755, EPI_ISL_7633759, EPI_ISL_7633765, EPI_ISL_7633766, EPI_ISL_7633767, EPI_ISL_7633769, EPI_ISL_7633770, EPI_ISL_7633776, EPI_ISL_7633787, EPI_ISL_7633791, EPI_ISL_7633800, EPI_ISL_7633822, EPI_ISL_7633836, EPI_ISL_7633837, EPI_ISL_7633847, EPI_ISL_7633856, EPI_ISL_7633860, EPI_ISL_7633873, EPI_ISL_7633875, EPI_ISL_7633886, EPI_ISL_7633916, EPI_ISL_7633917, EPI_ISL_7633936, EPI_ISL_7633942, EPI_ISL_7633944, EPI_ISL_7633976, EPI_ISL_7633978, EPI_ISL_7633993, EPI_ISL_7634000, EPI_ISL_7634001, EPI_ISL_7634009, EPI_ISL_7634025, EPI_ISL_7634053, EPI_ISL_7634060, EPI_ISL_7634074, EPI_ISL_7634102, EPI_ISL_7634116, EPI_ISL_7634119, EPI_ISL_7634122, EPI_ISL_7634133, EPI_ISL_7634137, EPI_ISL_7634138, EPI_ISL_7634145, EPI_ISL_7634151, EPI_ISL_7634164, EPI_ISL_7634187, EPI_ISL_7634201, EPI_ISL_7634209, EPI_ISL_7634211, EPI_ISL_7634222, EPI_ISL_7634224, EPI_ISL_7634235, EPI_ISL_7634242, EPI_ISL_7634249, EPI_ISL_7634261, EPI_ISL_7634262, EPI_ISL_7634269, EPI_ISL_7634270, EPI_ISL_7634294, EPI_ISL_7634298, EPI_ISL_7634337, EPI_ISL_7634360, EPI_ISL_7634364, EPI_ISL_7634383, EPI_ISL_7634393, EPI_ISL_7634395, EPI_ISL_7634414, EPI_ISL_7634415, EPI_ISL_7634418, EPI_ISL_7634419, EPI_ISL_7634442, EPI_ISL_7634446, EPI_ISL_7634447, EPI_ISL_7634453, EPI_ISL_7634463, EPI_ISL_7634466, EPI_ISL_7634480, EPI_ISL_7634482, EPI_ISL_7634510, EPI_ISL_7634531, EPI_ISL_7634540, EPI_ISL_7634550, EPI_ISL_7634552, EPI_ISL_7634564, EPI_ISL_7634566, EPI_ISL_7634583, EPI_ISL_7634587, EPI_ISL_7634595, EPI_ISL_7634607, EPI_ISL_7634610, EPI_ISL_7634623, EPI_ISL_7634647, EPI_ISL_7634651, EPI_ISL_7634656, EPI_ISL_7634659, EPI_ISL_7634678, EPI_ISL_7634680, EPI_ISL_7634684, EPI_ISL_7634703, EPI_ISL_7634704, EPI_ISL_7634712, EPI_ISL_7634739, EPI_ISL_7634756, EPI_ISL_7634769, EPI_ISL_7634770, EPI_ISL_7634785, EPI_ISL_7634794, EPI_ISL_7634813, EPI_ISL_7634821, EPI_ISL_7634824, EPI_ISL_7634832, EPI_ISL_7634833, EPI_ISL_7634846, EPI_ISL_7634855, EPI_ISL_7634865, EPI_ISL_7634867, EPI_ISL_7634871, EPI_ISL_7634878, EPI_ISL_7635532, EPI_ISL_7635602, EPI_ISL_7635633, EPI_ISL_7635708, EPI_ISL_7635758, EPI_ISL_7635793, EPI_ISL_7635832			
see above	Lighthouse Lab in Milton Keynes	Wellcome Sanger Institute for the COVID-19 Genomics UK (COG-UK) Consortium	Cordelia Langford; David K. Jackson; Dominic Kwiatkowski; Ewan Harrison; Ian Johnston; Jeffrey Barrett; John Sillitoe on behalf of the Wellcome Sanger Institute COVID-19 Surveillance Team; Roberto Amato; Sonia Goncalves; The Lighthouse Lab in Milton Keynes and Alex Alderton
EPI_ISL_7201997, EPI_ISL_7302326, EPI_ISL_7302955, EPI_ISL_7303330, EPI_ISL_7625911, EPI_ISL_7625953, EPI_ISL_7626237, EPI_ISL_7626242, EPI_ISL_7626494, EPI_ISL_7628520, EPI_ISL_7628997, EPI_ISL_7629036, EPI_ISL_7630124, EPI_ISL_7630316, EPI_ISL_7630810			
see above	Lighthouse Laboratory Plymouth	Wellcome Sanger Institute for the COVID-19 Genomics UK (COG-UK) Consortium	Cordelia Langford; David K. Jackson; Dominic Kwiatkowski; Ewan Harrison; Ian Johnston; Jeffrey Barrett; John Sillitoe on behalf of the Wellcome Sanger Institute COVID-19 Surveillance Team; Lighthouse Laboratory Plymouth and Alex Alderton; Roberto Amato; Sonia Goncalves
EPI_ISL_7413422, EPI_ISL_7413427, EPI_ISL_7413439, EPI_ISL_7413450, EPI_ISL_7597006	MB-Cadham Provincial laboratory	National Microbiology Laboratory (NML)	Adam Hedley; Anna Majer; Anneliese Landgraff; Ayo Bolaji; CanCOGeN's metadata curation team; Darian Hole; David Alexander; Elsie Grudeski; Gary Van Domselaar; Grace Seo; Janna Holowick; Jared Bullard; Jennifer Tanner; Joanne Sanders; Kerry Dust; Kirsten Biggar; Lori Johnson; Madison Chapel; Morag Graham; Natalie Knox; Nathalie Bastien; Paul Van Caeseele; Philip Mabon; Public Health Agency of Canada CanCOGeN team; Rhiannon Huzarewich; Russell Mandes; Shari Tyson; Timothy Booth; Yan Li
EPI_ISL_7405692	MCL Medizinische Laboratorien, Hauptstandort Niederwangen	Institute for Infectious Diseases, University of Bern	Alban Ramette; Christian Baumann; Cora Sägesser; Franziska Suter-Riniker; Loïc Borcard; Miguel A Terrazos Miani; Nicole Liechti; Pascal Bittel; Peter Keller; Sonja Gempeler; Stefan Neuenschwander; Stephen L Leib
EPI_ISL_7608613, EPI_ISL_7609854	MIRIALIS CLUSES BECHET	CNR Virus des Infections Respiratoires - France SUD	Antonin Bal; Bruno Lina; Bruno Simon; Gregory Destras; Gwendolyne Burfin; Hadrien Regue; Laurence Josset; Martine Valette; Quentin Semanas
EPI_ISL_7615139	MS PHL	MS PHL	Beata Karowlewicz; Daphne Ware
EPI_ISL_7444012, EPI_ISL_7444038, EPI_ISL_7444057	MVZ Labor Dr. Fenner und Kollegen (Standort Hamburg)	Robert Koch Institute	
EPI_ISL_7418337	MVZ Labor Dr. Limbach & Kollegen GbR	Robert Koch Institute	
EPI_ISL_7542261	MVZ Rheumatologie und Autoimmunmedizin Hamburg GmbH	Heinrich Pette Institute, Leibniz Institute for Experimental Virology	Adam Grundhoff; Alexis Robitaille; Johannes Knobloch; Martin Aepfelbacher; Nicole Fischer; Thomas Günther
EPI_ISL_7620047	Makeni Molecular Laboratory	Central Public Health Reference Laboratory	Allan K.O. Campbell; Anu J. Williams; Choe Miller; Doris Harding; Dr. Raoul E.G. Wadoum; Prof. Foday Sahr; Prof. Isatta Wurie; Prof. Sahr Gevao; Samuel Sorie; Warren C. Yiaba; Zein Souma
EPI_ISL_7154400, EPI_ISL_7154401, EPI_ISL_7154402, EPI_ISL_7500444	Mako Medical	Centers for Disease Control and Prevention Division of Viral Diseases, Pathogen Discovery	Benjamin Rambo-Martin; Christopher Gulvick; Clinton Paden; Dakota Howard; Dhwani Batra; Duncan MacCannell; Erisa Sula; Jason Caravas; Kristine Lacek; Lauren Moon; Matthew Schmerer; Matthew Tugwell; Peter Cook; Scott Sammons; Shatavia Morrison; Tymeckia Kendall; Victoria Caban Figueroa; Yvette Unoarumhi
EPI_ISL_7464406, EPI_ISL_7464407, EPI_ISL_7464408	Malawi Liverpool Wellcome Trust Clinical Research Program	Malawi Liverpool Wellcome Trust Clinical Research Program	Belson Kutambe; Ben Morton; Catherine Anscombe; Kondwani Jambo; Mavis Menyere; Philip Ashton; Sam Lissauer
EPI_ISL_7404794	Maryland Genomics, Institute for Genome Sciences, University of Maryland School of Medicine	Maryland Genomics, Institute for Genome Sciences, University of Maryland School of Medicine	Claire M; Fraser; George; Hazen; Holly; Humphrys; Jacques; Jain; Kevin; Kranthi; Lisa D; Luke J; Mike; Ott; Ravel; Regan; Roussey; Sadzewicz; Sandra; Tallon; Tracy; Vavikolanu
EPI_ISL_7469697	Mass General Brigham	Mass General Brigham	A.E.; Adams, G.; Anahtar, M.; B.L.; B.W.; Bauer, M.; Birren; Branda, J.; Carter, A.; Cerrato, F.; Chaluvadi, S.; Chapman; Cusick, C.; D.J.; DeRuff, K.; E. and Sabeti; Flowers, K.; Gallagher, G.; Gladden-Young, A.; Gnirke, A.; Harris, J.; J.E.; K.J.; LaRocque, R.; Lagerborg, K.; Lemieux; Lin; Loreth, C.; MacInnis; Neumann, A.; Normandin, E.; P.C.; Park; Pierce, V.; Reilly, S.; Rosenberg; Rudy, M.; Ryan, E.; S.B.; Shaw, B.; Siddle; Slater, D.; Smole, S.; Tomkins-Tinch, C.; Turbett, S.; Uddin, R.
EPI_ISL_6886593, EPI_ISL_6886594, EPI_ISL_6886595, EPI_ISL_6886596, EPI_ISL_7590775, EPI_ISL_7590919, EPI_ISL_7590929, EPI_ISL_7590955, EPI_ISL_7590991, EPI_ISL_7591005, EPI_ISL_7591006, EPI_ISL_7591007, EPI_ISL_7591008, EPI_ISL_7591009, EPI_ISL_7591010, EPI_ISL_7591011, EPI_ISL_7591012, EPI_ISL_7591013, EPI_ISL_7591014, EPI_ISL_7591015, EPI_ISL_7591016, EPI_ISL_7591018, EPI_ISL_7591019			
see above	Max von Pettenkofer Institute, Virology, National Reference Center for Retroviruses, LMU Munich	Laboratory for Functional Genome Analysis; Dept. Genomics; Gene Center of the LMU Munich	Alexander Graf; Helmut Blum; Max Muenchhoff; Oliver Keppler; Stefan Krebs
EPI_ISL_7267259, EPI_ISL_7470216, EPI_ISL_7470264, EPI_ISL_7470331	Medical Microbiology Unit, Department for Laboratory Medicine, Drammen Hospital, Vestre Viken Health Trust	Norwegian Institute of Public Health, Department of Virology	Atiya R Ali; Debech Nadia; Engebretsen Serina Beate; Garcia Llorente Ignacio; Hilde Elshaug; Hilde Vollan; Jon Bråte; Kamilla Heddeland Instefjord; Karoline Bragstad; Kathrine Stene-Johansen; Line Victoria Moen; Marie Paulsen Madsen; Olav Hungnes; Pedersen Benedikte Nevjen; Rasmus Riis Kopperud
EPI_ISL_7443804, EPI_ISL_7443805, EPI_ISL_7443809, EPI_ISL_7443815	Medizinische Laboratorien Düsseldorf	Robert Koch Institute	
EPI_ISL_7416680	Medizinisches Versorgungszentrum für Labormedizin und Mikrobiologie Ruhr GmbH - mvzlm RUHR GmbH	Robert Koch Institute	
EPI_ISL_6854346, EPI_ISL_6854347, EPI_ISL_6854348	Microbiologia e Virologia Cotugno	Microbiologia e Virologia Cotugno	Antonio Canonico; Antonio Fascione; Claudia Tiberio; Enza Mallardo; Francesco Nappo; Giovanni D'Auria; Giuseppe di Gennaro; Ilaria Cavallaro; Luigi Atripaldi
EPI_ISL_7462324	Microbiology Department, University Hospital Araba	Microbiology Department, University Hospital Donostia	Cilla G.; Gomez M; Hernaez S; Marimon JM; Martin-Peñaranda T; Montes M; Piñeiro L; Sorarrain A
EPI_ISL_7502103, EPI_ISL_7502107	Microbiology Department. Complexo Hospitalario Universitario de Vigo	Microbiology Department. Complexo Hospitalario Universitario de Vigo	Alvarez M; Cabrera JJ; Carballo R; Cortizo S; Davina C; Martinez L; Mediero G; Pena I; Perez S; Potel C; Regueiro B; Rey S; Vasallo FJ; del-Campo V
EPI_ISL_7566142	Ministry of Health Turkey	Ministry of Health Turkey	Fatma Bayrakdar; Gulay Korukluoglu; Suleyman Yalcin; Yasemin Cosgun
EPI_ISL_7496678	Minnesota Department of Health, Public Health Laboratory	Minnesota Department of Health, Public Health Laboratory	Alyssa Mondelli; Elizabeth Horn; Jacob Garfin; Kelly Pung; Matt Plumb; Sarah Namugenyi; and Xiong Wang
EPI_ISL_6963002	Mirialis	CNR Virus des Infections Respiratoires -	Antoine Oblette; Antonin Bal; Bruno Lina; Bruno Simon; Camille Delcloitre; Eva Oddoux; Florence Morfin; Gregory Destras; Gwendolyne Burfin; Hadrien Regue; Hervé Crehalet; Jean François Bore; Jeremy Cordier; Laurence Josset; Martine Valette; Noémie Fessy; Quentin Semanas; Richard

		France SUD	Chalvignac; Thibault Corsin; Thibault Gouiran
EPI_ISL_7193991, EPI_ISL_7286479, EPI_ISL_7516233, EPI_ISL_7519931, EPI_ISL_7527590, EPI_ISL_7527959	Molekylær Medicinsk Afdeling, Aarhus University Hospital, Aarhus, Denmark	Statens Serum Institut Bioinformatics and Microbial Genomics	Danish Covid-19 Genome Consortium
EPI_ISL_7501186	Mount Auburn Hospital via Lahey Hospital	New England Biolabs	Abel, G.; B.W.; C.J.; Colgrove, R.; Duncan, R.; Elfahal, M.; Flynn; Heim, K.; Karolides, M.; L. and Langhorst; Michaels, L.; Pinet, K.; Skelton, T.; Sun
EPI_ISL_7545652, EPI_ISL_7545653, EPI_ISL_7545654, EPI_ISL_7545658, EPI_ISL_7545660, EPI_ISL_7545661, EPI_ISL_7545662, EPI_ISL_7545663, EPI_ISL_7545664, EPI_ISL_7545665, EPI_ISL_7545666, EPI_ISL_7545667, EPI_ISL_7545668, EPI_ISL_7545669, EPI_ISL_7545670, EPI_ISL_7545671, EPI_ISL_7545673, EPI_ISL_7545674, EPI_ISL_7545675			
see above	NHLS Livingstone Laboratory	CERI, Centre for Epidemic Response and Innvoation, Stellenbosch University and KRISP, KZN Research Innovation and Sequencing Platform, UKZN.	Arisha Maharaj; Giandhari J; Naidoo Y; Oluwakemi Laguda-Akingba and Nokukhanya Mdlalose; Pillay S; Ramphal U; Ramphal Y; San JE; Tegally H; Tshiabuila D; Wilkinson E; de Oliveira T
EPI_ISL_7381208, EPI_ISL_7381209, EPI_ISL_7381210, EPI_ISL_7381211, EPI_ISL_7381212, EPI_ISL_7381213, EPI_ISL_7381214, EPI_ISL_7381215, EPI_ISL_7381216, EPI_ISL_7381217, EPI_ISL_7381218, EPI_ISL_7381219, EPI_ISL_7381220, EPI_ISL_7381221, EPI_ISL_7381222			
see above	NHLS Port Elizabeth Laboratory	CERI, Centre for Epidemic Response and Innvoation, Stellenbosch University and KRISP, KZN Research Innovation and Sequencing Platform, UKZN.	Arisha Maharaj; Giandhari J; Moir M; Naidoo Y; Oluwakemi Laguda-Akingba and Nokukhanya Mdlalose; Pillay S; Ramphal U; Ramphal Y; San JE; Tegally H; Tshiabuila D; Wilkinson E; de Oliveira T; van Wyk S
EPI_ISL_7462390, EPI_ISL_7462391, EPI_ISL_7462392, EPI_ISL_7462393, EPI_ISL_7462397, EPI_ISL_7462398, EPI_ISL_7462399, EPI_ISL_7462400, EPI_ISL_7462401, EPI_ISL_7462402, EPI_ISL_7462403, EPI_ISL_7462404			
see above	NHLS Universitas Academic	Division of Medical Virology, National Health Laboratory Service (NHLS), Tygerberg Hospital / Stellenbosch University	D Goedhals; Emmanuel Ogunbayo; MM Nyaga; MT Mogotsi; P Nthiga; PA Bester; Shannon Wilson; Susan Engelbrecht; T de Oliveira; Tongai Maponga; Wolfgang Preiser
EPI_ISL_7605460, EPI_ISL_7605516	NJDOH, Public Health and Environmental Laboratories	NJ_PHEL	Allison Roder; Byeong Jeong; Chelsea San Filippo; Dana Woell; Jacquelyn Deverell; Lindsey Bodnar; Maria-Magdalene Pugliese; Mohammad M. Ali; Ryan Pachucki; Shiv K. Verma
EPI_ISL_7568605, EPI_ISL_7568608	NYU Langone Health	Departments of Pathology and Medicine, New York University School of Medicine	Adriana Heguy; Christian Marier; Dacia Dimartino; Emily Guzman; Gael Westby; Guiqing Wang; Paul Zappile; Peter Meyn; Sitharam Ramaswami; Yutong Zhang
EPI_ISL_7285023	National Centre for Disease Control (NCDC) Biotechnology Division, Delhi	NCDC Delhi, Biotechnology Division INSACOG	Hema Gogia; Hemlata Lall; Kalaiarasan Ponnusamy; Mahesh S Dhar; Manoj K Singh; Meena Datta; Partha Rakshit; Preeti Madan; Priyanka Singh; Radhakrishnan V. S; Robin Marwal; Sandhya Kabra; Sujeet K Singh; Uma Sharma
EPI_ISL_7548907, EPI_ISL_7548908, EPI_ISL_7548911, EPI_ISL_7548912, EPI_ISL_7548913, EPI_ISL_7548915, EPI_ISL_7548916, EPI_ISL_7548918, EPI_ISL_7548919, EPI_ISL_7548920, EPI_ISL_7548921, EPI_ISL_7548922, EPI_ISL_7548924, EPI_ISL_7548925, EPI_ISL_7548926, EPI_ISL_7548927, EPI_ISL_7548928, EPI_ISL_7548929, EPI_ISL_7548930, EPI_ISL_7548932, EPI_ISL_7548933, EPI_ISL_7548934, EPI_ISL_7548935, EPI_ISL_7548936, EPI_ISL_7548937, EPI_ISL_7548938, EPI_ISL_7548939, EPI_ISL_7552700, EPI_ISL_7552701, EPI_ISL_7552702, EPI_ISL_7552703, EPI_ISL_7552704, EPI_ISL_7552705, EPI_ISL_7552706, EPI_ISL_7552707, EPI_ISL_7552708, EPI_ISL_7552709, EPI_ISL_7552710, EPI_ISL_7566335			
see above	National Health Laboratory	Botswana Harvard AIDS Institute Partnership, Plot 1836 North Ring Road, Princess Marina Hospital, Gaborone	Boitumelo Zuze; Botshelo Radibe; Dorcas Maruapula; Doreen Ditshwanelo; Joseph Makhema; Keoratile Ntshambiwa; Kgomotso Moruisi; Legodile Kooepile; Mosepele Mosepele; Mphaphi B. Mbulawa; Ontlametse T. Bareng; Pamela Smith-Lawrence; Roger Shapiro; Sefetogi Ramaologa; Shahin Lockman; Sikhulile Moyo; Simani Gaseitsiwe; Thongbotho Mphoyakgosi; Wonderful T. Choga
EPI_ISL_6795195, EPI_ISL_6795199, EPI_ISL_6795202, EPI_ISL_6795203, EPI_ISL_6795406, EPI_ISL_7015210, EPI_ISL_7015212, EPI_ISL_7015216, EPI_ISL_7015223, EPI_ISL_7015224, EPI_ISL_7015226, EPI_ISL_7015229, EPI_ISL_7015230, EPI_ISL_7310589, EPI_ISL_7310595, EPI_ISL_7310605, EPI_ISL_7310613, EPI_ISL_7310622, EPI_ISL_7310630, EPI_ISL_7310631, EPI_ISL_7310636, EPI_ISL_7310643, EPI_ISL_7310648, EPI_ISL_7310658, EPI_ISL_7310666, EPI_ISL_7310675, EPI_ISL_7310679, EPI_ISL_7310685, EPI_ISL_7310703, EPI_ISL_7310710, EPI_ISL_7310719, EPI_ISL_7310725, EPI_ISL_7310733, EPI_ISL_7310742, EPI_ISL_7310747, EPI_ISL_7358050, EPI_ISL_7358051, EPI_ISL_7358052, EPI_ISL_7358053, EPI_ISL_7358054, EPI_ISL_7358055, EPI_ISL_7358056, EPI_ISL_7358057, EPI_ISL_7358058, EPI_ISL_7358059, EPI_ISL_7358064, EPI_ISL_7358065, EPI_ISL_7358066, EPI_ISL_7358067, EPI_ISL_7358068, EPI_ISL_7358069, EPI_ISL_7358070, EPI_ISL_7358071, EPI_ISL_7358072, EPI_ISL_7358073, EPI_ISL_7358074, EPI_ISL_7358075, EPI_ISL_7358076, EPI_ISL_7358077, EPI_ISL_7358078, EPI_ISL_7358079, EPI_ISL_7358080, EPI_ISL_7358081, EPI_ISL_7358082, EPI_ISL_7358083, EPI_ISL_7358084, EPI_ISL_7358085, EPI_ISL_7358089, EPI_ISL_7358090, EPI_ISL_7358091, EPI_ISL_7358092, EPI_ISL_7358093, EPI_ISL_7381192, EPI_ISL_7381193, EPI_ISL_7381194, EPI_ISL_7381195, EPI_ISL_7381196, EPI_ISL_7381197, EPI_ISL_7381198, EPI_ISL_7381199, EPI_ISL_7381200, EPI_ISL_7381201, EPI_ISL_7381202, EPI_ISL_7381203, EPI_ISL_7381204, EPI_ISL_7381205, EPI_ISL_7381206, EPI_ISL_7381207, EPI_ISL_7545676, EPI_ISL_7545677, EPI_ISL_7545678, EPI_ISL_7545679, EPI_ISL_7545680, EPI_ISL_7545681, EPI_ISL_7545682, EPI_ISL_7545683, EPI_ISL_7545684, EPI_ISL_7545685, EPI_ISL_7545686, EPI_ISL_7545687, EPI_ISL_7545688, EPI_ISL_7545689, EPI_ISL_7545690, EPI_ISL_7545691, EPI_ISL_7545692, EPI_ISL_7545693, EPI_ISL_7545694, EPI_ISL_7545695, EPI_ISL_7545696, EPI_ISL_7545698, EPI_ISL_7545699, EPI_ISL_7545700, EPI_ISL_7545701, EPI_ISL_7545702, EPI_ISL_7545703, EPI_ISL_7545704, EPI_ISL_7545705, EPI_ISL_7545706, EPI_ISL_7545707, EPI_ISL_7545708, EPI_ISL_7545709, EPI_ISL_7545710, EPI_ISL_7545711, EPI_ISL_7545712, EPI_ISL_7545713, EPI_ISL_7545714, EPI_ISL_7545715, EPI_ISL_7545716, EPI_ISL_7545717, EPI_ISL_7545718, EPI_ISL_7545719, EPI_ISL_7545720, EPI_ISL_7545721, EPI_ISL_7545722, EPI_ISL_7545723, EPI_ISL_7545724, EPI_ISL_7545725, EPI_ISL_7545726, EPI_ISL_7545727, EPI_ISL_7545728, EPI_ISL_7545729, EPI_ISL_7545730, EPI_ISL_7545731, EPI_ISL_7545732, EPI_ISL_7545733, EPI_ISL_7545734, EPI_ISL_7545735, EPI_ISL_7545736, EPI_ISL_7545737, EPI_ISL_7545738, EPI_ISL_7545739, EPI_ISL_7545742, EPI_ISL_7545743, EPI_ISL_7545744, EPI_ISL_7545745, EPI_ISL_7545746, EPI_ISL_7545747, EPI_ISL_7545748, EPI_ISL_7545749, EPI_ISL_7545750, EPI_ISL_7545751, EPI_ISL_7545752, EPI_ISL_7545753, EPI_ISL_7545754, EPI_ISL_7545755, EPI_ISL_7545756, EPI_ISL_7545757, EPI_ISL_7545758, EPI_ISL_7545759, EPI_ISL_7545760, EPI_ISL_7545761, EPI_ISL_7545762, EPI_ISL_7545764, EPI_ISL_7545765, EPI_ISL_7545766, EPI_ISL_7545767, EPI_ISL_7545768, EPI_ISL_7545769, EPI_ISL_7545770, EPI_ISL_7545771, EPI_ISL_7545772, EPI_ISL_7545773, EPI_ISL_7545774, EPI_ISL_7545775, EPI_ISL_7545776, EPI_ISL_7545777, EPI_ISL_7545778, EPI_ISL_7545779, EPI_ISL_7545780, EPI_ISL_7545781, EPI_ISL_7545782, EPI_ISL_7545783, EPI_ISL_7545784, EPI_ISL_7545785, EPI_ISL_7545787, EPI_ISL_7545788, EPI_ISL_7545789, EPI_ISL_7545790, EPI_ISL_7545791, EPI_ISL_7545792, EPI_ISL_7545793, EPI_ISL_7545794			
see above	National Health Laboratory Service, Kwazulu-Natal, South Africa	CERI, Centre for Epidemic Response and Innvoation, Stellenbosch University and KRISP, KZN Research Innovation and Sequencing Platform, UKZN.	Arisha Maharaj; Giandhari J; Moir M; Naidoo Y; Nokukhanya Mdlalose; Pillay S; Ramphal U; Ramphal Y; San JE; Tegally H; Tshiabuila D; Wilkinson E; de Oliveira T; van Wyk S
EPI_ISL_6829557	National Health Laboratory Service, Kwazulu-Natal, South Africa	KRISP, KZn Research Innovation and Sequencing Platform	Arisha Maharaj; Giandhari J; Lessells R; Moir M; Naidoo Y; Nokukhanya M; Pillay S; Ramphal U; Ramphal Y; San JE; Tegally H; Tshiabuila D; Wilkinson E; de Oliveira T
EPI_ISL_6795188, EPI_ISL_6795189, EPI_ISL_6795190, EPI_ISL_6795191, EPI_ISL_6795192, EPI_ISL_6795193	National Health Laboratory Services, Virology	CERI, Centre for Epidemic Response and Innvoation, Stellenbosch University and KRISP, KZN Research Innovation and Sequencing Platform, UKZN.	Arisha Maharaj; Florette Treurnicht; Giandhari J; Kathleen Subramoney; Naidoo Y; Pillay S; Ramphal U; Ramphal Y; San JE; Tegally H; Tshiabuila D; Wilkinson E; de Oliveira T
EPI_ISL_6699728, EPI_ISL_6699729, EPI_ISL_6699730, EPI_ISL_6699731, EPI_ISL_6699732, EPI_ISL_6699733, EPI_ISL_6699734, EPI_ISL_6699735, EPI_ISL_6699736, EPI_ISL_6699737, EPI_ISL_6699738, EPI_ISL_6699739, EPI_ISL_6699740, EPI_ISL_6699741, EPI_ISL_6699742, EPI_ISL_6699743, EPI_ISL_6699744, EPI_ISL_6699745, EPI_ISL_6699746, EPI_ISL_6699747, EPI_ISL_6699748, EPI_ISL_6699749, EPI_ISL_6699750, EPI_ISL_6699751, EPI_ISL_6699752, EPI_ISL_6699753, EPI_ISL_6699754, EPI_ISL_6699755, EPI_ISL_6699756, EPI_ISL_6699757, EPI_ISL_6699758, EPI_ISL_6699759, EPI_ISL_6699760, EPI_ISL_6699761, EPI_ISL_6699762, EPI_ISL_6699763, EPI_ISL_6699764, EPI_ISL_6699765, EPI_ISL_6699766, EPI_ISL_6699767, EPI_ISL_6699768, EPI_ISL_6699769, EPI_ISL_6699770, EPI_ISL_6699771, EPI_ISL_6782043, EPI_ISL_6782055, EPI_ISL_6782056, EPI_ISL_6782071, EPI_ISL_6782079, EPI_ISL_6782080, EPI_ISL_6782084, EPI_ISL_6782090, EPI_ISL_6782092, EPI_ISL_6810482, EPI_ISL_6810483, EPI_ISL_6810484, EPI_ISL_6810485, EPI_ISL_6810486, EPI_ISL_6810487			
see above	National Health Laboratory Services, Virology, Charlotte Maxeke Johannesburg hospital, Parktown, Johannesburg, Gauteng	CERI, Centre for Epidemic Response and Innvoation, Stellenbosch University and KRISP, KZN Research Innovation and Sequencing Platform, UKZN.	Amoaka D; Arisha Maharaj; Avani Bharuthram; Bester P; Bhiman J; Engelbrecht S; Everatt J; Florette Treurnicht; Goedhals D; Hardie D; Hsiao M; Iranzadeh A; Kathleen Subramoney; Lessells R; Makatini Z; Maponga T; Mdlalose N; Mlisana K; Moir M; NGS-SA (Scheepers C; Naidoo Y; Nkhensani Mtileni; Nyaga M) Giandhari J; Oluwakemi M; Pillay S; Preiser W; Ramphal U; Ramphal Y; San JE; Tegally H; Tshiabuila D; Venter M; Wilkinson E; Williamson C; de Oliveira T; von Gottberg A
EPI_ISL_6939033, EPI_ISL_6939034, EPI_ISL_6939035, EPI_ISL_6939036, EPI_ISL_6939038, EPI_ISL_6939039, EPI_ISL_6939041, EPI_ISL_6939042, EPI_ISL_6939043, EPI_ISL_6939044, EPI_ISL_6939045, EPI_ISL_6939046, EPI_ISL_6939047, EPI_ISL_6939048, EPI_ISL_6939049, EPI_ISL_6939050, EPI_ISL_6939051, EPI_ISL_6939052, EPI_ISL_6939053, EPI_ISL_6939054, EPI_ISL_6939056, EPI_ISL_6939057, EPI_ISL_6939058, EPI_ISL_6939059, EPI_ISL_6939060, EPI_ISL_6939061, EPI_ISL_6939062, EPI_ISL_6939063, EPI_ISL_6939064, EPI_ISL_6939065, EPI_ISL_6939066, EPI_ISL_6939067, EPI_ISL_6939068			
see above	National Influenza Centre	National Influenza Centre	; Benjamiin B. Lindsey; Benjamin H. Foulkes; Bless Seyram Agbenyo; Bright Adu; Ernest Asiedu; Franklin Asiedu-Bekoe; Hilda Opoku Frempong; Ivy A. Asante; Joseph Oliver-Commey; Joyce Appiah-Kubi; Keren Okyerebea Attiku; Linda Boatemaa; Lorreta Kwasah; Mathew D. Parker; Michael Marks; Mildred Adusei-Poku; Quaneeta Mohktar; Sharon Hsu; Thushan I de Silva; William K. Ampofo
EPI_ISL_7456448, EPI_ISL_7456450, EPI_ISL_7456451, EPI_ISL_7456452, EPI_ISL_7456453, EPI_ISL_7456454, EPI_ISL_7456455, EPI_ISL_7456456, EPI_ISL_7456457			
see above	National Institute For Communicable Diseases Of The National Health Laboratory Service	National Institute for Communicable Diseases of the National Health Laboratory Service	Amoako DG; Bhiman JN; Everatt J; Ismail A; Mahlangu B; Mnguni A; Mohale T; Ntuli N; Scheepers C; Wolter N
EPI_ISL_7605585, EPI_ISL_7605586, EPI_ISL_7605593, EPI_ISL_7605594, EPI_ISL_7605629, EPI_ISL_7605630, EPI_ISL_7605631, EPI_ISL_7605632, EPI_ISL_7605637, EPI_ISL_7605638, EPI_ISL_7605666, EPI_ISL_7605720, EPI_ISL_7605759, EPI_ISL_7605762, EPI_ISL_7605765, EPI_ISL_7605767, EPI_ISL_7605768, EPI_ISL_7605770, EPI_ISL_7605771			
see above	National Institute for Communicable Diseases of the National Health Laboratory Service	National Institute for Communicable Diseases of the National Health Laboratory Service	Amoako DG; Bhiman JN; Everatt J; Ismail A; Mahlangu B; Mnguni A; Mohale T; Ntuli N; Scheepers C; Wolter N
EPI_ISL_7418017, EPI_ISL_7571617, EPI_ISL_7571618	National Institute of Infectious Diseases	National Institute of Infectious Diseases	Harutaka Katano; Ken Maeda; Kentaro Itokawa; Makoto Kuroda; Shuetsu Fukushi; Shun Iida; Tadaki Suzuki; Tsuyoshi Sekizuka; Yudai Kuroda
EPI_ISL_7021517, EPI_ISL_7074135	National Platform bis COVID ULB-IBC	National Platform bis COVID ULB-IBC	Arnaud Marchant; Benoit Haerlingen; Coralie Henin; Marie-Luce Delforge; Ricardo De Mendonça
EPI_ISL_7063764, EPI_ISL_7288348, EPI_ISL_7288357, EPI_ISL_7288375, EPI_ISL_7544936, EPI_ISL_7544937, EPI_ISL_7544938, EPI_ISL_7544939, EPI_ISL_7544940, EPI_ISL_7544941, EPI_ISL_7544942, EPI_ISL_7544947, EPI_ISL_7544948, EPI_ISL_7544949, EPI_ISL_7544950, EPI_ISL_7544953, EPI_ISL_7544954, EPI_ISL_7544956, EPI_ISL_7544960, EPI_ISL_7544963, EPI_ISL_7544964, EPI_ISL_7544965, EPI_ISL_7544969, EPI_ISL_7544971, EPI_ISL_7544972, EPI_ISL_7544974, EPI_ISL_7544975, EPI_ISL_7544980			
see above	National Platform bis UMONS/Jolimont	National Platform bis UMONS/Jolimont	Eric Tarantino; Florian Juszczak; Gautier Detry; Guillaume Bayon-Vicente; Laetitia Gheysen; Ruddy Wattiez
EPI_ISL_7137310, EPI_ISL_7137311, EPI_ISL_7195620, EPI_ISL_7195621, EPI_ISL_7195622, EPI_ISL_7195623, EPI_ISL_7460338, EPI_ISL_7604552, EPI_ISL_7604594, EPI_ISL_7604595, EPI_ISL_7604613, EPI_ISL_7604615, EPI_ISL_7604627			
see above	National Public Health Laboratory, National Centre for Infectious Diseases	National Public Health Laboratory, National Centre for Infectious Diseases	Benny Yeo Ken Yee; Constance Chen; Dennis Loy Song Qi; Dimitar Kenanov; Grace Ngan Jie Yin; Katherine Ching; Katherine Ching Zi Yan; Kwan Ki Ko; Lin Cui; Mak Tze Minn; Niranjan Nagarajan; Raymond Tzer Pin Lin; Royce Ang; Samuel Loo; Sebastian Maurer Stroh; Suphavilai Chayaporn; Zhenyang Zhou
EPI_ISL_7154394, EPI_ISL_7154403, EPI_ISL_7273097, EPI_ISL_7547734,	National Reference Laboratory, NCDC	National Reference Laboratory, Nigeria Centre for Disease Control	Catherine Okoi; Chimaobi Chukwu; Dr Ifedayo Adetifa; Dr Ndodo Nnaemeka; Dr Omoare Adesuyi; Nwando Mba; Olajumoke Babatunde; Olusola Anuoluwapo Akanbi; Oyeronke Ayansola

EPI_ISL_7547735			
EPI_ISL_6939819, EPI_ISL_7354124, EPI_ISL_7406048, EPI_ISL_7406049, EPI_ISL_7406082, EPI_ISL_7406083	National Virus Reference Laboratory	National Virus Reference Laboratory	Charlene Bennett; Cillian F De Gascun; Gabriel Gonzalez; Jonathan Dean; Michael Carr; Zoe Yandle
EPI_ISL_7616432, EPI_ISL_7616434, EPI_ISL_7616435, EPI_ISL_7616437, EPI_ISL_7616439, EPI_ISL_7616441, EPI_ISL_7616442			
see above	Naval Health Clinic Annapolis	Naval Medical Research Center Biological Defense Research Directorate	Catherine E. Arnold; Francisco Malagon; Kimberly A. Bishop-Lilly; Kyle A. Long; Logan J. Voegtly; Megan A. Schilling; Regina Z. Cer
EPI_ISL_7605624, EPI_ISL_7605625, EPI_ISL_7605626, EPI_ISL_7605627, EPI_ISL_7605628	Ndlovu Reaserch Centre	National Institute for Communicable Diseases of the National Health Laboratory Service	Amoako DG; Bhiman JN; Everatt J; Ismail A; Mahlangu B; Mnguni A; Mohale T; Ntuli N; Scheepers C; Wolter N
EPI_ISL_7116413, EPI_ISL_7116439, EPI_ISL_7116461, EPI_ISL_7116681, EPI_ISL_7116718, EPI_ISL_7116738	Nebraska Public Health Laboratory	NPHL COVID-19 Response Team	NPHL COVID-19 Response Team
EPI_ISL_7544379, EPI_ISL_7544739	New Somerset Hospital wc NSH	NHLS/UCT	Arash Iranzadeh; Bruna Galvao; Carolyn Williamson; Deelan Doolabh; Diana Hardie; Gert Marais; Innocent Mudau; Luicer Olubayo; Lynn Tyers; Marvin Hsiao; Nokuzola Mbhele; Rageema Joseph; Stephen Korsman
EPI_ISL_6814922, EPI_ISL_6814923, EPI_ISL_6829575, EPI_ISL_6829577, EPI_ISL_6958955, EPI_ISL_7162071, EPI_ISL_7162072, EPI_ISL_7162073, EPI_ISL_7265843, EPI_ISL_7379517, EPI_ISL_7379527, EPI_ISL_7457536, EPI_ISL_7457537, EPI_ISL_7457538, EPI_ISL_7503742, EPI_ISL_7503743, EPI_ISL_7503744, EPI_ISL_7503745, EPI_ISL_7503746, EPI_ISL_7551964, EPI_ISL_7551971, EPI_ISL_7569451, EPI_ISL_7569452, EPI_ISL_7569453, EPI_ISL_7622408, EPI_ISL_7622409, EPI_ISL_7622410, EPI_ISL_7622411, EPI_ISL_7622412			
see above	New South Wales Health Pathology Royal Prince Alfred Hospital	Microbiology RPAH	Au, J.; Bull, R.; Deveson, I.; Foster, C.; Rawlinson, W.; Ruiz Silva, M.; Van Hal, S.
EPI_ISL_7418387, EPI_ISL_7431953	Niedersächsisches Landesgesundheitsamt (NLGA)	Robert Koch Institute	
EPI_ISL_6958280, EPI_ISL_7571541, EPI_ISL_7571542	North Lantau Hospital	Hong Kong Department of Health	Alan K.L. Tsang; Edman T.K. Lam; Ken H.L. Ng; Patricia K. L. Leung; Peter C.W. Yip; Rickjason C.W. Chan
EPI_ISL_7601238, EPI_ISL_7601282, EPI_ISL_7601310, EPI_ISL_7614981	OKMI	University Hospital Brno, CMBG	Bezdicek Matej; Dolejska Monika; Kristyna Dufkova; Lengerova Martina; Svaton Jan
EPI_ISL_7334887, EPI_ISL_7334888	ONEIDA COUNTY HEALTH DEPT.	Wadsworth Center, New York State Department of Health	Alexis Russell; Catharine Prussing; Daryl M. Lamson; Erasmus Schneider; Erica Lasek-Nesselquist; John Kelly; Jonathan Plitnick; Kirsten St. George; Matthew Shudt; Melissa A Leisner; Navjot Singh
EPI_ISL_7552440, EPI_ISL_7552445, EPI_ISL_7552446	Ochsner Health	BioInfoExperts	Amy Feehan; Ben Lain; Chris Huston; David J. Nolan; Judy Crabtree; Julia-Garcia-Diaz; Lucio Miele; Rebecca Rose; Samual Moot; Susanna L. Lamers; Tessa LaFleur
EPI_ISL_7495763	Omega Diagnostics at Mounes	Omega Diagnostics at Mounes	Cherish Jackson; Cynthia Corley; Latira Haynes-Jacob; MD; Vivek Khare
EPI_ISL_7040235	Oslo University Hospital, Department of Medical Microbiology	Norwegian Institute of Public Health, Department of Virology	Atiya R Ali; Debech Nadia; Engebretsen Serina Beate; Garcia Llorente Ignacio; Hilde Elshaug; Hilde Vollan; Jon Bråte; Kamilla Heddeland Instefjord; Karoline Bragstad; Kathrine Stene-Johansen; Line Victoria Moen; Marie Paulsen Madsen; Olav Hungnes; Pedersen Benedikte Nevjen; Rasmus Riis Kopperud
EPI_ISL_7248730, EPI_ISL_7248745, EPI_ISL_7248753, EPI_ISL_7248763, EPI_ISL_7470724, EPI_ISL_7470728	Oslo University Hospital, Department of Microbiology	Norwegian Institute of Public Health, Department of Virology	Arvind Yegambaram Meenakshi Sundaram; Atiya R Ali; Cathrine Fladeby; Debech Nadia; Engebretsen Serina Beate; Garcia Llorente Ignacio; Gregor D. Gilfillan; Hilde Elshaug; Hilde Vollan; Jon Bråte; Kamilla Heddeland Instefjord; Karoline Bragstad; Kathrine Stene-Johansen; Line Victoria Moen; Lise Andresen; Mariann Nilsen; Marie Paulsen Madsen; Mona Holberg-Petersen; Olav Hungnes; Pedersen Benedikte Nevjen; Pål Marius Bjørnstad; Rasmus Riis Kopperud; Teodora Plamenova Ribarska
EPI_ISL_7470189	Ostfold Hospital Trust - Kalnes, Centre for Laboratory Medicine, Section for gene technology and infection serology	Norwegian Institute of Public Health, Department of Virology	Atiya R Ali; Debech Nadia; Engebretsen Serina Beate; Garcia Llorente Ignacio; Hilde Elshaug; Hilde Vollan; Jon Bråte; Kamilla Heddeland Instefjord; Karoline Bragstad; Kathrine Stene-Johansen; Line Victoria Moen; Marie Paulsen Madsen; Olav Hungnes; Pedersen Benedikte Nevjen; Rasmus Riis Kopperud
EPI_ISL_7190366, EPI_ISL_7467356	PHV-FSS	PHV-FSS	Chenwei Wang on behalf of Q-PHIRE Genomics
EPI_ISL_7605742, EPI_ISL_7605764	PORT ELIZABETH LABORATORY	National Institute for Communicable Diseases of the National Health Laboratory Service	Amoako DG; Bhiman JN; Everatt J; Ismail A; Mahlangu B; Mnguni A; Mohale T; Ntuli N; Scheepers C; Wolter N
EPI_ISL_7605607	POTCHEFSTROOM LABORATORY	National Institute for Communicable Diseases of the National Health Laboratory Service	Amoako DG; Bhiman JN; Everatt J; Ismail A; Mahlangu B; Mnguni A; Mohale T; Ntuli N; Scheepers C; Wolter N
EPI_ISL_6774082, EPI_ISL_6774086, EPI_ISL_6774092	Palapye Primary Hospital Laboratory	Botswana Harvard HIV Reference Laboratory	Boitumelo Zuze; Botshelo Radibe; Dorcas Maruapula; Joseph Makhema; Keoratile Ntshambiwa; Kgomotso Moruisi; Legodile Kooepile; Mosepele Mosepele; Mphaphi B. Mbulawa; Ontlametse T. Bareng; Pamela Smith-Lawrence; Roger Shapiro; Sefetogi Ramaologa; Shahin Lockman; Sikhulile Moyo; Simani Gaseitsiwe; Thongbotho Mphoyakgosi; Wonderful T. Choga
EPI_ISL_7129868, EPI_ISL_7129869, EPI_ISL_7602801, EPI_ISL_7604813, EPI_ISL_7604824, EPI_ISL_7614032, EPI_ISL_7614035, EPI_ISL_7614040			
see above	Pandemic Response Lab - NYC	Pandemic Response Lab, R&D	Alex Carpio; Cybill del Castillo; Dylan Law; Haiping Hao; Henry Lee; Isabel Fernandez Escapa; Jon Laurent; Melissa Hopkins; Michael Hammerling; Pradeep Bugga; Shinyoung Clair Kang; Sol Rey; William Ward
EPI_ISL_6842152, EPI_ISL_6842154, EPI_ISL_6842155, EPI_ISL_6842157, EPI_ISL_6842158, EPI_ISL_6842160, EPI_ISL_6842161, EPI_ISL_6842164, EPI_ISL_6842166, EPI_ISL_6842167, EPI_ISL_7452739, EPI_ISL_7452740, EPI_ISL_7452743, EPI_ISL_7452747, EPI_ISL_7452752, EPI_ISL_7452753, EPI_ISL_7452754, EPI_ISL_7452755, EPI_ISL_7452756, EPI_ISL_7452757, EPI_ISL_7452759, EPI_ISL_7452760, EPI_ISL_7452779, EPI_ISL_7452784, EPI_ISL_7452786, EPI_ISL_7452787, EPI_ISL_7452788, EPI_ISL_7452789, EPI_ISL_7452790, EPI_ISL_7452791, EPI_ISL_7452801, EPI_ISL_7452802, EPI_ISL_7452803, EPI_ISL_7452804, EPI_ISL_7456466, EPI_ISL_7456467, EPI_ISL_7456468, EPI_ISL_7456469, EPI_ISL_7456470, EPI_ISL_7456471, EPI_ISL_7456472, EPI_ISL_7456473, EPI_ISL_7456474, EPI_ISL_7456475, EPI_ISL_7456476, EPI_ISL_7456477, EPI_ISL_7456478, EPI_ISL_7456479, EPI_ISL_7456480, EPI_ISL_7456481, EPI_ISL_7456482, EPI_ISL_7456483, EPI_ISL_7456484, EPI_ISL_7456485, EPI_ISL_7456489, EPI_ISL_7456490, EPI_ISL_7456491, EPI_ISL_7456492, EPI_ISL_7456493, EPI_ISL_7456494, EPI_ISL_7456495, EPI_ISL_7456496, EPI_ISL_7456497, EPI_ISL_7456498, EPI_ISL_7456499, EPI_ISL_7456500, EPI_ISL_7456501, EPI_ISL_7456502, EPI_ISL_7456503, EPI_ISL_7456504, EPI_ISL_7456505, EPI_ISL_7456506, EPI_ISL_7456507, EPI_ISL_7456508, EPI_ISL_7456509, EPI_ISL_7456510, EPI_ISL_7456511, EPI_ISL_7456512, EPI_ISL_7456513, EPI_ISL_7456514, EPI_ISL_7456515, EPI_ISL_7456516, EPI_ISL_7456517, EPI_ISL_7456518, EPI_ISL_7456519, EPI_ISL_7456520, EPI_ISL_7456521, EPI_ISL_7456522, EPI_ISL_7456523, EPI_ISL_7456524, EPI_ISL_7544906, EPI_ISL_7544907, EPI_ISL_7544908, EPI_ISL_7544909, EPI_ISL_7544910, EPI_ISL_7544911, EPI_ISL_7544912, EPI_ISL_7544913, EPI_ISL_7544914, EPI_ISL_7544915, EPI_ISL_7544916, EPI_ISL_7544917, EPI_ISL_7544918, EPI_ISL_7544919, EPI_ISL_7544920, EPI_ISL_7544921, EPI_ISL_7544922, EPI_ISL_7544923, EPI_ISL_7544924, EPI_ISL_7544925, EPI_ISL_7544926, EPI_ISL_7544927, EPI_ISL_7544928			
see above	PathCare, Cape Town	Division of Medical Virology, National Health Laboratory Service (NHLS), Tygerberg Hospital / Stellenbosch University	Gert van Zyl; Jean Maritz; Kamela Mahlakwane; Nadine Cronje; Petra Raimond; Shannon Wilson; Tania Stander; Tongai Maponga; Wolfgang Preiser
EPI_ISL_7263932, EPI_ISL_7263933	Pathogenic Microorganisms Variability Laboratory	Pathogenic Microorganisms Variability Laboratory	Alexander Gintsburg; Alexander Voskoboinikov; Andrei Siniavin; Andrey Pochtovyy; Artem Tkachuk; Denis Logunov; Elena Shidlovskaya; Elizaveta Divisenko; Inna Dolzhikova; Lyudmila Vasilchenko; Nadezhda Kuznetsova; Odintsova Alina; Vladimir Gushchin
EPI_ISL_7621339	Pathology North - Gosford Hospital - NSW Health Pathology	NSW Health Pathology - Institute of Clinical Pathology and Medical Research; Westmead Hospital; University of Sydney	Arnott A.; Draper J.; Gall M.; Martinez E.; Rockett R.; Sintchenko V.; on behalf of ICPMR
EPI_ISL_7265083, EPI_ISL_7265084	Pathology North - Royal North Shore Hospital - NSW Health Pathology	NSW Health Pathology - Institute of Clinical Pathology and Medical Research; Westmead Hospital; University of Sydney	Arnott A.; Draper J.; Gall M.; Martinez E.; Rockett R.; Sintchenko V.; on behalf of ICPMR
EPI_ISL_6864915, EPI_ISL_6956011, EPI_ISL_6956014, EPI_ISL_7620963, EPI_ISL_7621211, EPI_ISL_7621359, EPI_ISL_7621961			
see above	Pathology West - NSW Health Pathology	NSW Health Pathology - Institute of Clinical Pathology and Medical Research; Westmead Hospital; University of Sydney	Arnott A.; Draper J.; Gall M.; Martinez E.; Rockett R.; Sintchenko V.; on behalf of ICPMR
EPI_ISL_7565646	Pathology and Laboratory Medicine Institute, Cleveland Clinic, Ohio, USA	Pathology and Laboratory Medicine Institute, Cleveland Clinic, Ohio, USA	Concetta Peck; Daniel H. Farkas; Daniel Rhoads; David Bosler; David Plunkett; Jay Brock; Jennifer Starbuck; Jessica Spildener; Joy Nakitandwe; Kristen McDonnell; Nicole Hamon; Thomas Rose; Yu-Wei Cheng; Zheng Jin Tu
EPI_ISL_7406251,	Plateforme de testing Namuroise	Plateforme de testing Namuroise	Degosserie Jonathan; Demars Aurore; Denis Olivier; Gilliard Nicolas; Maschietto Céline; Mullier François; Nobis Chloé; Otto Gaetan

EPI_ISL_7472848, EPI_ISL_7472859, EPI_ISL_7472865			
EPI_ISL_7173899, EPI_ISL_7566194, EPI_ISL_7606105, EPI_ISL_7606106, EPI_ISL_7606107, EPI_ISL_7606108	Platform BIS UZA/UAntwerpen	Labo Klinische Biologie, UZA	Basil Britto Xavier; Christine Lammens; Herman Goossens; Ines Verbesselt; Jasmine Coppens; Kathleen Holemans; Marie Le Mercier; Veerle Matheeussen
EPI_ISL_6777160	Policlinico San Donato	Laboratory of Clinical Microbiology, Virology and Bioemergencies, ASST Fatebenefratelli Sacco - Sacco Hospital	Valeria Micheli
EPI_ISL_7416687, EPI_ISL_7416708	Procomcure Biotech Germany GmbH	Robert Koch Institute	
EPI_ISL_7507116, EPI_ISL_7507117, EPI_ISL_7507119	Public Health Authority of the Slovak Republic	Public Health Authority of the Slovak Republic	Anna Gičová; Barbora Kotvasová; Elena Tichá; Lucia Ševčíková; Miroslav Böhmer; Pavol Mišenko; Terézia Vrabľová; Tomáš Szemes
EPI_ISL_7135501, EPI_ISL_7135502, EPI_ISL_7135503, EPI_ISL_7135504, EPI_ISL_7263924, EPI_ISL_7263925, EPI_ISL_7263926, EPI_ISL_7263927, EPI_ISL_7263928, EPI_ISL_7263929, EPI_ISL_7263930, EPI_ISL_7334884, EPI_ISL_7334885, EPI_ISL_7334886, EPI_ISL_7438844, EPI_ISL_7438882, EPI_ISL_7438901, EPI_ISL_7438918, EPI_ISL_7438932, EPI_ISL_7438964, EPI_ISL_7438994, EPI_ISL_7459993, EPI_ISL_7459994, EPI_ISL_7459995, EPI_ISL_7459996, EPI_ISL_7459997, EPI_ISL_7459998, EPI_ISL_7495449, EPI_ISL_7495450, EPI_ISL_7495451, EPI_ISL_7495452, EPI_ISL_7495453, EPI_ISL_7495454, EPI_ISL_7495455, EPI_ISL_7620050, EPI_ISL_7620051, EPI_ISL_7620052, EPI_ISL_7620053, EPI_ISL_7620054, EPI_ISL_7620055, EPI_ISL_7620056			
see above	Public Health Laboratory, Public Health Service Amsterdam, The Netherlands	Department of Medical Microbiology & Infection prevention, Amsterdam University Medical Centers location AMC	Akke Cornelissen; Fokla Zorgdrager; Janke Schinkel; Jelle Koopsen; Judith den Uil; Marcel Jonges; Matthijs Welkers; Menno de Jong; Robin van Houdt; Sebastien Matamoros; Sjoerd Rebers; Sylvia Bruisten; Tjalling Leenstra and Mariken van der Lubben on behalf of the Amsterdam Regional Genomic epidemiology and Outbreak Surveillance (ARGOS) consortium
EPI_ISL_7135499, EPI_ISL_7259544, EPI_ISL_7259701, EPI_ISL_7259710, EPI_ISL_7259716, EPI_ISL_7259721, EPI_ISL_7259732, EPI_ISL_7259741, EPI_ISL_7259744, EPI_ISL_7334879, EPI_ISL_7334880, EPI_ISL_7464493, EPI_ISL_7464494, EPI_ISL_7613101, EPI_ISL_7613160, EPI_ISL_7613179, EPI_ISL_7613185, EPI_ISL_7613224, EPI_ISL_7613230, EPI_ISL_7613235, EPI_ISL_7613242			
see above	Public Health Ontario Laboratory	Public Health Ontario Laboratory	Aimin Li; Alireza Eshaghi; Andre Villegas; Ashleigh Sullivan; Christine Frantz; Dean Maxwell; Esha Joshi; Jared Simpson; Jennifer L Guthrie; Jonathan B Gubbay; Karthikeyan Sivaraman; Lawrence Heisler; Matthew Watson; Michael CY Li; Michael Laszloffy; Nahuel Fittipaldi; Philip Banh; Richard de Borja; Samir N Patel; Sandeep Nagra; Sandra Zittermann; Sarah Teatero; Vanessa G Allen; Yao Chen; Yogi Sundaravadanam
EPI_ISL_7592963	Puerto Rico Department of Health	Centers for Disease Control and Prevention, Dengue Branch	Betzabel Flores; Gabriela Paz-Bailey; Gilberto A. Santiago; Glenda Gonzalez; Jorge L. Munoz-Jordan; Keyla Charriez
EPI_ISL_7605590, EPI_ISL_7605591, EPI_ISL_7605651, EPI_ISL_7605652, EPI_ISL_7605653, EPI_ISL_7605654, EPI_ISL_7605655, EPI_ISL_7605656, EPI_ISL_7605658, EPI_ISL_7605659, EPI_ISL_7605660, EPI_ISL_7605661, EPI_ISL_7605662, EPI_ISL_7605663, EPI_ISL_7605664, EPI_ISL_7605667, EPI_ISL_7605668, EPI_ISL_7605671, EPI_ISL_7605672, EPI_ISL_7605673, EPI_ISL_7605674, EPI_ISL_7605675, EPI_ISL_7605679, EPI_ISL_7605682, EPI_ISL_7605685, EPI_ISL_7605704, EPI_ISL_7605738, EPI_ISL_7605751, EPI_ISL_7605772, EPI_ISL_7605773, EPI_ISL_7605776, EPI_ISL_7605777, EPI_ISL_7605778			
see above	ROB FERREIRA LABORATORY	National Institute for Communicable Diseases of the National Health Laboratory Service	Amoako DG; Bhiman JN; Everatt J; Ismail A; Mahlangu B; Mnguni A; Mohale T; Ntuli N; Scheepers C; Wolter N
EPI_ISL_7605676, EPI_ISL_7605740	RUSTENBURG LABORATORY	National Institute for Communicable Diseases of the National Health Laboratory Service	Amoako DG; Bhiman JN; Everatt J; Ismail A; Mahlangu B; Mnguni A; Mohale T; Ntuli N; Scheepers C; Wolter N
EPI_ISL_6862005	Regional Hospital Liberec	Regional Hospital Liberec	Iva Dolinova; Katerina Arientova; Katerina Stillerova; Martin Kracik; Tomas Zajic
EPI_ISL_7166216, EPI_ISL_7345201, EPI_ISL_7345221, EPI_ISL_7345322, EPI_ISL_7398995, EPI_ISL_7399058, EPI_ISL_7399073, EPI_ISL_7399078, EPI_ISL_7399512, EPI_ISL_7400550, EPI_ISL_7400551, EPI_ISL_7400555, EPI_ISL_7463952, EPI_ISL_7463953, EPI_ISL_7463956, EPI_ISL_7463961, EPI_ISL_7463968, EPI_ISL_7463969, EPI_ISL_7463972, EPI_ISL_7463975, EPI_ISL_7463979, EPI_ISL_7463997, EPI_ISL_7464020, EPI_ISL_7464030, EPI_ISL_7464048, EPI_ISL_7464049, EPI_ISL_7464059, EPI_ISL_7509826, EPI_ISL_7509828, EPI_ISL_7509830, EPI_ISL_7563604, EPI_ISL_7563965, EPI_ISL_7563969, EPI_ISL_7583283, EPI_ISL_7583286, EPI_ISL_7583295, EPI_ISL_7583354, EPI_ISL_7583375, EPI_ISL_7583411, EPI_ISL_7637839, EPI_ISL_7637844, EPI_ISL_7637845			
see above	Respiratory Virus Unit, Microbiology Services Colindale, Public Health England	COVID-19 Genomics UK (COG-UK) Consortium	PHE Covid Sequencing Team
EPI_ISL_7116918	Robert Koch-Institut ZBS1 (Zentrum für biologische Gefahren und spezielle Pathogene hochpathogene Viren)	Robert Koch Institute	
EPI_ISL_7142714, EPI_ISL_7303373, EPI_ISL_7485193, EPI_ISL_7519924, EPI_ISL_7536363	Rosalind Franklin Laboratory	Wellcome Sanger Institute for the COVID-19 Genomics UK (COG-UK) Consortium	Cordelia Langford; David K. Jackson; Dominic Kwiatkowski; Donald Fraser; Ewan Harrison; Ian Johnston; Jeffrey Barrett; John Sillitoe on behalf of the Wellcome Sanger Institute COVID-19 Surveillance Team; Rob Howes; Roberto Amato; Sonia Goncalves; Suki Lee; The Rosalind Franklin Laboratory and Alex Alderton
EPI_ISL_7366154	Rush University Medical Center	RIPHL at Rush University Medical Center	Alyse Kittner; Cecilia Chau; Diane Springer; Edith Perez; Felix Araujo Perez; Hannah Barbian; Joyce Houlihan; Kevin Kunstman; Laura Furtado; Marieta Hyde; Mary Hayden; Sofiya Bobrovska; Stefan Green
EPI_ISL_7545637, EPI_ISL_7545638, EPI_ISL_7545639, EPI_ISL_7545640, EPI_ISL_7545641, EPI_ISL_7545642, EPI_ISL_7545643, EPI_ISL_7545644, EPI_ISL_7545645, EPI_ISL_7545646, EPI_ISL_7545647, EPI_ISL_7545648, EPI_ISL_7545649, EPI_ISL_7545650			
see above	SAMRC	CERI, Centre for Epidemic Response and Innvoation, Stellenbosch University and KRISP, KZN Research Innovation and Sequencing Platform, UKZN.	Arisha Maharaj; Giandhari J; MRC; Naidoo Y; Pillay S; Ramphal U; Ramphal Y; San JE; Tegally H; Tshiabuila D; Wilkinson E; de Oliveira T
EPI_ISL_6913953, EPI_ISL_6914908, EPI_ISL_7194610	SARS-CoV-2 testing team, National Institute of Infectious Diseases	Pathogen Genomics Center, National Institute of Infectious Diseases	Hazuka Y Furihata; Kentaro Itokawa; Makoto Kuroda; Masanori Hashino; Masumichi Saito; Naomi Nojiri; Nozomu Hanaoka; Rina Tanaka; Tsuguto Fujimoto; Tsuyoshi Sekizuka
EPI_ISL_7458718, EPI_ISL_7458719, EPI_ISL_7458720, EPI_ISL_7458721	SK-Roy Romanow Provincial Laboratory	Saskatchewan - Roy Romanow Provincial Laboratory (RRPL)	Alanna Senecal; Amanda Lang; Jessica Minion; Kara Loos; Keith MacKenzie; Meredith Faires; Rachel DePaulo; Roy Romanow Provincial Laboratory - Molecular Diagnostics; Ryan McDonald
EPI_ISL_7334889, EPI_ISL_7334890	SUNRISE MEDICAL LABORATORIES	Wadsworth Center, New York State Department of Health	Alexis Russell; Catharine Prussing; Daryl M. Lamson; Erasmus Schneider; Erica Lasek-Nesselquist; John Kelly; Jonathan Plitnick; Kirsten St. George; Matthew Shudt; Melissa A Leisner; Navjot Singh
EPI_ISL_7195727	SYNLAB	GIGA Medical Genomics	Bouchra Boujemla; Claire Gourzonès; Cécile Meex; Keith Durkin; Laurent Gillet; Maria Artesi; Marie-Pierre Hayette; Nadine Cambisano; Nathalie Renotte; Olivier Ek; Sébastien Bontems; Vincent Bours
EPI_ISL_7440440, EPI_ISL_7442466	SYNLAB MVZ Weiden	Robert Koch Institute	
EPI_ISL_7565722, EPI_ISL_7565723	Salud Digna	Instituto Nacional de Medicina Genomica	Abraham Campos-Romero; Cedro-Tanda A; Cruz-Islas Jazmin; Escobar-Arrazola MA; Garnica-Lopez Dora; Herrera-Montalvo LA.; Hidalgo-Miranda A; Luna-Ruiz Marco; Mendoza-Vargas A; Moreno-Camacho José Luis; Ramirez-Vega O; Rangel-DeLeon D; Reyes-Grajeda JP; Rodriguez-Gallegos Jorge; Yair Alfaro-Mora
EPI_ISL_7200823	Salzkammergutklinikum Vöcklabruck, Institut für Pathologie	Salzkammergutklinikum Vöcklabruck, Institut für Pathologie	Franz Pühringer; Penka Lechner; Regina Stitz; René Silye; Senka Rohregger
EPI_ISL_7015235	Selangor State Health Department	Institute for Medical Research, Infectious Disease Research Centre, National Institutes of Health, Ministry of Health Malaysia	Ahmad FA; Ahmad Fazilah NA; Anasir MI; Azizan MA; Kamel K; Mohamad Sukri MZ; Mohd Zawawi Z; Norhisham SN; Ramly N; Robert F; Rosli NR; Suppiah J; Thayan R
EPI_ISL_7467969	Servicio Virosis Respiratorias-Departamento Virología-INEI	Instituto Nacional Enfermedades Infecciosas C.G.Malbran	Avaro M.; Baumeister E.; Benedetti E.; Campos J.; Cisterna D.; Dattero ME; De Belder D.; Haim MS.; Lorenzo F.; Molina V.; Perandones C.; Poklepovich T.; Pontoriero A.; Russo M.; Sanchez Loria J.; Tuduri E.
EPI_ISL_7625809	Servicio de Microbiologia Hospital Ramon y Cajal	Servicio de Microbiologia Hospital Ramon y Cajal	Galan JC; Martinez L. Abreu M; Ponce M; y Gonzalez-Alba JM
EPI_ISL_6795212, EPI_ISL_6825365, EPI_ISL_7056045, EPI_ISL_7056614, EPI_ISL_7593654, EPI_ISL_7593690, EPI_ISL_7593776, EPI_ISL_7593892, EPI_ISL_7594058, EPI_ISL_7594110, EPI_ISL_7594549, EPI_ISL_7594591, EPI_ISL_7594719, EPI_ISL_7594860, EPI_ISL_7595155, EPI_ISL_7595730, EPI_ISL_7595974, EPI_ISL_7596005, EPI_ISL_7596893, EPI_ISL_7596897, EPI_ISL_7596904, EPI_ISL_7596918, EPI_ISL_7596923, EPI_ISL_7596932, EPI_ISL_7596941, EPI_ISL_7596950, EPI_ISL_7596956, EPI_ISL_7596957, EPI_ISL_7596962, EPI_ISL_7596969, EPI_ISL_7596978, EPI_ISL_7596990, EPI_ISL_7596997, EPI_ISL_7597002, EPI_ISL_7603138, EPI_ISL_7603139, EPI_ISL_7603140, EPI_ISL_7604317, EPI_ISL_7604355, EPI_ISL_7604363, EPI_ISL_7613573, EPI_ISL_7614184, EPI_ISL_7614185, EPI_ISL_7614286, EPI_ISL_7614287			
see above	Shamir Medical Center (Asaf Harofe)	Shamir Medical Center (Asaf Harofe)	Abu Hamad Ramzia; Adina Bar Chaim; Alona Frenkel; Anna Vishnevsky; Chen Weiner; Nir Rainy; Patricia Benveniste-Lekovitz; Reut Sorek Abramovich; Yevgeni Yegorov
EPI_ISL_7062525, EPI_ISL_7405371	Spital Riggisberg	Institute for Infectious Diseases, University of Bern	Alban Ramette; Christian Baumann; Cora Sägesser; Franziska Suter-Riniker; Loïc Borcard; Miguel A Terrazos Miani; Nicole Liechti; Pascal Bittel; Peter Keller; Sonja Gempeler; Stefan Neuenschwander; Stephen L Leib
EPI_ISL_6963509	Sri Jayadeva Institute of Cardiovascular Sciences and Research / Strand Life Sciences	National Centre for Biological Sciences, TIFR - Rockefeller Foundation	Aarati Karaba; Anson Kunjumon George; Aparnaa Ramanathan; Apurva Sarin; Chandrasekhar Vadlamudi; Chitra Pattabiraman; Darshan Sreenivas; Dasaradhi Palakodeti; Dimple Notani; Divya Priya A; Madhusudhan J; Manisha Bharadwaj; Manoj Kumar Jha; Mudasir Nazaar; Pradeep B P; Priyanka Ananta Mulay; Ramesh Hariharan; Rohan Pais; Satyajit Mayor; Saumitra Mardikar; Srivathsan Adimoolam; Uma Ramakrishnan; Vamsi Veeramachaneni; Vasanthapuram Ravi; Vijay Chandru; Vishal G Rao; Yasodha Kannan
EPI_ISL_7265233	St Vincent's Pathology (SydPath)	NSW Health Pathology - Institute of Clinical Pathology and Medical Research; Westmead Hospital; University of Sydney	Arnott A.; Draper J.; Gall M.; Martinez E.; Rockett R.; Sintchenko V.; on behalf of ICPMR

EPI_ISL_7170972	Stadtspital Triemli	Institute of Medical Virology	Alexandra Trkola; Annette Audigé; Cyril Shah; Gabriela Ziltener; Guido Bloemberg; Jon Huder; Jürg Böni; Kevin Steiner; Maria Grünberg; Maryam Zaheri; Michael Huber; Riccarda Capaul; Stefan Schmutz; Verena Kufner
EPI_ISL_6971472, EPI_ISL_7632035, EPI_ISL_7632045	Stadtspital Triemli	Institute of Medical Virology, University of Zurich	Alexandra Trkola; Annette Audigé; Catharine Aquino; Cyril Shah; Daniel Ehrsam; Gabriela Ziltener; Guido Bloemberg; Hubert Rehrauer; Isabel Stürmer; Joel Wirz; Jon Huder; Jürg Böni; Kevin Steiner; Maria Grünberg; Maryam Zaheri; Michael Huber; Riccarda Capaul; Stefan Schmutz; Verena Kufner; Weihong Qi
EPI_ISL_7445855	State Hygienic Laboratory at the University of Iowa	State Hygienic Laboratory at the University of Iowa	Alankar Kampoowale; Anna Yakos; Cindy Toll; Davis Rieckenberg; Erik Twait; Jeff Benfer; Kris Eveland; Krishnaveni Sompallae; Kristen Zanon; Mariah Knutson; Mohammed Allam; Valerie Reeb; Wes Hottel
EPI_ISL_7013425, EPI_ISL_7264087, EPI_ISL_7264088, EPI_ISL_7456351, EPI_ISL_7456393, EPI_ISL_7456394, EPI_ISL_7456395, EPI_ISL_7456396, EPI_ISL_7456397, EPI_ISL_7456398, EPI_ISL_7456399, EPI_ISL_7456400, EPI_ISL_7620798, EPI_ISL_7620819			
see above	State Laboratories Division, Hawaii State Department of Health	State Laboratories Division, Hawaii State Department of Health	Ayana Garnet; Daniel Strange; Drew Kuwazaki; Edward Desmond; Pamela O'Brien; Razvan Sultana
EPI_ISL_7544013, EPI_ISL_7544494, EPI_ISL_7544584, EPI_ISL_7544730	Study: Rapid Diag POC Covid	NHLS/UCT	Arash Iranzadeh; Bruna Galvao; Carolyn Williamson; Deelan Doolabh; Diana Hardie; Gert Marais; Innocent Mudau; Luicer Olubayo; Lynn Tyers; Marvin Hsiao; Nokuzola Mbhele; Rageema Joseph; Stephen Korsman
EPI_ISL_7364700, EPI_ISL_7364701, EPI_ISL_7364702	Summit Clinical Laboratories	City of Milwaukee Health Department Laboratory	Amy Bauer; Manjeet Khubbar; Samantha Scott; Sanjib Bhattacharyya
EPI_ISL_6883250, EPI_ISL_7452246, EPI_ISL_7452247	Swedish national genomic surveillance program of SARS-CoV-2	The Public Health Agency of Sweden	Alma Brolund; Maria Lind Karlberg; Maximilian Riess; Swedish national genomic surveillance program of SARS-CoV-2
EPI_ISL_7457427, EPI_ISL_7457428, EPI_ISL_7457429, EPI_ISL_7457430, EPI_ISL_7457431	Synlab Medilab	Karolinska University Hospital Huddinge	Annika Tiveljung Lindell; Henning Onsbring; Jan Albert; Karina Hentrich; Lynda Eneh; Martin Ekman; Natalija Gerasimcik; Robert Dyrdak; Sandra Broddesson; Shambhu Ganeshappa Aralaguppe; Tanja Normark; Tobias Allander; Valtteri Wirta; Zhibing Yun
EPI_ISL_7605688	TINTSWALO LABORATORY	National Institute for Communicable Diseases of the National Health Laboratory Service	Amoako DG; Bhiman JN; Everatt J; Ismail A; Mahlangu B; Mnguni A; Mohale T; Ntuli N; Scheepers C; Wolter N
EPI_ISL_7605589, EPI_ISL_7605602, EPI_ISL_7605603, EPI_ISL_7605604, EPI_ISL_7605605, EPI_ISL_7605606, EPI_ISL_7605608, EPI_ISL_7605609, EPI_ISL_7605613, EPI_ISL_7605614, EPI_ISL_7605615, EPI_ISL_7605616, EPI_ISL_7605620, EPI_ISL_7605621, EPI_ISL_7605623, EPI_ISL_7605665, EPI_ISL_7605684, EPI_ISL_7605713, EPI_ISL_7605746, EPI_ISL_7605747, EPI_ISL_7605766			
see above	TSHEPONG LABORATORY	National Institute for Communicable Diseases of the National Health Laboratory Service	Amoako DG; Bhiman JN; Everatt J; Ismail A; Mahlangu B; Mnguni A; Mohale T; Ntuli N; Scheepers C; Wolter N
EPI_ISL_7605805	TXDSHS	TXDSHS	Anita Pokharel; Bonnie Oh; Chun Wang; Grace Kubin; Karen Bobier; Maliha Rahman; Mayela Pedrueza; Rachel Lee; Rashmi Tuladhar
EPI_ISL_7456440	Tambo Memorial Laboratory	National Institute for Communicable Diseases of the National Health Laboratory Service	Amoako DG; Bhiman JN; Everatt J; Ismail A; Mahlangu B; Mnguni A; Mohale T; Ntuli N; Scheepers C; Wolter N
EPI_ISL_7571540	Temporary Specimen Collection Centre at the AsiaWorld-Expo	Hong Kong Department of Health	Alan K.L. Tsang; Edman T.K. Lam; Ken H.L. Ng; Patricia K. L. Leung; Peter C.W. Yip; Rickjason C.W. Chan
EPI_ISL_6825551	Territory Pathology	Territory Pathology	Dimitrios Menouhos; Ella Meumann; Robert Baird
EPI_ISL_7171744	The Hope Clinic of Emory Vaccine Center, Emory University	Piantadosi Lab, Emory Department of Pathology	Anne Piantadosi; Azmain Taz; Dara Khosravi; Ethan Wang; Jesse Waggoner; Ludy Carmola; Marybeth Sexton; Nadine Rouphael
EPI_ISL_7446810	Thüringer Landesamtes für Verbraucherschutz	Robert Koch Institute	
EPI_ISL_7235629	Tulane University School of Medicine	Tulane University School of Medicine	Di Tian
EPI_ISL_7505962	UC Davis Genome Center	UC Davis Genome Center	Healthy Davis Together; UC Davis
EPI_ISL_7451261	UNILABS	Instituto Nacional de Saude (INSA)	Borges et al
EPI_ISL_7160037, EPI_ISL_7160038, EPI_ISL_7160039, EPI_ISL_7545421, EPI_ISL_7545422, EPI_ISL_7545423, EPI_ISL_7545424			
see above	UW Virology Lab	UW Virology Lab	Alexander Greninger; Hong Xie; Isabel Arnould; Keith R Jerome; Meei-Li Huang; Nathan Breit; Patrick Mathias; Pavitra Roychoudhury; Pooneh Hajian; Ricardo Perez; Robert J. Livingston; Saraswathi Sathees; Sean Ellis; Seffir T. Wendm; Shah Mohamed Bakhash; Tien V. Nguyen
EPI_ISL_7544861, EPI_ISL_7631788	Unilabs	Institute of Medical Virology, University of Zurich	Alexandra Trkola; Annette Audigé; Catharine Aquino; Cyril Shah; Daniel Ehrsam; Gabriela Ziltener; Guido Bloemberg; Hubert Rehrauer; Isabel Stürmer; Joel Wirz; Jon Huder; Jürg Böni; Kevin Steiner; Maria Grünberg; Maryam Zaheri; Michael Huber; Riccarda Capaul; Stefan Schmutz; Verena Kufner; Weihong Qi
EPI_ISL_7470330, EPI_ISL_7470341	Unilabs Laboratory Medicine	Norwegian Institute of Public Health, Department of Virology	Atiya R Ali; Debech Nadia; Engebretsen Serina Beate; Garcia Llorente Ignacio; Hilde Elshaug; Hilde Vollan; Jon Bråte; Kamilla Heddeland Instefjord; Karoline Bragstad; Kathrine Stene-Johansen; Line Victoria Moen; Marie Paulsen Madsen; Olav Hungnes; Pedersen Benedikte Nevjen; Rasmus Riis Kopperud
EPI_ISL_7141056	Unipath Speciality Laboratory Limited, Ahmedabad	Gujarat Biotechnology Research Centre	Apurvasinh Puvar; Bhadreshsinh Gohil; Chaitanya Joshi; Dinesh Kumar; Janvi Raval; Jwalant Shah; Madhvi Joshi; Nimesh Patel; Nitin Savaliya; Nitin Shukla; Priyank Chavda; Ramesh Pandit; Sonal Sharma; Zarna Patel
EPI_ISL_7613414	University Hospital, San Antonio	STRL UT Health San Antonio, Greehey Children's Cancer Research Institute	Bethany Landry; Dawn Garcia; Guillermo Nunez; Hongxin Fan; Josefina Stoever; Korri Weldon; Kumari Vadlamudi; Marjorie Parker David; San Antonio Metropolitan Health District; Texas Department of State Health Services; Weijing He; Yidong Chen; Zhao Lai; Zhenqing Ye
EPI_ISL_7605542, EPI_ISL_7605543, EPI_ISL_7605546, EPI_ISL_7605547	University Hospitals of Geneva, Laboratory of Virology	HUG, Laboratory of Virology and the Health2030 Genome Center	Aline Mamin; Ana Rita Goncalves; Cedric Howald; Deborah Penet; Francisco Perez; Henri Pegeot; Ioannis Xenarios; Keith Harshman; Laurent Kaiser; Lorenzo Cerutti; Melyssa Elies; Samuel Cordey
EPI_ISL_7542255	University Medical Center Hamburg Eppendorf	Heinrich Pette Institute, Leibniz Institute for Experimental Virology	Adam Grundhoff; Alexis Robitaille; Johannes Knobloch; Martin Aepfelbacher; Nicole Fischer; Thomas Günther
EPI_ISL_6929785, EPI_ISL_7195724, EPI_ISL_7195725, EPI_ISL_7195726, EPI_ISL_7405404, EPI_ISL_7405405, EPI_ISL_7405406, EPI_ISL_7405407, EPI_ISL_7405408, EPI_ISL_7405409, EPI_ISL_7405412, EPI_ISL_7462253, EPI_ISL_7462258, EPI_ISL_7462265, EPI_ISL_7462271, EPI_ISL_7601672			
see above	University of Liège COVID-19 testing center	GIGA Medical Genomics	Bouchra Boujemla; Claire Gourzonès; Cécile Meex; Keith Durkin; Laurent Gillet; Maria Artesi; Marie-Pierre Hayette; Nadine Cambisano; Nathalie Renotte; Olivier Ek; Sébastien Bontems; Vincent Bours
EPI_ISL_7379462	University of Wisconsin-Madison AIDS Vaccine Research Laboratories	University of Wisconsin-Madison AIDS Vaccine Research Laboratories	Gage Moreno; Katarina Braun; et al. AIDS Vaccine Research Laboratories
EPI_ISL_7605921, EPI_ISL_7605979	Universität Zürich	Institute of Medical Virology, University of Zurich	Alexandra Trkola; Annette Audigé; Catharine Aquino; Cyril Shah; Daniel Ehrsam; Gabriela Ziltener; Guido Bloemberg; Hubert Rehrauer; Isabel Stürmer; Joel Wirz; Jon Huder; Jürg Böni; Kevin Steiner; Maria Grünberg; Maryam Zaheri; Michael Huber; Riccarda Capaul; Stefan Schmutz; Verena Kufner; Weihong Qi
EPI_ISL_7544862, EPI_ISL_7544872, EPI_ISL_7631828	UniversitätsSpital Zürich	Institute of Medical Virology, University of Zurich	Alexandra Trkola; Annette Audigé; Catharine Aquino; Cyril Shah; Daniel Ehrsam; Gabriela Ziltener; Guido Bloemberg; Hubert Rehrauer; Isabel Stürmer; Joel Wirz; Jon Huder; Jürg Böni; Kevin Steiner; Maria Grünberg; Maryam Zaheri; Michael Huber; Riccarda Capaul; Stefan Schmutz; Verena Kufner; Weihong Qi
EPI_ISL_7418452, EPI_ISL_7418453, EPI_ISL_7418454, EPI_ISL_7418455, EPI_ISL_7418456, EPI_ISL_7418463, EPI_ISL_7418464, EPI_ISL_7418465			
see above	Universitätsklinikum Frankfurt - Institut für Medizinische Virologie	Robert Koch Institute	
EPI_ISL_7136300, EPI_ISL_7136771, EPI_ISL_7336152, EPI_ISL_7503247	Utah Public Health Laboratory	Utah Public Health Laboratory	Erin L. Young; John Arnn; Kelly F. Oakeson; Olinto Linares-Perdomo; Pooja Gupta; Tom Iverson
EPI_ISL_7261603	VA Tampa Healthcare System	VHA Public Health Reference Laboratory	Mark Holodniy on behalf of VA SEQFORCE; US Department of Veterans Affairs
EPI_ISL_6989662	Vault Health	Minnesota Department of Health, Public Health Laboratory	Alyssa Mondelli; Elizabeth Horn; Jacob Garfin; Kelly Pung; Matt Plumb; Sarah Namugenyi; and Xiong Wang
EPI_ISL_7398681, EPI_ISL_7398758	Vichaivej International Hospital Nongkhaem	National Institute of Health, Department of Medical Sciences, Ministry of Public Health, Thailand	Archawin Rojanawiwat; Ballang Uppapong; Beth Skaggs; Donlaya Maunplueng; Kazuhisa Okada; Natchaya Khiadsang; Nuttida Thongpramul; Pakorn Piromtong; Pilailuk Akkapaiboon Okada; Piroon Jenjaroenpun; Pongpun Sawatwong; Prapat Suriyaphol; Sirikanda Wimol; Siripaporn Phuygun; Sittiporn Parnmen; Supakit Sirilak; Suratchana Mitrat; Thanutsapa Thanadachakul; Thidathip Wongsurawat
EPI_ISL_7224567, EPI_ISL_7224577	Viollier AG	Clinical Bacteriology, University Hospital Basel	Adrian Egli; Alfredo Mari; Christiane Beckmann; Fanny Wegner; Hans Hirsch; Helena MB Seth-Smith; Julia Bielicki; Karoline Leuzinger; Manuel Battegay; Tim Roloff
EPI_ISL_7371749, EPI_ISL_7371751, EPI_ISL_7372207, EPI_ISL_7566519, EPI_ISL_7566755, EPI_ISL_7566802, EPI_ISL_7566826, EPI_ISL_7566947, EPI_ISL_7620490, EPI_ISL_7620538, EPI_ISL_7620551, EPI_ISL_7620714, EPI_ISL_7620766			
see above	Viollier AG	Department of Biosystems Science and Engineering, ETH Zürich	Andrea Patrignani; Andrea Patrizia Salzmann; Andreia Cabral de Gouvea; Catharine Aquino; Catharine Aquino Fournier; Chaoran Chen; Christian Beisel; Christian Urban; Christiane Beckmann; Christoph Noppen; Daniel Ehrsam; David Dreifuss; Doris Popovic; Elodie Burcklen; Franziska Singer; Griffin White; Hai Bui; Henriette Kurth; Ina Nissen; Isabel Stürmer; Ivan Topolsky; Jay Tracy; Kim Philipp Jablonski; Lara Fuhrmann; Laura Neff; Lennart Opitz; Louis du Plessis; Maria Domenica Moccia; Matteo Carrara; Maurice Redondo; Mirjam Feldkamp; Natascha Santacroce; Niko Beerenwinkel; Olivier Kobel; Pelin Icer; Ralph Schlapbach; Rebecca Denes; Sarah Nadeau; Shuqing Yu; Simon Grüter; Tanja Stadler; Timothy Sykes; Tobias Schär

EPI_ISL_7509012, EPI_ISL_7509019, EPI_ISL_7509025, EPI_ISL_7509030, EPI_ISL_7561327, EPI_ISL_7561395, EPI_ISL_7561411, EPI_ISL_7561414, EPI_ISL_7561423, EPI_ISL_7561425, EPI_ISL_7561434, EPI_ISL_7561441, EPI_ISL_7561446, EPI_ISL_7561454, EPI_ISL_7561464, EPI_ISL_7561472, EPI_ISL_7561477, EPI_ISL_7561482			
see above	Virology Department, Royal Infirmary of Edinburgh, NHS Lothian / School of Biological Sciences, University of Edinburgh	COVID-19 Genomics UK (COG-UK) Consortium	Colquhoun R; Cotton S; Dewar R; Fernandez G; Gallagher A; Hill V; Jackson B; Maloney D; McCrone JT; McHugh M; O'Toole Á; Rambaut A; Scher E; Templeton K; Yu X
EPI_ISL_7285844, EPI_ISL_7285845, EPI_ISL_7285846, EPI_ISL_7285847, EPI_ISL_7285848	Virology Laboratory, Scientific Department, Army Medical Center	Virology Laboratory, Scientific Department, Army Medical Center	Anella Monte; Anna Anselmo; Antonella Fortunato; Filippo Molinari; Florigio Lista; Francesco Giordani; Giancarlo Petralito; Giandomenico Cerreto; Giulia Campoli; Lucia Nicosia; Marzia Cavalli; Pietro Marco D'Angelo; Riccardo De Sanctis; Rossella Brandi; Silvia Fillo; Vanessa Vera Fain
EPI_ISL_7503508, EPI_ISL_7503509	Washington State Department of Health Public Health Laboratories	Washington State Department of Health Public Health Laboratories	Alex Lathum; Ardizon Valdez; Avi Singh; Claire Howell; Denny Russell; Drew MacKellar; Holly Halstead; JohnAric Peterson; Kathryn Sickles; Kristin Roche; Lisa Jones; Philip Dykema; Rebecca Cao
EPI_ISL_7548944, EPI_ISL_7548945	Wexner Medical Center	OSU College of Medicine	Corcoran, S.; Koenig, S.
EPI_ISL_7501188	Winchester Hospital via Lahey Hospital	New England Biolabs	Abel, G.; B.W.; C.J.; Colgrove, R.; Duncan, R.; Elfahal, M.; Flynn; Heim, K.; Karolides, M.; L. and Langhorst; Michaels, L.; Pinet, K.; Skelton, T.; Sun
EPI_ISL_7263803	Wisconsin State Laboratory of Hygiene Communicable Disease Division	Wisconsin State Laboratory of Hygiene Communicable Disease Division	Abigail C. Shockey; Alicia J. Mooney; Erika M. Hanson; Kelsey R. Florek; Richard Griesser; Sara Wagner; Tonya Danz
EPI_ISL_7478524, EPI_ISL_7478525, EPI_ISL_7478526, EPI_ISL_7478527, EPI_ISL_7478529, EPI_ISL_7478530, EPI_ISL_7502154, EPI_ISL_7502155			
see above	Yale Clinical Virology Lab	Grubaugh Lab - Yale School of Public Health	Anderson Brito; Chaney Kalinich; Chantal Vogels; Isabel Ott; Joseph Fauver; Kendall Billig; Mallery Breban; Marie L. Landry; Mary Petrone; Nathan Grubaugh; Tobias Koch
EPI_ISL_7472293	Yale Pathology Labs	Yale Pathology Labs	Angelique Levi; Brian Daley; Chen Liu; Guangxiao Yang; Heidi Herrick; Jianhui Wang; Jinglan Wang; John Sinard; Katherine Fajardo; Kevin Schofield; Laura Brady; Michael Stankewich; Minghao Zhong; Monica Talmor; Pei Hui; Peter Gershkovich; Richard Bouffard; Stephanie Weirsman; Susan Bell; Sylvia White
EPI_ISL_6795833, EPI_ISL_6795834, EPI_ISL_6795835, EPI_ISL_6795836, EPI_ISL_6795837, EPI_ISL_6795838, EPI_ISL_6795839, EPI_ISL_6795840, EPI_ISL_6795841, EPI_ISL_6795842, EPI_ISL_6795844, EPI_ISL_6795845, EPI_ISL_6795846, EPI_ISL_6795847, EPI_ISL_6795848, EPI_ISL_6795849, EPI_ISL_6795850, EPI_ISL_6825389, EPI_ISL_6825390, EPI_ISL_6825391, EPI_ISL_6825392, EPI_ISL_6825393, EPI_ISL_6825394, EPI_ISL_6825395, EPI_ISL_6825396, EPI_ISL_6825397, EPI_ISL_6825398, EPI_ISL_7015172, EPI_ISL_7015173, EPI_ISL_7015174, EPI_ISL_7015175, EPI_ISL_7015176, EPI_ISL_7015177, EPI_ISL_7015178, EPI_ISL_7015179, EPI_ISL_7015180, EPI_ISL_7015181, EPI_ISL_7015182, EPI_ISL_7015183, EPI_ISL_7015184, EPI_ISL_7015185, EPI_ISL_7015186, EPI_ISL_7015187, EPI_ISL_7015188, EPI_ISL_7015189, EPI_ISL_7015190, EPI_ISL_7015191, EPI_ISL_7015192, EPI_ISL_7015193, EPI_ISL_7015194, EPI_ISL_7015195, EPI_ISL_7015196, EPI_ISL_7015197, EPI_ISL_7015198, EPI_ISL_7015199, EPI_ISL_7015200, EPI_ISL_7015201, EPI_ISL_7015202, EPI_ISL_7015203, EPI_ISL_7015204, EPI_ISL_7015205, EPI_ISL_7015206, EPI_ISL_7015207, EPI_ISL_7015208			
see above	ZARV/NHLS, Department Medical Virology, University of Pretoria	CERI, Centre for Epidemic Response and Innvoation, Stellenbosch University and KRISP, KZN Research Innovation and Sequencing Platform, UKZN.	Adriano Mendes; Amoaka D; Amy Strydom; Arisha Maharaj; Bester P; Bhiman J; Engelbrecht S; Everatt J; Giandhari J; Goedhals D; Hardie D; Hsiao M; Iranzadeh A; Lessells R; Makatini Z; Maponga T; Mdlalose N; Micheala Davids; Mlisana K; Moir M; NGS-SA (Scheepers C; Naidoo Y; Nyaga M) Giandhari J; Oluwakemi M; Pillay S; Preiser W; Ramphal U; Ramphal Y; San JE; Sim Mayaphi and Marietjie Venter; Tegally H; Tshiabuila D; Venter M; Wilkinson E; Williamson C; de Oliveira T; von Gottberg A
EPI_ISL_7548959, EPI_ISL_7548966, EPI_ISL_7549083	ZOTZ KLIMAS MVZ Düsseldorf-Centrum GbR ÜBAG für Labormedizin, Genetik, Zytologie, Pathologie	Center of Medical Microbiology, Virology, and Hospital Hygiene, University of Duesseldorf	Alexander Dilthey; Andreas Walker; Daniel Strelow; Jessica Nicolai; Jörg Timm; Katrin Hoffmann; Klaus Pfeffer; Lisanna Hülse; Malte Kohns Vasconcelos; Maximilian Damagnez; Nadine Lübke; Patrick Finzer; Rainer Zotz; Tobias Wienemann; Torsten Houwaart
EPI_ISL_7544865	Zentrallabor Zürich	Institute of Medical Virology, University of Zurich	Alexandra Trkola; Annette Audigé; Cyril Shah; Gabriela Ziltener; Guido Bloemberg; Jon Huder; Jürg Böni; Kevin Steiner; Maria Grünberg; Maryam Zaheri; Michael Huber; Riccarda Capaul; Stefan Schmutz; Verena Kufner
EPI_ISL_7423603	amedes MVZ für Laboratoriumsdiagnostik Raubling GmbH	Robert Koch Institute	
EPI_ISL_7368223, EPI_ISL_7594806	labor team w AG	Department of Biosystems Science and Engineering, ETH Zürich	Andrea Patrignani; Andreas Lindauer; Andreia Cabral de Gouvea; Catharine Aquino; Catharine Aquino Fournier; Chaoran Chen; Christian Urban; Daniel Ehrsam; David Dreifuss; Doris Popovic; Franziska Singer; Griffin White; Hai Bui; Isabel Stürmer; Ivan Topolsky; Jay Tracy; Kim Philipp Jablonski; Lara Fuhrmann; Laura Neff; Lennart Opitz; Louis du Plessis; Maria Domenica Moccia; Matteo Carrara; Monika Bucher; Niko Beerenwinkel; Pelin Icer; Ralph Schlapbach; Rebekka Pohl; Sarah Nadeau; Shuqing Yu; Simon Grüter; Tanja Stadler; Timothy Sykes
